# Meta-Analysis of Preclinical Studies of Fibrinolytic Therapy for Acute Lung Injury

**DOI:** 10.3389/fimmu.2018.01898

**Published:** 2018-08-20

**Authors:** Cong Liu, Yana Ma, Zhenlei Su, Runzhen Zhao, Xiaoli Zhao, Hong-Guang Nie, Ping Xu, Lili Zhu, Mo Zhang, Xiumin Li, Xiaoju Zhang, Michael A. Matthay, Hong-Long Ji

**Affiliations:** ^1^Institute of Lung and Molecular Therapy, Xinxiang Medical University, Xinxiang, China; ^2^Department of Molecular and Cellular Biology, University of Texas Health Science Center at Tyler, Tyler, TX, United States; ^3^Department of Physiological Sciences, Eastern Virginia Medical School, Norfolk, VA, United States; ^4^Institute of Metabolic Disease Research and Drug Development, China Medical University, Shenyang, China; ^5^School of Nursing, Xinxiang Medical University, Xinxiang, China; ^6^Department of Pulmonary Medicine, Henan Provincial People’s Hospital, Zhengzhou, China; ^7^Department of Anesthesia and Medicine, University of California, San Francisco, San Francisco, CA, United States

**Keywords:** lung diseases, fibrinolytic agents, molecular therapy, interventions, preclinical study

## Abstract

**Background:**

Acute lung injury (ALI) is characterized by suppressed fibrinolytic activity in bronchoalveolar lavage fluid (BALF) attributed to elevated plasminogen activator inhibitor-1 (PAI-1). Restoring pulmonary fibrinolysis by delivering tissue-type plasminogen activator (tPA), urokinase plasminogen activator (uPA), and plasmin could be a promising approach.

**Objectives:**

To systematically analyze the overall benefit of fibrinolytic therapy for ALI reported in preclinical studies.

**Methods:**

We searched PubMed, Embase, Web of Science, and CNKI Chinese databases, and analyzed data retrieved from 22 studies for the beneficial effects of fibrinolytics on animal models of ALI.

**Results:**

Both large and small animals were used with five routes for delivering tPA, uPA, and plasmin. Fibrinolytics significantly increased the fibrinolytic activity both in the plasma and BALF. Fibrin degradation products in BALF had a net increase of 408.41 ng/ml vs controls (*P* < 0.00001). In addition, plasma thrombin–antithrombin complexes increased 1.59 ng/ml over controls (*P* = 0.0001). In sharp contrast, PAI-1 level in BALF decreased 21.44 ng/ml compared with controls (*P* < 0.00001). Arterial oxygen tension was improved by a net increase of 15.16 mmHg, while carbon dioxide pressure was significantly reduced (11.66 mmHg, *P* = 0.0001 vs controls). Additionally, fibrinolytics improved lung function and alleviated inflammation response: the lung wet/dry ratio was decreased 1.49 (*P* < 0.0001 vs controls), lung injury score was reduced 1.83 (*P* < 0.00001 vs controls), and BALF neutrophils were lesser (3 × 10^4^/ml, *P* < 0.00001 vs controls). The mortality decreased significantly within defined study periods (6 h to 30 days for mortality), as the risk ratio of death was 0.2-fold of controls (*P* = 0.0008).

**Conclusion:**

We conclude that fibrinolytic therapy may be effective pharmaceutic strategy for ALI in animal models.

## Introduction

Suppressed fibrinolysis is a pathological hallmark of acute lung injury (ALI) in addition to pulmonary edema and cytokine/chemokine “storm” (1, 2). Over the last two decades, the mortality of acute respiratory distress syndrome (ARDS), the late stage of ALI remains unacceptably high. There are approximately 200,000 new cases annually in the United States (3, 4). ALI could be caused by pulmonary (e.g., pneumonia and smoke inhalation) or systemic disorders (e.g., sepsis, hemorrhagic shock, and trauma) (5). The heterogeneity of ALI apparently makes it difficult to identify the etiology precisely and promptly for designing case-specific salutary interventions. To date, supportive strategies have been shown to be beneficial (5). In addition, some promising therapeutic strategies are being evaluated by registered clinical trials, including stem cell therapy (6, 7), corticosteroid, interferon beta, and tumor necrosis factor-alpha (8).

In injured lungs, alveolar fibrinolytic activity is depressed markedly and intravascular and extracellular fibrin is deposited in the air spaces (9, 10). The eliminated fibrinolytic activity was predominately attributed to elevated plasminogen activator inhibitor-1 (PAI-1) in both the plasma and bronchoalveolar lavage fluid (BALF). The fibrinolytic system is composed of proteases and anti-proteases, including plasminogen, plasminogen activators (tissue-type plasminogen activator, tPA; urokinase plasminogen activator, uPA), plasmin, PAI-1, and plasmin catalytic antagonists (α2-antiplasmin and α2-macroglobulin). Both uPA and tPA proteolytically cleave zymogen plasminogen to generate plasmin with catalytic activity, which degrades fibrin. To date, the fibrinolytic therapy has clinically been applied to pleural effusion/empyema as fibrinolysins (11), cardiovascular diseases as thrombolytics (12), and obstructive airway diseases as mucolytics (13, 14). The benefit of fibrinolytic therapy for ALI, however, is still at the earlier stage of preclinical studies. Intravenous delivery of either uPA or tPA might be protective for traumatic lung injury, as improved survival and gas exchange were observed in treated pigs (15). Moreover, tPA attenuated pulmonary abnormalities in smoke inhalation injured sheep (16). Interestingly, an earlier pilot study reported a potential improvement in lung function following administration of either uPA or tPA in 20 ALI patients (17). Because of inconclusive preclinical studies to address optimized dose, routes, and benefit, clinical trials have not been conducted to date.

The main purpose of this meta-analysis is, therefore, to assess preclinical studies of ALI for the potential effects of fibrinolytic therapy. Additionally, we quantified the differences in outcomes between large and small animal models, variable fibrinolytic regimes, and routes. Our analysis suggests that three fibrinolytic regimens may benefit ALI by improving gas exchange, inflammation, and lung injury.

## Materials and Methods

The study was conducted in accordance with the methods recommended in the PRISMA guidelines. Please see Datasheet S1 in Supplementary Material for detail search strategies.

### Data Sources

Three investigators (CL, ZS, and YM) independently searched the published studies indexed by the PubMed, Embase, Web of Science, and CNKI on March 2017, using the strategy: (fibrinolytics OR plasmin OR fibrinase OR fibrinolysin OR alfimeprase OR uPA OR urokinase OR abbokinase OR breokinase OR win-kase OR kinlytic OR tPA OR activase OR reteplase OR rctPA OR retavase OR actilyse OR repilysin OR alteplase OR tenecteplase OR TNKase OR TNK tPA OR metalyse OR SK OR streptokinase OR streptase OR kabikinase OR actase OR thrombolysin OR eminase OR desmoteplase OR pro-urokinase OR staphylokinase OR plasminogen activator) AND (lung injury OR ALI OR ARDS OR respiratory distress syndrome OR septic OR sepsis OR bacteremia OR endotoximia OR multi-organ failure OR respiratory failure OR pneumonia OR shock OR pulmonary edema OR lung edema OR edematous OR pulmonary edema). The fourth investigator (H-LJ) was consulted in case of no consensus on inclusion. There was no language restriction in the searching and non-English literature was translated into English *via* a professional service. We also checked the references of included studies for additional publications that were not hit by the searching strategy.

### Inclusion and Exclusion Criteria

Studies were included in the current meta-analysis if: (1) the species of studies were animals; (2) animal models were ALI, including *Pseudomonas aeruginosa* pneumonia, *Klebsiella pneumonia*, fibrotic lung injury, trauma, septic shock, burn and smoke inhalation injury, and disseminated intravascular coagulation; (3) the animals were treated with three fibrinolytics, genetically engineered for overexpressing of plasminogen activators or knocking out PAI-1; and (4) results were expressed or could be digitized or converted to mean and SD.

The following studies were excluded: (1) review articles, letters, case reports, posters, or without objective data to be evaluated; (2) data were from PAI-1 transgenic, PAI-1 knock-in, and tPA- and uPA-deficient animals; (3) insufficient publications existed to perform a meta-analysis; (4) the number of control group or experimental group was less than three animals; (5) the animals were treated with combined heparin, antithrombin, activated protein C, and other medicines with potential effects on fibrinolysis; and (6) combined regimens and fibrinolytic therapy for lung injury.

### Data Extraction

Data extraction was carried out by three authors (CL, ZS, and YM). The following items from the eligible studies were extracted: article information (first author name, publication date, and country of origin), animal species, gender, method of ALI induction, type and dose of fibrinolytics, duration of treatment, delivery approach, and number of animals for both control and treated groups. Data were extracted as mean and SD if available. When only graphic presentations were available, values for mean and SD were obtained *via* calibrating images using GetData Graph Digitizer software (version 2.26.0.20) (18–20). If raw data were represented by median and interquartile range (20, 21) or range (22), these values were converted to SD by the formulas: SD^2^ = 1/12 [(a − 2 m + b)^2^/4 + (b − a)^2^], and mean = (a + 2 m + b)/4, where m represents median, a and b are lower and upper range (23). If the SE was reported (20), it was converted to SD using the function, SD = SE ×√n, where *n* is the sample size. For one study (24), we combined individual data using the formulas, $\overset{¯}{X}=\frac{\Sigma X}{n}$ and $\text{SD}=\surd \frac{\Sigma (X-\bar{X})}{n-1}$, where *x* represent variance, $\bar{X}$ is the mean of the pooled individual data. If neither SD nor SEM were found (15, 25), we borrowed SD value of similar studies for the same parameter. For parameters reported with divergent units, for example, PaO_2_, PaCO_2_, and neutrophils, the units were converted to the same one. For lung water content that shown as weight gain or lung leak index, we converted them to W/D ratio following the formulas: W/D ratio = (W_0_ + W_1_)/D_0_ = lung leak index × 100, where W_0_ represents normal animal lung wet weight, W_1_ represents weigh gain, and D_0_ represents normal animal lung dry weight. Lung injury scores were analyzed as occurrence of at least one of following indices: (1) leukocyte and red blood cell infiltration, (2) alveolar epithelium damage, (3) interstitial edema, and (4) fibrin deposition and hyaline membrane formation. Extracted raw data are included in Datasheet S2 in Supplementary Material.

### Quality Assessment

Risk of biases was assessed using the Cochrane Handbook for Systematic Reviews of Interventions (26). All included studies were assessed on seven fronts: randomization (selection bias), blinding of personnel (performance bias), blinding of outcome assessment (detection bias), incomplete outcome data (attrition bias), allocation concealment (selection bias), selective outcome reporting (reporting bias), and other biases. These assessments are included in Table 6. In addition, we examined the quality of the included 22 studies with the ARRIVE guidelines specifically for animal studies (Table 2).

### Statistical Analysis

We performed statistical analysis in the preclinical studies using Review Manager (RevMan), version 5.3 (Copenhagen: The Nordic Cochrane Centre, The Cochrane Collaboration, 2014) and STATA V.12 (StataCorp. College Station, TX, USA). The mean differences were considered to be statistically significant when *P* ≤ 0.05. If *P*-value was less than 0.05, or the *I*^2^-value was greater than 50%, the overall estimate was analyzed in a random effects model. Groups within a parameter were included if there were ≥3 comparisons. Eleven readouts were selected: PaO_2_, PaCO_2_, plasma thrombin–antithrombin complexes (TATc), plasma PAA, fibrin degradation products (FDP) in BALF, PAA in BALF, PAI-1 in BALF, neutrophils in BALF, lung water content, lung injury score, and mortality. The data were combined using inverse variance method and shown as weighted mean differences (WMD) with 95% confidence intervals (95% CI) in the forest plots in addition to mortality. The measures for the parameters (PaO_2_ and PaCO_2_) at the same time points were continuously monitored. If unavailable, we used the data collected at an adjacent time point. We pooled dichotomous variables (i.e., mortality) using the Mantel–Haenszel method. If the mortality was measured within a period, the end time point was used. The RR was computed with the random effects model for a high heterogeneity. The potential publication bias was assessed with funnel plots and the Egger’s regression test (40) (Stata, version 12). Heterogeneity among studies was defined with the *I*^2^-statistic function as an unimportant (*I*^2^ < 25%), a moderate (25% < *I*^2^ < 75%), or a high degree of heterogeneity (*I*^2^ > 75%). To eliminate heterogeneity, the meta-analyses were further performed for data grouped by animal size (small or large animals), individual fibrinolytics (tPA, uPA, or plasmin), and routes (i.v., i.p., i.t., nebulization, or transgenic). Small animals included mice, rats, and rabbits. Large animals were comprised of sheep, pigs, and dogs. Subgroup analysis was not performed when there was only one sample. In addition, the robustness of the results was confirmed by sensitivity analysis. If multiple studies with the same first author and publication year, they were distinguished with an asterisk (*) (19, 20). The same control group was used for several comparisons and denoted with superscript letters a, b, c, and d (15, 18, 29, 32).

## Results

### Characteristics of Included Studies

**Figure 1:**
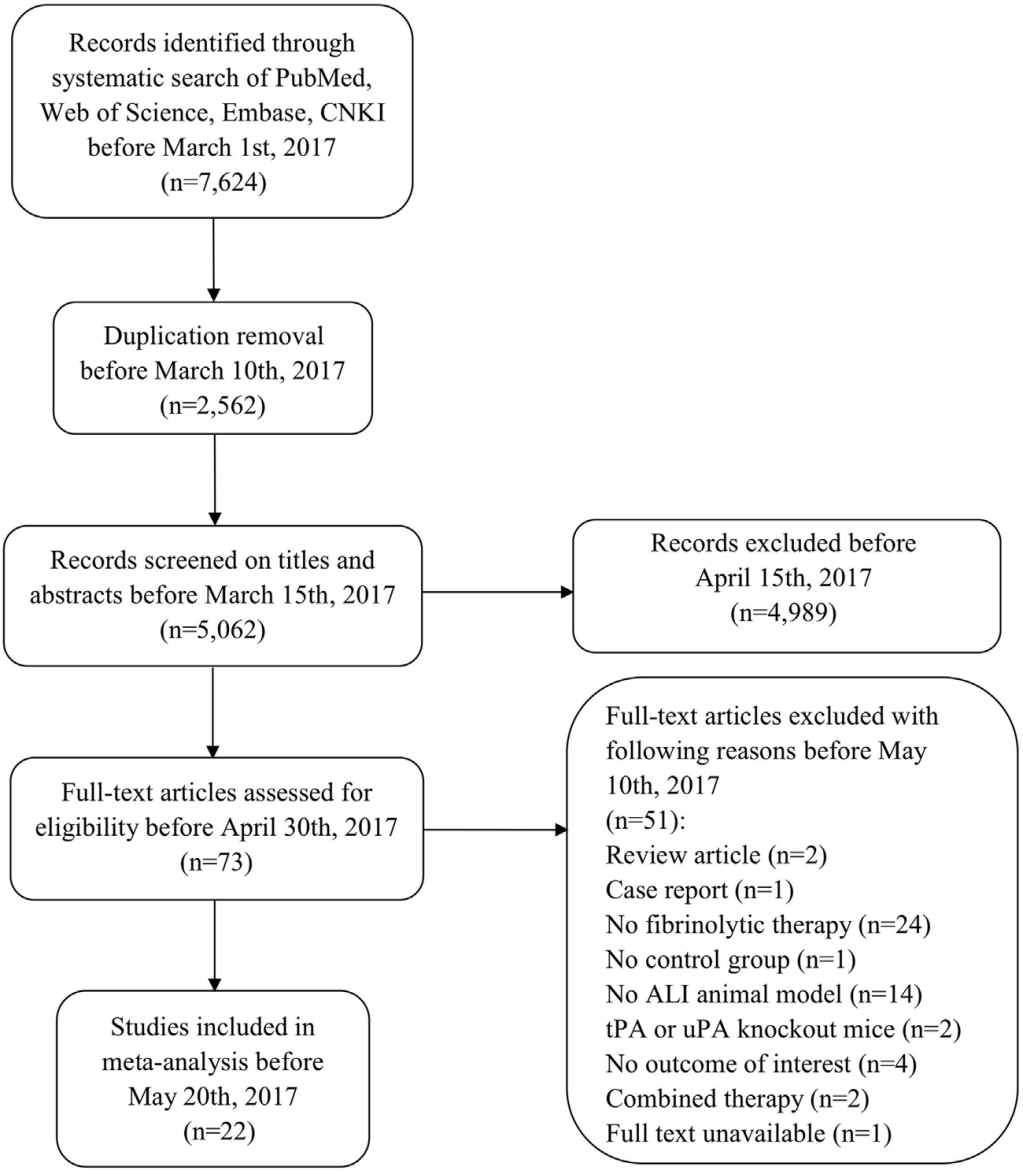
Flow diagram of the literature search and selection.

Our comprehensive search of the PubMed, the Web of Science, the Embase, and the CNKI databases hit 7,624 publications. After carefully examining the titles and abstracts, we selected 73 articles with full texts available for further screening. Twenty-two studies were finally included for systematic review and meta-analysis (Figure 1). General characteristics of these 22 studies were summarized in Table 1. Ten studies were performed for tPA (0.098–250 mg) (15, 16, 19, 20, 22, 24, 27–30), seven for uPA (up to 240 mg or 2,230,000 IU) (18, 21, 25, 32–35), three for plasmin (0.5 mg or 100,000 IU) (37–39), and two in gene therapeutic mice by either over expressing uPA (36) or tPA (31). The geological distributions of these studies are: 10 from USA (48%) (15, 16, 25, 27–30, 32, 36, 37), 4 from Netherlands (17%) (19, 20, 24, 31), 4 from China (17%) (22, 33–35), 2 from Japan (9%) (38, 39), 1 from Germany (4%) (18), and 1 from Denmark (21). Fifteen papers were published in small animals. Twelve studies were conducted in rodents: 2 in mice (31, 36) and 10 in rats (19, 20, 22, 24, 27–30, 38, 39). In addition, 3 studies used rabbits (18, 34, 35). Seven studies used large animals: 5 in pigs (15, 21, 25, 32, 33), 1 in dogs (37), and 1 in sheep (16). Five routes used in the studies included intravenous injection/infusion (i.v., *n* = 12), nebulization (Neb, *n* = 5), intratracheal inhalation (i.t., *n* = 2), intraperitoneal injection (i.p., *n* = 1), and gene delivery (Tg, *n* = 2). To evaluate the strength of the included 22 studies, we checked the adherence to the 20 checklists of the ARRIVE guidelines (Table 2). In general, no papers fully reported 20 checklists, most of studies partially reported these requests except title, objectives, and experimental outcomes. In particular, none of 22 studies performed sample size calculation and provided detailed adverse events. Thus, we examined the quality of the included studies for mortality (Table 3). In total, 200 animals (112 for control and 88 for treated group) from 10 studies were included for meta-analyzing mortality. There were no animals excluded by the original authors due to incident death or severe side-effects. Average mortality was 87 and 17%, respectively, for controls and treated animals. Correspondingly, 7 animals (power = 80%, alpha = 0.05) or 10 animals (power = 95%, alpha = 0.05) are requested. Definitely, the animals for both groups are sufficient for performing meta-analysis. On the other hand, because only 6 of 22 studies simply mentioned paucity of hemorrhage descriptively instead of quantitative measurements, we could not have high quality primary data to analyze this adverse effect.

**Table 1:** General characteristics of included studies.

Author (year), country	Species, gender control/treated	Insult	Total dose	Route	Medicine, vehicle	Time
Enkhbaatar et al., (16), USA	Sheep, F (6/6)	Burn and smoke inhalation	22 mg (2 mg every 4 h, beginning 4 h after injury)	Neb	rhtPA, saline	48 h
Choi et al., (19, 20) ^a,b^, Netherlands	Rat, M (11/8 or 12/8)	LPS	0.281 mg, 0.84 mg (1.25 mg/kg, 30 min before injection of LPS; at 6 and 12 h for group b)	i.v.	rtPA, saline	4 or 16 h
Choi et al., (19, 20)^*,a,b^, Netherlands	Rat, M (8/8)	*Pseudomonas aeruginosa*	0.31 mg, 0.94 mg (1.25 mg/kg, 30 min before induction of pneumonia; at 6 and 12 h for group b)		rtPA, saline	6 or 16 h
Huang et al., (22), China	Rat, M (7/7)	Ventilation	0.34 mg (1.25 mg/kg in 0.5 ml saline, 15 min before ventilation)		tPA, saline	2 h
Stringer et al., (27), USA	Rat, M (10/6)	IL-1	4.2 mg (6 mg/kg, 10 min before IL-1, 6 mg/kg after 2.5 h)	i.p.	tPA, saline	5 h
Hofstra et al., (24) ^a,b^, Netherlands	Rat, M (7/7)	*P. aeruginosa* or LPS	0.84 mg (1.25 mg/kg, 30 min before induction of pneumonia or injection of LPS; at 6 and 12 h)	Neb	rtPA, saline	16 h
Conhaim et al., (28), USA	Rat, M (6/6)	Acute blood loss	0.136 mg (320 μg/kg × 0.325 kg × 1)	Neb	tPA, ipratropium bromide	24 h
Veress et al., (29) ^a,b,c,d^, USA	Rat, M (4/12)	Sulfur mustard Inhalation	0.098, 0.195, 0.325, or 0.455 mg (0.15, 0.30, 0.50, or 0.70 mg/kg × 0.325 kg × 2, at 5.5 and 6.5 h)	i.t.	tPA, PBS	48 h
Veress et al., (30), USA	Rat, M (13/9)		2.06 mg (0.70 mg/kg × 0.245 kg × 12, every 4 h)		tPA, PBS	
Renckens et al., (31), Netherlands	Mice, F (12/12)	*K. pneumoniae*	NR	Tg	htPA DNA, control Ad.	30 days
Hardaway et al., (15) ^b,a^, USA	Pig, NR (9/5)	Trauma	250 mg (initially 50 mg, then 200 mg, beginning 4 h after injury)	i.v.	tPA, NT	48 h
			250,000 IU (250,000 IU in 500 ml 5% glucose solution and administered at 15 drops/min, beginning 4 h after injury)		uPA, NT	
Hardaway et al., (25), USA	Pig, NR (8/8)	*E. coli*	250,000 IU (250,000 IU in 20 ml saline and inject over a 20-min period)		uPA, saline	24 h
Vasquez et al., (32) ^a,b^, USA	Pig, NR (6/6 or 7/7)	*E. coli*	2,230,000 IU (initially 250,000 IU, then 2,000 IU/pound/h for12 h)		uPA, NT	
			250,000 IU (250,000 IU in 20 ml saline over a 20-min period 1 h after *E. coli* infusion)			
Munster et al., (21), Denmark	Pig, NR (14/14)	Trauma	240 mg (5 mg/ml × 4 ml × 12, 5 mg/ml as 12 consecutive nebulizations of 4 ml)	Neb	scuPA, saline	
Chen et al., (33), China	Pig, NR (6/6)	LPS	74,184 IU (initially 4,400 IU/kg in 10 min, then 4,400 IU/kg/h for 2 h)	i.v.	uPA, NT	6 h
Gunther et al., (18) ^a,b^, Germany	Rabbit, NR (9/5 or 9/7)	Bleomycin	6,319 ± 26.5 IU (6,319 ± 26.5 IU × 1) or 6,889 ± 12.3 IU (6,889 ± 12.3 IU × 1)	Neb	rhuPA, NT	28 days
Yu et al., (34), China	Rabbit, M (18/18)	Embolism	45,000 IU (20,000 U/kg × 2.25 kg)	i.v.	uPA, saline	14 days
Chen et al., (35), China	Rabbit, NR (6/6)	Embolism	55,000 IU (18,333 IU in 3 ml saline, 36,667 IU in 5 ml saline)		uPA, saline	12 h
Sisson et al., (36), USA	Mice, NR (10/11)	Bleomycin	NR	Tg	muPA DNA, plasmid	28 days
Hardaway et al., (37), USA	Dog, NR (34/13)	Hemorrhagic shock	100,000 IU	i.v.	Plasmin, NT	48 h
Motoyama et al., (38), Japan	Rat, M (7/7)	Hypoxia reperfusion	0.5 mg			1 h
Motoyama et al., (39), Japan	Rat, M (7/7)					3.5 h

*tPA, tissue-type plasminogen activator; uPA, urokinase plasminogen activator; rhtPA/rhuPA, recombinant human tPA/uPA; scuPA, single-chain uPA; i.v., intravenous injection/infusion; i.p., intraperitoneal injection; i.t., intratracheal inhalation; Neb, nebulization; Tg, transgenic; LPS, lipopolysaccharide; E. coli, Escherichia coli; IL-1, interleukin 1; PBS, phosphate buffered saline; F/M, female/male; Ad., adenoviral vector; NT, not treated; NR, not reported*.

**Table 2:** ARRIVE checklists of included studies.

20 ARRIVE checklists		Fully reported *n*/*N*(% reported)	Partially reported *n*/*N* (% reported)	Not reported *n*/*N*(% reported)
1	Title	22/22 (100)	0/22 (0)	0/22 (0)
2	Abstract	12/22 (54.5)	10/22 (45.5)	0/22 (0)
3	Background	0/22 (0)	21/22 (95.5)	1/22 (4.5)
4	Objectives	21/22 (95.5)	0/22 (0)	1/22 (4.5)
5	Ethical statement	16/22 (72.7)	0/22 (0)	6/22 (27.3)
6	Study design	1/22 (4.5)	20/22 (91)	1/22 (4.5)
7	Experimental procedure	0/22 (0)	22/22 (100)	0/22 (0)
8	Experimental animals	2/22 (9.1)	19/22 (86.4)	1/22 (4.5)
9	Housing and husbandry	0/22 (0)	4/22 (18.2)	18/22 (81.8)
10	Sample size	0/22 (0)	0/22 (0)	22/22 (100)
11	Allocating animals	0/22 (0)	10/22 (45.5)	12/22 (54.5)
12	Experimental outcomes	22/22 (100)	0/22 (0)	0/22 (0)
13	Statistical methods	0/22 (0)	20/22 (90.9)	2/22 (9.1)
14	Baseline data	10/22 (45.5)	0/22 (0)	12/22 (54.5)
15	Numbers analyzed	2/22 (9.1)	1/22 (4.5)	19/22 (86.4)
16	Outcomes and estimation	7/22 (31.8)	14/22 (63.7)	1/22 (4.5)
17	Adverse events	6/22 (27.3)	0/22 (0)	16/22 (72.7)
17a	Details of adverse events	0/22 (0)	0/22 (0)	22/22 (100)
18	Interpretation/scientific implications	0/22 (0)	22/22 (100)	0/22 (0)
19	Generalizability/translation	0/22 (0)	0/22 (0)	22/22 (100)
20	Funding	11/22 (50)	0/22 (0)	11/22 (50)

*N, total number of papers where the item was applicable; n, total number of papers reporting the item*.

**Table 3:** Data quality included for analyzing mortality.

Study	Total number (control/treated)	Included number (control/treated)	Death incidence (control/treated)	Follow-up time (hour or day)
Vasquez et al., (32)^a^	6/6	6/6	6/5	6–8 h
Vasquez et al., (32)^b^	7/7	7/7	4/0	23 h
Hardaway et al., (37)	34/13	34/13	31/5	48 h
Renckens et al., (31)	12/12	12/12	9/4	30 days
Sisson et al., (36)	10/11	10/11	5/1	21 days
Hardaway et al., (25)	8/8	8/8	7/0	24 h
Veress et al., (30)	13/9	13/9	13/0	41.5 h
Hardaway et al., (15)^a^	9/5	9/5	9/0	44 h
Hardaway et al., (15)^b^	9/5	9/5	9/0	44 h
Veress et al., (29)	4/12	4/12	4/0	41.5 h
Total	112/88	112/88	97/15	6 h–30 days

*None of the animals were excluded during the experiments due to severe adverse events. Sample size for both control and treated groups was computed by the online server, the Clincalc (www.clincalc.com/stats/samplesize.aspx) with the settings for two independent study groups for “Study Group Design,” dichotomous for “Primary Endpoint,” 87% mortality for control (group 1), 17% mortality for treated group (group 2), alpha = 0.05, and meta (power) = 80 or 95%. Calculated sample size for both groups is 7 animals (power = 80%) or 10 animals (power = 95%)*.

### Mortality Within Defined Follow-Up Periods

**Figure 2:**
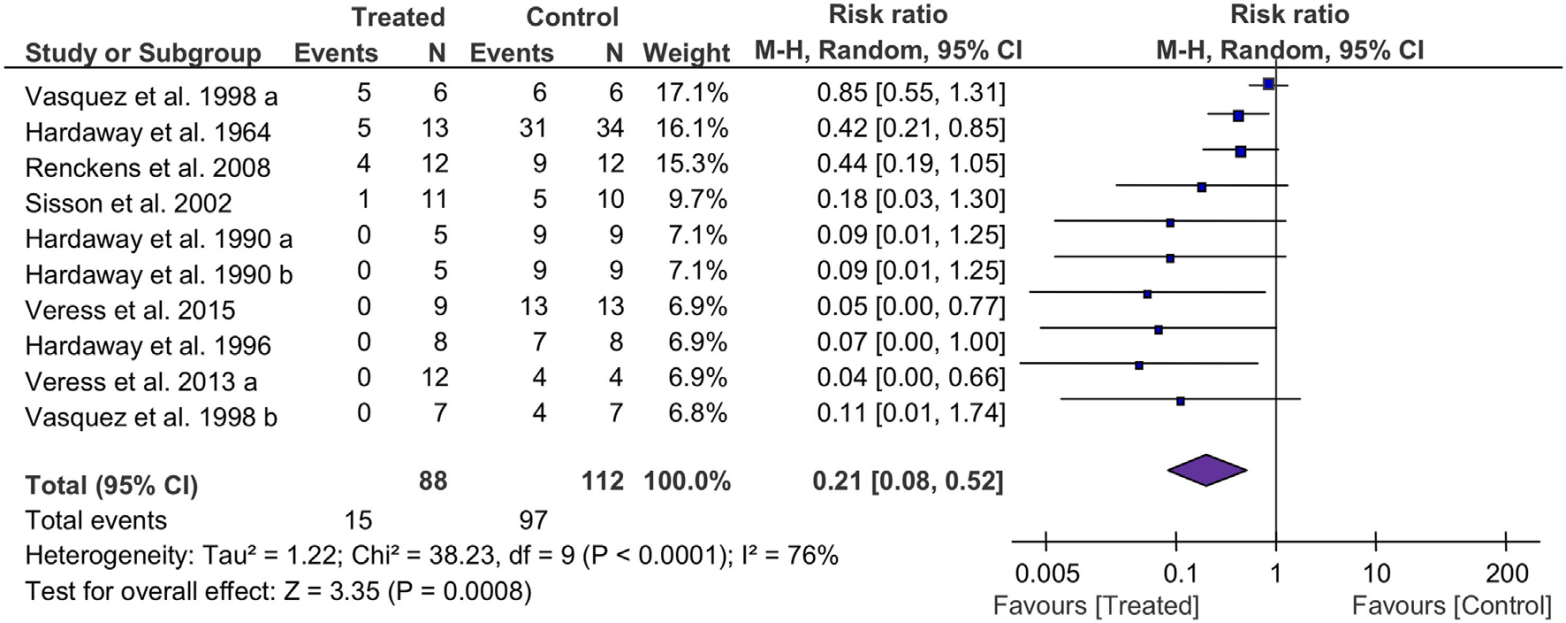
Forest plot summarizing the effect of fibrinolytic therapy on overall mortality of acute lung injury animals. Squares and their sizes represent the risk ratio (RR) and corresponding contributions to overall effect (diamond), respectively. Horizontal lines through each square represent 95% confidence intervals (95% CI). I^2^ depicts heterogeneity.

The mortality associated with fibrinolytic therapy was evaluated (Figure 2). Compared with controls, fibrinolytics significantly reduced the deaths of treated animals, as shown by the overall risk ratio (RR) (0.21, 95% CI: 0.08 to 0.52, *P* = 0.0008). Furthermore, we analyzed mortality of small and large animals separately (Table 4). The mortality was reduced significantly in both large and small animals, as shown by RR values of 0.24 (*P* = 0.02) and 0.17 (*P* = 0.01), respectively. The mortality associated with three fibrinolytics was evaluated individually for tPA (38 controls, 38 treated from 5 studies), uPA (40 controls, 37 treated from 5 studies), and plasmin (34 controls, 13 treated from 1 study). The mortality was reduced in a treatment-dependent manner; the RR values were 0.13 for tPA (*P* = 0.01), 0.18 for uPA (*P* = 0.13), and 0.42 for plasmin (*P* = 0.02). The mortality was further analyzed by three routes: i.v. (73 controls, 44 treated from 6 studies), i.t. (17 controls, 21 treated from 2 studies), and gene delivery (22 controls, 23 treated from 2 studies). The RR values were 0.24 for i.v. (95% CI: 0.07 to 0.82, *P* = 0.02), 0.05 for i.t. (95% CI: 0.01 to 0.32, *P* = 0.002), and 0.38 for gene delivery (95% CI: 0.17 to 0.85, *P* = 0.02). The defined follow-up periods for counting mortality of 10 studies are included in Table 3.

**Table 4:** Summary of analyzed parameters.

	Overall effect	Small animal	Large animal	tPA	uPA	Plasmin	i.v.	Nebulization	i.t.	i.p.	Transgenic
Mortality (RR)	0.21 (0.08, 0.52) 0.0008, 88/112	0.17 (0.04, 0.65) 0.01, 44/39	0.24 (0.07, 0.82) 0.02, 44/73	0.13 (0.03, 0.66) 0.01, 38/38	0.18 (0.02, 1.70) 0.13, 37/40	0.42 (0.21, 0.85) 0.02, 13/34	0.24 (0.07,0.82) 0.02, 44/73	–	0.05 (0.01,0.32) 0.002, 21/17	–	0.38 (0.17, 0.85) 0.02, 23/22
PaO_2_, mmHg	15.16 (7.78, 22.55) <0.0001, 119/138	14.79 (6.28, 23.30) 0.0007, 77/85	21.01 (−25.54, 67.55) 0.38, 42/53	23.41 (6.06, 40.76) 0.008, 32/38	7.68 (2.23, 13.03) 0.005, 73/86	22.74 (−5.72, 51.21) 0.12, 14/14	15.18 (3.59, 26.76) 0.01, 76/84	3.17 (−1.50, 7.83) 0.18, 29/38	30.31 (23.74, 36.89) <0.00001, 14/16	–	–
PaO_2_, mmHg^a^	15.03 (5.63, 24.42) 0.002, 116/104	–	–	–	–	–	–	–	–	–	–
PaCO_2_, mmHg	−11.66 (−17.58, −5.73) 0.0001, 71/80	−14.82 (−23.11, −6.52) 0.0005, 60/66	−0.38 (−1.92, 1.16) 0.63, 11/14	−21.23 (−34.40, −8.05) 0.002, 36/42	0.29 (−1.84, 2.42) 0.79, 35/38	–	0.89 (−1.71, 3.48) 0.50, 31/31	1.36 (−2.60, 5.31) 0.50, 17/20	−31.39 (−44.66, −18.12) <0.00001, 23/29	−	–
PaCO_2_, mmHg^a^	−13.65 (−22.39, −4.92) 0.002, 58/49	–	–	–	–	–	–	–	–	–	–
Plasma PAA,%	38.84 (25.36, 52.31) <0.00001, 46/53	–	–	–	–	–	35.80 (16.40, 55.19) 0.0003, 32/39	44.48 (41.99, 46.98) <0.00001, 14/14	–	–	–
BALF PAA,%	50.53 (45.33, 55.73) <0.00001, 46/53	–	–	–	–	–	48.04 (43.04, 53.04) <0.00001, 32/39	58.27 (32.38, 84.15) <0.0001, 14/14	–	–	–
BALF FDP, ng/ml	408.41 (351.65, 465.16) <0.00001, 60/67	–	–	590.58 (441.78, 739.38) <0.00001, 46/53	–	4.90 (3.12, 6.68) <0.00001, 14/14	392.22 (331.41, 453.02) <0.00001, 46/53	549.03 (48.10, 1049.96) 0.03, 14/14	–	–	–
BALF PAI-1, ng/ml	−21.44 (−23.78, −19.09) <0.00001, 46/53	–	–	–	–	–	−21.12 (−24.54, −17.69) <0.00001	−22.46 (−24.10, −20.82) <0.00001	–	–	–
Plasma TATc, ng/ml	1.59 (0.78, 2.40) 0.0001, 46/53	–	–	–	–	–	1.24 (0.37, 2.12) 0.005, 32/39	3.73 (1.56, 5.89) 0.0007, 14/14	–	–	–
Neutrophil, 10^6^ cells/ml	−0.03 (−0.05, −0.02) <0.00001, 60/68	–	–	−0.08 (−0.16, −0.00) 0.04, 48/50	−0.03 (−0.05, −0.02) <0.00001, 12/18	–	−0.10 (−0.26, 0.07) 0.26, 24/28	−0.04 (−0.06, −0.02) 0.0003, 26/32	–	−0.20 (−1.26, 0.86) 0.71, 10/8	–
Lung water content	−1.49 (−2.15, −0.83) <0.00001, 32/36	−2.09 (−2.72, −1.45) <0.00001, 20/24	−0.60 (−1.26, 0.06) 0.07, 12/12	−2.75 (−6.64, 1.14) 0.17, 12/16	−0.22 (−0.75, 0.31) 0.41, 6/6	−1.84 (−2.38, −1.30) <0.00001, 14/14	−1.35 (−2.10, −0.61) 0.0004, 20/20	−0.90 (−1.13, −0.67) <0.00001, 6/6	–	−4.88 (−6.94, −2.82) <0.00001, 6/10	–
Lung injury score	−1.83 (−2.55, −1.12) <0.00001, 64/64	−1.94 (−2.69, −1.19) <0.00001, 58/58	−1.16 (−1.96, −0.36) 0.004, 6/6	−0.64 (−2.83, 1.55) 0.55, 38/38	−1.67 (−2.48, −0.87) <0.0001, 12/12	−3.50 (−3.61, −3.39) <0.00001, 14/14	−1.92 (−2.65, −1.19) <0.00001, 55/55	–	–	–	−0.36 (−2.81, 2.09) 0.77, 9/9

*RR, risk ratio; tPA, tissue-type plasminogen activator; uPA, urokinase-type plasminogen activator; i.v., intravenous injection/infusion; i.t., intratracheal inhalation; i.p., intraperitoneal injection; PaO_2_, arterial oxygen tension; PaCO_2_, arterial carbon dioxide pressure; PAA, plasminogen activator activity; PAI-1, plasminogen activator inhibitor-1; BALF, bronchoalveolar lavage fluid; FDP, fibrin degradation products; TATc, thrombin–antithrombin complexes. Weighted mean differences, 95% CI in brackets, and p values and animal numbers for treated/control groups are listed on three rows for each parameter. Analyzed results from grouped data are filled with blue, orange, and green for animal size, fibrinolytics, and routes, respectively*.

*^a^Analyzed with endpoint data*.

### Levels of PaO_2_ and PaCO_2_

**Figure 3:**
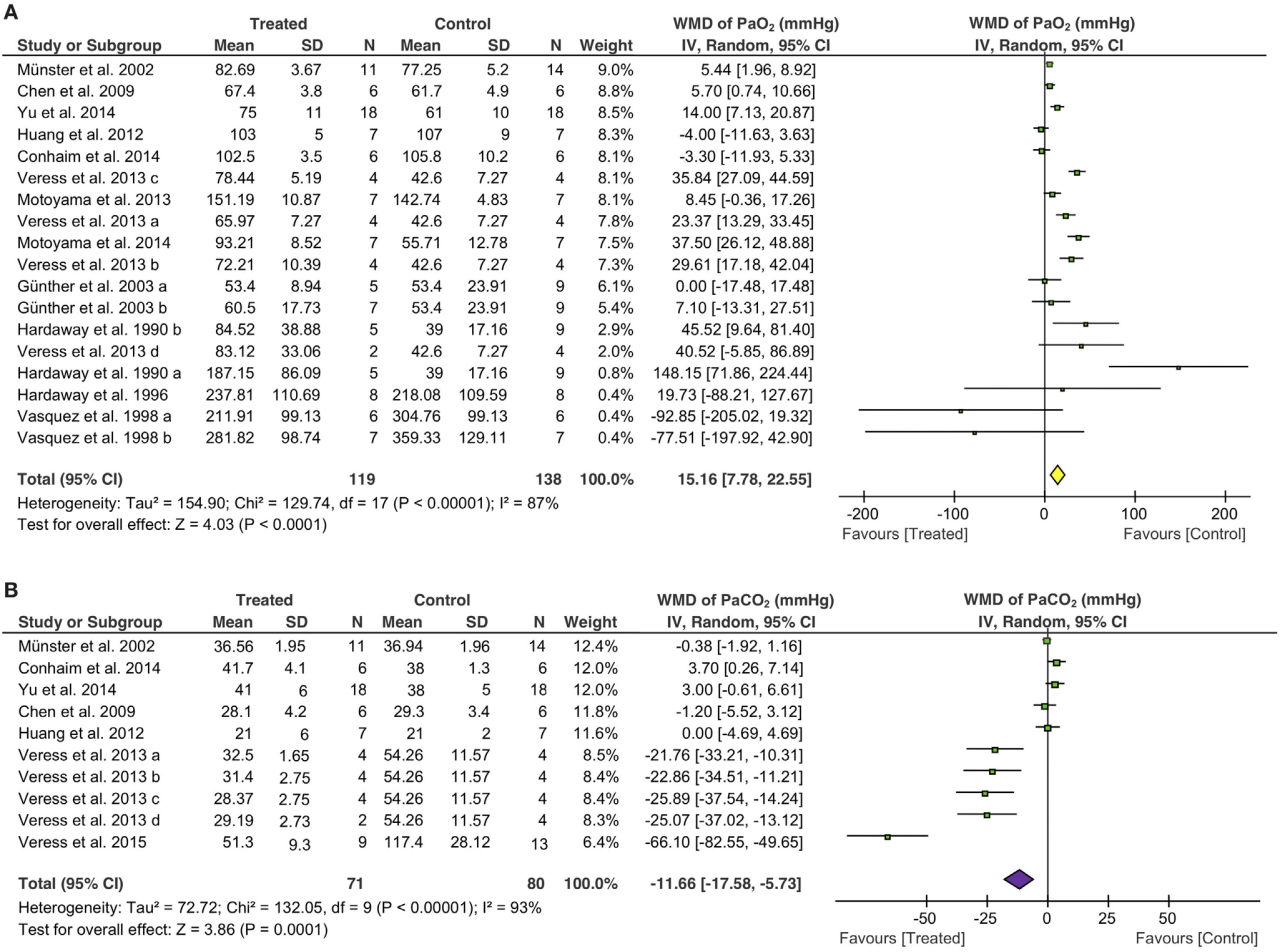
Effect of fibrinolytic therapy on PaO_2_ **(A)** and PaCO_2_ **(B)**. Weighted mean difference (WMD, square) and overall effect (diamond) are depicted.

Eighteen studies examined the effects of fibrinolytic therapy on blood oxygen concentration. Compared with controls, an increment of 15 mmHg (95% CI: 8 to 23 mmHg, *P* < 0.0001) in arterial oxygen tension (PaO_2_) was observed (Figure 3A). Because the heterogeneity between these studies was significant (*I*^2^ = 87%), we, therefore, analyzed the beneficial effects of each individual fibrinolysin. As a result, tPA, uPA, and plasmin elevated PaO_2_ with a net value of 23 mmHg (95% CI: 6 to 41 mmHg, *P* = 0.008), 8 mmHg (95% CI: 2 to 13 mmHg, *P* = 0.005), and 23 mmHg (95% CI: −6 to 51 mmHg, *P* = 0.12), respectively (Table 4). Next, the contribution of three routes to the efficacy of fibrinolytics on PaO_2_ was assessed. 5 of 10 studies delivered intravenously showed an increase of 15 mmHg in PaO_2_ (95% CI: 4 to 27 mmHg, *P* = 0.01) in treated animals. Intratracheal administration showed a beneficial effect (30 mmHg, 95% CI: 24 to 37 mmHg, *P* < 0.00001), whereas nebulization did not (3 mmHg, 95% CI: −2.0 to 8 mmHg, *P* = 0.18) (Table 4). Finally, we analyzed this benefit in small and large animals separately. Twelve small animal studies (85 animals for controls, 77 animals for treated group) were analyzed. Six studies favored fibrinolytic therapy, while another half did not. Ultimately, there was an improvement of 14 mmHg in PaO_2_ in small animals compared with controls (95% CI: 7 to 23 mmHg, *P* = 0.0007). In contrast, the improvement in large animals was statistically insignificant due to a large variance (21 mmHg, 95% CI: −26 to 68 mmHg, *P* = 0.38) (Table 4).

Overall arterial carbon dioxide pressure (PaCO_2_), another physiological parameter for gas exchange capacity of the lung, showed a significant reduction of 12 mmHg (95% CI: −18 to −5 mmHg, *P* = 0.0001) in treated animals (Figure 3B). Moreover, five of seven tPA studies (42 controls, 36 treated animals) favored fibrinolytic therapy with a decrease of 21 mmHg (95% CI: −34 to −8 mmHg, *P* = 0.002) in PaCO_2_, whereas the other two studies did not. In contrast, uPA did not significantly alter PaCO_2_ (0.3 mmHg, 95% CI: −2 to 2 mmHg, *P* = 0.79) in three studies (Table 4). Regarding routes of administration, all of the five studies investigating i.t. treatment (29 controls, 23 treated animals) demonstrated a favor, as there was a 31 mmHg reduction in PaCO_2_ (95% CI: −45 to −18 mmHg, *P* < 0.00001). In contrast, i.v. (3 studies) and nebulization (2 studies) did not exhibit significant effects on PaCO_2_ with a change of 1 mmHg (Table 4). Nine studies in small animals (66 controls, 60 treated animals) were evaluated for the efficacy of fibrinolytic therapy in the reduction of PaCO_2_. Five studies favored fibrinolytic therapy, and four studies did not. Taken together, the overall PaCO_2_ showed a reduction of 15 mmHg in small animals (95% CI: −23 to −7 mmHg, *P* = 0.0005). However, fibrinolytics did not reduce PaCO_2_ significantly in large animals (Table 4). In addition, the overall benefit of fibrinolytics on PO_2_ and PaCO_2_ levels at the same time point were confirmed by analyzing data at the endpoint of each study (Table 4, data with *).

### Fibrinolytic Activity

**Figure 4:**
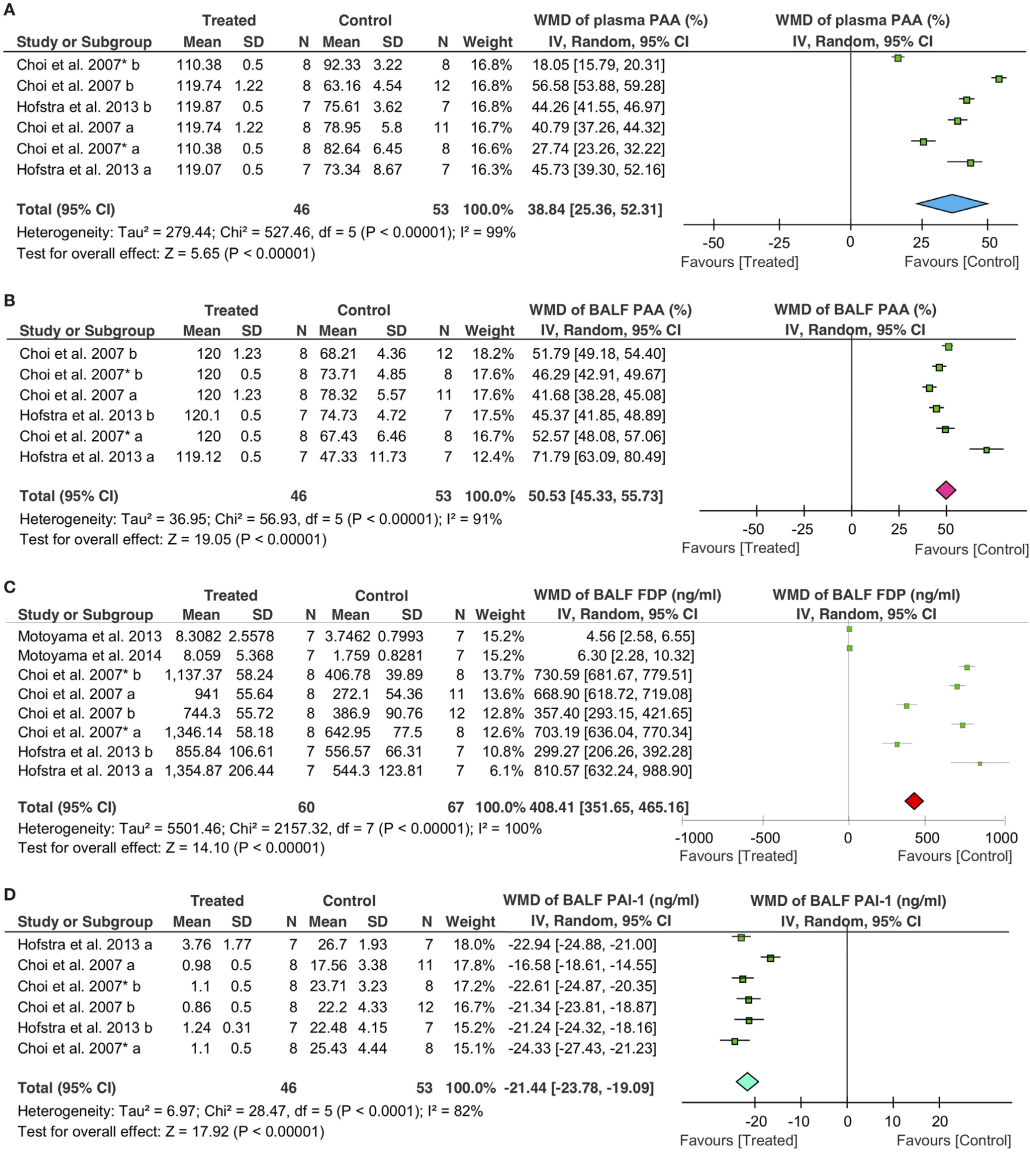
Effects of fibrinolytic treatment on the fibrinolysis in the plasma and bronchoalveolar lavage fluid (BALF). **(A)** Plasma plasminogen activator activity (PAA). **(B)** BALF PAA. **(C)** BALF fibrin degradation products (FDP). **(D)** BALF plasminogen activator inhibitor type 1 (PAI-1).

Fibrinolytic therapy significantly increased the fibrinolytic activity both in the plasma and BALF, as measured by the alterations in plasminogen activator activity (PAA), PAI-1, and FDP. Overall, fibrinolytics significantly increased 39% (95% CI: 25 to 52%, *P* < 0.00001) for PAA in the plasma, 51% (95% CI: 45 to 56%, *P* < 0.00001) for PAA in BALF, and 408 ng/ml (95% CI: 352 to 465 ng/ml, *P* < 0.00001) for FDP in BALF, respectively. In sharp contrast, PAI-1 in BALF was decreased significantly in treated animals (−21 ng/ml, 95% CI: −24 to −19 ng/ml, *P* < 0.00001) (Figure 4).

The effects of fibrinolytics on fibrinolytic activity were further analyzed with the data grouped by delivering routes. For PAA in the plasma, intravenous delivery was used in two studies (39 controls, 32 treated animals), and nebulization was applied in one publication (14 controls, 14 treated animals). Both systemic (i.v.) and local (nebulization) routes were effective in augmenting plasma PAA: an increase of 35% of basal level for i.v. (95% CI: 16 to 55%, *P* = 0.0003) and 44% for nebulization (95% CI: 42 to 47%, *P* < 0.00001), respectively, were observed (Table 4). Similarly, fibrinolytic activity in BALF was improved significantly. PAA in BALF was increased by 48% (95% CI: 43 to 53%, *P* < 0.00001) for i.v. and 58% (95% CI: 32 to 84%, *P* < 0.0001) for nebulization, respectively (Table 4); FDP in BALF were also elevated: 392 ng/ml for i.v. (95% CI: 331 to 453 ng/ml, *P* < 0.00001) and 549 ng/ml for nebulization (95% CI: 48 to 1,050 ng/ml, *P* = 0.03), respectively. With regards to individual fibrinolytic regimen, six tPA (53 controls, 46 treated animals) and two plasmin studies (14 controls, 14 treated animals) demonstrated an increment of 591 ng/ml (95% CI: 442 to 739 ng/ml, *P* < 0.00001) and 5 ng/ml (95% CI: 3 to 7 ng/ml, *P* < 0.00001) in FDP, respectively (Table 4). In contrast, PAI-1 in BALF was decreased significantly by fibrinolytics delivered *via* either i.v. (−21 ng/ml, 95% CI: −25 to −18 ng/ml, *P* < 0.00001) or nebulization (−22 ng/ml, 95% CI: −24 to −21 ng/ml, *P* < 0.00001) (Table 4).

### Coagulative Activity

**Figure 5:**
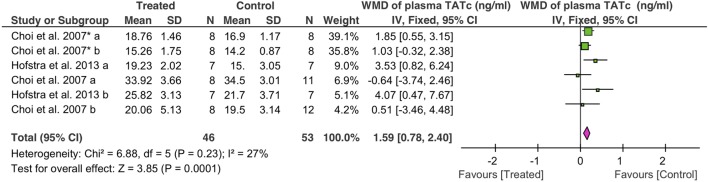
Effect of fibrinolytic therapy on plasma thrombin–antithrombin complexes.

Coagulation was analyzed as TATc. Overall, fibrinolytics significantly increased plasma TATc (1.6 ng/ml, 95% CI: 0.8 to 2.4 ng/ml, *P* = 0.0001) (Figure 5). Plasma TATc was measured in six studies: four studies *via* i.v. (39 controls, 32 treated animals) and two studies *via* nebulization (14 controls, 14 treated animals). Both nebulization (3.7 ng/ml, 95% CI: 1.6 to 5.9 ng/ml, *P* = 0.0007) and i.v. (1.2 ng/ml, 95% CI: 0.4 to 2.1 ng/ml, *P* = 0.005) increased plasma TATc (Table 4).

### Neutrophil Infiltration

**Figure 6:**
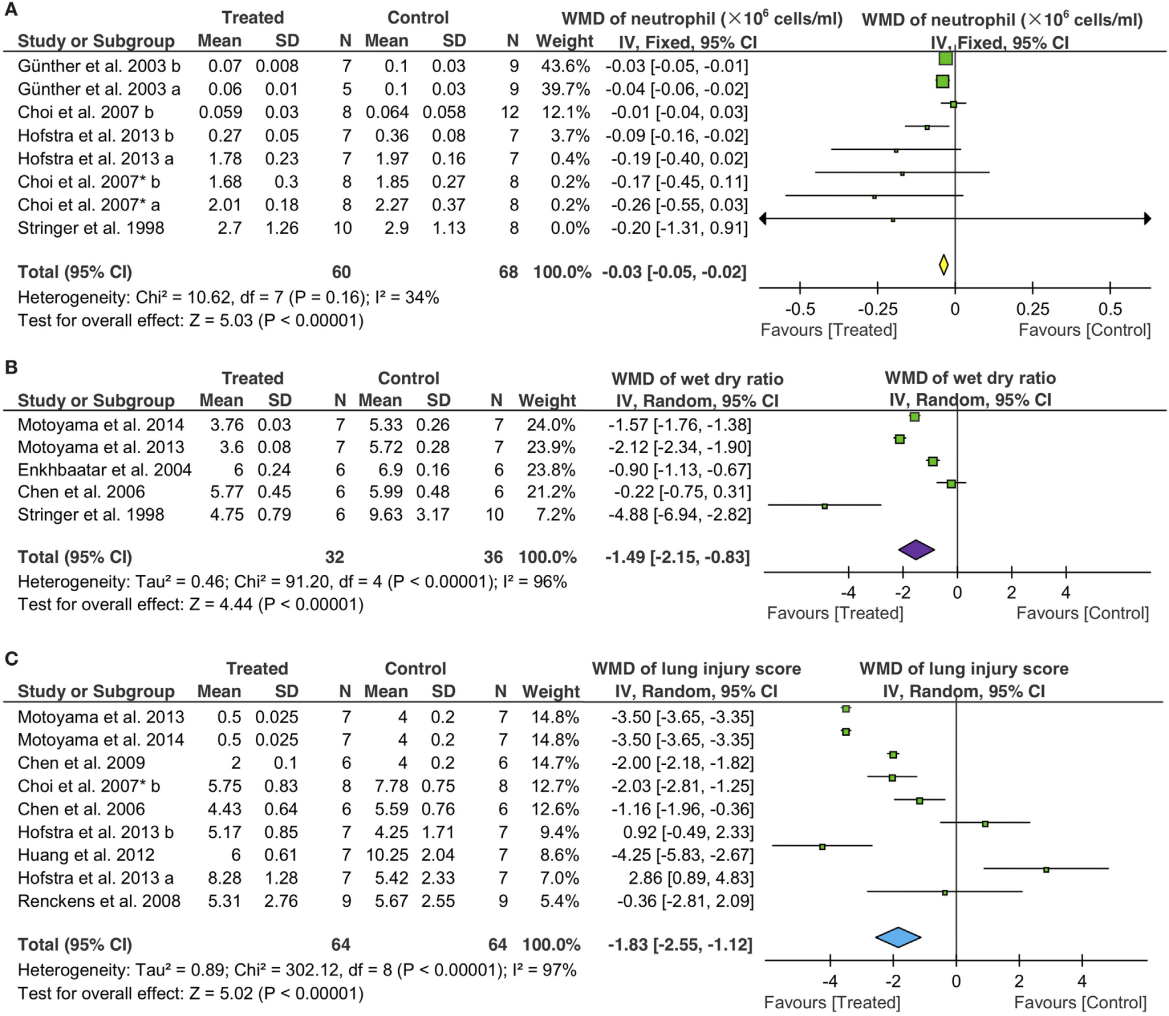
Effects of fibrinolytic therapy on lung neutrophils **(A)**, lung water content **(B)**, and lung injury score **(C)**.

We evaluated potential effects of fibrinolytic administration on neutrophils in BALF for lung inflammation. First, we adopted the fixed effect model (*I*^2^ = 34%) to analyze BALF neutrophils. An overall reduction of 3 × 10^4^ cells/ml in neutrophils was seen (95% CI: −5 to −2 × 10^4^ cells/ml, *P* < 0.00001) (Figure 6A). Six tPA (50 controls, 48 treated animals) and two uPA studies (18 controls, 12 treated animals) were analyzed separately. Only one tPA study showed lesser neutrophils (8 × 10^4^ cells/ml, 95% CI: −16 to −0 × 10^4^ cells/ml, *P* = 0.04) over that of controls. The other 5 tPA studies, however, did not exhibit significant changes in neutrophils compared with controls. UPA suppressed the infiltration of neutrophils into alveoli (−3 × 10^4^ cells/ml, 95% CI: −5 to −2 × 10^4^ cells/ml, *P* < 0.00001) in two studies. Nebulization of fibrinolytics was effective in reducing alveolar neutrophils (−4 × 10^4^ cells/ml, 95% CI: −6 to −2 × 10^4^ cells/ml, *P* = 0.0003). In contrast, neither intravenous nor intraperitoneal administration reduced alveolar neutrophils significantly: −1 × 10^5^ cells/ml for i.v. (95% CI: −26 to 7 × 10^4^ cells/ml, *P* = 0.26) and −2 × 10^5^ cells/ml for i. *P*. (95% CI: −1 to 1 × 10^6^ cells/ml, *P* = 0.71), respectively (Table 4). Apparently, fibrinolytic therapy decreased alveolar neutrophils in a route-dependent manner.

### Lung Edema

The random effect model was used to assess the efficacy of fibrinolytics on the resolution of edema fluid in injured lungs (Figure 6B). Total gravimetric lung wet/dry ratios were analyzed in two studies for plasmin (14 controls, 14 treated animals), two for tPA (16 controls, 12 treated animals), and one for uPA (6 controls, 6 treated animals). Our results showed that plasmin reduced lung water content (wet/dry ratio) significantly (−1.8, 95% CI: −2.4 to −1.3, *P* < 0.00001). However, the effects of tPA and uPA on edema resolution were insignificant statistically, albeit a greater extent than plasmin was reduced by tPA (−2.8, 95% CI: −6.6 to 1.1, *P* = 0.17) but not uPA (−0.2, 95% CI: −0.8 to 0.3, *P* = 0.41). Fibrinolytic effects on the resolution of edema fluid were examined by routes in five studies: three *via* i.v., one *via* nebulization, and one *via* i.p. A significant improvement in edema fluid clearance was seen when delivered *via* i.v. (−1.4, 95% CI: −2.1 to −0.6, *P* = 0.0004), nebulization (−0.9, 95% CI: −1.1 to −0.7, *P* < 0.00001), and i.p. (−5, 95% CI: −7 to −3, *P* < 0.00001). The effects of fibrinolytics on lung water content were analyzed by grouping data per animal sizes: three studies in small (20 controls, 24 treated animals) and two in large animals (12 controls, 12 treated animals). Lung water content was reduced to a greater extent by fibrinolytics in small animals (−2.1, 95% CI: −2.7 to −1.5, *P* < 0.00001) than that in large animals (−0.6, 95% CI: −1.3 to 0.1, *P* = 0.07) (Table 4).

### Histologic Lung Injury Score

The random effect model was utilized to examine the efficacy of fibrinolytics on histological lung injury score (Figure 6C) for the substantial heterogeneity existed among nine studies: five for tPA (38 controls, 38 treated animals), two for uPA (12 controls, 12 treated animals), and two for plasmin (14 controls, 14 treated animals). Both uPA and plasmin improved lung injury score, respectively, by a decrease of 1.7 for uPA (95% CI: −2.5 to −0.9, *P* < 0.0001) and 3.5 for plasmin (95% CI: −3.6 to −3.4, *P* < 0.00001). By comparison, tPA reduced lung injury score to a lesser extent (−0.6, 95% CI: −2.8 to 1.6, *P* = 0.55) insignificantly. Further, we analyzed potential differences between two routes, eight for i.v. (55 animals for both control and treated groups) and one for gene therapy (9 animals for both control and treated groups). Fibrinolytics delivered *via* i.v. reduced lung injury score significantly (−1.9 *via* i.v., 95% CI: −2.7 to −1.2, *P* < 0.00001), but not *via* gene therapy (−0.4, 95% CI: −2.8 to 2.1, *P* = 0.77). Finally, we assessed the data from nine studies grouped per animal sizes: eight in small (58 controls, 58 treated animals) and one in large animal (6 controls, 6 treated animals). A significant reduction in lung injury score was found in both small (−1.9, 95% CI: −2.7 to −1.2, *P* < 0.00001) and large animals (−1.2, 95% CI: −2.0 to −0.4, *P* = 0.004) treated with fibrinolytics (Table 4).

### Preventive and Therapeutic Intervention on Effects of Fibrinolysis

The mortality, PaO_2_, lung water content, and lung injury score of preventive and therapeutic intervention was analyzed. As to mortality, preventive strategy showed a beneficial effect (0.41, 95% CI: 0.24 to 0.68, *P* = 0.0007), whereas treatment strategy did not (0.11, 95% CI: 0.01 to 1.10, *P* = 0.06) (Table 5). Treatment strategy showed a beneficial effect (18.53 mmHg, 95% CI: 7.2 to 29.9 mmHg, *P* = 0.001) (Table 5) on PaO_2_, but not preventive strategy (8.09 mmHg, 95% CI: −11.5 to 27.7 mmHg, *P* = 0.42). With regards to lung water content, there was a significant decrease of 2.1 (95% CI: −2.7 to −1.5, *P* < 0.00001) compared with control groups in preventive maneuvers, but this decrease was not significant in therapeutic maneuver (−0.6, 95% CI: −1.3 to 0.06, *P* = 0.07). Compared with control group, the lung injury score was reduced by 2.1 for preventive maneuver (95% CI: −2.8 to −1.5), 1.7 for therapeutic maneuver (95% CI: −2.5 to −0.9) (Table 5).

**Table 5:** Effects of preventive strategies vs treatment strategies only.

Endpoints	Preventive strategies	Treatment strategies
Mortality	0.41 (0.24, 0.68) 0.0007	0.11 (0.01, 1.10) 0.06
PaO_2_	8.09 (−11.49, 27.67) 0.42	18.53 (7.15, 29.91) 0.001
Lung water content	−2.09 (−2.72, −1.45) <0.00001	−0.60 (−1.26, 0.06) 0.07
Lung injury score	−2.14 (−2.80, −1.48) <0.00001	−1.67 (−2.48, −0.87) <0.0001

### Assessment of Bias and Sensitivity Analysis

To determine if potential threats to internal validity influenced our findings, we evaluated the quality of included 22 studies in addition to the checklists of the ARRIVE guidelines (Table 2). Incomplete outcome data and selective outcome reporting in all studies were low risk. Randomization, blinding of personnel, allocation concealment, and blinding outcome assessment in 11 projects were low risk, whereas the risk in remaining projects was unclear (Table 6).

**Table 6:** Risk of bias assessments.

Study	Randomization	Blinding of personnel	Allocation concealment	Blinding of outcome assessment	Incomplete outcome data	Selective outcome reporting	Other bias
Hardaway et al., (37)	U	U	U	U	L	L	U
Hardaway et al., (15)	U	U	U	U	L	L	U
Hardaway et al., (25)	U	U	U	U	L	L	U
Stringer et al., (27)	U	U	U	U	L	L	U
Vasquez et al., (32)	U	U	U	U	L	L	U
Munster et al., (21)	L	L	L	L	L	L	U
Sisson et al., (36)	U	U	U	U	L	L	U
Gunther et al., (18)	U	U	U	U	L	L	U
Enkhbaatar et al., (16)	L	L	L	L	L	L	U
Chen et al., (33)	L	L	L	L	L	L	U
Choi et al., (20)	L	L	L	L	L	L	U
Choi et al., (19)*	L	L	L	L	L	L	U
Renckens et al., (31)	U	U	U	U	L	L	U
Chen et al., (35)	L	L	L	L	L	L	U
Huang et al., (22)	L	L	L	L	L	L	U
Hofstra et al., (24)	L	L	L	L	L	L	U
Motoyama et al., (38)	L	L	L	L	L	L	U
Veress et al., (29)	U	U	U	U	L	L	U
Conhaim et al., (28)	U	U	U	U	L	L	U
Motoyama et al., (39)	L	L	L	L	L	L	U
Yu et al., (34)	L	L	L	L	L	L	U
Veress et al., (30)	U	U	U	U	L	L	U

L, low risk of bias; U, unclear risk of bias.*Second publication from the same author.

**Figure 7:**
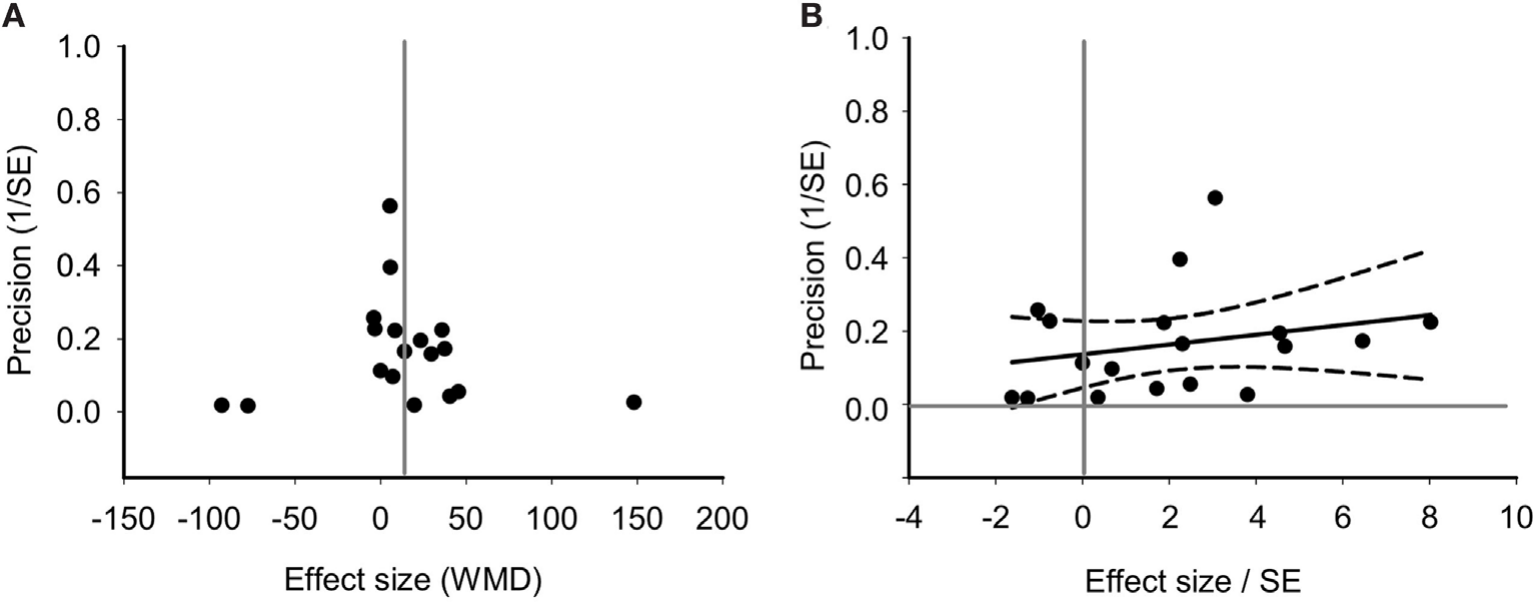
Bias assessment of PaO_2_ from 18 studies. **(A)** Funnel plots of precision (1/SE) as a function of weighted mean differences (WMD) showing the distribution of published study outcomes (filled circles). Vertical gray line is a global estimate of efficacy. **(B)** Egger regression of PaO_2_ precision (1/SE) against WMD/SE.

Funnel plot found no asymmetrical distribution for PaO_2_ (Figure 7A), which was confirmed by the Egger regression (Figure 7B; *P* = 0.199 for PaO_2_). Without imputing any missing studies for PaO_2_, the Trim and Fill analysis exhibited a symmetrical funnel plot too (data not shown).

**Figure 8:**
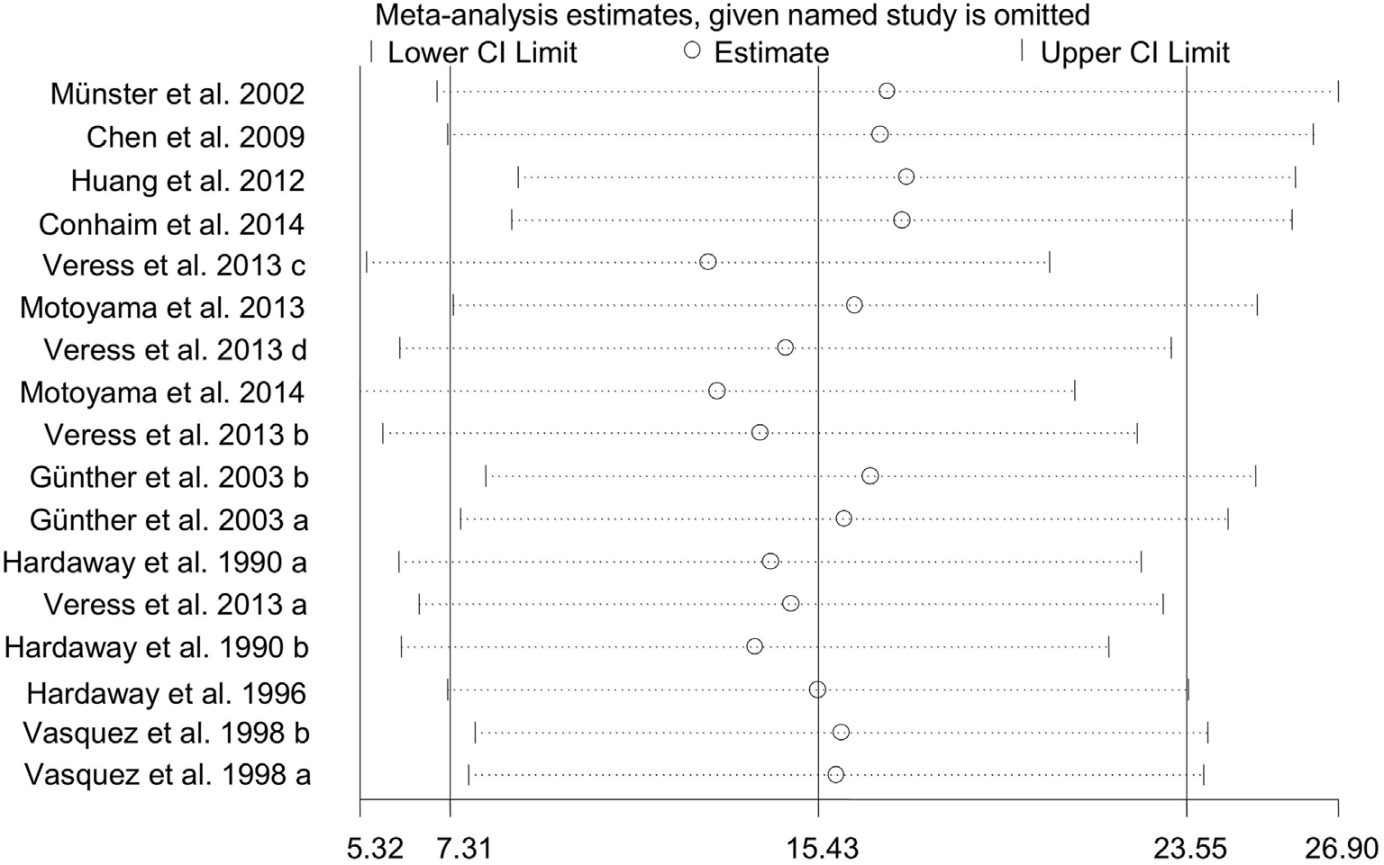
Sensitivity analysis of PaO_2_.

To test the stability and dependability of the results, we omitted one study (34), which had a relative large sample size. The combined WMD of PaO_2_ for remaining 17 studies was estimated by the sensitivity plot again, yielding a value of 15 (95% CI: 7 to 24) (Figure 8), which is same as the estimate of overall effect. These results indicated that the reliability of our meta-analysis was considerably strong.

## Discussion

Fibrinolytic therapy for ALI has been emerging during the last decade (41). Comparing with systematic evaluation (including systematic review and meta-analysis) of the efficacy of anticoagulants in clinical and preclinical studies of ALI (42), the potential benefit of fibrinolytics for ALI patients has not been tested by well-designed clinical trials yet. In this analysis, we have performed meta-analysis and systematic review, focusing on efficacy in 22 published preclinical studies of ALI. Our results suggest that fibrinolytic therapy significantly improves gas exchange, reduces alveolar neutrophils, increases fibrinolytic activity, reduces the quantity of pulmonary edema fluid, and suppresses the histologic severity of lung injury in a fibrinolysin-, route-, and species-dependent manners. The overall effects of fibrinolytics on death toll, oxygenation, fibrinolysis, and lung function were corroborated by analyzing subgrouped data and sensitivity. To our knowledge, this is the first meta-analysis to summarize previous preclinical studies aiming to provide evidence for further animal studies and clinical trials.

### Fibrinolytic Therapy for ALI Is Feasible and Tolerant

All of three fibrinolytic reagents, tPA, uPA, and plasmin were administered for 14 animal models of ALI in the included studies. The large range of applied doses demonstrates feasibility and tolerance. In addition, tolerability of tPA was confirmed in mice by a classic study (43). Airway bleeding was observed only where treatment was given locally with a large dose of tPA (≥1 mg/kg/d). Although the scarcity of gross hemorrhage and other severe adverse events applied *via* five routes were reported in 6 of 22 studies, considering the non-adherence to the ARRIVE guidelines (44) and informal systematic measurements of local and systemic hemorrhage quantitatively, the potential risk for bleeding associated with fibrinolytic therapy could not be ruled out.

### Mortality Improved by Fibrinolytic Therapy

Overall mortality was improved during the defined follow-up period from 1 h to 28 days. The most effective route to reduce mortality is intratracheal administration, and plasmin is the least effective lytic to improve survival rate. It is note worthy that only two of 10 studies followed mortality 48 h to 30 days. Other eight studies determined death toll within 2 days. Given the inconsistent follow-up time that differs from clinical studies, the overall improvement of mortality by fibrinolytic therapy shall be confirmed by further studies designed per the ARRIVE guidelines (44) and with extended follow-up periods identical to that for ALI/ARDS clinical studies.

### Fibrinolytic Therapy Improves Gas Exchange

Intriguingly, fibrinolytics increase gas exchange based on data at the same time-point, and to the most extent when delivered *via* intratracheal route. Fibrinolytics benefited gas exchange differently between small and large animals. It is probably due to divergent dose applied between species. Furthermore, the structure of the respiratory tract, lung function, inner surface of the lung for gas exchange, and oxygen consumption are body size dependent. Additionally, inconclusive results of oxygenation are seen for plasmin due to insufficient samples for meta-analysis. Hypoxia is correlated with the mortality of ALI (45). This notion is supported by the benefit of both gas exchange and survival rate by intratracheal delivery of fibrinolytic regimens.

### Fibrinolytic Therapy Alleviates Lung Injury

Our meta-analysis demonstrates that fibrinolytic therapy restores the dysfunctional fibrinolysis and coagulation in ALI. This benefit does not depend on routes and fibrinolytic regimens. Alveolar fibrin deposition attracted neutrophils and fibroblasts, and decreased lung edema fluid clearance (46). We identify that fibrinolysin tPA and i.v. route are the most potent for reducing neutrophil infiltration. We also find that fibrinolytics facilitate edema fluid resolution in small animals to a greater extent than in large animals. Our previous studies proved that uPA and plasmin but not tPA could activate apically located epithelial sodium channels, a predominate pathway to remove edema fluid (47, 48). Considering that tPA competitively binds to PAI-1 with a greater affinity than uPA, delivered tPA may form complexes with elevated PAI-1 and serve as a “cage” to separate endogenous uPA from PAI-1 (49). Under this situation, freed uPA is able to activate epithelial sodium channels and resolve edema fluid. It is unclear why intraperitoneal administration eliminates lung edema to the most extent. At least, this is the only way to get rid of direct addition of extra fluid to the flooded air spaces intratracheally or indirectly leaking to the alveoli through the impaired blood–gas barrier post intravenously fluid infusion. In addition, we find that plasmin may be the best fibrinolysin to improve lung injury score. Plasmin may defragment deposited fibrin. However, this is not supported by FDP content in BALF. It is probably due to the cleavage of epithelial sodium channels by plasmin proteolytically to expedite transalveolar fluid re-absorption (unpublished data). Exogenously applied tPA displayed anti-inflammatory effects (50). Therefore, anti-inflammatory properties of three fibrinolytic agents could be a mechanism for alleviating ALI. Taken together, fibrinolytic regimens may suppress the mortality in preclinical models of ALI through multifaceted mechanisms in a route- and model-dependent manner. ALI animal models may not be representative of human ALI, because of the timing and severity of ALI induction, the dose and timing of the treatment in relation to ALI induction, the use of small/young animals without comorbid illnesses, and lack of administration of standard of care co-interventions, such as fluids and antibiotics during the study period. How well animal models of ALI mimic the pathophysiology of human ALI has also been a contentious issue. Thus, the effect of construct validity on fibrinolytic therapy of ALI remains to be determined.

### Limitations of Our Analysis

Our review has some limitations. First, the duration of delivery of fibrinolytic agents and the period of observation were not consistent. More than half of the included studies do not adhere to the ARRIVE guidelines. Second, although subgroups and sensitivity analyses were conducted, we could not completely explain the substantial heterogeneity and some diverse effects in experimental ALI. Third, uncertainty of the potential benefits of fibrinolytics in ALI may be improved by future preclinical studies with larger numbers of animal studies. For example, we could not perform dose-effect analysis because of a paucity of sufficient data. Finally, the effects of fibrinolytics on ALI animal models provide basic information for further preclinical studies. Considering the difference between relative homogeneous animals (e.g., healthy young or adult animals without comorbidity and extremely heterogeneous patients), it shall be cautious to link the findings in preclinical studies to ALI patients.

## Conclusion

Our results identify that the best route is intratracheal delivery, and the most efficacious regimen is tPA. FDA approved fibrinolytic therapies for cardiovascular and pleural diseases, including hypertensive intraventricular hemorrhage (51), loculated pleural effusions, parapneumonic effusions, pleural empyema, malignant effusions, hemothorax, and myocardial infarction (52–55), demonstrate the tolerance and feasibility of this treatment. This meta-analysis, however, did not provide data on the optimized dose of each therapy, the optimal time to initiate therapy, and the model-specific strategy. These unsolved questions await further well-designed preclinical studies following the ARRIVE guidelines. On balance, this review provides preclinical evidence for designing clinical trials to test the benefit of the fibrinolytic therapies for ALI.

## Author Contributions

Project design: H-LJ, RZ, MM, and XLZ. Data collection: CL, ZS, MZ, YM, and H-LJ. Data analysis: CL, ZS, YM, and LZ. Manuscript preparation: CL, YM, RZ, H-LJ, H-GN, MM, XL, and PX. Revision: H-LJ, XJZ, and MM. Approval and submission: H-LJ and MM.

## Conflict of Interest Statement

The authors declare that the research was conducted in the absence of any commercial or financial relationships that could be construed as a potential conflict of interest.
