# Supplementary material for: Meta-Analysis of Preclinical Studies of Fibrinolytic Therapy for Acute Lung Injury
Source: Front Immunol. 2018 Aug 20;9:1898. doi: 10.3389/fimmu.2018.01898 (PMC6110197; doi:10.3389/fimmu.2018.01898)
Supplement: Supplementary file 1 [file Data_Sheet_1.docx]

**S1. Systematic search strategies**

**Web of Science**

#1 Topic = (tPA) OR Topic = (tissue-type plasminogen activator)

#2 Topic= (uPA) OR Topic = (urokinase plasminogen activator) OR Topic = (urokinase) OR Topic= (Abbokinase) OR Topic= (breokinase)

#3 Topic= (fibrinolytics)

#4 Topic= (plasmin)

#5 Topic= (thrombolysin)

#6 Topic= (Eminase) OR

#7 Topic= (desmoteplase) OR

#8 Topic= (pro-urokinase) OR

#9 Topic= (staphylokinase) OR

#10 Topic= (fibrinase) OR

#11 Topic= (fibrinolysin) OR

#12 Topic= (Alfimeprase) OR

#13 Topic= (SK) OR (streptokinase) OR (streptase) OR (kabikinase)

#14 Topic= (win-kase)

#15 Topic= (kinlytic)

#16 Topic= (reteplase) OR (rctPA) OR (retavase) OR (actilyse)

#17 Topic= (Activase) OR (alteplase)

#18 Topic= (Tenecteplase) OR (TNkase) OR (TNktpA) OR (metalyse)

#19 Topic=(plasminogen activator)

#20 #1 OR #2 OR #3 OR #4 OR #5 OR #6 OR #7 OR #8 OR #9 OR #10 OR #11 OR #12 OR #13 OR #14 OR #15 OR #16 OR #17 OR #18 OR #19

#21 Topic= (ARDS) OR Topic = (Acute respiratory distress syndrome) OR Topic = (respiratory distress syndrome) OR Topic = (ALI) OR Topic= (acute lung injury) respiratory distress syndrome

#22 Topic= (lung injury)

#23 Topic= (septic) OR Topic = (sepsis) OR Topic = (bacteremia) OR Topic = (endotoximia)

#24 Topic= (pneumonia)

#25 Topic= (shock)

#26 Topic= (multi-organ failure) OR Topic = (respiratory failure)

#27 Topic= (pulmonary edema) OR Topic = (lung edema) OR Topic = (edematous) OR Topic = (pulmonary oedem)

#28 #21 OR #22 OR #23 OR #24 OR #25 OR #26 OR #27

#29 #20 AND #28

**PubMed**

("fibrinolytics" OR "plasmin" OR "urokinase" OR "uPA" OR "tPA" OR "actase" OR "thrombolysin" OR "Eminase" OR "desmoteplase" OR "pro-urokinase" OR "staphylokinase" OR "fibrinase" OR "fibrinolysin" OR "Alfimeprase" OR "SK" OR "streptokinase" OR "streptase" OR "kabikinase" OR "Abbokinase" OR "breokinase" OR "win-kase" OR "kinlytic" OR "Activase" OR "reteplase" OR "rctPA" OR "retavase" OR "actilyse" OR "repilysin" OR "alteplase" OR "Tenecteplase" OR "TNkase" OR "TNktpA" OR "metalyse" OR "plasminogen activator") AND ("lung injury" OR "ARDS" OR "ALI" OR " respiratory distress syndrome" OR "septic" OR "sepsis" OR "bacteremia" OR "endotoximia" OR "multi-organ failure" OR "respiratory failure" OR "pneumonia" OR "shock" OR "pulmonary edema" OR "lung edema" OR "edematous" OR "pulmonary oedem")

**EMbase**

#1 fibrinolytics

#2 plasmin

#3 urokinase

#4 uPA

#5 tPA

#6 actase

#7 thrombolysin

#8 Eminase

#9 desmoteplase

#10 pro-urokinase

#11 staphylokinase

#12 fibrinase

#13 fibrinolysin

#14 Alfimeprase

#15 SK

#16 streptokinase

#17 streptase

#18 kabikinase

#19 Abbokinase

#20 breokinase

#21 win-kase

#22 kinlytic

#23 Activase

#24 reteplase

#25 rctPA

#26 retavase

#27 actilyse

#28 repilysin

#29 alteplase

#30 Tenecteplase

#31 TNkase

#32 TNktpA

#33 metalyse

#34 plasminogen activator

#35 #1 OR #2 OR #3 OR #4 OR #5 OR #6 OR #7 OR #8 OR #9 OR #10 OR #11 OR #12 OR #13 OR #14 OR #15 OR #16 OR #17 OR #18 OR #19 OR #20 OR #21 OR #22 OR #23 OR #24 OR #25 OR #26 OR #27 OR #28 OR #29 OR #30 OR #31 OR #32 OR #33 OR #34

#36 lung injury

#37 ARDS

#38 ALI

#39 respiratory distress syndrome

#40 septic

#41 sepsis

#42 bacteremia

#43 endotoximia

#44 multi-organ failure

#45 respiratory failure

#46 pneumonia

#47 shock

#48 pulmonary edema

#49 lung edema

#50 edematous

#51 pulmonary oedem

#52 #36 OR #37 OR #38 OR #39 OR #40 OR #41 OR #42 OR #43 OR #44 OR #45 OR #46 OR #47 OR #48 OR #49 OR #50 OR #51

#53 #35 AND #52

#54 #35 AND #52 AND ([animal experiment]/lim OR [animal model]/lim)

**CNKI**

检索式：（纤溶酶 OR尿激酶 OR 组织型纤溶酶原激活物）and （肺损伤 OR 急性肺损伤OR 急性呼吸窘迫综合征 OR 败血症 OR 脓血症）

检索字段：主题

**Retrieved Results:**

**Web of Science: 3248**

**PubMed: 3317**

**EMbase: 992**

**CNKI: 67**

**Total: 7624**

**Duplication: 2562**

**Eligibility studies: 73**

**Included studies: 22**

**Detailed Results**

**Web of Science- Result: 3248**

[1] Acun T, Doberstein N, Habermann JK, et al. HLJ1 (DNAJB4) Gene Is a Novel Biomarker Candidate in Breast Cancer [J]. Omics-a Journal of Integrative Biology, 2017,21(5): 257-265.

[2] Al-Ashry H, Abuzaid A, Asim M, et al. Microcirculation Alteration and Biomarker Dilemma in Early Septic Shock Diagnosis and Treatment [J]. Current Vascular Pharmacology, 2016,14(4): 330-344.

[3] Aldabbous L, Abdul-Salam V, McKinnon T, et al. Neutrophil Extracellular Traps Promote Angiogenesis: Evidence From Vascular Pathology in Pulmonary Hypertension [J]. Arteriosclerosis Thrombosis and Vascular Biology, 2016,36(10): 2078-2087.

[4] Alharbi NK, Padron-Regalado E, Thompson CP, et al. ChAdOx1 and MVA based vaccine candidates against MERS-CoV elicit neutralising antibodies and cellular immune responses in mice [J]. Vaccine, 2017,35(30): 3780-3788.

[5] Amin Z, Rahmawati FN. Recent insight into potential acute respiratory distress syndrome [J]. Saudi Medical Journal, 2017,38(4): 344-349.

[6] Anan C, Oomura M, Saeki T, et al. Fatal Intraperitoneal Bleeding after Intravenous Administration of Tissue Plasminogen Activator [J]. Journal of Stroke & Cerebrovascular Diseases, 2015,24(7): E177-E178.

[7] Armstrong PW, Zheng YG, Westerhout CM, et al. Reduced dose tenecteplase and outcomes in elderly ST-segment elevation myocardial infarction patients: Insights from the STrategic Reperfusion Early After Myocardial infarction trial [J]. American Heart Journal, 2015,169(6): 890-+.

[8] Arnold SM, Dinkins M, Mooney LH, et al. Very Early Mobilization in Stroke Patients Treated with Intravenous Recombinant Tissue Plasminogen Activator [J]. Journal of Stroke & Cerebrovascular Diseases, 2015,24(6): 1168-1173.

[9] Avgerinos ED, Abou Ali AN, Liang NL, et al. Predictors of failure and complications of catheter-directed interventions for pulmonary embolism [J]. Journal of Vascular Surgery-Venous and Lymphatic Disorders, 2017,5(3): 303-310.

[10] Babayigit A, Cebeci B, Buyukkale G, et al. Treatment of neonatal fungal infective endocarditis with recombinant tissue plasminogen: activator in a low birth weight infant case report and review of the literature [J]. Mycoses, 2015,58(10): 578-581.

[11] Ballard DW, Kim AS, Huang J, et al. Implementation of Computerized Physician Order Entry Is Associated With Increased Thrombolytic Administration for Emergency Department Patients With Acute Ischemic Stroke [J]. Annals of Emergency Medicine, 2015,66(6): 601-610.

[12] Ban CJ, Wang TD, Zhang S, et al. Fibrinolytic system related to pulmonary arterial pressure and lung function of patients with idiopathic pulmonary fibrosis [J]. Clinical Respiratory Journal, 2017,11(5): 640-647.

[13] Barton AK, Wirth C, Bondzio A, et al. Are pulmonary hemostasis and fibrinolysis out of balance in equine chronic pneumopathies? [J]. Journal of Veterinary Science, 2017,18(3): 349-357.

[14] Bataillard A, Hebrard A, Gaide-Chevronnay L, et al. Extracorporeal life support for massive pulmonary embolism during pregnancy [J]. Perfusion-Uk, 2016,31(2): 169-171.

[15] Beitl E, Banasova A, Vlcek M, et al. Nitric oxide as an indicator for severity of injury in polytrauma [J]. Bratislava Medical Journal-Bratislavske Lekarske Listy, 2016,117(4): 217-220.

[16] Bhandary YP. p53-Fibrinolytic system and acute lung injury [J]. Biologia, 2016,71(10): 1098-1102.

[17] Bhandary YP, Shetty SK, Marudamuthu AS, et al. Plasminogen Activator Inhibitor-1 in Cigarette Smoke Exposure and Influenza A Virus Infection-Induced Lung Injury [J]. Plos One, 2015,10(5).

[18] Biswas A, Jantz MA, Mehta HJ. "Less Is More" Approach for Management of Intrapleural Sepsis [J]. Annals of the American Thoracic Society, 2017,14(8): 1355-1356.

[19] Bleiziffer I, Eikmeier J, Pohlentz G, et al. The Plasmin-Sensitive Protein Pls in Methicillin-Resistant Staphylococcus aureus (MRSA) Is a Glycoprotein [J]. Plos Pathogens, 2017,13(1).

[20] Bock A, Tucker N, Kelher MR, et al. alpha-ENOLASE CAUSES PROINFLAMMATORY ACTIVATION OF PULMONARY MICROVASCULAR ENDOTHELIAL CELLS AND PRIMES NEUTROPHILS THROUGH PLASMIN ACTIVATION OF PROTEASE-ACTIVATED RECEPTOR 2 [J]. Shock, 2015,44(2): 137-142.

[21] Boeddha NP, Driessen GJ, Cnossen MH, et al. Circadian Variation of Plasminogen-Activator Inhibitor-1 Levels in Children with Meningococcal Sepsis [J]. Plos One, 2016,11(11).

[22] Boeddha NP, Emonts M, Cnossen MH, et al. Gene Variations in the Protein C and Fibrinolytic Pathway: Relevance for Severity and Outcome in Pediatric Sepsis [J]. Seminars in Thrombosis and Hemostasis, 2017,43(1): 36-47.

[23] Bolis RM, Morard G, Vinci T, et al. Decaying shock studies of phase transitions in MgO-SiO2 systems: Implications for the super-Earths' interiors [J]. Geophysical Research Letters, 2016,43(18): 9475-9483.

[24] Bonkain F, Van Hulle F, Janssens P, et al. Urokinase-containing locking solution in the prevention of dialysis catheter dysfunction: a double blind randomized controlled trial [J]. Journal of Vascular Access, 2017,18(5): 436-442.

[25] Boren J, Shryock G, Fergis A, et al. Inhibition of Glycogen Synthase Kinase 3 beta Blocks Mesomesenchymal Transition and Attenuates Streptococcus pneumonia-Mediated Pleural Injury in Mice [J]. American Journal of Pathology, 2017,187(11): 2461-2472.

[26] Bos LD, Schouten LR, van Vught LA, et al. Identification and validation of distinct biological phenotypes in patients with acute respiratory distress syndrome by cluster analysis [J]. Thorax, 2017,72(10): 876-883.

[27] Branco BC, Montero-Baker MF, Mills JL. The pros and cons of endovascular and open surgical treatments for patients with acute limb ischemia [J]. Journal of Cardiovascular Surgery, 2015,56(3): 401-407.

[28] Brifault C, Gilder AS, Laudati E, et al. Shedding of membrane-associated LDL receptor-related protein-1 from microglia amplifies and sustains neuroinflammation [J]. Journal of Biological Chemistry, 2017,292(45): 18699-18712.

[29] Brooks MB, Turk JR, Guerrero A, et al. Non-Lethal Endotoxin Injection: A Rat Model of Hypercoagulability [J]. Plos One, 2017,12(1).

[30] Brown RV, Wang T, Chappeta VR, et al. The Consequences of Overlapping G-Quadruplexes and i-Motifs in the Platelet-Derived Growth Factor Receptor beta Core Promoter Nuclease Hypersensitive Element Can Explain the Unexpected Effects of Mutations and Provide Opportunities for Selective Targeting of Both Structures by Small Molecules To Downregulate Gene Expression [J]. Journal of the American Chemical Society, 2017,139(22): 7456-7475.

[31] Bruening T, Al-Khaled M. Stroke-Associated Pneumonia in Thrombolyzed Patients: Incidence and Outcome [J]. Journal of Stroke & Cerebrovascular Diseases, 2015,24(8): 1724-1729.

[32] Bundhun PK, Janoo G, Chen MH. Bleeding events associated with fibrinolytic therapy and primary percutaneous coronary intervention in patients with STEMI A systematic review and meta-analysis of randomized controlled trials [J]. Medicine, 2016,95(23).

[33] Burns TA, Watts MR, Weber PS, et al. Laminar inflammatory events in lean and obese ponies subjected to high carbohydrate feeding: Implications for pasture-associated laminitis [J]. Equine Veterinary Journal, 2015,47(4): 489-493.

[34] Bustamante A, Garcia-Berrocoso T, Penalba A, et al. Sepsis.biomarkers reprofiling to predict stroke-associated infections [J]. Journal of Neuroimmunology, 2017,312: 19-23.

[35] Caffarelli C, Santamaria F, Cesari S, et al. Advances in pediatrics in 2014: current practices and challenges in allergy, gastroenterology, infectious diseases, neonatology, nutrition, oncology and respiratory tract illnesses [J]. Italian Journal of Pediatrics, 2015,41.

[36] Cai Q, Zhang HP, Zhao D, et al. Analysis of three surgical treatments for spontaneous supratentorial intracerebral hemorrhage [J]. Medicine, 2017,96(43).

[37] Cantu E, Suzuki Y, Diamond JM, et al. Protein Quantitative Trait Loci Analysis Identifies Genetic Variation in the Innate Immune Regulator TOLLIP in Post-Lung Transplant Primary Graft Dysfunction Risk [J]. American Journal of Transplantation, 2016,16(3): 833-840.

[38] Casagranda I, Vendramin C, Callegari T, et al. Usefulness of suPAR in the risk stratification of patients with sepsis admitted to the emergency department [J]. Internal and Emergency Medicine, 2015,10(6): 725-730.

[39] Cascorbi I, Lampl L, Adams HA, et al. Haemostasis in shock Part 5: Haemostatically active drugs [J]. Anasthesiologie & Intensivmedizin, 2016,57: 120-+.

[40] Cekmez F, Fidanci MK, Ayar G, et al. Diagnostic Value of Upar, IL-33, and ST2 Levels in Childhood Sepsis [J]. Clinical Laboratory, 2016,62(5): 751-755.

[41] Cha LMJ, Choi S, Kim T, et al. Intrapleural urokinase therapy in a neonate with pleural empyema [J]. Pediatrics International, 2016,58(7): 616-619.

[42] Chang KC. Cilostazol inhibits HMGB1 release in LPS-activated RAW 264.7 cells and increases the survival of septic mice [J]. Thrombosis Research, 2015,136(2): 456-464.

[43] Chapman MP, Moore EE, Moore HB, et al. Overwhelming tPA release, not PAI-1 degradation, is responsible for hyperfibrinolysis in severely injured trauma patients [J]. Journal of Trauma and Acute Care Surgery, 2016,80(1): 16-25.

[44] Chaudhry SA, Afzal MR, Chaudhry BZ, et al. Rates of Adverse Events and Outcomes among Stroke Patients Admitted to Primary Stroke Centers [J]. Journal of Stroke & Cerebrovascular Diseases, 2016,25(8): 1960-1965.

[45] Chen S, He Y, Hu ZW, et al. Heparanase Mediates Intestinal Inflammation and Injury in a Mouse Model of Sepsis [J]. Journal of Histochemistry & Cytochemistry, 2017,65(4): 241-249.

[46] Chen XY, Walther FJ, Laghmani E, et al. Adult Lysophosphatidic Acid Receptor 1-Deficient Rats with Hyperoxia-Induced Neonatal Chronic Lung Disease Are Protected against Lipopolysaccharide-Induced Acute Lung Injury [J]. Frontiers in Physiology, 2017,8.

[47] Chen Y, Lu ZJ, Yang Y, et al. Suppression of plasminogen activator inhibitor-1 by inhaled nitric oxide attenuates the adverse effects of hyperoxia in a rat model of acute lung injury [J]. Thrombosis Research, 2015,136(1): 131-138.

[48] Chen YH, Liu W, Wang YW, et al. Casein Kinase 2 Interacting Protein-1 regulates M1 and M2 inflammatory macrophage polarization [J]. Cellular Signalling, 2017,33: 107-121.

[49] Chestovich PJ, Browder TD, Morrissey SL, et al. The clinical significance of circulating soluble RAGE in patients with severe sepsis [J]. Journal of Trauma and Acute Care Surgery, 2015,78(6): 1076-1083.

[50] Chin-Chan M, Navarro-Yepes J, Quintanilla-Vega B. Environmental pollutants as risk factors for neurodegenerative disorders: Alzheimer and Parkinson diseases [J]. Frontiers in Cellular Neuroscience, 2015,9.

[51] Choi H, Ann H, Shin M. Heat shock protein 90 inhibitor (17-Dimethylaminoethylamino-17-demethoxygeldanamycin) enhances apoptosis by inhibiting the AKT and p38 MAPK in thermal stimulated SK-MEL-2 human melanoma cell line [J]. Journal of Investigative Dermatology, 2017,137(10): S294-S294.

[52] Choi KS, Kim JD, Kim HC, et al. Percutaneous Aspiration Embolectomy Using Guiding Catheter for the Superior Mesenteric Artery Embolism [J]. Korean Journal of Radiology, 2015,16(4): 736-743.

[53] Christiansen ME, Kumar G, Mahabir RC, et al. Intravenous Alteplase for Acute Stroke and Pulmonary Embolism in a Patient With Recent Abdominoplasty [J]. Neurologist, 2017,22(4): 150-152.

[54] Chu SG, El-Chemaly S, Rosas IO. Genetics and Idiopathic Interstitial Pneumonias [J]. Seminars in Respiratory and Critical Care Medicine, 2016,37(3): 321-330.

[55] Chung EJ, McKay-Corkum G, Chung S, et al. Truncated Plasminogen Activator Inhibitor-1 Protein Protects From Pulmonary Fibrosis Mediated by Irradiation in a Murine Model [J]. International Journal of Radiation Oncology Biology Physics, 2016,94(5): 1163-1172.

[56] Cinar RK, Sonmez MB, Gorgulu Y. Peripheral blood mRNA expressions of stress biomarkers in manic episode and subsequent remission [J]. Psychoneuroendocrinology, 2016,70: 10-16.

[57] Colombo EA, Spaccini L, Volpi L, et al. Viable phenotype of ILNEB syndrome without nephrotic impairment in siblings heterozygous for unreported integrin alpha3 mutations [J]. Orphanet Journal of Rare Diseases, 2016,11.

[58] Corcoran JP, Wrightson JM, Belcher E, et al. Pleural infection: past, present, and future directions [J]. Lancet Respiratory Medicine, 2015,3(7): 563-577.

[59] Coy P, Yanagimachi R. The Common and Species-Specific Roles of Oviductal Proteins in Mammalian Fertilization and Embryo Development [J]. Bioscience, 2015,65(10): 973-984.

[60] Csuka D, Veszeli N, Imreh E, et al. Comprehensive study into the activation of the plasma enzyme systems during attacks of hereditary angioedema due to C1-inhibitor deficiency [J]. Orphanet Journal of Rare Diseases, 2015,10.

[61] Cunningham FG, Nelson DB. Disseminated Intravascular Coagulation Syndromes in Obstetrics [J]. Obstetrics and Gynecology, 2015,126(5): 999-1011.

[62] Danger JL, Cao TN, Cao TH, et al. The small regulatory RNA FasX enhances group A Streptococcus virulence and inhibits pilus expression via serotype-specific targets [J]. Molecular Microbiology, 2015,96(2): 249-262.

[63] Davenport RA, Brohi K. Cause of trauma-induced coagulopathy [J]. Current Opinion in Anesthesiology, 2016,29(2): 212-219.

[64] Davis RW, Eggleston H, Johnson F, et al. In Vivo Tracking of Streptococcal Infections of Subcutaneous Origin in a Murine Model [J]. Molecular Imaging and Biology, 2015,17(6): 793-801.

[65] Day DE, Oedorf K, Kogan S, et al. The Utility of Inflammatory and Endothelial Markers to Identify Infection in Emergency Department Patients [J]. Shock, 2015,44(3): 215-220.

[66] de Guadiana-Romualdo LG, Berger M, Jimenez-Santos E, et al. Pancreatic stone protein and soluble CD25 for infection and sepsis in an emergency department [J]. European Journal of Clinical Investigation, 2017,47(4): 297-304.

[67] Deganello A, Rafailidis V, Sellars ME, et al. Intravenous and Intracavitary Use of Contrast-Enhanced Ultrasound in the Evaluation and Management of Complicated Pediatric Pneumonia [J]. Journal of Ultrasound in Medicine, 2017,36(9): 1943-1954.

[68] Delano MJ, Rizoli SB, Rhind SG, et al. Prehospital Resuscitation of Traumatic Hemorrhagic Shock with Hypertonic Solutions Worsens Hypocoagulation and Hyperfibrinolysis [J]. Shock, 2015,44(1): 25-31.

[69] Deng Y, Verron E, Rohanizadeh R. Molecular Mechanisms of Anti-metastatic Activity of Curcumin [J]. Anticancer Research, 2016,36(11): 5639-5647.

[70] Desjarlais MP, Knudson MD, Cochrane KR. Extension of the Hugoniot and analytical release model of alpha-quartz to 0.2-3 TPa [J]. Journal of Applied Physics, 2017,122(3).

[71] Di Battista AP, Rizoli SB, Lejnieks B, et al. SYMPATHOADRENAL ACTIVATION IS ASSOCIATED WITH ACUTE TRAUMATIC COAGULOPATHY AND ENDOTHELIOPATHY IN ISOLATED BRAIN INJURY [J]. Shock, 2016,46(3): 96-103.

[72] Dibb MJ, Abraham A, Chadwick PR, et al. Central Venous Catheter Salvage in Home Parenteral Nutrition Catheter-Related Bloodstream Infections: Long-Term Safety and Efficacy Data [J]. Journal of Parenteral and Enteral Nutrition, 2016,40(5): 699-704.

[73] Diebel LN, Martin JV, Liberati DM. Early tranexamic acid administration ameliorates the endotheliopathy of trauma and shock in an in vitro model [J]. Journal of Trauma and Acute Care Surgery, 2017,82(6): 1080-1086.

[74] Ding RY, Zhao DM, Li XX, et al. Rho-kinase inhibitor treatment prevents pulmonary inflammation and coagulation in lipopolysaccharide-induced lung injury [J]. Thrombosis Research, 2017,150: 59-64.

[75] Dorman RM, Vali K, Rothstein DH. Trends in treatment of infectious parapneumonic effusions in US children's hospitals, 2004-2014 [J]. Journal of Pediatric Surgery, 2016,51(6): 885-890.

[76] Doty KA, Wilburn DB, Bowen KE, et al. Co-option and evolution of non-olfactory proteinaceous pheromones in a terrestrial lungless salamander [J]. Journal of Proteomics, 2016,135: 101-111.

[77] Duburcq T, Tournoys A, Gnemmi V, et al. IMPACT OF OBESITY ON ENDOTOXIN-INDUCED DISSEMINATED INTRAVASCULAR COAGULATION [J]. Shock, 2015,44(4): 341-347.

[78] Ducci RDP, Lange MC, Zetola VDF. Predictors of in-hospital mortality and dependence at discharge in patients with MCA stroke with intravenous thrombolysis [J]. Internal and Emergency Medicine, 2017,12(4): 453-460.

[79] Dudzinski DM, Giri J, Rosenfield K. Interventional Treatment of Pulmonary Embolism [J]. Circulation-Cardiovascular Interventions, 2017,10(2).

[80] Duman E, Coven I, Yildirim E, et al. Endovascular Treatment of Wide Necked Ruptured Saccular Aneurysms with Flow-Diverter Stent [J]. Turkish Neurosurgery, 2017,27(3): 362-367.

[81] Dunne WM. Laboratory Diagnosis of Sepsis? No SIRS, Not Just Yet [J]. Journal of Clinical Microbiology, 2015,53(8): 2404-2409.

[82] Durila M, Harustiak T. Coagulation profile after esophagectomy in SIRS and sepsis evaluated by thromboelastography and relationship with organ dysfunction development [J]. Bratislava Medical Journal-Bratislavske Lekarske Listy, 2016,117(6): 312-315.

[83] Eddy JL, Schroeder JA, Zimbler DL, et al. Proteolysis of plasminogen activator inhibitor-1 by Yersinia pestis remodulates the host environment to promote virulence [J]. Journal of Thrombosis and Haemostasis, 2016,14(9): 1833-1843.

[84] Ehrnthaller C, Flierl M, Perl M, et al. The molecular fingerprint of lung inflammation after blunt chest trauma [J]. European Journal of Medical Research, 2015,20.

[85] Eichhorn T, Rauscher S, Hammer C, et al. Polystyrene-Divinylbenzene-Based Adsorbents Reduce Endothelial Activation and Monocyte Adhesion Under Septic Conditions in a Pore Size-Dependent Manner [J]. Inflammation, 2016,39(5): 1737-1746.

[86] El-Mekkawy MS, Saleh NY, Sonbol AA. Soluble Urokinase Plasminogen Activator Receptor: A New Biomarker in the Pediatric Intensive Care Unit [J]. Indian Journal of Pediatrics, 2016,83(7): 661-669.

[87] Eugen-Olsen J, Giamarellos-Bourboulis EJ. suPAR: The unspecific marker for disease presence, severity and prognosis [J]. International Journal of Antimicrobial Agents, 2015,46: S33-S34.

[88] Fanelli V, Morita Y, Cappello P, et al. Neuromuscular Blocking Agent Cisatracurium Attenuates Lung Injury by Inhibition of Nicotinic Acetylcholine Receptor-alpha 1 [J]. Anesthesiology, 2016,124(1): 132-140.

[89] Ferreiro L, San Jose ME, Valdes L. Management of Parapneumonic Pleural Effusion in Adults [J]. Archivos De Bronconeumologia, 2015,51(12): 637-646.

[90] Fitzgerald DB, Lee YCG. Pleural infection: To drain or not to drain? [J]. Respirology, 2017,22(6): 1055-1056.

[91] Fiusa MML, Carvalho MA, Annichino-Bizzacchi JM, et al. Causes and consequences of coagulation activation in sepsis: an evolutionary medicine perspective [J]. Bmc Medicine, 2015,13.

[92] Fletcher DJ, Rozanski EA, Brainard BM, et al. Assessment of the relationships among coagulopathy, hyperfibrinolysis, plasma lactate, and protein C in dogs with spontaneous hemoperitoneum [J]. Journal of Veterinary Emergency and Critical Care, 2016,26(1): 41-51.

[93] Florova G, Azghani A, Karandashova S, et al. Targeting of Plasminogen Activator Inhibitor 1 Improves Fibrinolytic Therapy for Tetracycline-Induced Pleural Injury in Rabbits [J]. American Journal of Respiratory Cell and Molecular Biology, 2015,52(4): 429-437.

[94] Fratanduono DE, Celliers PM, Braun DG, et al. Equation of state, adiabatic sound speed, and Gruneisen coefficient of boron carbide along the principal Hugoniot to 700 GPa [J]. Physical Review B, 2016,94(18).

[95] Fu Y, Gaelings L, Jalovaara P, et al. Protein profiling of nasopharyngeal aspirates of hospitalized and outpatients revealed cytokines associated with severe influenza A(H1N1)pdm09 virus infections: A pilot study [J]. Cytokine, 2016,86: 10-14.

[96] Funk J, Schaarschmidt B, Slesiona S, et al. The glycolytic enzyme enolase represents a plasminogen-binding protein on the surface of a wide variety of medically important fungal species [J]. International Journal of Medical Microbiology, 2016,306(1): 59-68.

[97] Gall LS, Brohi K, Davenport RA. Diagnosis and Treatment of Hyperfibrinolysis in Trauma (A European Perspective) [J]. Seminars in Thrombosis and Hemostasis, 2017,43(2): 224-234.

[98] Gando S, Levi M, Toh CH. Disseminated intravascular coagulation [J]. Nature Reviews Disease Primers, 2016,2.

[99] Gao YW, Gao WS, Chen X, et al. Enhancing the treatment effect on melanoma by heat shock protein 70-peptide complexes purified from human melanoma cell lines [J]. Oncology Reports, 2016,36(3): 1243-1250.

[100] Geboers D, de Beer FM, Tuip-de Boer AM, et al. Plasma suPAR as a prognostic biological marker for ICU mortality in ARDS patients [J]. Intensive Care Medicine, 2015,41(7): 1281-1290.

[101] Ghasemzadeh N, Hayek SS, Ko YA, et al. Pathway-Specific Aggregate Biomarker Risk Score Is Associated With Burden of Coronary Artery Disease and Predicts Near-Term Risk of Myocardial Infarction and Death [J]. Circulation-Cardiovascular Quality and Outcomes, 2017,10(3).

[102] Ghosh S, Hoselton SA, Dorsam GP, et al. Hyaluronan fragments as mediators of inflammation in allergic pulmonary disease [J]. Immunobiology, 2015,220(5): 575-588.

[103] Gilbert CR, Gorden JA. Use of intrapleural tissue plasminogen activator and deoxyribonuclease in pleural space infections: an update on alternative regimens [J]. Current Opinion in Pulmonary Medicine, 2017,23(4): 371-375.

[104] Giuffre M, Lo Verso C, Serra G, et al. Portal Vein Thrombosis in a Preterm Newborn with Mutation of the MTHFR and PAI-1 Genes and Sepsis by Candida parapsilosis [J]. American Journal of Perinatology, 2016,33(11): 1099-1103.

[105] Gombedza F, Kondeti V, Al-Azzam N, et al. Mechanosensitive transient receptor potential vanilloid 4 regulates Dermatophagoides farinae-induced airway remodeling via 2 distinct pathways modulating matrix synthesis and degradation [J]. Faseb Journal, 2017,31(4): 1556-1570.

[106] Gomez-Salinero JM, Rafii S. Plasmin regulation of acute cytokine storm [J]. Blood, 2017,130(1): 5-6.

[107] Gong XQ, Duan R, Ao JE, et al. Metformin suppresses intrahepatic coagulation activation in mice with lipopolysaccharide/D-galactosamine-induced fulminant hepatitis [J]. Molecular Medicine Reports, 2015,12(4): 6384-6390.

[108] Gopinathan U, Brusletto BS, Olstad OK, et al. IL-10 immunodepletion from meningococcal sepsis plasma induces extensive changes in gene expression and cytokine release in stimulated human monocytes [J]. Innate Immunity, 2015,21(4): 429-449.

[109] Goswami S, Kumar RR, Sharma SK, et al. Calcium triggers protein kinases-induced signal transduction for augmenting the thermotolerance of developing wheat (Triticum aestivum) grain under the heat stress [J]. Journal of Plant Biochemistry and Biotechnology, 2015,24(4): 441-452.

[110] Gould TJ, Vu TT, Stafford AR, et al. Cell-Free DNA Modulates Clot Structure and Impairs Fibrinolysis in Sepsis [J]. Arteriosclerosis Thrombosis and Vascular Biology, 2015,35(12): 2544-2553.

[111] Griffin JH, Zlokovic BV, Mosnier LO. Activated protein C: biased for translation [J]. Blood, 2015,125(19): 2898-2907.

[112] Grundel A, Pfeiffer M, Jacobs E, et al. Network of Surface-Displayed Glycolytic Enzymes in Mycoplasma pneumoniae and Their Interactions with Human Plasminogen [J]. Infection and Immunity, 2016,84(3): 666-676.

[113] Gu HM, Fisher AJ, Mickler EA, et al. Contribution of the anaphylatoxin receptors, C3aR and C5aR, to the pathogenesis of pulmonary fibrosis [J]. Faseb Journal, 2016,30(6): 2336-2350.

[114] Gui L, Zhang PP, Liang XM, et al. Adaptive responses to osmotic stress in kidney-derived cell lines from Scatophagus argus, a euryhaline fish [J]. Gene, 2016,583(2): 134-140.

[115] Gul U, Hussain S, Munir R, et al. Neutrophil Lymphocyte Ratio: A Prognostic Marker in Acute ST Elevation Myocardial Infarction [J]. Jcpsp-Journal of the College of Physicians and Surgeons Pakistan, 2017,27(1): 4-7.

[116] Guo YQ, Zhang WW, Zheng LN, et al. Impacts of dynamic mechanical stretch on the expression of plasminogen activator inhibitor-1 (PAI-1) in human A549 cells [J]. International Journal of Clinical and Experimental Pathology, 2016,9(6): 5871-5881.

[117] Gupta KK, Donahue DL, Sandoval-Cooper MJ, et al. Abrogation of Plasminogen Activator Inhibitor-1-Vitronectin Interaction Ameliorates Acute Kidney Injury in Murine Endotoxemia [J]. Plos One, 2015,10(3).

[118] Gupta KK, Xu Z, Castellino FJ, et al. Plasminogen activator inhibitor-1 stimulates macrophage activation through Toll-like Receptor-4 [J]. Biochemical and Biophysical Research Communications, 2016,477(3): 503-508.

[119] Haas V, Bayerstorfer R, Kuhtin O, et al. Significance of Thoracic Surgery for Treatment of Pleural Empyema in Childhood and Adolescence [J]. Klinische Padiatrie, 2016,228(1): 29-34.

[120] Hafen GM, Grenzbach AC, Moeller A, et al. Lack of concordance in parapneumonic effusion management in children in central Europe [J]. Pediatric Pulmonology, 2016,51(4): 411-417.

[121] Han CK, Tien YC, Hsieh DJY, et al. Attenuation of the LPS-induced, ERK-mediated upregulation of fibrosis-related factors FGF-2, uPA, MMP-2, and MMP-9 by Carthamus tinctorius L in cardiomyoblasts [J]. Environmental Toxicology, 2017,32(3): 754-763.

[122] Han JH, Nachamkin I, Coffin SE, et al. Use of a Combination Biomarker Algorithm To Identify Medical Intensive Care Unit Patients with Suspected Sepsis at Very Low Likelihood of Bacterial Infection [J]. Antimicrobial Agents and Chemotherapy, 2015,59(10): 6494-6500.

[123] Hashimoto K, Kim H, Oishi H, et al. Annexin V homodimer protects against ischemia reperfusion-induced acute lung injury in lung transplantation [J]. Journal of Thoracic and Cardiovascular Surgery, 2016,151(3): 861-869.

[124] Heimes J, Copeland H, Lulla A, et al. The use of thrombolytics in the management of complex pleural fluid collections [J]. Journal of Thoracic Disease, 2017,9(5): 1310-1316.

[125] Hendaus MA, Janahi IA. Parapneumonic Effusion in Children: An Up-to-Date Review [J]. Clinical Pediatrics, 2016,55(1): 10-18.

[126] Hilty MP, Zugel S, Schoeb M, et al. Soluble Urokinase-Type Plasminogen Activator Receptor Plasma Concentration May Predict Susceptibility to High Altitude Pulmonary Edema [J]. Mediators of Inflammation, 2016.

[127] Hohensinner PJ, Baumgartner J, Kral-Pointner JB, et al. PAI-1 (Plasminogen Activator Inhibitor-1) Expression Renders Alternatively Activated Human Macrophages Proteolytically Quiescent [J]. Arteriosclerosis Thrombosis and Vascular Biology, 2017,37(10): 1913-+.

[128] Hoshino K, Kawano Y, Maruyama J, et al. PLASMINOGEN ACTIVATOR INHIBITOR-1 IS THE MOST PREDICTIVE MARKER OF MORTALITY IN SEPSIS [J]. Critical Care Medicine, 2016,44(12).

[129] Hou JW, Li X, Li CF, et al. Plasma membrane gp96 enhances invasion and metastatic potential of liver cancer via regulation of uPAR [J]. Molecular Oncology, 2015,9(7): 1312-1323.

[130] Hou PC, Filbin MR, Wang H, et al. Endothelial Permeability and Hemostasis in Septic Shock Results From the ProCESS Trial [J]. Chest, 2017,152(1): 22-31.

[131] Hourmouzis Z, Bhalla MC, Frey JA, et al. Pulmonary embolism and heparin-induced thrombocytopenia successfully treated with tissue plasminogen activator and argatroban [J]. American Journal of Emergency Medicine, 2015,33(5).

[132] Huang J, Tian R, Yang YQ, et al. The SIRT1 inhibitor EX-527 suppresses mTOR activation and alleviates acute lung injury in mice with endotoxiemia [J]. Innate Immunity, 2017,23(8): 678-686.

[133] Huebner BR, Moore EE, Moore HB, et al. Freeze-dried plasma enhances clot formation and inhibits fibrinolysis in the presence of tissue plasminogen activator similar to pooled liquid plasma [J]. Transfusion, 2017,57(8): 2007-2015.

[134] Hughes CG, Pandharipande PP, Thompson JL, et al. Endothelial Activation and Blood-Brain Barrier Injury as Risk Factors for Delirium in Critically Ill Patients [J]. Critical Care Medicine, 2016,44(9): E809-E817.

[135] Hwang I, Lee JW, Kim JS, et al. Surfactant toxicity in a case of (4-chloro-2-methylphenoxy) acetic acid herbicide intoxication [J]. Human & Experimental Toxicology, 2015,34(8): 848-855.

[136] Ichiyanagi K, Nakamura KG. Structural Dynamics of Materials under Shock Compression Investigated with Synchrotron Radiation [J]. Metals, 2016,6(1).

[137] Idell RD, Florova G, Komissarov AA, et al. The fibrinolytic system: A new target for treatment of depression with psychedelics [J]. Medical Hypotheses, 2017,100: 46-53.

[138] Ilhan N, Susam S, Gul HF, et al. Which one is more effective for the treatment of rat sepsis model: thalidomide or etanercept? [J]. Bratislava Medical Journal-Bratislavske Lekarske Listy, 2017,118(5): 283-287.

[139] Isik DU, Celik IH, Yilmaz O, et al. A Previously Healthy Premature Infant Treated With Thrombolytic Therapy for Life-threatening Pulmonary Artery Thrombosis [J]. Journal of Pediatric Hematology Oncology, 2016,38(8): E319-E321.

[140] Iswandana R, Pham BT, van Haaften WT, et al. Organ- and species-specific biological activity of rosmarinic acid [J]. Toxicology in Vitro, 2016,32: 261-268.

[141] Ito T, Kakihana Y, Maruyama I. Thrombomodulin as an intravascular safeguard against inflammatory and thrombotic diseases [J]. Expert Opinion on Therapeutic Targets, 2016,20(2): 151-158.

[142] Jagadish N, Agarwal S, Gupta N, et al. Heat shock protein 70-2 (HSP70-2) overexpression in breast cancer [J]. Journal of Experimental & Clinical Cancer Research, 2016,35.

[143] James CA, Braswell LE, Pezeshkmehr AH, et al. Stratifying fibrinolytic dosing in pediatric parapneumonic effusion based on ultrasound grade correlation [J]. Pediatric Radiology, 2017,47(1): 89-95.

[144] Jia XF, Hong Z, Fan JH, et al. CLINICAL EFFECT OF MECHANICAL FRAGMENTATION COMBINED WITH RECOMBINANT TISSUE PLASMINOGEN ACTIVATOR ARTERY THROMBOLYSIS ON ACUTE CEREBRAL INFARCTION [J]. Journal of Biological Regulators and Homeostatic Agents, 2016,30(3): 821-826.

[145] Jo AR, Jeong HS, Kim MK, et al. Geranylgeranylacetone induces apoptosis via the intrinsic pathway in human melanoma cells [J]. Biomedicine & Pharmacotherapy, 2016,82: 15-19.

[146] Joseph K, Tholanikunnel BG, Kaplan AP. Cytokine and estrogen stimulation of endothelial cells augments activation of the prekallikrein-high molecular weight kininogen complex: Implications for hereditary angioedema [J]. Journal of Allergy and Clinical Immunology, 2017,140(1): 170-176.

[147] Kalaiarasu LP, Subramanian V, Sowndharrajan B, et al. Insight into the Anti-Inflammatory Mechanism of Action of Atrial Natriuretic Peptide, a Heart Derived Peptide Hormone: Involvement of COX-2, MMPs, and NF-kB Pathways [J]. International Journal of Peptide Research and Therapeutics, 2016,22(4): 451-463.

[148] Kania A, Kudlicki J, Frania-Baryluk A, et al. Double thrombolysis in early pregnancy can be safe [J]. Kardiologia Polska, 2016,74: 29-33.

[149] Kara I, Pampal HK, Yildirim F, et al. Role of ischemic modified albumin in the early diagnosis of increased intracranial pressure and brain death [J]. Bratislava Medical Journal-Bratislavske Lekarske Listy, 2017,118(2): 112-117.

[150] Kartal O, Kartal AT. Value of neutrophil to lymphocyte and platelet to lymphocyte ratios in pneumonia [J]. Bratislava Medical Journal-Bratislavske Lekarske Listy, 2017,118(9): 513-516.

[151] Katayama S, Nunomiya S, Koyama K, et al. Markers of acute kidney injury in patients with sepsis: the role of soluble thrombomodulin [J]. Critical Care, 2017,21.

[152] Kelly BJ, Lautenbach E, Nachamkin I, et al. Combined biomarkers discriminate a low likelihood of bacterial infection among surgical intensive care unit patients with suspected sepsis [J]. Diagnostic Microbiology and Infectious Disease, 2016,85(1): 109-115.

[153] Kerschen E, Hernandez I, Zogg M, et al. Survival advantage of heterozygous factor V Leiden carriers in murine sepsis [J]. Journal of Thrombosis and Haemostasis, 2015,13(6): 1073-1080.

[154] Kikkert WJ, Hassell M, Delewi R, et al. Predictors and prognostic consequence of gastrointestinal bleeding in patients with ST-segment elevation myocardial infarction [J]. International Journal of Cardiology, 2015,184: 128-134.

[155] Kim MJ, Kim WS, Kim DO, et al. Macrophage migration inhibitory factor interacts with thioredoxin-interacting protein and induces NF-kappa B activity [J]. Cellular Signalling, 2017,34: 110-120.

[156] Kim SJ, Cheresh P, Jablonski RP, et al. The Role of Mitochondrial DNA in Mediating Alveolar Epithelial Cell Apoptosis and Pulmonary Fibrosis [J]. International Journal of Molecular Sciences, 2015,16(9): 21486-21519.

[157] Kim SR, Ha YM, Kim YM, et al. Ascorbic acid reduces HMGB1 secretion in lipopolysaccharide-activated RAW 264.7 cells and improves survival rate in septic mice by activation of Nrf2/HO-1 signals [J]. Biochemical Pharmacology, 2015,95(4): 279-289.

[158] Kimura D, Saravia J, Rovnaghi CR, et al. Plasma Biomarker Analysis in Pediatric ARDS: Generating Future Framework from a Pilot Randomized Control Trial of Methylprednisolone: A Framework for Identifying Plasma Biomarkers Related to Clinical Outcomes in Pediatric ARDS [J]. Frontiers in Pediatrics, 2016,4.

[159] Kingsley SMK, Bhat BV. Role of microRNAs in sepsis [J]. Inflammation Research, 2017,66(7): 553-569.

[160] Klein D, Schmetter A, Imsak R, et al. Therapy with Multipotent Mesenchymal Stromal Cells Protects Lungs from Radiation-Induced Injury and Reduces the Risk of Lung Metastasis [J]. Antioxidants & Redox Signaling, 2016,24(2): 53-69.

[161] Knudson MD, Desjarlais MP, Lemke RW. Shock compression experiments on Lithium Deuteride (LiD) single crystals [J]. Journal of Applied Physics, 2016,120(23).

[162] Knudson MD, Desjarlais MP, Pribram-Jones A. Adiabatic release measurements in aluminum between 400 and 1200 GPa: Characterization of aluminum as a shock standard in the multimegabar regime [J]. Physical Review B, 2015,91(22).

[163] Koami H, Sakamoto Y, Furukawa T, et al. Utility of rotational thromboelastometry for the diagnosis of asymptomatic hyperfibrinolysis secondary to anaphylaxis [J]. Blood Coagulation & Fibrinolysis, 2016,27(4): 450-453.

[164] Koami H, Sakamoto Y, Miyasho T, et al. Haptoglobin Reduces Inflammatory Cytokine INF-gamma and Facilitates Clot Formation in Acute Severe Burn Rat Model [J]. Journal of Nippon Medical School, 2017,84(2): 64-72.

[165] Koenigs A, Zipfel PF, Kraiczy P. Translation Elongation Factor Tuf of Acinetobacter baumannii Is a Plasminogen-Binding Protein [J]. Plos One, 2015,10(7).

[166] Komissarov AA, Florova G, Azghani AO, et al. Dose dependency of outcomes of intrapleural fibrinolytic therapy in new rabbit empyema models [J]. American Journal of Physiology-Lung Cellular and Molecular Physiology, 2016,311(2): L389-L399.

[167] Komissarov AA, Florova G, Azghani AO, et al. The time course of resolution of adhesions during fibrinolytic therapy in tetracycline-induced pleural injury in rabbits [J]. American Journal of Physiology-Lung Cellular and Molecular Physiology, 2015,309(6): L562-L572.

[168] Konstantinides SV, Warntges S. Acute phase treatment of venous thromboembolism: advanced therapy Systemic fibrinolysis and pharmacomechanical therapy [J]. Thrombosis and Haemostasis, 2015,113(6): 1202-1209.

[169] Koppurapu V, Meena N. A review of the management of complex para-pneumonic effusion in adults [J]. Journal of Thoracic Disease, 2017,9(7): 2135-2141.

[170] Kosova F, Kasar Z, Tuglu I, et al. Apoptosis of colon cancer cells under the effect of geldanamycin derivate [J]. Bratislava Medical Journal-Bratislavske Lekarske Listy, 2017,118(5): 288-291.

[171] Kraus D, Chapman DA, Kritcher AL, et al. X-ray scattering measurements on imploding CH spheres at the National Ignition Facility [J]. Physical Review E, 2016,94(1).

[172] Krenke K, Urbankowska E, Urbankowski T, et al. Clinical characteristics of 323 children with parapneumonic pleural effusion and pleural empyema due to community acquired pneumonia [J]. Journal of Infection and Chemotherapy, 2016,22(5): 292-297.

[173] Krychtiuk KA, Kaun C, Hohensinner PJ, et al. Anti-thrombotic and pro-fibrinolytic effects of levosimendan in human endothelial cells in vitro [J]. Vascular Pharmacology, 2017,90: 44-50.

[174] Kuchler L, Sha LK, Giegerich AK, et al. Elevated intrathymic sphingosine-1-phosphate promotes thymus involution during sepsis [J]. Molecular Immunology, 2017,90: 255-263.

[175] Kumar RR, Sharma SK, Goswami S, et al. Salicylic acid alleviates the heat stress-induced oxidative damage of starch biosynthesis pathway by modulating the expression of heat-stable genes and proteins in wheat (Triticum aestivum) [J]. Acta Physiologiae Plantarum, 2015,37(8).

[176] Kumar V, Sinha S, Kumar P, et al. Short-term outcome of acute inferior wall myocardial infarction with emphasis on conduction blocks: a prospective observational study in Indian population [J]. Anatolian Journal of Cardiology, 2017,17(3): 229-234.

[177] Lahiri S, Schlick K, Kavi T, et al. Optimizing Outcomes for Mechanically Ventilated Patients in an Era of Endovascular Acute Ischemic Stroke Therapy [J]. Journal of Intensive Care Medicine, 2017,32(8): 467-472.

[178] Lamas-Pinheiro R, Henriques-Coelho T, Fernandes S, et al. Thoracoscopy in the management of pediatric empyemas [J]. Revista Portuguesa De Pneumologia, 2016,22(3): 157-162.

[179] Lawton SK, Xu F, Tran A, et al. N-Arachidonoyl Dopamine Modulates Acute Systemic Inflammation via Nonhematopoietic TRPV1 [J]. Journal of Immunology, 2017,199(4): 1465-1475.

[180] Lazicki A, London RA, Coppari F, et al. Shock equation of state of (LiH)-Li-6 to 1.1 TPa [J]. Physical Review B, 2017,96(13).

[181] Lecuyer H, Borgel D, Nassif X, et al. Pathogenesis of meningococcal purpura fulminans [J]. Pathogens and Disease, 2017,75(3).

[182] Lee JA, Yerbury JJ, Farrawell N, et al. SerpinB2 (PAI-2) Modulates Proteostasis via Binding Misfolded Proteins and Promotion of Cytoprotective Inclusion Formation [J]. Plos One, 2015,10(6).

[183] Lee YCG, Idell S, Stathopoulos GT. Translational Research in Pleural Infection and Beyond [J]. Chest, 2016,150(6): 1361-1370.

[184] Leeper CM, Neal MD, McKenna CJ, et al. Trending Fibrinolytic Dysregulation Fibrinolysis Shutdown in the Days After Injury Is Associated With Poor Outcome in Severely Injured Children [J]. Annals of Surgery, 2017,266(3): 508-515.

[185] Leonard MC, Bauer SR, Ahrens C, et al. Institutional care paths: Development, implementation, and evaluation [J]. American Journal of Health-System Pharmacy, 2017,74(18): 1486-1493.

[186] Letheulle J, Kerjouan M, Benezit F, et al. Parapneumonic pleural effusions: Epidemiology, diagnosis, classification and management [J]. Revue Des Maladies Respiratoires, 2015,32(4): 344-357.

[187] Levi M. Management of cancer-associated disseminated intravascular coagulation [J]. Thrombosis Research, 2016,140: S66-S70.

[188] Levi M, van der Poll T. Coagulation and sepsis [J]. Thrombosis Research, 2017,149: 38-44.

[189] Li J, Mzimbiri JM, Zhao J, et al. Surviving the Largest Atypical Parasagittal Meningioma in a 2-Year-Old Child: A Case Report and a Brief Review of the Literature [J]. World Neurosurgery, 2016,87.

[190] Li L, Chen XD, Shi L, et al. A Proteome Translocation Response to Complex Desert Stress Environments in Perennial Phragmites Sympatric Ecotypes with Contrasting Water Availability [J]. Frontiers in Plant Science, 2017,8: 1-15.

[191] Li LF, Lee CS, Lin CW, et al. Trichostatin A attenuates ventilation-augmented epithelial-mesenchymal transition in mice with bleomycin-induced acute lung injury by suppressing the Akt pathway [J]. Plos One, 2017,12(2).

[192] Li M, Wang JX, Yuan T, et al. Dynamic high pressure generation through plasma implosion driven by an intense laser pulse [J]. Aip Advances, 2017,7(3).

[193] Li N, Ouyang BS, Liu L, et al. Dexmedetomidine protected COPD-induced lung injury by regulating miRNA-146a [J]. Bratislava Medical Journal-Bratislavske Lekarske Listy, 2016,117(9): 539-542.

[194] Liao YT, Wang SM, Wang JR, et al. Norepinephrine and Epinephrine Enhanced the Infectivity of Enterovirus 71 [J]. Plos One, 2015,10(8).

[195] Lieber BA, Han J, Appelboom G, et al. Association of Steroid Use with Deep Venous Thrombosis and Pulmonary Embolism in Neurosurgical Patients: A National Database Analysis [J]. World Neurosurgery, 2016,89: 126-132.

[196] Lin CY, Hu CT, Cheng CC, et al. Oxidation of heat shock protein 60 and protein disulfide isomerase activates ERK and migration of human hepatocellular carcinoma HepG2 [J]. Oncotarget, 2016,7(10): 11067-11082.

[197] Lin MT, Jian MY, Taylor MS, et al. Functional coupling of TRPV4, IK, and SK channels contributes to Ca2+-dependent endothelial injury in rodent lung [J]. Pulmonary Circulation, 2015,5(2): 279-290.

[198] Lin TJ, Lin HT, Chang WT, et al. Shikonin-enhanced cell immunogenicity of tumor vaccine is mediated by the differential effects of DAMP components [J]. Molecular Cancer, 2015,14.

[199] Lin XF, Wu M, Liu B, et al. Candesartan ameliorates acute myocardial infarction in rats through inducible nitric oxide synthase, nuclear factor-B, monocyte chemoattractant protein-1, activator protein-1 and restoration of heat shock protein 72 [J]. Molecular Medicine Reports, 2015,12(6): 8193-8200.

[200] Liu B, Tang Y, Yi MX, et al. Genetic variants in the plasminogen activator inhibitor-1 gene are associated with an increased risk of radiation pneumonitis in lung cancer patients [J]. Cancer Medicine, 2017,6(3): 681-688.

[201] Liu FB, Lin Q, Liu ZW. A study on the role of apoptotic human umbilical cord mesenchymal stem cells in bleomycin-induced acute lung injury in rat models [J]. European Review for Medical and Pharmacological Sciences, 2016,20(5): 969-982.

[202] Liu HX. Plasminogen activator inhibitor-1 gene is associated with radioactive lung injury in lung cancer patients [J]. Biomedical Research-India, 2017,28(15): 6541-6545.

[203] Liu RM, Desai LP. Reciprocal regulation of TGF-beta and reactive oxygen species: A perverse cycle for fibrosis [J]. Redox Biology, 2015,6: 565-577.

[204] Liu X, Shen Y, Li ZH, et al. Prognostic significance of APACHE II score and plasma suPAR in Chinese patients with sepsis: a prospective observational study [J]. Bmc Anesthesiology, 2016,16.

[205] Liu Y, Hou JH, Li Q, et al. Biomarkers for diagnosis of sepsis in patients with systemic inflammatory response syndrome: a systematic review and meta-analysis [J]. Springerplus, 2016,5.

[206] Livingston MH, Cohen E, Giglia L, et al. Are some children with empyema at risk for treatment failure with fibrinolytics? A multicenter cohort study [J]. Journal of Pediatric Surgery, 2016,51(5): 832-837.

[207] Loonen AJM, Kesarsing C, Kusters R, et al. High pneumococcal DNA load, procalcitonin and suPAR levels correlate to severe disease development in patients with pneumococcal pneumonia [J]. European Journal of Clinical Microbiology & Infectious Diseases, 2017,36(9): 1541-1547.

[208] Lorente L, Martin MM, Borreguero-Leon JM, et al. The 4G/4G Genotype of PAI-1 Polymorphism Is Associated with Higher Plasma PAI-1 Concentrations and Mortality in Patients with Severe Sepsis [J]. Plos One, 2015,10(6).

[209] Ludwig KR, Hummon AB. Mass spectrometry for the discovery of biomarkers of sepsis [J]. Molecular Biosystems, 2017,13(4): 648-664.

[210] Lukasiewicz A, Lichota W, Thews M. Outcomes of accelerated catheter-directed thrombolysis in patients with acute arterial thrombosis [J]. Vascular Medicine, 2016,21(5): 453-458.

[211] Lumbera WML, Vergara EJS, Kim YS, et al. Anonna squamosa leaves ethanolic extract mediates apoptosis through heat shock protein mitigation and G2/M cell cycle arrest in Human Hepatocellular carcinoma (SK-Hepi) cells [J]. Faseb Journal, 2016,30.

[212] Lumbera WML, Vergara EJS, Kim YS, et al. Anonna squamosa leaves ethanolic extract mediates apoptosis through heat shock protein mitigation and G2/M cell cycle arrest in Human Hepatocellular carcinoma (SK-Hep1) cells [J]. Faseb Journal, 2016,30.

[213] Lyden P, Hemmen T, Grotta J, et al. Results of the ICTuS 2 Trial (Intravascular Cooling in the Treatment of Stroke 2) [J]. Stroke, 2016,47(12): 2888-2895.

[214] Ma SB, Zhao HP, Ji XM, et al. Peripheral to central: Organ interactions in stroke pathophysiology [J]. Experimental Neurology, 2015,272: 41-49.

[215] Makki N, Brennan TM, Girotra S. Acute Coronary Syndrome [J]. Journal of Intensive Care Medicine, 2015,30(4): 186-200.

[216] Mantuano E, Azmoon P, Brifault C, et al. Tissue-type plasminogen activator regulates macrophage activation and innate immunity [J]. Blood, 2017,130(11): 1364-1374.

[217] Mao LQ, Li XB. Effects of N-acetyl cysteine to improve acute lung injury in rats [J]. Bratislava Medical Journal-Bratislavske Lekarske Listy, 2017,118(9): 552-556.

[218] Marin A, Gazmuri JT, Andresen M, et al. Thrombolytic therapy for submassive/intermediate risk Pulmonary Embolism Evidence and suggestions after PEITHO trial [J]. Revista Medica De Chile, 2015,143(7): 895-904.

[219] Markelova EV, Romanchuk AL, Prosekova EV, et al. Assessing the level of matrix metal proteinases 1,8,9, their tissue inhibitor, type I, in cases of odontogenic phlegmons [J]. Bratislava Medical Journal-Bratislavske Lekarske Listy, 2017,118(1): 51-55.

[220] Martin JV, Liberati DM, Diebel LN. Disparate effects of catecholamines under stress conditions on endothelial glycocalyx injury: An in vitro model [J]. American Journal of Surgery, 2017,214(6): 1166-1172.

[221] Marudamuthu AS, Shetty SK, Bhandary YP, et al. Plasminogen Activator Inhibitor-1 Suppresses Profibrotic Responses in Fibroblasts from Fibrotic Lungs [J]. Journal of Biological Chemistry, 2015,290(15): 9428-9441.

[222] Matalon S, Bartoszewski R, Collawn JF. Role of epithelial sodium channels in the regulation of lung fluid homeostasis [J]. American Journal of Physiology-Lung Cellular and Molecular Physiology, 2015,309(11): L1229-L1238.

[223] Matsumoto H, Yamakawa K, Ogura H, et al. CLINICAL SIGNIFICANCE OF TISSUE FACTOR AND CD13 DOUBLE-POSITIVE MICROPARTICLES IN SIRS PATIENTS WITH TRAUMA AND SEVERE SEPSIS [J]. Shock, 2017,47(4): 409-415.

[224] Matzkies LM, Raggam RB, Flick H, et al. Prognostic and diagnostic potential of suPAR levels in pleural effusion [J]. Journal of Infection, 2017,75(5): 465-467.

[225] McCoy CA, Gregor MC, Polsin DN, et al. Shock-wave equation-of-state measurements in fused silica up to 1600 GPa [J]. Journal of Applied Physics, 2016,119(21).

[226] McNair E, Marcoux JA, Bally C, et al. Bivalirudin as an adjunctive anticoagulant to heparin in the treatment of heparin resistance during cardiopulmonary bypass-assisted cardiac surgery [J]. Perfusion-Uk, 2016,31(3): 189-199.

[227] Mehta HJ, Biswas A, Penley AM, et al. Management of Intrapleural Sepsis with Once Daily Use of Tissue Plasminogen Activator and Deoxyribonuclease [J]. Respiration, 2016,91(2): 101-106.

[228] Mehta HJ, Biswas A, Penley AM, et al. Utilizing Once Daily Use Of Tissue Plasminogen Activator (tpa) And Deoxyribonuclease (dnase) For Management Of Intrapleural Sepsis [J]. American Journal of Respiratory and Critical Care Medicine, 2016,193.

[229] Milani A, Bolhassani A, Heshmati M. Delivery of HIV-1 Nef linked to heat shock protein 27 using a cationic polymer is more effective than cationic lipid in mammalian cells [J]. Bratislava Medical Journal-Bratislavske Lekarske Listy, 2017,118(6): 334-338.

[230] Minakov DV, Levashov PR. Thermodynamic properties of LiD under compression with different pseudopotentials for lithium [J]. Computational Materials Science, 2016,114: 128-134.

[231] Mishra D, Shekhar S, Agrawal L, et al. Cultivar-specific high temperature stress responses in bread wheat (Triticum aestivum L.) associated with physicochemical traits and defense pathways [J]. Food Chemistry, 2017,221: 1077-1087.

[232] Mithal LB, Palac HL, Yogev R, et al. Cord Blood Acute Phase Reactants Predict Early Onset Neonatal Sepsis in Preterm Infants [J]. Plos One, 2017,12(1).

[233] Miyanishi K, Tange Y, Ozaki N, et al. Laser-shock compression of magnesium oxide in the warm-dense-matter regime [J]. Physical Review E, 2015,92(2).

[234] Miyashita T, Ahmed AK, Nakanuma S, et al. A Three-phase Approach for the Early Identification of Acute Lung Injury Induced by Severe Sepsis [J]. In Vivo, 2016,30(4): 341-349.

[235] Monagle K, Ryan A, Hepponstall M, et al. Inhalational use of antithrombotics in humans: Review of the literature [J]. Thrombosis Research, 2015,136(6): 1059-1066.

[236] Moore EE, Moore HB, Gonzalez E, et al. Rationale for the selective administration of tranexamic acid to inhibit fibrinolysis in the severely injured patient [J]. Transfusion, 2016,56: S110-S114.

[237] Moore HB, Moore EE, Chapman MP, et al. Viscoelastic measurements of platelet function, not fibrinogen function, predicts sensitivity to tissue-type plasminogen activator in trauma patients [J]. Journal of Thrombosis and Haemostasis, 2015,13(10): 1878-1887.

[238] Moore HB, Moore EE, Chapman MP, et al. Viscoelastic Tissue Plasminogen Activator Challenge Predicts Massive Transfusion in 15 Minutes [J]. Journal of the American College of Surgeons, 2017,225(1): 138-147.

[239] Moore HB, Moore EE, Gonzalez E, et al. Reperfusion Shutdown: Delayed Onset of Fibrinolysis Resistance after Resuscitation from Hemorrhagic Shock Is Associated with Increased Circulating Levels of Plasminogen Activator Inhibitor-1 and Postinjury Complications [J]. Blood, 2016,128(22).

[240] Moore HB, Moore EE, Lawson PJ, et al. Fibrinolysis shutdown phenotype masks changes in rodent coagulation in tissue injury versus hemorrhagic shock [J]. Surgery, 2015,158(2): 386-392.

[241] Moore HB, Moore EE, Morton AP, et al. HEMORRHAGIC SHOCK STIUMLATES ORGAN TISSUE RELEASE OF TPA INDEPENDENT OF DISSEMINATED INTRAVASCULAR COAGULATION [J]. Shock, 2015,43(6): 101-101.

[242] Moore HB, Moore EE, Morton AP, et al. Shock-induced systemic hyperfibrinolysis is attenuated by plasma-first resuscitation [J]. Journal of Trauma and Acute Care Surgery, 2015,79(6): 897-903.

[243] Morozov AV, Yurinskaya MM, Mitkevich VA, et al. Heat-Shock Protein HSP70 Decreases Activity of Proteasomes in Human Neuroblastoma Cells Treated by Amyloid-Beta 1-42 with Isomerized Asp7 [J]. Molecular Biology, 2017,51(1): 143-147.

[244] Mrozek S, Jabaudon M, Jaber S, et al. Elevated Plasma Levels of sRAGE Are Associated With Nonfocal CT-Based Lung Imaging in Patients With ARDS A Prospective Multicenter Study [J]. Chest, 2016,150(5): 998-1007.

[245] Muller RB, Ostrowski SR, Haase N, et al. Markers of endothelial damage and coagulation impairment in patients with severe sepsis resuscitated with hydroxyethyl starch 130/0.42 vs Ringer acetate [J]. Journal of Critical Care, 2016,32: 16-20.

[246] Nagaraju GP, Long TE, Park W, et al. Heat Shock Protein 90 Promotes Epithelial to Mesenchymal Transition, Invasion, and Migration in Colorectal Cancer [J]. Molecular Carcinogenesis, 2015,54(10): 1147-1158.

[247] Naidu SD, Dinkova-Kostova AT. Regulation of the mammalian heat shock factor 1 [J]. Febs Journal, 2017,284(11): 1606-1627.

[248] Nair MM, Zhao RZ, Xie XP, et al. Impact of glycated LDL on endothelial nitric oxide synthase in vascular endothelial cells: involvement of transmembrane signaling and endoplasmic reticulum stress [J]. Journal of Diabetes and Its Complications, 2016,30(3): 391-397.

[249] Nargesi AA, Shalchi M, Nargesi RA, et al. The lost correlation between heat shock protein 70 (HSPA1A) and plasminogen activator inhibitor-1 in patients with type 2 diabetes and albuminuria [J]. Cell Stress & Chaperones, 2016,21(2): 361-365.

[250] Nedredal GI, Elvevold K, Chedid MF, et al. Pulmonary vascular clearance of harmful endogenous macromolecules in a porcine model of acute liver failure [J]. Annals of Hepatology, 2016,15(3): 427-435.

[251] Ni WT, Han YL, Zhao J, et al. Serum soluble urokinase-type plasminogen activator receptor as a biological marker of bacterial infection in adults: a systematic review and meta-analysis [J]. Scientific Reports, 2016,6.

[252] Nwugo CC, Doud MS, Duan YP, et al. Proteomics analysis reveals novel host molecular mechanisms associated with thermotherapy of 'Ca. Liberibacter asiaticus'-infected citrus plants [J]. Bmc Plant Biology, 2016,16.

[253] Obi AT, Andraska E, Kanthi Y, et al. Endotoxaemia-augmented murine venous thrombosis is dependent on TLR-4 and ICAM-1, and potentiated by neutropenia [J]. Thrombosis and Haemostasis, 2017,117(2): 339-348.

[254] Ohkouchi C, Kumamoto K, Saito M, et al. ING2, a tumor associated gene, enhances PAI-1 and HSPA1A expression with HDAC1 and mSin3A through the PHD domain and C-terminal [J]. Molecular Medicine Reports, 2017,16(5): 7367-7374.

[255] Okulu E, Arsan S, Akin IM, et al. Serum Levels of Soluble Urokinase Plasminogen Activator Receptor in Infants with Late-onset Sepsis [J]. Journal of Clinical Laboratory Analysis, 2015,29(5): 347-352.

[256] Ong CT, Sung SF, Wong YS, et al. Risk Factors for In-Hospital Mortality among Ischemic Stroke Patients in Southern Taiwan [J]. International Journal of Gerontology, 2016,10(2): 86-90.

[257] O'Reilly MA, Hynynen K. Emerging non-cancer applications of therapeutic ultrasound [J]. International Journal of Hyperthermia, 2015,31(3): 310-318.

[258] Ostrowski SR, Haase N, Muller RB, et al. Association between biomarkers of endothelial injury and hypocoagulability in patients with severe sepsis: a prospective study [J]. Critical Care, 2015,19.

[259] Ott HJ, Assefa D, Michaelis G. Fibrinolytic therapy as ultima ratio in disseminated intravascular coagulation (DIC) [J]. Anasthesiologie & Intensivmedizin, 2015,56: 237-241.

[260] Overstreet JM, Samarakoon R, Cardona-Grau D, et al. Tumor suppressor ataxia telangiectasia mutated functions downstream of TGF-beta 1 in orchestrating profibrotic responses [J]. Faseb Journal, 2015,29(4): 1258-1268.

[261] Ozaki N, Nellis WJ, Mashimo T, et al. Dynamic compression of dense oxide (Gd3Ga5O12) from 0.4 to 2.6 TPa: Universal Hugoniot of fluid metals [J]. Scientific Reports, 2016,6.

[262] Ozmen I, Karakurt Z, Salturk C, et al. Can N-terminal pro B-type natriuretic peptide, neutrophil-to-lymphocyte ratio, C-reactive protein help to predict short and long term mortality? [J]. Bratislava Medical Journal-Bratislavske Lekarske Listy, 2016,117(10): 587-594.

[263] Ozmerdiven G, Vuruskan B, Kaygisiz O, et al. Protective effects of diltiazem and tadalafil on shock wave-induced kidney injury in rats [J]. Bratislava Medical Journal-Bratislavske Lekarske Listy, 2017,118(4): 228-232.

[264] Palazzo P, Brooks A, James D, et al. Risk of pneumonia associated with zero-degree head positioning in acute ischemic stroke patients treated with intravenous tissue plasminogen activator [J]. Brain and Behavior, 2016,6(2).

[265] Panigada M, Zacchetti L, L'Acqua C, et al. Assessment of Fibrinolysis in Sepsis Patients with Urokinase Modified Thromboelastography [J]. Plos One, 2015,10(8).

[266] Park CK, Oh HJ, Choi HY, et al. Microbiological Characteristics and Predictive Factors for Mortality in Pleural Infection: A Single-Center Cohort Study in Korea [J]. Plos One, 2016,11(8).

[267] Park EJ, Kim YM, Kim HJ, et al. (S)YS-51, a novel isoquinoline alkaloid, attenuates obesity-associated non-alcoholic fatty liver disease in mice by suppressing lipogenesis, inflammation and coagulation [J]. European Journal of Pharmacology, 2016,788: 200-209.

[268] Park S, Hwang IW, Kim JS, et al. The effects of nonyl phenoxypolyethoxyl ethanol on cell damage pathway gene expression in SK-N-SH cells [J]. Korean Journal of Internal Medicine, 2015,30(6): 873-883.

[269] Patrani M, Tsaganos T, Kotzampassi K, et al. Soluble urokinase plasminogen activator receptor informs on the progression course after multiple injuries [J]. Biomarkers, 2016,21(7): 660-664.

[270] Peetermans M, Vanassche T, Liesenborghs L, et al. Bacterial pathogens activate plasminogen to breach tissue barriers and escape from innate immunity [J]. Critical Reviews in Microbiology, 2016,42(6): 866-882.

[271] Peiris P, Pai SL, Aniskevich S, et al. Intracardiac thrombosis during liver transplant: A 17-year single-institution study [J]. Liver Transplantation, 2015,21(10): 1280-1285.

[272] Perconti G, Maranto C, Romancino DP, et al. Pro-invasive stimuli and the interacting protein Hsp70 favour the route of alpha-enolase to the cell surface [J]. Scientific Reports, 2017,7.

[273] Perez-Fentes D. Complications of double j catheters and their endourological management [J]. Archivos Espanoles De Urologia, 2016,69(8): 527-543.

[274] Pfeiler S, Stark K, Massberg S, et al. Propagation of thrombosis by neutrophils and extracellular nucleosome networks [J]. Haematologica, 2017,102(2): 206-213.

[275] Philip F, Sahu S, Golebiewska U, et al. RNA-induced silencing attenuates G protein-mediated calcium signals [J]. Faseb Journal, 2016,30(5): 1958-1967.

[276] Pollert L, Prikrylova Z, Berousek J, et al. Direct thrombolysis of multiple thrombi in both right and left heart atrium in a patient on extracorporeal membrane oxygenation support following urgent double-lung transplantation: a case report [J]. Therapeutics and Clinical Risk Management, 2016,12: 1003-1008.

[277] Poole L, Massey V, Torres-Gonzalez E, et al. Plasminogen Activator Inhibitor-1 (pai-1) Plays A Critical Role In Alcohol-Enhanced Acute Lung Injury [J]. American Journal of Respiratory and Critical Care Medicine, 2016,193.

[278] Poole LG, Massey VL, Siow DL, et al. Plasminogen Activator Inhibitor-1 Is Critical in Alcohol-Enhanced Acute Lung Injury in Mice [J]. American Journal of Respiratory Cell and Molecular Biology, 2017,57(3): 315-323.

[279] Popowicz N, Thomas R, Lee YCG. Reply: "Less Is More" Approach for Management of Intrapleural Sepsis [J]. Annals of the American Thoracic Society, 2017,14(8): 1356-1357.

[280] Prakash S, Verghese S, Roxby D, et al. Changes in fibrinolysis and severity of organ failure in sepsis: A prospective observational study using point-of-care test-ROTEM [J]. Journal of Critical Care, 2015,30(2): 264-270.

[281] Puthusseri B, Marudamuthu A, Tiwari N, et al. Regulation of p53-mediated changes in the uPA-fibrinolytic system and in lung injury by loss of surfactant protein C expression in alveolar epithelial cells [J]. American Journal of Physiology-Lung Cellular and Molecular Physiology, 2017,312(6): L783-L796.

[282] Puthusseri B, Marudamuthu A, Twari N, et al. Regulation Of P53-Mediated Changes In The Upa-Fibrinolytic System And In Lung Injury By Loss Of Surfactant Protein-C Expression In Alveolar Epithelial Cells [J]. American Journal of Respiratory and Critical Care Medicine, 2017,195.

[283] Qian C, Li PC, Jiao Y, et al. Precise Characterization of the Penumbra Revealed by MRI: A Modified Photothrombotic Stroke Model Study [J]. Plos One, 2016,11(4).

[284] Rao ABP, Uma A, Chiranjeevi T, et al. The in vitro antitumor activity of oligonuclear polypyridyl rhodium and iridium complexes against cancer cells and human pathogens [J]. Journal of Organometallic Chemistry, 2016,824: 131-139.

[285] Redden MD, Chin TY, van Driel ML. Surgical versus non-surgical management for pleural empyema [J]. Cochrane Database of Systematic Reviews, 2017, (3).

[286] Remington TP, Remington BA, Hahn EN, et al. Deformation and failure in extreme regimes by high-energy pulsed lasers: A review [J]. Materials Science and Engineering a-Structural Materials Properties Microstructure and Processing, 2017,688: 429-458.

[287] Richards MK, McAteer JP, Edwards TC, et al. Establishing Equipoise: National Survey of the Treatment of Pediatric Para-Pneumonic Effusion and Empyema [J]. Surgical Infections, 2017,18(2): 137-142.

[288] Richardson PG, Krishnan A, Giralt S, et al. Defibrotide for the treatment of severe hepatic veno-occlusive disease/sinusoidal obstruction syndrome: evidence for clinical benefit [J]. Expert Opinion on Orphan Drugs, 2015,3(12): 1491-1501.

[289] Roesslein M, Froehlich C, Miltenberger V, et al. Thiopental protects human neuroblastoma cells from apoptotic cell death - Potential role of heat shock protein 70 [J]. Life Sciences, 2015,139: 40-45.

[290] Root S, Shulenburger L, Lemke RW, et al. Shock Response and Phase Transitions of MgO at Planetary Impact Conditions [J]. Physical Review Letters, 2015,115(19).

[291] Roule V, Ardouin P, Blanchart K, et al. Prehospital fibrinolysis versus primary percutaneous coronary intervention in ST-elevation myocardial infarction: a systematic review and meta-analysis of randomized controlled trials [J]. Critical Care, 2016,20.

[292] Rox K, Jansen R, Loof TG, et al. Linoleic and palmitoleic acid block streptokinase-mediated plasminogen activation and reduce severity of invasive group A streptococcal infection [J]. Scientific Reports, 2017,7.

[293] Roy M, Burggraf M, Lendemans S, et al. Tranexamic acid prolongs survival after controlled hemorrhage in rats [J]. Journal of Surgical Research, 2017,208: 104-110.

[294] Rundgren M, Lyngbaek S, Fisker H, et al. The Inflammatory Marker suPAR After Cardiac Arrest [J]. Therapeutic Hypothermia and Temperature Management, 2015,5(2): 89-94.

[295] Russo JJ, Goodman SG, Cantor WJ, et al. Efficacy and Safety of a Routine Early Invasive Strategy in Relation to Time from Symptom Onset to Fibrinolysis (a Subgroup Analysis of TRANSFER-AMI) [J]. American Journal of Cardiology, 2015,115(8): 1005-1012.

[296] Rybarczyk P, Vanlaeys A, Brassart B, et al. The Transient Receptor Potential Melastatin 7 Channel Regulates Pancreatic Cancer Cell Invasion through the Hsp90 alpha/uPA/MMP2 pathway [J]. Neoplasia, 2017,19(4): 288-300.

[297] Sadeghzadeh S, Liu L. Resistance and rupture analysis of single- and few-layer graphene nanosheets impacted by various projectiles [J]. Superlattices and Microstructures, 2016,97: 617-629.

[298] Sallustio F, Koch G, Motta C, et al. Efficacy and Safety of Mechanical Thrombectomy in Older Adults with Acute Ischemic Stoke [J]. Journal of the American Geriatrics Society, 2017,65(8): 1816-1820.

[299] Salzler GG, Graham A, Connolly PH, et al. Safety and Effectiveness of Adjunctive Intra-Arterial Abciximab in the Management of Acute Limb Ischemia [J]. Annals of Vascular Surgery, 2016,30: 66-71.

[300] Sangalli F, Greco G, Galbiati L, et al. Regional Thrombolysis with Tenecteplase During Extracorporeal Membrane Oxygenation: A New Approach for Left Ventricular Thrombosis [J]. Journal of Cardiac Surgery, 2015,30(6): 541-543.

[301] Sapru A, Flori H, Quasney MW, et al. Pathobiology of Acute Respiratory Distress Syndrome [J]. Pediatric Critical Care Medicine, 2015,16(5): S6-S22.

[302] Sase T, Arito M, Onodera H, et al. Effects of edaravone on hypoxic human astrocytes revealed by a proteomic approach [J]. Biomedical Research-India, 2016,27(4): 1064-1070.

[303] Schuliga M, Jaffar J, Berhan A, et al. Annexin A2 contributes to lung injury and fibrosis by augmenting factor Xa fibrogenic activity [J]. American Journal of Physiology-Lung Cellular and Molecular Physiology, 2017,312(5): 1772-1782.

[304] Segerer FJ, Seeger K, Maier A, et al. Therapy of 645 children with parapneumonic effusion and empyemaA German nationwide surveillance study [J]. Pediatric Pulmonology, 2017,52(4): 540-547.

[305] Semeraro N, Ammollo CT, Semeraro F, et al. Coagulopathy of Acute Sepsis [J]. Seminars in Thrombosis and Hemostasis, 2015,41(6): 650-658.

[306] Shan L, Shan F, Li J, et al. Association of circulating suPAR with disease severity and clinical outcomes in patients with ARDS induced by intra-abdominal infections: a prospective observational study [J]. International Journal of Clinical and Experimental Medicine, 2016,9(7): 12788-12795.

[307] Shao LD, Su J, Ye BX, et al. Design, Synthesis, and Biological Activities of Vibsanin B Derivatives: A New Class of HSP90 C-Terminal Inhibitors [J]. Journal of Medicinal Chemistry, 2017,60(21): 9053-9066.

[308] Shetty SK, Tiwari N, Marudamuthu AS, et al. p53 and miR-34a Feedback Promotes Lung Epithelial Injury and Pulmonary Fibrosis [J]. American Journal of Pathology, 2017,187(5): 1016-1034.

[309] Shi CF, Sui ZF, Li L, et al. No Association of SERPINE1-675 Polymorphism With Sepsis Susceptibility A Meta-Analysis [J]. Medicine, 2015,94(45).

[310] Shi JH, Cui NP, Wang S, et al. Overexpression of YB1 C-terminal domain inhibits proliferation, angiogenesis and tumorigenicity in a SK-BR-3 breast cancer xenograft mouse model [J]. Febs Open Bio, 2016,6: 33-42.

[311] Shi QK, Mu XW, Hong L, et al. SERPINE1 rs1799768 polymorphism contributes to sepsis risk and mortality [J]. Journal of the Renin-Angiotensin-Aldosterone System, 2015,16(4): 1218-1224.

[312] Siemens N, Kittang BR, Chakrakodi B, et al. Increased cytotoxicity and streptolysin O activity in group G streptococcal strains causing invasive tissue infections [J]. Scientific Reports, 2015,5.

[313] Sikri G, Bhattachar S. Comment on "Soluble Urokinase-Type Plasminogen Activator Receptor Plasma Concentration May Predict Susceptibility to High Altitude Pulmonary Edema" [J]. Mediators of Inflammation, 2017.

[314] Simmons JW, Powell MF. Acute traumatic coagulopathy: pathophysiology and resuscitation [J]. British Journal of Anaesthesia, 2016,117: 31-43.

[315] Singh N, Raghav M, Narula S, et al. Profiling of Virulence Determinants in Cronobacter sakazakii Isolates from Different Plant and Environmental Commodities [J]. Current Microbiology, 2017,74(5): 560-565.

[316] Sippel TR, Shimizu T, Strnad F, et al. Arginase I release from activated neutrophils induces peripheral immunosuppression in a murine model of stroke [J]. Journal of Cerebral Blood Flow and Metabolism, 2015,35(10): 1657-1663.

[317] Smeds MR, Sandhu HK, Leake SS, et al. Patterns in the Management of Acute Limb Ischemia: A VESS Survey [J]. Annals of Vascular Surgery, 2017,38: 164-171.

[318] Sondeen JL, Hanson MA, Prince MD, et al. Double-blinded, placebo-controlled study of early tranexamic acid treatment in swine uncontrolled hemorrhage model [J]. Journal of Trauma and Acute Care Surgery, 2016,80(1): 81-88.

[319] Soto-Rivera CL, Agus MSD, Sawyer JE, et al. Pediatric Cardiac Intensive Care Society 2014 Consensus Statement: Pharmacotherapies in Cardiac Critical Care Hormone Replacement Therapy [J]. Pediatric Critical Care Medicine, 2016,17(3): S59-S68.

[320] Starr ME, Steele AM, Cohen DA, et al. Short-Term Dietary Restriction Rescues Mice From Lethal Abdominal Sepsis and Endotoxemia and Reduces the Inflammatory/Coagulant Potential of Adipose Tissue [J]. Critical Care Medicine, 2016,44(7): E509-E519.

[321] Stieber B, Monecke S, Muller E, et al. Direct, Specific and Rapid Detection of Staphylococcal Proteins and Exotoxins Using a Multiplex Antibody Microarray [J]. Plos One, 2015,10(12).

[322] Stope MB, Klinkmann G, Diesing K, et al. Heat Shock Protein HSP27 Secretion by Ovarian Cancer Cells Is Linked to Intracellular Expression Levels, Occurs Independently of the Endoplasmic Reticulum Pathway and HSP27's Phosphorylation Status, and Is Mediated by Exosome Liberation [J]. Disease Markers, 2017.

[323] Stope MB, Wiegank L, Weiss M, et al. Drug-induced Modulation of Heat Shock Protein HSPB1 in an Ovarian Cancer Cell Model [J]. Anticancer Research, 2016,36(7): 3321-3327.

[324] Suhail N, Bilal N, Hasan S, et al. Chronic unpredictable stress (CUS) enhances the carcinogenic potential of 7,12-dimethylbenz(a)anthracene (DMBA) and accelerates the onset of tumor development in Swiss albino mice [J]. Cell Stress & Chaperones, 2015,20(6): 1023-1036.

[325] Sunnetcioglu A, Sunnetcioglu M, Adiyaman F, et al. Could soluble urokinase plasminogen receptor (suPAR) be used as a diagnostic biomarker for ventilator-associated pneumonia? [J]. Clinical Respiratory Journal, 2017,11(6): 925-930.

[326] Swarbreck SB, Secor D, Ellis CG, et al. Effect of ascorbate on plasminogen activator inhibitor-1 expression and release from platelets and endothelial cells in an in-vitro model of sepsis [J]. Blood Coagulation & Fibrinolysis, 2015,26(4): 436-442.

[327] Taghavi N, Yazdi I. Prognostic Factors of Survival Rate in Oral Squamous Cell Carcinoma: Clinical, Histologic, Genetic and Molecular Concepts [J]. Archives of Iranian Medicine, 2015,18(5): 314-319.

[328] Tamburro RF, Kneyber MCJ, Pediatric Acute Lung Injury C. Pulmonary Specific Ancillary Treatment for Pediatric Acute Respiratory Distress Syndrome: Proceedings From the Pediatric Acute Lung Injury Consensus Conference [J]. Pediatric Critical Care Medicine, 2015,16(5): S61-S72.

[329] Tang W, Jing B, Huang YF, et al. Feature extraction for latent fault detection and failure modes classification of board-level package under vibration loadings [J]. Science China-Technological Sciences, 2015,58(11): 1905-1914.

[330] Tanpure S, Boyineini J, Gnanamony M, et al. SPARC overexpression suppresses radiation-induced HSP27 and induces the collapse of mitochondrial Delta psi in neuroblastoma cells [J]. Oncology Letters, 2017,13(6): 4602-4610.

[331] Tatlisu MA, Kaya A, Keskin M, et al. The association of blood urea nitrogen levels with mortality in acute pulmonary embolism [J]. Journal of Critical Care, 2017,39: 248-253.

[332] Tavenier J, Haupt TH, Andersen AL, et al. A high-protein diet during hospitalization is associated with an accelerated decrease in soluble urokinase plasminogen activator receptor levels in acutely ill elderly medical patients with SIRS [J]. Nutrition Research, 2017,41: 56-64.

[333] Tazawa H, Sato K, Tsutiya A, et al. A microfluidic cell culture system for monitoring of sequential changes in endothelial cells after heat stress [J]. Thrombosis Research, 2015,136(2): 328-334.

[334] Terkelsen T, Schmitz ML, Simonsen CZ, et al. Thrombolysis in acute ischemic stroke is associated with lower long-term hospital bed day use: A nationwide propensity score-matched follow-up study [J]. International Journal of Stroke, 2016,11(8): 910-916.

[335] Thachil J. Disseminated intravascular coagulation - new pathophysiological concepts and impact on management [J]. Expert Review of Hematology, 2016,9(8): 803-814.

[336] Thomson EM, Pal S, Guenette J, et al. Ozone Inhalation Provokes Glucocorticoid-Dependent and -Independent Effects on Inflammatory and Metabolic Pathways [J]. Toxicological Sciences, 2016,152(1): 17-28.

[337] Tian YH, Li LJ, Zhang FB, et al. Seminal plasma HSPA2 mRNA content is associated with semen quality [J]. Journal of Assisted Reproduction and Genetics, 2016,33(8): 1079-1084.

[338] Timmermans K, Vaneker M, Scheffer GJ, et al. Soluble urokinase-type plasminogen activator levels are related to plasma cytokine levels but have low predictive value for mortality in trauma patients [J]. Journal of Critical Care, 2015,30(3): 476-480.

[339] Tiwari N, Marudamuthu AS, Tsukasaki Y, et al. p53-and PAI-1-mediated induction of C-X-C chemokines and CXCR2: importance in pulmonary inflammation due to cigarette smoke exposure [J]. American Journal of Physiology-Lung Cellular and Molecular Physiology, 2016,310(6): L496-L506.

[340] Toh CH, Alhamdi Y, Abrams ST. Current Pathological and Laboratory Considerations in the Diagnosis of Disseminated Intravascular Coagulation [J]. Annals of Laboratory Medicine, 2016,36(6): 505-512.

[341] Tomlinson JE, Byrne E, Pusterla N, et al. The Use of Recombinant Tissue Plasminogen Activator (rTPA) in The Treatment of Fibrinous Pleuropneumonia in Horses: 25 Cases (2007-2012) [J]. Journal of Veterinary Internal Medicine, 2015,29(5): 1403-1409.

[342] Tsirigotis P, Chondropoulos S, Frantzeskaki F, et al. Thrombocytopenia in critically ill patients with severe sepsis/septic shock: Prognostic value and association with a distinct serum cytokine profile [J]. Journal of Critical Care, 2016,32: 9-15.

[343] Tsuji M. Cold-stress responses in the Antarctic basidiomycetous yeast Mrakia blollopis [J]. Royal Society Open Science, 2016,3(7).

[344] Tversky S, Libman RB, Schloss ER, et al. Catastrophic Intracranial Hemorrhages after IV tPA in a Patient with Insidious Onset of Fever and Back Pain [J]. Journal of Stroke & Cerebrovascular Diseases, 2016,25(5): E69-E70.

[345] Tziolos N, Kotanidou A, Orfanos SE. Biomarkers in infection and sepsis: Can they really indicate final outcome? [J]. International Journal of Antimicrobial Agents, 2015,46: S29-S32.

[346] Umemura Y, Yamakawa K, Kiguchi T, et al. Design and Evaluation of New Unified Criteria for Disseminated Intravascular Coagulation Based on the Japanese Association for Acute Medicine Criteria [J]. Clinical and Applied Thrombosis-Hemostasis, 2016,22(2): 153-160.

[347] van Zyl N, Milford EM, Diab S, et al. Activation of the protein C pathway and endothelial glycocalyx shedding is associated with coagulopathy in an ovine model of trauma and hemorrhage [J]. Journal of Trauma and Acute Care Surgery, 2016,81(4): 674-684.

[348] Velineni S, DeNegri R, Artiushin SC, et al. Comparison of specificities of serum antibody responses of horses to clinical infections caused by Streptococcus equi or zooepidemicus [J]. Veterinary Microbiology, 2015,180(3-4): 253-259.

[349] Vercaigne LM, Allan DR, Armstrong SW, et al. An ethanol/sodium citrate locking solution compared to heparin to prevent hemodialysis catheter-related infections: a randomized pilot study [J]. Journal of Vascular Access, 2016,17(1): 55-62.

[350] Viergutz T, Gruttner J, Walter T, et al. Preclinical fibrinolysis in patients with ST-segment elevation myocardial infarction in a rural region [J]. Anaesthesist, 2016,65(9): 673-680.

[351] Villarini M, Gambelunghe A, Giustarini D, et al. No evidence of DNA damage by co-exposure to extremely low frequency magnetic fields and aluminum on neuroblastoma cell lines [J]. Mutation Research-Genetic Toxicology and Environmental Mutagenesis, 2017,823: 11-21.

[352] Vogelberg C. Diagnostics and therapy of pleural empyema [J]. Monatsschrift Kinderheilkunde, 2015,163(11): 1179-1188.

[353] Vora AN, Peterson ED, McCoy LA, et al. Factors Associated With Initial Prasugrel Versus Clopidogrel Selection for Patients With Acute Myocardial Infarction Undergoing Percutaneous Coronary Intervention: Insights From the Treatment With ADP Receptor Inhibitors: Longitudinal Assessment of Treatment Patterns and Events After Acute Coronary Syndrome (TRANSLATE-ACS) Study [J]. Journal of the American Heart Association, 2016,5(9).

[354] Vorster MJ, Koegelenberg CFN. Breaking Down the Barriers in Complicated Pleural Sepsis [J]. Respiration, 2016,91(2): 99-100.

[355] Wang GD, Kong FY, Zhang S, et al. A tomato chloroplast-targeted DnaJ protein protects Rubisco activity under heat stress [J]. Journal of Experimental Botany, 2015,66(11): 3027-3040.

[356] Wang J, Coppari F, Smith RF, et al. X-ray diffraction of molybdenum under ramp compression to 1 TPa [J]. Physical Review B, 2016,94(10).

[357] Wang Q, Wang YS, Zhang Y, et al. Involvement of urokinase in cigarette smoke extract-induced epithelial-mesenchymal transition in human small airway epithelial cells [J]. Laboratory Investigation, 2015,95(5): 469-479.

[358] Wang RC, Huang CY, Pan TL, et al. Proteomic Characterization of Annexin 1 (ANX1) and Heat Shock Protein 27 (HSP27) as Biomarkers for Invasive Hepatocellular Carcinoma Cells [J]. Plos One, 2015,10(10).

[359] Wang Y, Ivany JN, Perkovic V, et al. Anticoagulants and antiplatelet agents for preventing central venous haemodialysis catheter malfunction in patients with end-stage kidney disease [J]. Cochrane Database of Systematic Reviews, 2016, (4).

[360] Wang YM, Zhao Z, Rege SV, et al. 3K3A-activated protein C stimulates postischemic neuronal repair by human neural stem cells in mice [J]. Nature Medicine, 2016,22(9): 1050-+.

[361] Wegmann C, Pfister R, Scholz S, et al. Diagnostic value of left bundle branch block in patients with acute myocardial infarction. A prospective analysis [J]. Herz, 2015,40(8): 1107-1114.

[362] Weijer R, Clavier S, Zaal EA, et al. Multi-OMIC profiling of survival and metabolic signaling networks in cells subjected to photodynamic therapy [J]. Cellular and Molecular Life Sciences, 2017,74(6): 1133-1151.

[363] Wheaton AK, Velikoff M, Agarwal M, et al. The vitronectin RGD motif regulates TGF-beta-induced alveolar epithelial cell apoptosis [J]. American Journal of Physiology-Lung Cellular and Molecular Physiology, 2016,310(11): L1206-L1217.

[364] White CW, Rancourt RC, Veress LA. Sulfur mustard inhalation: mechanisms of injury, alteration of coagulation, and fibrinolytic therapy[A]. In: Countermeasures against Chemical Threats Ii (Laskin JD, Braaten D, eds), Vol. 1378, 2016: 87-95.

[365] Wiener G, Moore HB, Moore EE, et al. Shock releases bile acidinducing platelet inhibition and fibrinolysis [J]. Journal of Surgical Research, 2015,195(2): 390-395.

[366] Wilkes EJA, Hughes KJ, Kessell AE, et al. Successful management of multiple extrapulmonary complications associated with Rhodococcus equi pneumonia in a foal [J]. Equine Veterinary Education, 2016,28(4): 186-192.

[367] Wilson JAS, Mossop P, Soroka SD, et al. Comparison of intensive versus standard hemodialysis central venous catheter dysfunction protocol using rt-PA: a quality assurance initiative [J]. Journal of Vascular Access, 2016,17(2): 143-150.

[368] Woloszynska I, Stepien A. RISK FACTORS OF rt-PA THERAPY IN PATIENTS WITH ISCHEMIC STROKE [J]. Acta Poloniae Pharmaceutica, 2017,74(1): 293-298.

[369] Wrotek A, Jackowska T. The role of the soluble urokinase plasminogen activator (suPAR) in children with pneumonia [J]. Respiratory Physiology & Neurobiology, 2015,209: 120-123.

[370] Wu CZ, Chang LC, Lin YF, et al. Urokinase plasminogen activator receptor and its soluble form in common biopsy-proven kidney diseases and in staging of diabetic nephropathy [J]. Clinical Biochemistry, 2015,48(18): 1324-1329.

[371] Wu J, Zhao J, Yu J, et al. Cylindromatosis (CYLD) inhibits Streptococcus pneumonia-induced plasminogen activator inhibitor-1 expression via interacting with TRAF-6 [J]. Biochemical and Biophysical Research Communications, 2015,463(4): 942-947.

[372] Wu XW, Dubick MA, Schwacha MG, et al. TRANEXAMIC ACID ATTENUATES THE LOSS OF LUNG BARRIER FUNCTION IN A RAT MODEL OF POLYTRAUMA AND HEMORRHAGE WITH RESUSCITATION [J]. Shock, 2017,47(4): 500-505.

[373] Wujak L, Markart P, Wygrecka M. The low density lipoprotein receptor-related protein (LRP) 1 and its function in lung diseases [J]. Histology and Histopathology, 2016,31(7): 733-745.

[374] Xiao YZ, Luo H, Zhou B, et al. Comparison of soluble urokinase plasminogen activator receptor, soluble triggering receptor expressed on myeloid cells 1, procalcitonin and C-reactive protein in distinguishing concurrent bacterial infection from idiopathic inflammatory myopathy [J]. Rheumatology International, 2017,37(4): 585-592.

[375] Xie HR, Hu H, Chang M, et al. Identification of chaperones in a MPP+-induced and ATRA/TPA-differentiated SH-SY5Y cell PD model [J]. American Journal of Translational Research, 2016,8(12): 5659-5671.

[376] Xu W, Cai SY, Zhang Y, et al. Melatonin enhances thermotolerance by promoting cellular protein protection in tomato plants [J]. Journal of Pineal Research, 2016,61(4): 457-469.

[377] Xu X, Wang HC, Li HJ, et al. SERPINE I-844 and-675 polymorphisms and chronic obstructive pulmonary disease in a Chinese Han population [J]. Journal of International Medical Research, 2016,44(6): 1292-1301.

[378] Xu XL, Wu Y, Hu MY, et al. Structure-activity relationship of Garcinia xanthones analogues: Potent Hsp90 inhibitors with cytotmdcity and antiangiogenesis activity [J]. Bioorganic & Medicinal Chemistry, 2016,24(19): 4626-4635.

[379] Yan YS, Du SS, Ji Y, et al. Discovery of enzymatically depolymerized heparins capable of treating Bleomycin-induced pulmonary injury and fibrosis in mice [J]. Carbohydrate Polymers, 2017,174: 82-88.

[380] Yang J, Zhao LX, Li CS, et al. Variations of Postresuscitation Lung Function after Thrombolysis Therapy in a Cardiac Arrest Porcine Model Caused by Pulmonary Thromboembolism [J]. Chinese Medical Journal, 2017,130(12): 1475-1480.

[381] Yang MY, Guan X, Wan Z, et al. Prehospital facilitated intervention as a potential "sally port" for the rescue of acute ST-segment elevation myocardial infarction: a meta-analysis [J]. International Journal of Clinical and Experimental Medicine, 2016,9(11): 20761-20776.

[382] Yang W, Cui M, Lee JC, et al. Heat shock protein inhibitor, quercetin, as a novel adjuvant agent to improve radiofrequency ablation-induced tumor destruction and its molecular mechanism [J]. Chinese Journal of Cancer Research, 2016,28(1): 19-28.

[383] Yanilkin AV. Investigation of alpha-phase and liquid uranium by the method of quantum molecular dynamics [J]. High Temperature, 2017,55(1): 40-46.

[384] Yu L, Da XW, Wu XL, et al. Simvastatin prevents lipopolysaccharide-induced septic shock in rats [J]. Journal of Huazhong University of Science and Technology-Medical Sciences, 2017,37(2): 226-230.

[385] Yuan KF, Chen M, He GF, et al. Comparison of anticoagulation and thrombolysis therapies in lipopolysaccharide-induced disseminated intravascular coagulation [J]. International Journal of Clinical and Experimental Medicine, 2017,10(8): 11796-11803.

[386] Yue L, Xiang JY, Sun P, et al. Relationship Between HSP70 and ERBB2 Expression in Breast Cancer Cell Lines Regarding Drug Resistance [J]. Anticancer Research, 2016,36(3): 1243-1249.

[387] Yurinskaya MM, Funikov SY, Evgen'ev MB, et al. Exogenous heat shock protein HSP70 reduces response of human neuroblastoma cells to lipopolysaccharide [J]. Doklady Biochemistry and Biophysics, 2016,469(1): 239-243.

[388] Yurinskaya MM, Mit'kevich VA, Barykin EP, et al. Heat-shock protein HSP70 protects neuroblastoma cells SK-N-SH from the neurotoxic effects of hydrogen peroxide and the beta-amyloid peptide [J]. Molecular Biology, 2015,49(6): 924-927.

[389] Yurinskaya MM, Mit'kevich VA, Evgen'ev MB, et al. Heat-shock protein HSP70 reduces the secretion of TNF alpha by neuroblastoma cells and human monocytes induced with beta-amyloid peptides [J]. Molecular Biology, 2016,50(6): 930-932.

[390] Zampoli M, Kappos A, Verwey C, et al. Impact of fibrinolytics on the outcome of empyema in South African children [J]. Samj South African Medical Journal, 2015,105(7): 549-553.

[391] Zeng M, Chang MC, Zheng HC, et al. Clinical value of soluble urokinase-type plasminogen activator receptor in the diagnosis, prognosis, and therapeutic guidance of sepsis [J]. American Journal of Emergency Medicine, 2016,34(3): 375-380.

[392] Zeng YQ, Cao RY, Zhang TH, et al. Design and synthesis of piperidine derivatives as novel human heat shock protein 70 inhibitors for the treatment of drug-resistant tumors [J]. European Journal of Medicinal Chemistry, 2015,97: 19-31.

[393] Zhaleh M, Azadbakht M, Pour AB. Inhibitory effects of mouse bone marrow mesenchymal stem cell soup on staurospurine-induced cell death in MCF-7 and AGS [J]. Bratislava Medical Journal-Bratislavske Lekarske Listy, 2017,118(1): 34-43.

[394] Zhang F, Luo J, Min S, et al. Propofol alleviates electroconvulsive shock-induced memory impairment by modulating proBDNF/mBDNF ratio in depressive rats [J]. Brain Research, 2016,1642: 43-50.

[395] Zhang H, Song Y, Li ZY, et al. Evaluation of breviscapine on prevention of experimentally induced abdominal adhesions in rats [J]. American Journal of Surgery, 2016,211(6): 1143-1152.

[396] Zhao Q, Nie JX, Zhang W, et al. Effect of the Al/O ratio on the Al reaction of aluminized RDX-based explosives [J]. Chinese Physics B, 2017,26(5).

[397] Zhao RZ, Le K, Moghadasian MH, et al. Reduced monocyte adhesion to aortae of diabetic plasminogen activator inhibitor-1 knockout mice [J]. Inflammation Research, 2017,66(9): 783-792.

[398] Zhao RZ, Xie XP, Le K, et al. Endoplasmic reticulum stress in diabetic mouse or glycated LDL-treated endothelial cells: protective effect of Saskatoon berry powder and cyanidin glycans [J]. Journal of Nutritional Biochemistry, 2015,26(11): 1248-1253.

[399] Zhao ZL, Jinde S, Kakiuchi C, et al. Extracellular elevation of adrenomedullin, a gene associated with schizophrenia, suppresses heat shock protein 1A/1B mRNA [J]. Neuroreport, 2016,27(18): 1312-1316.

[400] Zhu B, Xu TT, Lin ZF, et al. Recombinant heat shock protein 78 enhances enterovirus 71 propagation in Vero cells and is induced in SK-N-SH cells during the infection [J]. Archives of Virology, 2017,162(6): 1649-1660.

[401] Zimbler DL, Schroeder JA, Eddy JL, et al. Early emergence of Yersinia pestis as a severe respiratory pathogen [J]. Nature Communications, 2015,6.

[402] Zipperle J, Altenburger K, Ponschab M, et al. Potential role of platelet-leukocyte aggregation in trauma-induced coagulopathy: Ex vivo findings [J]. Journal of Trauma and Acute Care Surgery, 2017,82(5): 921-926.

[403] Zivkovic AR, Sedlaczek O, von Haken R, et al. Muscarinic M1 receptors modulate endotoxemia-induced loss of synaptic plasticity [J]. Acta Neuropathologica Communications, 2015,3.

[404] Zolakova B, Zolak V, Hatok J, et al. Soluble receptor for advanced glycation end products in late onset neonatal infection [J]. Bratislava Medical Journal-Bratislavske Lekarske Listy, 2016,117(1): 15-18.

[405] Zolali E, Asgharian P, Hamishehkar H, et al. Effects of gamma oryzanol on factors of oxidative stress and sepsis-induced lung injury in experimental animal model [J]. Iranian Journal of Basic Medical Sciences, 2015,18(12): 1257-1263.

[406] Zuchtriegel G, Uhl B, Puhr-Westerheide D, et al. Platelets Guide Leukocytes to Their Sites of Extravasation [J]. Plos Biology, 2016,14(5).

[407] Zwiech R, Adelt M, Chrul S. A Taurolidine-Citrate-Heparin Lock Solution Effectively Eradicates Pathogens From the Catheter Biofilm in Hemodialysis Patients [J]. American Journal of Therapeutics, 2016,23(2): E363-E368.

[408] Abitagaoglu S, Akinci SB, Saricaoglu F, et al. Effect of coenzyme Q10 on organ damage in sepsis [J]. Bratislava Medical Journal-Bratislavske Lekarske Listy, 2015,116(7): 433-439.

[409] Abrosimov SA, Bazhulin AP, Bolshakov AP, et al. Strength of synthetic diamonds under tensile stresses produced by picosecond laser action [J]. Journal of Applied Mechanics and Technical Physics, 2015,56(1): 143-149.

[410] Ali SK, Reveles KR, Davis R, et al. The Association of Statin Use and Gonado-Sexual Function in Women: A Retrospective Cohort Analysis [J]. Journal of Sexual Medicine, 2015,12(1): 83-92.

[411] Bersani I, Auriti C, Ronchetti MP, et al. Use of Early Biomarkers in Neonatal Brain Damage and Sepsis: State of the Art and Future Perspectives [J]. Biomed Research International, 2015.

[412] Bhandary YP, Shetty SK, Marudamuthu AS, et al. Role of p53-fibrinolytic system cross-talk in the regulation of quartz-induced lung injury [J]. Toxicology and Applied Pharmacology, 2015,283(2): 92-98.

[413] Bondu V, Schrader R, Gawinowicz MA, et al. Elevated Cytokines, Thrombin and PAI-1 in Severe HCPS Patients Due to Sin Nombre Virus [J]. Viruses-Basel, 2015,7(2): 559-589.

[414] Brunkhorst FM, Patchev V. Sepsis-associated Purpura Fulminans International Registry-Europe (SAPFIRE) [J]. Medizinische Klinik-Intensivmedizin Und Notfallmedizin, 2014,109(8): 591-595.

[415] Bucova M, Suchankova M, Tibenska E, et al. Diagnostic value of TREM-1 and TREM-2 expression in bronchoalveolar lavage fluid in sarcoidosis and other lung diseases [J]. Bratislava Medical Journal-Bratislavske Lekarske Listy, 2015,116(12): 707-713.

[416] Carreras E. How I manage sinusoidal obstruction syndrome after haematopoietic cell transplantation [J]. British Journal of Haematology, 2015,168(4): 481-491.

[417] Cartin-Ceba R, Hubmayr RD, Qin R, et al. Predictive value of plasma biomarkers for mortality and organ failure development in patients with acute respiratory distress syndrome [J]. Journal of Critical Care, 2015,30(1).

[418] Chen JJ, Chang HF, Hsu YC, et al. Anton-Babinski syndrome in an old patient: a case report and literature review [J]. Psychogeriatrics, 2015,15(1): 58-61.

[419] Chen ZX, Zhao RZ, Zhao MM, et al. Regulation of epithelial sodium channels in urokinase plasminogen activator deficiency [J]. American Journal of Physiology-Lung Cellular and Molecular Physiology, 2014,307(8): L609-L617.

[420] Chi YF, Chai JK, Yu YM, et al. Association between PAI-1 polymorphisms and plasma PAI-1 level with sepsis in severely burned patients [J]. Genetics and Molecular Research, 2015,14(3): 10081-10086.

[421] Cruz-Lagunas A, Jimenez-Alvarez L, Ramirez G, et al. Obesity and pro-inflammatory mediators are associated with acute kidney injury in patients with A/H1N1 influenza and acute respiratory distress syndrome [J]. Experimental and Molecular Pathology, 2014,97(3): 453-457.

[422] Dastgheyb S, Parvizi J, Shapiro IM, et al. Effect of Biofilms on Recalcitrance of Staphylococcal Joint Infection to Antibiotic Treatment [J]. Journal of Infectious Diseases, 2015,211(4): 641-650.

[423] Davis R, Reveles KR, Ali SK, et al. Statins and Male Sexual Health: A Retrospective Cohort Analysis [J]. Journal of Sexual Medicine, 2015,12(1): 158-167.

[424] Dinarvand P, Hassanian SM, Weiler H, et al. Intraperitoneal administration of activated protein C prevents postsurgical adhesion band formation [J]. Blood, 2015,125(8): 1339-1348.

[425] Djuric M, Djuric D, Culibrk T, et al. Parapneumonic Effusions: Features, Diagnostics and Treatment Options [J]. Srpski Arhiv Za Celokupno Lekarstvo, 2014,142(11-12): 680-687.

[426] Donaldson JF, Tait C, Rad M, et al. Obstructive Uropathy and Vesicovaginal Fistula Secondary to a Retained Sex Toy in the Vagina [J]. Journal of Sexual Medicine, 2014,11(10): 2595-2600.

[427] Engelberger RP, Moschovitis A, Fahrni J, et al. Fixed low-dose ultrasound-assisted catheter-directed thrombolysis for intermediate and high-risk pulmonary embolism [J]. European Heart Journal, 2015,36(10): 597-604.

[428] Francois D, Venisse L, Marchal-Somme J, et al. Increased expression of protease nexin-1 in fibroblasts during idiopathic pulmonary fibrosis regulates thrombin activity and fibronectin expression [J]. Laboratory Investigation, 2014,94(11): 1237-1246.

[429] Galliera E, Drago L, Marazzi MG, et al. Soluble urokinase-type plasminogen activator receptor (suPAR) as new biomarker of the prosthetic joint infection: Correlation with inflammatory cytokines [J]. Clinica Chimica Acta, 2015,441: 23-28.

[430] Georgescu AM, Szederjesi J, Voidazan S, et al. Soluble urokinase-type plasminogen activator receptor (suPAR) - a possible biomarker for bacteremia in sepsis [J]. Revista Romana De Medicina De Laborator, 2015,23(1): 59-73.

[431] Gerner ST, Kuramatsu JB, Abel H, et al. Intraventricular Fibrinolysis has No Effects on Shunt Dependency and Functional Outcome in Endovascular-Treated Aneurysmal SAH [J]. Neurocritical Care, 2014,21(3): 435-443.

[432] Gupta OP, Mishra V, Singh NK, et al. Deciphering the dynamics of changing proteins of tolerant and intolerant wheat seedlings subjected to heat stress [J]. Molecular Biology Reports, 2015,42(1): 43-51.

[433] Hayakawa M, Gando S, Ono Y, et al. NOBLE-COLLIP DRUM TRAUMA INDUCES DISSEMINATED INTRAVASCULAR COAGULATION BUT NOT ACUTE COAGULOPATHY OF TRAUMA-SHOCK [J]. Shock, 2015,43(3): 261-267.

[434] Hoppensteadt D, Tsuruta K, Hirman J, et al. Dysregulation of Inflammatory and Hemostatic Markers in Sepsis and Suspected Disseminated Intravascular Coagulation [J]. Clinical and Applied Thrombosis-Hemostasis, 2015,21(2): 120-127.

[435] Huang ST, Lin CL, Chang YJ, et al. Pneumococcal pneumonia infection is associated with end-stage renal disease in adult hospitalized patients [J]. Kidney International, 2014,86(5): 1023-1030.

[436] Iriz E, Iriz A, Take G, et al. Iloprost and vitamin C attenuates acute myocardial injury induced by suprarenal aortic ischemia-reperfusion in rabbits [J]. Bratislava Medical Journal-Bratislavske Lekarske Listy, 2015,116(10): 627-631.

[437] Jespersen D, Huang BR. Proteins associated with heat-induced leaf senescence in creeping bentgrass as affected by foliar application of nitrogen, cytokinins, and an ethylene inhibitor [J]. Proteomics, 2015,15(4): 798-812.

[438] Ji HL, Zhao RZ, Komissarov AA, et al. Proteolytic Regulation of Epithelial Sodium Channels by Urokinase Plasminogen Activator CUTTING EDGE AND CLEAVAGE SITES [J]. Journal of Biological Chemistry, 2015,290(9): 5241-5255.

[439] Komarova EY, Meshalkina DA, Aksenov ND, et al. The discovery of Hsp70 domain with cell-penetrating activity [J]. Cell Stress & Chaperones, 2015,20(2): 343-354.

[440] Kongbunkiat K, Kasemsap N, Travanichakul S, et al. Hospital mortality from atrial fibrillation associated with ischemic stroke: a national data report [J]. International Journal of Neuroscience, 2015,125(12): 924-928.

[441] Lee PH, Liu CM, Ho TS, et al. Enterovirus 71 Virion-Associated Galectin-1 Facilitates Viral Replication and Stability [J]. Plos One, 2015,10(2).

[442] Levi M, van der Poll T. A Short Contemporary History of Disseminated Intravascular Coagulation [J]. Seminars in Thrombosis and Hemostasis, 2014,40(8): 874-880.

[443] Levi M, van der Poll T. Coagulation in Patients with Severe Sepsis [J]. Seminars in Thrombosis and Hemostasis, 2015,41(1): 9-15.

[444] Liu YY, Li LF, Fu JY, et al. Induced Pluripotent Stem Cell Therapy Ameliorates Hyperoxia-Augmented Ventilator-Induced Lung Injury through Suppressing the Src Pathway [J]. Plos One, 2014,9(10).

[445] Lo HH, Cheng WS. Distribution of virulence factors and association with emm polymorphism or isolation site among beta-hemolytic group G Streptococcus dysgalactiae subspecies equisimilis [J]. Apmis, 2015,123(1): 45-52.

[446] Lurie F, Vaidya V, Comerota AJ. Clinical outcomes and cost-effectiveness of initial treatment strategies for nonembolic acute limb ischemia in real-life clinical settings [J]. Journal of Vascular Surgery, 2015,61(1): 138-146.

[447] Ma ST, Feng CT, Dai GL, et al. In silico target fishing for the potential bioactive components contained in Huanglian Jiedu Tang (HLJDD) and elucidating molecular mechanisms for the treatment of sepsis [J]. Chinese Journal of Natural Medicines, 2015,13(1): 30-40.

[448] Manns BJ, Scott-Douglas N, Tonelli M, et al. An Economic Evaluation of rt-PA Locking Solution in Dialysis Catheters [J]. Journal of the American Society of Nephrology, 2014,25(12): 2887-2895.

[449] Marhuenda C, Barcelo C, Fuentes I, et al. Urokinase Versus VATS for Treatment of Empyema: A Randomized Multicenter Clinical Trial [J]. Pediatrics, 2014,134(5): E1301-E1307.

[450] Marudamuthu AS, Bhandary YP, Shetty SK, et al. Role of the Urokinase-Fibrinolytic System in Epithelial-Mesenchymal Transition during Lung Injury [J]. American Journal of Pathology, 2015,185(1): 55-68.

[451] McGarrah RW, Ahmad T, Koeberl DD, et al. The Heart Is Just a Muscle [J]. Circulation, 2015,131(10): 914-922.

[452] Mearelli F, Orso D, Fiotti N, et al. Sepsis outside intensive care unit: the other side of the coin [J]. Infection, 2015,43(1): 1-11.

[453] Mohan S, Hertweck C, Dudda A, et al. Tuf of Streptococcus pneumoniae is a surface displayed human complement regulator binding protein [J]. Molecular Immunology, 2014,62(1): 249-264.

[454] Montero-Martin M, Inwald DP, Carrol ED, et al. Prognostic markers of meningococcal disease in children: recent advances and future challenges [J]. Expert Review of Anti-Infective Therapy, 2014,12(11): 1357-1369.

[455] Moore HB, Moore EE, Gonzalez E, et al. Hyperfibrinolysis, physiologic fibrinolysis, and fibrinolysis shutdown: The spectrum of postinjury fibrinolysis and relevance to antifibrinolytic therapy [J]. Journal of Trauma and Acute Care Surgery, 2014,77(6): 811-817.

[456] Moore HB, Moore EE, Gonzalez E, et al. HEMOLYSIS EXACERBATES HYPERFIBRINOLYSIS, WHEREAS PLATELOLYSIS SHUTS DOWN FIBRINOLYSIS: EVOLVING CONCEPTS OF THE SPECTRUM OF FIBRINOLYSIS IN RESPONSE TO SEVERE INJURY [J]. Shock, 2015,43(1): 39-46.

[457] Mosnier LO, Zlokovic BV, Griffin JH. Cytoprotective-selective activated protein C therapy for ischaemic stroke [J]. Thrombosis and Haemostasis, 2014,112(5): 883-892.

[458] Nasr DM, Biller J, Rabinstein AA. Use and In-Hospital Outcomes of Recombinant Tissue Plasminogen Activator in Pediatric Arterial Ischemic Stroke Patients [J]. Pediatric Neurology, 2014,51(5): 624-631.

[459] Olgun H, Buyukavci M, Ceviz N, et al. Clinical experience with recombinant tissue plasminogen activator in the management of intracardiac and arterial thrombosis in children [J]. Blood Coagulation & Fibrinolysis, 2014,25(7): 726-730.

[460] Park SJ, Jo DS, Shin JH, et al. Suppression of Cpn10 Increases Mitochondrial Fission and Dysfunction in Neuroblastoma Cells [J]. Plos One, 2014,9(11).

[461] Pekny T, Andersson D, Wilhelmsson U, et al. Short general anaesthesia induces prolonged changes in gene expression in the mouse hippocampus [J]. Acta Anaesthesiologica Scandinavica, 2014,58(9): 1127-1133.

[462] Proesmans M, Gijsens B, Van de Wijdeven P, et al. Clinical outcome of parapneumonic empyema in children treated according to a standardized medical treatment [J]. European Journal of Pediatrics, 2014,173(10): 1339-1345.

[463] Psallidas I, Corcoran JP, Rahman NM. Management of Parapneumonic Effusions and Empyema [J]. Seminars in Respiratory and Critical Care Medicine, 2014,35(6): 715-722.

[464] Qi J, Liu Y, Yang P, et al. Heat shock protein 90 inhibition by 17-Dimethylaminoethylamino-17-demethoxygeldanamycin protects blood-brain barrier integrity in cerebral ischemic stroke [J]. American Journal of Translational Research, 2015,7(10): 1826-1837.

[465] Raggam RB, Wagner J, Pruller F, et al. Soluble urokinase plasminogen activator receptor predicts mortality in patients with systemic inflammatory response syndrome [J]. Journal of Internal Medicine, 2014,276(6): 651-658.

[466] Rancourt RC, Ahmad A, Veress LA, et al. Antifibrinolytic Mechanisms in Acute Airway Injury after Sulfur Mustard Analog Inhalation [J]. American Journal of Respiratory Cell and Molecular Biology, 2014,51(4): 559-567.

[467] Raymond BBA, Jenkins C, Seymour LM, et al. Proteolytic processing of the cilium adhesin MHJ_0194 (P123(J)) in Mycoplasma hyopneumoniae generates a functionally diverse array of cleavage fragments that bind multiple host molecules [J]. Cellular Microbiology, 2015,17(3): 425-444.

[468] Rech MA, Day SA, Kast JM, et al. Major publications in the critical care pharmacotherapy literature: January-December 2013 [J]. American Journal of Health-System Pharmacy, 2015,72(3): 224-236.

[469] Reichsoellner M, Raggam RB, Wagner J, et al. Clinical Evaluation of Multiple Inflammation Biomarkers for Diagnosis and Prognosis for Patients with Systemic Inflammatory Response Syndrome [J]. Journal of Clinical Microbiology, 2014,52(11): 4063-4066.

[470] Ren WY, Wang ZH, Hua F, et al. Plasminogen Activator Inhibitor-1 Regulates LPS-Induced TLR4/MD-2 Pathway Activation and Inflammation in Alveolar Macrophages [J]. Inflammation, 2015,38(1): 384-393.

[471] Rincon F, Maltenfort M, Dey S, et al. The Prevalence and Impact of Mortality of the Acute Respiratory Distress Syndrome on Admissions of Patients With Ischemic Stroke in the United States [J]. Journal of Intensive Care Medicine, 2014,29(6): 357-364.

[472] Scheitz JF, Endres M, Heuschmann PU, et al. Reduced risk of poststroke pneumonia in thrombolyzed stroke patients with continued statin treatment [J]. International Journal of Stroke, 2015,10(1): 61-66.

[473] Schouten M, van't Veer C, Poulussen N, et al. The cytoprotective effects of endogenous activated protein C reduce activation of coagulation during murine pneumococcal pneumonia and sepsis [J]. Thrombosis Research, 2015,135(3): 537-543.

[474] Schuliga M. The Inflammatory Actions of Coagulant and Fibrinolytic Proteases in Disease [J]. Mediators of Inflammation, 2015.

[475] Shimada Y, Tanaka R, Shimura H, et al. PHOSPHORYLATION ENHANCES RECOMBINANT HSP27 NEUROPROTECTION AGAINST FOCAL CEREBRAL ISCHEMIA IN MICE [J]. Neuroscience, 2014,278: 113-121.

[476] Starr ME, Takahashi H, Okamura D, et al. Increased coagulation and suppressed generation of activated protein C in aged mice during intra-abdominal sepsis [J]. American Journal of Physiology-Heart and Circulatory Physiology, 2015,308(2): H83-H91.

[477] Stillion JR, Letendre JA. A clinical review of the pathophysiology, diagnosis, and treatment of pyothorax in dogs and cats [J]. Journal of Veterinary Emergency and Critical Care, 2015,25(1): 113-129.

[478] Swarbreck S, Secor D, Li FY, et al. Effect of ascorbate on fibrinolytic factors in septic mouse skeletal muscle [J]. Blood Coagulation & Fibrinolysis, 2014,25(7): 745-753.

[479] Tagliamonte M, Petrizzo A, Tornesello ML, et al. Antigen-specific vaccines for cancer treatment [J]. Human Vaccines & Immunotherapeutics, 2014,10(11): 3332-3346.

[480] Tanoglu A, Yamanel L, Inal V, et al. Appreciation of trimetazidine treatment in experimental sepsis rat model [J]. Bratislava Medical Journal-Bratislavske Lekarske Listy, 2015,116(2): 124-127.

[481] Tekin SO, Teksoz S, Terzioglu D, et al. Use of infliximab in treatment of acute pancreatitis [J]. Bratislava Medical Journal-Bratislavske Lekarske Listy, 2015,116(3): 167-172.

[482] Tsai HJ, Ding C, Tsao CM, et al. Effects of gabexate mesilate on coagulopathy and organ dysfunction in rats with endotoxemia: a potential use of thrombelastography in endotoxin-induced sepsis [J]. Blood Coagulation & Fibrinolysis, 2015,26(2): 175-184.

[483] van der Poll T, Herwald H. The coagulation system and its function in early immune defense [J]. Thrombosis and Haemostasis, 2014,112(4): 640-648.

[484] van Lieshout MHP, van der Poll T, van't Veer C. TLR4 inhibition impairs bacterial clearance in a therapeutic setting in murine abdominal sepsis [J]. Inflammation Research, 2014,63(11): 927-933.

[485] Vaschetto R, Navalesi P, Clemente N, et al. Osteopontin induces soluble urokinase-type plasminogen activator receptor production and release [J]. Minerva Anestesiologica, 2015,81(2): 157-165.

[486] Veress LA, Anderson DR, Hendry-Hofer TB, et al. Airway Tissue Plasminogen Activator Prevents Acute Mortality Due to Lethal Sulfur Mustard Inhalation [J]. Toxicological Sciences, 2015,143(1): 178-184.

[487] Wang X, Dinler BS, Vignjevic M, et al. Physiological and proteome studies of responses to heat stress during grain filling in contrasting wheat cultivars [J]. Plant Science, 2015,230: 33-50.

[488] Wang Y, Chen JM, Ling MH, et al. Hypochlorous Acid Generated by Neutrophils Inactivates ADAMTS13 AN OXIDATIVE MECHANISM FOR REGULATING ADAMTS13 PROTEOLYTIC ACTIVITY DURING INFLAMMATION [J]. Journal of Biological Chemistry, 2015,290(3): 1422-1431.

[489] Wigenstam E, Koch B, Bucht A, et al. N-acetyl cysteine improves the effects of corticosteroids in a mouse model of chlorine-induced acute lung injury [J]. Toxicology, 2015,328: 40-47.

[490] Wrotek A, Jackowska T, Pawlik K. Soluble Urokinase Plasminogen Activator Receptor: An Indicator of Pneumonia Severity in Children[A]. In: Respiratory Infections (Pokorski M, ed), Vol. 835, 2015: 1-7.

[491] Wu AL, Xiong YS, Li ZQ, et al. Correlation between single nucleotide polymorphisms in hypoxia-related genes and susceptibility to acute high-altitude pulmonary edema [J]. Genetics and Molecular Research, 2015,14(3): 11562-11572.

[492] Xu F, Diao R, Liu J, et al. Curcumin attenuates staphylococcus aureus-induced acute lung injury [J]. Clinical Respiratory Journal, 2015,9(1): 87-97.

[493] Xu L, Yu WK, Lin ZL, et al. Chemical sympathectomy attenuates inflammation, glycocalyx shedding and coagulation disorders in rats with acute traumatic coagulopathy [J]. Blood Coagulation & Fibrinolysis, 2015,26(2): 152-160.

[494] Xu L, Yu WK, Lin ZL, et al. Impact of beta-Adrenoceptor Blockade on Systemic Inflammation and Coagulation Disturbances in Rats with Acute Traumatic Coagulopathy [J]. Medical Science Monitor, 2015,21: 468-476.

[495] Xue GP, Drenth J, McIntyre CL. TaHsfA6f is a transcriptional activator that regulates a suite of heat stress protection genes in wheat (Triticum aestivum L.) including previously unknown Hsf targets [J]. Journal of Experimental Botany, 2015,66(3): 1025-1039.

[496] Yamaguti EMM, Brito MB, Ferriani RA, et al. Comparison of the hemostatic effects of a levonorgestrel-releasing intrauterine system and leuprolide acetate in women with endometriosis: A randomized clinical trial [J]. Thrombosis Research, 2014,134(6): 1193-1197.

[497] Yang SF, Liu BC, Ding WW, et al. Acute Superior Mesenteric Venous Thrombosis: Transcatheter Thrombolysis and Aspiration Thrombectomy Therapy by Combined Route of Superior Mesenteric Vein and Artery in Eight Patients [J]. Cardiovascular and Interventional Radiology, 2015,38(1): 88-99.

[498] Yilmaz MZ, Torun AC, Guzel A, et al. Ameliorative effect of Leflunomide on lung injury following an aspiration [J]. Bratislava Medical Journal-Bratislavske Lekarske Listy, 2015,116(3): 177-183.

[499] Yin WB, Li BY, Li XL, et al. Critical Role of Prohibitin in Endothelial Cell Apoptosis Caused by Glycated Low-density Lipoproteins and Protective Effects of Grape Seed Procyanidin B2 [J]. Journal of Cardiovascular Pharmacology, 2015,65(1): 13-21.

[500] Zateyshchikov D, Fattakhova E, Demchinsky V, et al. Late Silent Stent Abscess [J]. Cardiology, 2015,132(1): 65-67.

[501] Abrosimov SA, Bazhulin AP, Bolshakov AP, et al. Experimental Investigation into Polycrystalline and Single-Crystal Diamonds under Negative Pressures Formed by Picosecond Laser Pulses [J]. Doklady Physics, 2014,59(7): 309-312.

[502] Abrosimov SA, Bazhulin AP, Bol'shakov AP, et al. Generation of negative pressures and spallation phenomena in diamond exposed to a picosecond laser pulse [J]. Quantum Electronics, 2014,44(6): 530-534.

[503] Adamik B, Lipinska-Gediga MB, Kubler A. Soluble Urokinase-type Plasminogen Activator Receptor as a marker of outcome in severe sepsis [J]. Infection, 2011,39: S120-S120.

[504] Agrawal A, Zhuo HJ, Brady S, et al. Pathogenetic and predictive value of biomarkers in patients with ALI and lower severity of illness: results from two clinical trials [J]. American Journal of Physiology-Lung Cellular and Molecular Physiology, 2012,303(8): L634-L639.

[505] Alar T, Ozcelik C, Onat S, et al. Treatment of pediatric parapneumonic empyemas with pulmonary cavitary lesions [J]. Turk Gogus Kalp Damar Cerrahisi Dergisi-Turkish Journal of Thoracic and Cardiovascular Surgery, 2013,21(1): 84-88.

[506] Alberelli MA, De Candia E. Functional role of protease activated receptors in vascular biology [J]. Vascular Pharmacology, 2014,62(2): 72-81.

[507] Al-Khaled M, Matthis C, Eggers J. Predictors of In-hospital Mortality and the Risk of Symptomatic Intracerebral Hemorrhage after Thrombolytic Therapy with Recombinant Tissue Plasminogen Activator in Acute Ischemic Stroke [J]. Journal of Stroke & Cerebrovascular Diseases, 2014,23(1): 7-11.

[508] Al-Salih G, Al-Attar N, Delbosc S, et al. Role of Vegetation-Associated Protease Activity in Valve Destruction in Human Infective Endocarditis [J]. Plos One, 2012,7(9).

[509] Angchaisuksiri P. Coagulopathy in malaria [J]. Thrombosis Research, 2014,133(1): 5-9.

[510] Angelo LS, Wu JY, Meng F, et al. Combining Curcumin (Diferuloylmethane) and Heat Shock Protein Inhibition for Neurofibromatosis 2 Treatment: Analysis of Response and Resistance Pathways [J]. Molecular Cancer Therapeutics, 2011,10(11): 2094-2103.

[511] Ankermann T, Klein MO. Complicated pneumonia in children. Local complications [J]. Monatsschrift Kinderheilkunde, 2014,162(2): 130-136.

[512] Antonov O, Efimov S, Yanuka D, et al. Generation of converging strong shock wave formed by microsecond timescale underwater electrical explosion of spherical wire array [J]. Applied Physics Letters, 2013,102(12).

[513] Arancibia R, Oyarzun A, Silva D, et al. Tumor Necrosis Factor-alpha Inhibits Transforming Growth Factor-beta-Stimulated Myofibroblastic Differentiation and Extracellular Matrix Production in Human Gingival Fibroblasts [J]. Journal of Periodontology, 2013,84(5): 683-693.

[514] Armand R, Harti K, Lacroix R, et al. Prognostic value of plasmin generation linked to microparticles in severe septic states [J]. Annales Francaises D Anesthesie Et De Reanimation, 2013,32: A143-A143.

[515] Armangil D, Yurdakok M, Okur H, et al. Plasminogen Activator Inhibitor 1 4G/5G Polymorphism in Neonatal Respiratory Distress Syndrome [J]. Clinical and Applied Thrombosis-Hemostasis, 2011,17(4): 352-357.

[516] Armstrong PW, Gershlick AH, Goldstein P, et al. Fibrinolysis or Primary PCI in ST-Segment Elevation Myocardial Infarction [J]. New England Journal of Medicine, 2013,368(15): 1379-1387.

[517] Atahan K, Cokmez A, Bekoglu M, et al. The effect of antiseptic solution in central venous catheter care [J]. Bratislava Medical Journal-Bratislavske Lekarske Listy, 2012,113(9): 548-551.

[518] Attia A, Hassan H, Elsammak M. Prognostic value of urokinase plasminogen activator receptor (uPAR) and neutrophil CD64 expression in acute respiratory distress syndrome patients [J]. European Respiratory Journal, 2013,42.

[519] Augustin P, Alsalih G, Launey Y, et al. Predominant Role of Host Proteases in Myocardial Damage Associated with Infectious Endocarditis Induced by Enterococcus faecalis in a Rat Model [J]. Infection and Immunity, 2013,81(5): 1721-1729.

[520] Backes Y, van der Sluijs KF, Mackie DP, et al. Usefulness of suPAR as a biological marker in patients with systemic inflammation or infection: a systematic review [J]. Intensive Care Medicine, 2012,38(9): 1418-1428.

[521] Badin J, Francois M, Birmele B, et al. An unusual case of central venous catheter-related bacteremia [J]. Journal of Vascular Access, 2012,13(1): 122-123.

[522] Bae HB, Tadie JM, Jiang SN, et al. Vitronectin Inhibits Efferocytosis through Interactions with Apoptotic Cells as well as with Macrophages [J]. Journal of Immunology, 2013,190(5): 2273-2281.

[523] Bae YK. Creating nanostars with buckyballs [J]. Physics Letters A, 2013,377(45-48): 3304-3311.

[524] Balci C, Kaya E, Demirkan P, et al. Effects of abdominal compartment syndrome on gastric emptying time [J]. Bratislava Medical Journal-Bratislavske Lekarske Listy, 2012,113(2): 99-102.

[525] Bao MH, Zheng YG, Westerhout CM, et al. Prognostic implications of quantitative evaluation of baseline Q-wave width in ST-segment elevation myocardial infarction [J]. Journal of Electrocardiology, 2014,47(4): 465-471.

[526] Baradari AG, Khezri HD, Arabi S. Comparison of antibacterial effects of oral rinses chlorhexidine and herbal mouth wash in patients admitted to intensive care unit [J]. Bratislava Medical Journal-Bratislavske Lekarske Listy, 2012,113(9): 556-560.

[527] Barra A, Del Ferraro G, Tantari D. Mean field spin glasses treated with PDE techniques [J]. European Physical Journal B, 2013,86(7).

[528] Barratt-Due A, Thorgersen EB, Egge K, et al. Combined Inhibition of Complement (C5) and CD14 Markedly Attenuates Inflammation, Thrombogenicity, and Hemodynamic Changes in Porcine Sepsis [J]. Journal of Immunology, 2013,191(2): 819-827.

[529] Barreto GE, White RE, Xu LJ, et al. Effects of heat shock protein 72 (Hsp72) on evolution of astrocyte activation following stroke in the mouse [J]. Experimental Neurology, 2012,238(2): 284-296.

[530] Barve A, Jin W, Cheng K. Prostate cancer relevant antigens and enzymes for targeted drug delivery [J]. Journal of Controlled Release, 2014,187: 118-132.

[531] Bastarache JA, Ware LB, Girard TD, et al. Markers of Inflammation and Coagulation May Be Modulated by Enteral Feeding Strategy [J]. Journal of Parenteral and Enteral Nutrition, 2012,36(6): 732-740.

[532] Basu A, Jain P, Sarkar P, et al. Dengue virus infection of SK Hep1 cells: inhibition of in vitro angiogenesis and altered cytomorphology by expressed viral envelope glycoprotein [J]. Fems Immunology and Medical Microbiology, 2011,62(2): 140-147.

[533] Baumer W, Herrling GM, Feige K. Pharmacokinetics and thrombolytic effects of the recombinant tissue-type plasminogen activator in horses [J]. Bmc Veterinary Research, 2013,9.

[534] Belleannee C, Belghazi M, Labas V, et al. Purification and identification of sperm surface proteins and changes during epididymal maturation [J]. Proteomics, 2011,11(10): 1952-1964.

[535] Bendok BR, Arnaout O. Intracranial Retrievable Stenting for Septic Emboli [J]. World Neurosurgery, 2012,77(3-4): 470-471.

[536] Benes J, Zeman J, Pouckova P, et al. Biological effects of tandem shock waves demonstrated on magnetic resonance [J]. Bratislava Medical Journal-Bratislavske Lekarske Listy, 2012,113(6): 335-338.

[537] Bhandary YP, Shetty SK, Marudamuthu AS, et al. Regulation of alveolar epithelial cell apoptosis and pulmonary fibrosis by coordinate expression of components of the fibrinolytic system [J]. American Journal of Physiology-Lung Cellular and Molecular Physiology, 2012,302(5): L463-L473.

[538] Bhandary YP, Shetty SK, Marudamuthu AS, et al. Regulation of Lung Injury and Fibrosis by p53-Mediated Changes in Urokinase and Plasminogen Activator Inhibitor-1 [J]. American Journal of Pathology, 2013,183(1): 131-143.

[539] Bhat R, Monagle P. The preterm infant with thrombosis [J]. Archives of Disease in Childhood-Fetal and Neonatal Edition, 2012,97(6): F423-F428.

[540] Bhatnagar R, Maskell NA. Treatment of Complicated Pleural Effusions in 2013 [J]. Clinics in Chest Medicine, 2013,34(1): 47-+.

[541] Bhatt NS, Solhpour A, Balan P, et al. Comparison of In-Hospital Outcomes With Low-Dose Fibrinolytic Therapy Followed by Urgent Percutaneous Coronary Intervention Versus Percutaneous Coronary Intervention Alone for Treatment of ST-Elevation Myocardial Infarction [J]. American Journal of Cardiology, 2013,111(11): 1576-1579.

[542] Bice T, Cox CE, Carson SS. Cost and Health Care Utilization in ARDSDifferent from Other Critical Illness? [J]. Seminars in Respiratory and Critical Care Medicine, 2013,34(4): 529-536.

[543] Bloos F, Reinhart K. Rapid diagnosis of sepsis [J]. Virulence, 2014,5(1): 154-160.

[544] Bockenhauer D. Draining the edema: a new role for aquaretics? [J]. Pediatric Nephrology, 2014,29(5): 767-769.

[545] Bodenant M, Debette S, Cordonnier C, et al. A very early neurological improvement after intravenous thrombolysis for acute cerebral ischaemia does not necessarily predict a favourable outcome [J]. Acta Neurologica Belgica, 2013,113(1): 67-72.

[546] Boehme AK, Kapoor N, Albright KC, et al. Predictors of Systemic Inflammatory Response Syndrome in Ischemic Stroke Undergoing Systemic Thrombolysis with Intravenous Tissue Plasminogen Activator [J]. Journal of Stroke & Cerebrovascular Diseases, 2014,23(4): E271-E276.

[547] Boehme AK, Kumar AD, Dorsey AM, et al. Infections Present on Admission Compared with Hospital-Acquired Infections in Acute Ischemic Stroke Patients [J]. Journal of Stroke & Cerebrovascular Diseases, 2013,22(8): E582-E589.

[548] Bonkain F, Racape J, Goncalvez I, et al. Prevention of Tunneled Cuffed Hemodialysis Catheter-Related Dysfunction and Bacteremia by a Neutral-Valve Closed-System Connector: A Single-Center Randomized Controlled Trial [J]. American Journal of Kidney Diseases, 2013,61(3): 459-465.

[549] Boonyasai RT, Steinberg DI, Schleyer AM, et al. Update in Hospital Medicine for the General Internist 2010-2011 [J]. Journal of General Internal Medicine, 2011,26(12): 1492-1497.

[550] Britland S, Hoyle M. Transcriptional gene silencing of kallikrein 5 and kallikrein 7 using siRNA prevents epithelial cell detachment induced by alkaline shock in an in vitro model of eczema [J]. Biotechnology Progress, 2012,28(2): 485-489.

[551] Brooks AL, Lyerly MJ, Sands KA, et al. Risk of Pneumonia Associated with Zero-Degree Head Positioning (Heads Down) In Acute Ischemic Stroke Patients Treated with Intravenous tPA [J]. Cerebrovascular Diseases, 2013,35: 667-667.

[552] Brouwer MC, Meijers JCM, Baas F, et al. Plasminogen activator inhibitor-1 influences cerebrovascular complications and death in pneumococcal meningitis [J]. Acta Neuropathologica, 2014,127(4): 553-564.

[553] Brunelli A. DeepVein Thrombosis/Pulmonary Embolism: Prophylaxis, Diagnosis, and Management [J]. Thoracic Surgery Clinics, 2012,22(1): 25-+.

[554] Buckley S, Shi W, Carraro G, et al. The Milieu of Damaged Alveolar Epithelial Type 2 Cells Stimulates Alveolar Wound Repair by Endogenous and Exogenous Progenitors [J]. American Journal of Respiratory Cell and Molecular Biology, 2011,45(6): 1212-1221.

[555] Bundesmann MM, Wagner TE, Chow YH, et al. Role of Urokinase Plasminogen Activator Receptor-Associated Protein in Mouse Lung [J]. American Journal of Respiratory Cell and Molecular Biology, 2012,46(2): 233-239.

[556] Burns JD, Green DM, Metivier K, et al. Intensive Care Management of Acute Ischemic Stroke [J]. Emergency Medicine Clinics of North America, 2012,30(3): 713-+.

[557] Byrne RM, Taha AG, Avgerinos E, et al. Contemporary outcomes of endovascular interventions for acute limb ischemia [J]. Journal of Vascular Surgery, 2014,59(4): 988-995.

[558] Caluza ACV, Barbosa AH, Goncalves I, et al. ST-Elevation Myocardial Infarction Network: Systematization in 205 Cases Reduced Clinical Events in the Public Health Care System [J]. Arquivos Brasileiros De Cardiologia, 2012,99(5): 1040-1048.

[559] Cardenas JC, Matijevic N, Baer LA, et al. ELEVATED TISSUE PLASMINOGEN ACTIVATOR AND REDUCED PLASMINOGEN ACTIVATOR INHIBITOR PROMOTE HYPERFIBRINOLYSIS IN TRAUMA PATIENTS [J]. Shock, 2014,41(6): 514-521.

[560] Cardenas JC, Wade CE, Holcomb JB. Mechanisms of trauma-induced coagulopathy [J]. Current Opinion in Hematology, 2014,21(5): 404-409.

[561] Cassidy MR, Sherburne AC, Sheldon HK, et al. Histone deacetylase inhibitors decrease intra-abdominal adhesions with one intraoperative dose by reducing peritoneal fibrin deposition pathways [J]. Surgery, 2014,155(2): 234-244.

[562] Cekmez F, Aydemir G, Yildirim S, et al. DIAGNOSTIC VALUE OF 25-HYDROXYVITAMIN D LEVEL AND NEW CYTOKINES IN NEONATAL SEPSIS [J]. European Journal of Inflammation, 2014,12(2): 297-304.

[563] Cekmez Y, Cekmez F, Ozkaya E, et al. uPAR, IL-33, and ST2 Values as a Predictor of Subclinical Chorioamnionitis in Preterm Premature Rupture of Membranes [J]. Journal of Interferon and Cytokine Research, 2013,33(12): 778-782.

[564] Celen S, Ozyer S, Unlu S, et al. Rapidly progressing A/H1N1 (09) pandemic influenza virus infection in pregnancy [J]. Bratislava Medical Journal-Bratislavske Lekarske Listy, 2012,113(6): 379-381.

[565] Cerny M, Jedelsky PL, Novak J, et al. Cytokinin modulates proteomic, transcriptomic and growth responses to temperature shocks in Arabidopsis [J]. Plant Cell and Environment, 2014,37(7): 1641-1655.

[566] Chai FY, Kuan YC. Massive hemothorax following administration of intrapleural streptokinase [J]. Annals of Thoracic Medicine, 2011,6(3): 149-151.

[567] Chan KYY, Leung FWL, Lam HS, et al. Immunoregulatory Protein Profiles of Necrotizing Enterocolitis versus Spontaneous Intestinal Perforation in Preterm Infants [J]. Plos One, 2012,7(5).

[568] Chen CJ, Unger C, Hoffmann W, et al. Characterization and Comparison of 2 Distinct Epidemic Community-Associated Methicillin-Resistant Staphylococcus aureus Clones of ST59 Lineage [J]. Plos One, 2013,8(9).

[569] Chen H, Zheng DH, Abbott J, et al. Myxomavirus-Derived Serpin Prolongs Survival and Reduces Inflammation and Hemorrhage in an Unrelated Lethal Mouse Viral Infection [J]. Antimicrobial Agents and Chemotherapy, 2013,57(9): 4114-4127.

[570] Chen TT, Jiandong L, Wang G, et al. Combined Treatment of Ulinastatin and Tranexamic Acid Provides Beneficial Effects by Inhibiting Inflammatory and Fibrinolytic Response in Patients Undergoing Heart Valve Replacement Surgery [J]. Heart Surgery Forum, 2013,16(1): E38-E47.

[571] Chen TW, Liang YN, Feng D, et al. Metformin inhibits proliferation and promotes apoptosis of HER2 positive breast cancer cells by downregulating HSP90 [J]. Journal of Buon, 2013,18(1): 51-56.

[572] Cheung EW, Aponte-Patel L, Bacha EA, et al. Successful Treatment of Severe Mechanical Mitral Valve Thrombosis With Tissue Plasminogen Activator in a 7-Month-Old Infant [J]. Pediatric Cardiology, 2013,34(8): 1903-1907.

[573] Chian CF, Chiang CH, Chu YJ, et al. APOCYNIN ATTENUATES LIPOPOLYSACCHARIDE-INDUCED LUNG INJURY IN AN ISOLATED AND PERFUSED RAT LUNG MODEL [J]. Shock, 2012,38(2): 196-202.

[574] Chian CF, Chiang CH, Chuang CH, et al. Inhibitor of nuclear factor-kappa B, SN50, attenuates lipopolysaccharide-induced lung injury in an isolated and perfused rat lung model [J]. Translational Research, 2014,163(3): 211-220.

[575] Chibuk TK, Cohen E, Robinson JL, et al. Paediatric complicated pneumonia: Diagnosis and management of empyema [J]. Paediatrics & Child Health, 2011,16(7): 425-427.

[576] Chibuk TK, Cohen E, Robinson JL, et al. Complex pediatric pneumonia: diagnosis and empyema care [J]. Paediatrics & Child Health, 2011,16(7): 428-429.

[577] Chow AM, Beraud E, Tang DWF, et al. Hsp60 protein pattern in coral is altered by environmental changes in light and temperature [J]. Comparative Biochemistry and Physiology a-Molecular & Integrative Physiology, 2012,161(3): 349-353.

[578] Christiaans SC, Wagener BM, Esmon CT, et al. Protein C and acute inflammation: a clinical and biological perspective [J]. American Journal of Physiology-Lung Cellular and Molecular Physiology, 2013,305(7): L455-L466.

[579] Chuang YC, Lei HY, Lin YS, et al. Dengue Virus-Induced Autoantibodies Bind to Plasminogen and Enhance Its Activation [J]. Journal of Immunology, 2011,187(12): 6483-6490.

[580] Chuansumrit A, Chaiyaratana W. Hemostatic derangement in dengue hemorrhagic fever [J]. Thrombosis Research, 2014,133(1): 10-16.

[581] Chung LP, Waterer GW. Genetic predisposition to respiratory infection and sepsis [J]. Critical Reviews in Clinical Laboratory Sciences, 2011,48(5-6): 250-268.

[582] Cohen JE, Rabinstein AA, Ramirez-de-Noriega F, et al. Excellent rates of recanalization and good functional outcome after stent-based thrombectomy for acute middle cerebral artery occlusion. Is it time for a paradigm shift? [J]. Journal of Clinical Neuroscience, 2013,20(9): 1219-1223.

[583] Cohen MJ, Call M, Nelson M, et al. Critical Role of Activated Protein C in Early Coagulopathy and Later Organ Failure, Infection and Death in Trauma Patients [J]. Annals of Surgery, 2012,255(2): 379-385.

[584] Cole E, Davenport R, De-Ath H, et al. Coagulation system changes associated with susceptibility to infection in trauma patients [J]. Journal of Trauma and Acute Care Surgery, 2013,74(1): 51-57.

[585] Conhaim RL, Watson KE, Dovi WF, et al. INHALED THROMBOLYTICS REDUCE LUNG MICROCLOT AND LEUKOCYTE INFILTRATION AFTER ACUTE BLOOD LOSS [J]. Shock, 2014,41(6): 528-536.

[586] Cornet AD, Hofstra JJ, Vlaar AP, et al. Activated protein C attenuates pulmonary coagulopathy in patients with acute respiratory distress syndrome [J]. Journal of Thrombosis and Haemostasis, 2013,11(5): 894-901.

[587] Cornet AD, Hofstra JJ, Vlaar AP, et al. NEBULIZED ANTICOAGULANTS LIMIT COAGULOPATHY BUT NOT INFLAMMATION IN PSEUDOMONAS AERUGINOSA-INDUCED PNEUMONIA IN RATS [J]. Shock, 2011,36(4): 417-423.

[588] Coskun F, Saylam B, Kulah B, et al. Comparison of the therapeutic effects of tri-iodothyronine and methylprednisolone during early sepsis in laboratory animals [J]. Bratislava Medical Journal-Bratislavske Lekarske Listy, 2012,113(6): 339-346.

[589] Cottee NS, Wilson IW, Tan DKY, et al. Understanding the molecular events underpinning cultivar differences in the physiological performance and heat tolerance of cotton (Gossypium hirsutum) [J]. Functional Plant Biology, 2014,41(1): 56-67.

[590] Cotton BA, Harvin JA, Kostousouv V, et al. Hyperfibrinolysis at admission is an uncommon but highly lethal event associated with shock and prehospital fluid administration [J]. Journal of Trauma and Acute Care Surgery, 2012,73(2): 365-370.

[591] Courey AJ, Horowitz JC, Kim KK, et al. The vitronectin-binding function of PAI-1 exacerbates lung fibrosis in mice [J]. Blood, 2011,118(8): 2313-2321.

[592] Couture R, Blaes N, Girolami JP. Kinin Receptors in Vascular Biology and Pathology [J]. Current Vascular Pharmacology, 2014,12(2): 223-248.

[593] Czarnecki A, Welsh RC, Yan RT, et al. Reperfusion Strategies and Outcomes of ST-Segment Elevation Myocardial Infarction Patients in Canada: Observations From the Global Registry of Acute Coronary Events (GRACE) and the Canadian Registry of Acute Coronary Events (CANRACE) [J]. Canadian Journal of Cardiology, 2012,28(1): 40-47.

[594] Dale AP, Read RC. Genetic susceptibility to meningococcal infection [J]. Expert Review of Anti-Infective Therapy, 2013,11(2): 187-199.

[595] Danisoglu ME, Aytac B, Kilicaslan H, et al. Reduction of shock wave lithotripsy-induced renal tubular injury by tadalafil [J]. Bratislava Medical Journal-Bratislavske Lekarske Listy, 2013,114(11): 616-620.

[596] de Abreu CEB, Araujo GD, Monteiro-Moreira ACD, et al. Proteomic analysis of salt stress and recovery in leaves of Vigna unguiculata cultivars differing in salt tolerance [J]. Plant Cell Reports, 2014,33(8): 1289-1306.

[597] De Felice F, Fiorilli R, Parma A, et al. Comparison of One-Year Outcome of Patients Aged < 75 Years Versus >= 75 Years Undergoing "Rescue" Percutaneous Coronary Intervention [J]. American Journal of Cardiology, 2011,108(8): 1075-1080.

[598] De Felice F, Fiorilli R, Parma A, et al. One-year clinical outcome of patients treated with or without abciximab in rescue coronary angioplasty [J]. International Journal of Cardiology, 2013,163(3): 294-298.

[599] De Marchis GM, Katan M, Weck A, et al. Copeptin and risk stratification in patients with ischemic stroke and transient ischemic attack: The CoRisk Study [J]. International Journal of Stroke, 2013,8(3): 214-218.

[600] DeBarros M, Hatch Q, Porta CR, et al. Tranexamic acid corrects fibrinolysis in the presence of acidemia in a swine model of severe ischemic reperfusion [J]. Journal of Trauma and Acute Care Surgery, 2014,76(3): 625-632.

[601] Della Bona R, Liuzzo G, Pedicino D, et al. Anti-inflammatory Treatment of Acute Coronary Syndromes [J]. Current Pharmaceutical Design, 2011,17(37): 4172-4189.

[602] Desa V, Green R. Cavernous Sinus Thrombosis: Current Therapy [J]. Journal of Oral and Maxillofacial Surgery, 2012,70(9): 2085-2091.

[603] Desai B, Mattson J, Paintal H, et al. Differential expression of monocyte/macrophage-selective markers in human idiopathic pulmonary fibrosis [J]. Experimental Lung Research, 2011,37(4): 227-238.

[604] Desai H, Agrawal A. Pulmonary Emergencies Pneumonia, Acute Respiratory Distress Syndrome, Lung Abscess, and Empyema [J]. Medical Clinics of North America, 2012,96(6): 1127-+.

[605] Deutsch MA, Cleuziou J, Noebauer C, et al. Successful Management of Neonatal Myocardial Infarction with ECMO and Intracoronary r-tPA lysis [J]. Congenital Heart Disease, 2014,9(5): E169-E174.

[606] Djamiatun K, Faradz SMH, Setiati TE, et al. Increase of Plasminogen Activator Inhibitor-1 and Decrease of Transforming Growth Factor-Beta 1 in Children with Dengue Haemorrhagic Fever in Indonesia [J]. Journal of Tropical Pediatrics, 2011,57(6): 424-432.

[607] Donadello K, Scolletta S, Covajes C, et al. suPAR as a prognostic biomarker in sepsis [J]. Bmc Medicine, 2012,10.

[608] Donadello K, Scolletta S, Taccone FS, et al. Soluble urokinase-type plasminogen activator receptor as a prognostic biomarker in critically ill patients [J]. Journal of Critical Care, 2014,29(1): 144-149.

[609] Donet M, Brassart-Pasco S, Salesse S, et al. Elastin peptides regulate HT-1080 fibrosarcoma cell migration and invasion through an Hsp90-dependent mechanism [J]. British Journal of Cancer, 2014,111(1): 139-148.

[610] DuBose J, Inaba K, Demetriades D, et al. Management of post-traumatic retained hemothorax: A prospective, observational, multicenter AAST study [J]. Journal of Trauma and Acute Care Surgery, 2012,72(1): 11-22.

[611] Dumas EK, Cox PM, Fullenwider CO, et al. Anthrax Lethal Toxin-Induced Gene Expression Changes in Mouse Lung [J]. Toxins, 2011,3(9): 1111-1130.

[612] Dupuy AM, Philippart F, Pean Y, et al. Role of biomarkers in the management of antibiotic therapy: an expert panel review: I - currently available biomarkers for clinical use in acute infections [J]. Annals of Intensive Care, 2013,3.

[613] Enkhbaatar P. Activated Protein C: From Excitement to Disappointment? [J]. Critical Care Medicine, 2013,41(10): 2463-2464.

[614] Enkhbaatar P, Traber DL. Differential host response to Burkholderia pseudomallei: Tissue plasminogen activator [J]. Critical Care Medicine, 2012,40(7): 2257-2257.

[615] Erer D, Dursun AD, Oktar GL, et al. The effects of iloprost on lung injury induced by skeletal muscle ischemia-reperfusion [J]. Bratislava Medical Journal-Bratislavske Lekarske Listy, 2014,115(7): 405-410.

[616] Esmon CT. Molecular circuits in thrombosis and inflammation [J]. Thrombosis and Haemostasis, 2013,109(3): 416-420.

[617] Esmon CT, Xu J, Lupu F. Innate immunity and coagulation [J]. Journal of Thrombosis and Haemostasis, 2011,9: 182-188.

[618] Eugen-Olsen J. suPAR - a future risk marker in bacteremia [J]. Journal of Internal Medicine, 2011,270(1): 29-31.

[619] Faria RS, Sartori CR, Canova F, et al. CLASSICAL AVERSIVE CONDITIONING INDUCES INCREASED EXPRESSION OF MATURE-BDNF IN THE HIPPOCAMPUS AND AMYGDALA OF PIGEONS [J]. Neuroscience, 2013,255: 122-133.

[620] Farina F, Sancini G, Longhin E, et al. Milan PM1 Induces Adverse Effects on Mice Lungs and Cardiovascular System [J]. Biomed Research International, 2013.

[621] Ferrigno L, Bloch R, Threlkeld J, et al. Management of pulmonary embolism with rheolytic thrombectomy [J]. Canadian Respiratory Journal, 2011,18(4): E52-E58.

[622] Fourrier F. Severe sepsis, coagulation, and fibrinolysis: Dead end or one way? [J]. Critical Care Medicine, 2012,40(9): 2704-2708.

[623] Franco AA, Hu L, Grim CJ, et al. Characterization of Putative Virulence Genes on the Related RepFIB Plasmids Harbored by Cronobacter spp [J]. Applied and Environmental Microbiology, 2011,77(10): 3255-3267.

[624] Fulde M, Steinert M, Bergmann S. Interaction of streptococcal plasminogen binding proteins with the host fibrinolytic system [J]. Frontiers in Cellular and Infection Microbiology, 2013,3.

[625] Galkova K, Vrabelova M. Normalization of blood lactate as early end-point of polytrauma treatment [J]. Bratislava Medical Journal-Bratislavske Lekarske Listy, 2013,114(11): 637-641.

[626] Gando S. Role of Fibrinolysis in Sepsis [J]. Seminars in Thrombosis and Hemostasis, 2013,39(4): 392-399.

[627] Gando S, Sawamura A, Hayakawa M. Trauma, Shock, and Disseminated Intravascular Coagulation Lessons from the Classical Literature [J]. Annals of Surgery, 2011,254(1): 10-19.

[628] Garcia A, Gander JW, Gross ER, et al. The use of recombinant tissue-type plasminogen activator in a newborn with an intracardiac thrombus developed during extracorporeal membrane oxygenation [J]. Journal of Pediatric Surgery, 2011,46(10): 2021-2024.

[629] Gasior AC, Knott EM, St Peter SD. Management of peripherally inserted central catheter associated deep vein thrombosis in children [J]. Pediatric Surgery International, 2013,29(5): 445-449.

[630] Gazia MMA, El-Kordy EAM. Role of Activated Protein C and Soya-bean in Experimental Lung Toxicity in Adult Male Albino Rat: Histological and Immunohistochemical Studies [J]. Life Science Journal-Acta Zhengzhou University Overseas Edition, 2012,9(2): 73-85.

[631] Genet GF, Johansson PI, Meyer MAS, et al. Trauma-Induced Coagulopathy: Standard Coagulation Tests, Biomarkers of Coagulopathy, and Endothelial Damage in Patients with Traumatic Brain Injury [J]. Journal of Neurotrauma, 2013,30(4): 301-306.

[632] Genet GF, Ostrowski SR, Sorensen AM, et al. Detection of tPA-Induced Hyperfibrinolysis in Whole Blood by RapidTEG, KaolinTEG, and Functional FibrinogenTEG in Healthy Individuals [J]. Clinical and Applied Thrombosis-Hemostasis, 2012,18(6): 638-644.

[633] Gerischer LM, Fiebach JB, Scheitz JF, et al. Magnetic Resonance Imaging-Based versus Computed Tomography-Based Thrombolysis in Acute Ischemic Stroke: Comparison of Safety and Efficacy within a Cohort Study [J]. Cerebrovascular Diseases, 2013,35(3): 250-256.

[634] Giamarellos-Bourboulis EJ, Norrby-Teglund A, Mylona V, et al. Risk assessment in sepsis: a new prognostication rule by APACHE II score and serum soluble urokinase plasminogen activator receptor [J]. Critical Care, 2012,16(4).

[635] Girard TD, Ware LB, Bernard GR, et al. Associations of markers of inflammation and coagulation with delirium during critical illness [J]. Intensive Care Medicine, 2012,38(12): 1965-1973.

[636] Glas GJ, Van der Sluijs KF, Schultz MJ, et al. Bronchoalveolar hemostasis in lung injury and acute respiratory distress syndrome [J]. Journal of Thrombosis and Haemostasis, 2013,11(1): 17-25.

[637] Goldin AB, Parimi C, LaRiviere C, et al. Outcomes associated with type of intervention and timing in complex pediatric empyema [J]. American Journal of Surgery, 2012,203(5): 665-673.

[638] Gomez-Sucerquia LJ, Blas-Garcia A, Marti-Cabrera M, et al. Profile of stress and toxicity gene expression in human hepatic cells treated with Efavirenz [J]. Antiviral Research, 2012,94(3): 232-241.

[639] Goodwin JE, Feng Y, Velazquez H, et al. Endothelial glucocorticoid receptor is required for protection against sepsis [J]. Proceedings of the National Academy of Sciences of the United States of America, 2013,110(1): 306-311.

[640] Goolaerts A, Lafargue M, Song YL, et al. PAI-1 is an essential component of the pulmonary host response during Pseudomonas aeruginosa pneumonia in mice [J]. Thorax, 2011,66(9): 788-796.

[641] Gormus ZI, Celik JB, Ergene N, et al. Does preoperative administration of allopurinol protect the lungs from ischemia-reperfusion injury occuring during cardiopulmonary bypass? [J]. Bratislava Medical Journal-Bratislavske Lekarske Listy, 2013,114(10): 561-565.

[642] Gralinski LE, Bankhead A, Jeng S, et al. Mechanisms of Severe Acute Respiratory Syndrome Coronavirus-Induced Acute Lung Injury [J]. Mbio, 2013,4(4).

[643] Gul EE, Can I, Kayrak M, et al. Thrombolysis in patients with pulmonary embolism and elevated heart-type fatty acid-binding protein levels [J]. Journal of Thrombosis and Thrombolysis, 2014,37(4): 483-489.

[644] Guo YZ, Li JN, Hagstrom E, et al. Beneficial and Detrimental Effects of Plasmin(ogen) during Infection and Sepsis in Mice [J]. Plos One, 2011,6(9).

[645] Harslund J, Frees D, Leifsson PS, et al. The role of Serpine-1 and Tissue inhibitor of metalloproteinase type-1 in early host responses to Staphylococcus aureus intracutaneous infection of mice [J]. Pathogens and Disease, 2013,68(3): 96-104.

[646] Hasanoglu HC, Hezer H, Karalezli A, et al. Half- Dose Recombinant Tissue Plasminogen Activator Treatment in Venous Thromboembolism [J]. Journal of Investigative Medicine, 2014,62(1): 71-77.

[647] Hasegawa T, Oshima Y, Maruo A, et al. Pediatric Cardiothoracic Surgery in Patients With Unilateral Pulmonary Agenesis or Aplasia [J]. Annals of Thoracic Surgery, 2014,97(5): 1652-1658.

[648] Hassan H, Attia A, Raslan H, et al. PROGNOSTIC VALUE OF UROKINASE PLASMINOGEN ACTIVATOR RECEPTOR (uPAR) AND NEUTROPHIL CD64 EXPRESSION IN ACUTE RESPIRATORY DISTRESS SYNDROME PATIENTS [J]. European Journal of Inflammation, 2012,10(2): 175-183.

[649] Hawryluk JM, Moreira TS, Takakura AC, et al. KCNQ Channels Determine Serotonergic Modulation of Ventral Surface Chemoreceptors and Respiratory Drive [J]. Journal of Neuroscience, 2012,32(47): 16943-16952.

[650] Hayakawa M, Sawamura A, Gando S, et al. A low TAFI activity and insufficient activation of fibrinolysis by both plasmin and neutrophil elastase promote organ dysfunction in disseminated intravascular coagulation associated with sepsis [J]. Thrombosis Research, 2012,130(6): 906-913.

[651] Hayes WN, Watson AR, Callaghan N, et al. Vascular access: choice and complications in European paediatric haemodialysis units [J]. Pediatric Nephrology, 2012,27(6): 999-1004.

[652] Henczka M, Djas M, Filipek K. Optimisation of a direct plating method for the detection and enumeration of Alicyclobacillus acidoterrestris spores [J]. Journal of Microbiological Methods, 2013,92(1): 1-8.

[653] Henriquez-Camacho C, Losa J. Biomarkers for Sepsis [J]. Biomed Research International, 2014.

[654] Hoenigl M, Raggam RB, Wagner J, et al. Diagnostic accuracy of soluble urokinase plasminogen activator receptor (suPAR) for prediction of bacteremia in patients with systemic inflammatory response syndrome [J]. Clinical Biochemistry, 2013,46(3): 225-229.

[655] Hoffmeister L, Lavados PM, Comas M, et al. Performance measures for in-hospital care of acute ischemic stroke in public hospitals in Chile [J]. Bmc Neurology, 2013,13.

[656] Hofstra JJ, Cornet AD, Declerck PJ, et al. Nebulized Fibrinolytic Agents Improve Pulmonary Fibrinolysis but Not Inflammation in Rat Models of Direct and Indirect Acute Lung Injury [J]. Plos One, 2013,8(2).

[657] Hofstra JJ, Vlaar AP, Knape P, et al. Pulmonary Activation of Coagulation and Inhibition of Fibrinolysis After Burn Injuries and Inhalation Trauma [J]. Journal of Trauma-Injury Infection and Critical Care, 2011,70(6): 1389-1397.

[658] Hook KM, Abrams CS. The Loss of Homeostasis in Hemostasis: New Approaches in Treating and Understanding Acute Disseminated Intravascular Coagulation in Critically Ill Patients [J]. Cts-Clinical and Translational Science, 2012,5(1): 85-92.

[659] Hua F, Ren WY, Zhu L. Plasminogen activator inhibitor type-1 deficiency exaggerates LPS-induced acute lung injury through enhancing Toll-like receptor 4 signaling pathway [J]. Blood Coagulation & Fibrinolysis, 2011,22(6): 480-486.

[660] Huang H, Moller IM, Song SQ. Proteomics of desiccation tolerance during development and germination of maize embryos [J]. Journal of Proteomics, 2012,75(4): 1247-1262.

[661] Huang LT, Chou HC, Wang LF, et al. Tissue plasminogen activator attenuates ventilator-induced lung injury in rats [J]. Acta Pharmacologica Sinica, 2012,33(8): 991-997.

[662] Huang LT, Chou HC, Wang LF, et al. Tissue plasminogen activator attenuates ventilator-induced lung injury in rats (vol 33, pg 991, 2012) [J]. Acta Pharmacologica Sinica, 2014,35(9): 1226-1226.

[663] Huang TC, Chang HY, Chen CY, et al. Silencing of miR-124 induces neuroblastoma SK-N-SH cell differentiation, cell cycle arrest and apoptosis through promoting AHR [J]. Febs Letters, 2011,585(22): 3582-3586.

[664] Huggins JT, Doelken P, Sahn SA. Intrapleural therapy [J]. Respirology, 2011,16(6): 891-899.

[665] Huq MA, Takeyama N, Harada M, et al. 4G/5G Polymorphism of the Plasminogen Activator Inhibitor-1 Gene Is Associated with Multiple Organ Dysfunction in Critically III Patients [J]. Acta Haematologica, 2012,127(2): 72-80.

[666] Hutan M, Sen HM, Skultety J, et al. Use of intraabdominal VAC (Vacuum Assisted Closure) lowers mortality and morbidity in patients with open abdomen [J]. Bratislava Medical Journal-Bratislavske Lekarske Listy, 2013,114(8): 451-454.

[667] Huttunen R, Syrjanen J, Vuento R, et al. Plasma level of soluble urokinase-type plasminogen activator receptor as a predictor of disease severity and case fatality in patients with bacteraemia: a prospective cohort study [J]. Journal of Internal Medicine, 2011,270(1): 32-40.

[668] Hwang H, Cho MH, Bhoo SH, et al. Proteome analysis of chlorotic leaves of the Arabidopsis mex1 mutant defective in the mobilization of starch degradation products [J]. Plant Biotechnology Reports, 2013,7(3): 321-330.

[669] Hynes BG, Margey RJ, Ruggiero N, et al. Endovascular Management of Acute Limb Ischemia [J]. Annals of Vascular Surgery, 2012,26(1): 110-124.

[670] Ideh RC, Pollock L, Sanneh A, et al. Management of persistent purulent pericarditis using streptokinase for intrapericardial fibrinolysis [J]. Paediatrics and International Child Health, 2014,34(3): 220-223.

[671] Ikeuchi H, Kinjo T, Klinman DM. Effect of Suppressive Oligodeoxynucleotides on the Development of Inflammation-Induced Papillomas [J]. Cancer Prevention Research, 2011,4(5): 752-757.

[672] Islam S, Calkins CM, Goldin AB, et al. The diagnosis and management of empyema in children: a comprehensive review from the APSA Outcomes and Clinical Trials Committee [J]. Journal of Pediatric Surgery, 2012,47(11): 2101-2110.

[673] Israel EN, Blackmer AB. Tissue Plasminogen Activator for the Treatment of Parapneumonic Effusions in Pediatric Patients [J]. Pharmacotherapy, 2014,34(5): 521-532.

[674] Iwan-Zietek I, Zietek Z, Sulikowski T, et al. Impact of kidney donor hemostasis on risk of complications after transplantation - preliminary outcomes [J]. Medical Science Monitor, 2013,19: 1102-1108.

[675] Izumikawa T, Sakamoto S, Takeshita S, et al. Outcomes of primary percutaneous coronary intervention for acute myocardial infarction with unprotected left main coronary artery occlusion [J]. Catheterization and Cardiovascular Interventions, 2012,79(7): 1111-1116.

[676] Jackowska T, Zaleska-Ponganis J, Dziurda D. Invasive Pneumococcal Bacteremia in a 9-Year-Old Boy Caused by Serotype 1: Course, Treatment and Costs[A]. In: Respiratory Regulation - Clinical Advances (Pokorski M, ed), Vol. 755, 2013: 257-266.

[677] Jalkanen V, Yang RK, Linko R, et al. SuPAR and PAI-1 in critically ill, mechanically ventilated patients [J]. Intensive Care Medicine, 2013,39(3): 489-496.

[678] Jamal W, Al Roomi E, AbdulAziz LR, et al. Evaluation of Curetis Unyvero, a Multiplex PCR-Based Testing System, for Rapid Detection of Bacteria and Antibiotic Resistance and Impact of the Assay on Management of Severe Nosocomial Pneumonia [J]. Journal of Clinical Microbiology, 2014,52(7): 2487-2492.

[679] Jaradat ZW, Al Mousa W, Elbetieha A, et al. Cronobacter spp. - opportunistic food-borne pathogens. A review of their virulence and environmental-adaptive traits [J]. Journal of Medical Microbiology, 2014,63: 1023-1037.

[680] Jennewein C, Tran N, Paulus P, et al. Novel Aspects of Fibrin(ogen) Fragments during Inflammation [J]. Molecular Medicine, 2011,17(5-6): 568-573.

[681] Ji SC, Pan YT, Lu QY, et al. Screening of differentially expressed genes between multiple trauma patients with and without sepsis [J]. Genetics and Molecular Research, 2014,13(1): 1855-1864.

[682] Jiang CG, Huang H, Liu J, et al. Fasudil, a Rho-Kinase Inhibitor, Attenuates Bleomycin-Induced Pulmonary Fibrosis in Mice [J]. International Journal of Molecular Sciences, 2012,13(7): 8293-8307.

[683] Jiang QY, Hu Z, Pan XL, et al. Comparative Proteomic Analysis of Wheat (Triticum aestivum L.) Hybrid Necrosis [J]. Journal of Integrative Agriculture, 2013,12(3): 387-397.

[684] Jin YS, Sun CW, Zhao JH, et al. Optimization of loading pressure waveforms for piston driven isentropic compression [J]. Journal of Applied Physics, 2014,115(24).

[685] Jo SR, Kallo I, Bardoczi Z, et al. Neuronal Hypoxia Induces Hsp40-Mediated Nuclear Import of Type 3 Deiodinase As an Adaptive Mechanism to Reduce Cellular Metabolism [J]. Journal of Neuroscience, 2012,32(25): 8491-8500.

[686] Johansson PI, Haase N, Perner A, et al. Association between sympathoadrenal activation, fibrinolysis, and endothelial damage in septic patients: A prospective study [J]. Journal of Critical Care, 2014,29(3): 327-333.

[687] Johansson PI, Sorensen AM, Perner A, et al. High sCD40L levels early after trauma are associated with enhanced shock, sympathoadrenal activation, tissue and endothelial damage, coagulopathy and mortality [J]. Journal of Thrombosis and Haemostasis, 2012,10(2): 207-216.

[688] Johansson PI, Stensballe J, Rasmussen LS, et al. High circulating adrenaline levels at admission predict increased mortality after trauma [J]. Journal of Trauma and Acute Care Surgery, 2012,72(2): 428-436.

[689] Johnson MM, David JA, Michelhaugh SK, et al. Commentary on: Johnson MM, David JA, Michelhaugh SK, Schmidt CJ, Bannon MJ. Increased heat shock protein 70 gene expression in the brains of cocaine-related fatalities may be reflective of postdrug survival and intervention rather than excited delirium. J Forensic Sci 2012;57(6):1519-23. Authors' Response [J]. Journal of Forensic Sciences, 2013,58(2): 562-562.

[690] Jones EM, Albright KC, Fossati-Bellani M, et al. Emergency Department Shift Change Is Associated With Pneumonia in Patients With Acute Ischemic Stroke [J]. Stroke, 2011,42(11): 3226-3230.

[691] Jonkam C, Zhu Y, Jacob S, et al. Assessment of Combined Muscarinic Antagonist and Fibrinolytic Therapy for Inhalation Injury [J]. Journal of Burn Care & Research, 2012,33(4): 524-531.

[692] Juarez-Herrera U, Jerjes-Sanchez C, Investigators RI. Risk Factors, Therapeutic Approaches, and In-Hospital Outcomes in Mexicans With ST-Elevation Acute Myocardial Infarction: The RENASICA II Multicenter Registry [J]. Clinical Cardiology, 2013,36(5): 241-248.

[693] Kadioglu A, De Filippo K, Bangert M, et al. The Integrins Mac-1 and alpha(4)beta(1) Perform Crucial Roles in Neutrophil and T Cell Recruitment to Lungs during Streptococcus pneumoniae Infection [J]. Journal of Immunology, 2011,186(10): 5907-5915.

[694] Kager LM, van der Poll T, Wiersinga WJ. The coagulation system in melioidosis: from pathogenesis to new treatment strategies [J]. Expert Review of Anti-Infective Therapy, 2014,12(8): 993-1002.

[695] Kager LM, van der Windt GJW, Wieland CW, et al. Plasminogen activator inhibitor type I may contribute to transient, non-specific changes in immunity in the subacute phase of murine tuberculosis [J]. Microbes and Infection, 2012,14(9): 748-755.

[696] Kager LM, Weehuizen TA, Wiersinga WJ, et al. Endogenous alpha(2)-Antiplasmin Is Protective during Severe Gram-Negative Sepsis (Melioidosis) [J]. American Journal of Respiratory and Critical Care Medicine, 2013,188(8): 967-975.

[697] Kager LM, Wiersinga WJ, Roelofs J, et al. Plasminogen activator inhibitor type I contributes to protective immunity during experimental Gram-negative sepsis (melioidosis) [J]. Journal of Thrombosis and Haemostasis, 2011,9(10): 2020-2028.

[698] Kager LM, Wiersinga WJ, Roelofs J, et al. Endogenous tissue-type plasminogen activator impairs host defense during severe experimental gram-negative sepsis (melioidosis) [J]. Critical Care Medicine, 2012,40(7): 2168-2175.

[699] Kager LM, Wiersinga WJ, Roelofs JJ, et al. Plasminogen activator inhibitor type I contributes to protective immunity during experimental gram-negative sepsis (melioidosis) [J]. Journal of Thrombosis and Haemostasis, 2011,9: 295-295.

[700] Kamouh A, John R, Eckman P. Successful Treatment of Early Thrombosis of HeartWare Left Ventricular Assist Device With Intraventricular Thrombolytics [J]. Annals of Thoracic Surgery, 2012,94(1): 281-283.

[701] Kanse SM, Etscheid M. Factor VII activating protease Single nucleotide polymorphisms light the way [J]. Hamostaseologie, 2011,31(3): 174-178.

[702] Kapan M, Gumus M, Onder A, et al. The effects of ellagic acid on the liver and remote organs' oxidative stress and structure after hepatic ischemia reperfusion injury caused by pringle maneuver in rats [J]. Bratislava Medical Journal-Bratislavske Lekarske Listy, 2012,113(5): 274-281.

[703] Kaplan AP, Joseph K. Pathogenic Mechanisms of Bradykinin Mediated Diseases: Dysregulation of an Innate Inflammatory Pathway[A]. In: Advances in Immunology, Vol 121 (Alt FW, ed), Vol. 121, 2014: 41-89.

[704] Kar S, Webel R. Septic Thrombophlebitis: Percutaneous Mechanical Thrombectomy and Thrombolytic Therapies [J]. American Journal of Therapeutics, 2014,21(2): 131-136.

[705] Karabiyik A, Yilmaz E, Gulec S, et al. The Dual Diverse Dynamic Reversible Effects of Ankaferd Blood Stopper on EPCR and PAT-1 Inside Vascular Endothelial Cells With and Without LPS Challenge [J]. Turkish Journal of Hematology, 2012,29(4): 361-366.

[706] Karandashova S, Florova G, Azghani AO, et al. Intrapleural Adenoviral Delivery of Human Plasminogen Activator Inhibitor-1 Exacerbates Tetracycline-Induced Pleural Injury in Rabbits [J]. American Journal of Respiratory Cell and Molecular Biology, 2013,48(1): 44-52.

[707] Karlisch C, Harati K, Chromik AM, et al. Effects of TRAIL and taurolidine on apoptosis and proliferation in human rhabdomyosarcoma, leiomyosarcoma and epithelioid cell sarcoma [J]. International Journal of Oncology, 2013,42(3): 945-956.

[708] Katoh S, Honda S, Watanabe T, et al. Atrial endothelial impairment through Toll-like receptor 4 signaling causes atrial thrombogenesis [J]. Heart and Vessels, 2014,29(2): 263-272.

[709] Katre A, Ballinger C, Akhter H, et al. Increased transforming growth factor beta 1 expression mediates ozone-induced airway fibrosis in mice [J]. Inhalation Toxicology, 2011,23(8): 486-494.

[710] Kau JH, Shih YL, Lien TS, et al. Activated protein C ameliorates Bacillus anthracis lethal toxin-induced lethal pathogenesis in rats [J]. Journal of Biomedical Science, 2012,19.

[711] Kawasugi K, Wada H, Hatada T, et al. Prospective evaluation of hemostatic abnormalities in overt DIC due to various underlying diseases [J]. Thrombosis Research, 2011,128(2): 186-190.

[712] Kaya S, Koksal I, Mentese A, et al. The significance of serum urokinase plasminogen activation receptor (suPAR) in the diagnosis and follow-up of febrile neutropenic patients with hematologic malignancies [J]. International Journal of Infectious Diseases, 2013,17(11): E1056-E1059.

[713] Kaymakci A, Guven S, Ciftci I, et al. Protective effects of growth hormone on bacterial translocation and intestinal damage in rats with partial intestinal obstruction [J]. Bratislava Medical Journal-Bratislavske Lekarske Listy, 2014,115(7): 395-399.

[714] Kikkert WJ, van Geloven N, van der Laan MH, et al. The Prognostic Value of Bleeding Academic Research Consortium (BARC)-Defined Bleeding Complications in ST-Segment Elevation Myocardial Infarction A Comparison With the TIMI (Thrombolysis In Myocardial Infarction), GUSTO (Global Utilization of Streptokinase and Tissue Plasminogen Activator for Occluded Coronary Arteries), and ISTH (International Society on Thrombosis and Haemostasis) Bleeding Classifications [J]. Journal of the American College of Cardiology, 2014,63(18): 1866-1875.

[715] Kim JH, Jeong SJ, Kim B, et al. Melatonin synergistically enhances cisplatin-induced apoptosis via the dephosphorylation of ERK/p90 ribosomal S6 kinase/heat shock protein 27 in SK-OV-3 cells [J]. Journal of Pineal Research, 2012,52(2): 244-252.

[716] Kim S, Kim J, Kim B, et al. Melatonin synergistically enhances cisplatin-induced apoptosis via the dephosphorylation of ERK/p90 ribosomal S6 kinase/heat shock protein 27 in SK-OV-3 cells [J]. Clinical Cancer Research, 2013,19.

[717] Kline JA, Hernandez J, Hogg MM, et al. Rationale and methodology for a multicentre randomised trial of fibrinolysis for pulmonary embolism that includes quality of life outcomes [J]. Emergency Medicine Australasia, 2013,25(6): 515-526.

[718] Kline JA, Nordenholz KE, Courtney DM, et al. Treatment of submassive pulmonary embolism with tenecteplase or placebo: cardiopulmonary outcomes at 3 months: multicenter double-blind, placebo-controlled randomized trial [J]. Journal of Thrombosis and Haemostasis, 2014,12(4): 459-468.

[719] Koch A, Tacke F. Risk stratification and triage in the emergency department: has this become 'suPAR' easy? [J]. Journal of Internal Medicine, 2012,272(3): 243-246.

[720] Koch A, Zimmermann HW, Gassler N, et al. Clinical relevance and cellular source of elevated soluble urokinase plasminogen activator receptor (suPAR) in acute liver failure [J]. Liver International, 2014,34(9): 1330-1339.

[721] Koh G, Meijers JCM, Maude RR, et al. Diabetes does not influence activation of coagulation, fibrinolysis or anticoagulant pathways in Gram-negative sepsis (melioidosis) [J]. Thrombosis and Haemostasis, 2011,106(6): 1139-1148.

[722] Komissarov AA, Florova G, Azghani A, et al. Active alpha-macroglobulin is a reservoir for urokinase after fibrinolytic therapy in rabbits with tetracycline-induced pleural injury and in human pleural fluids [J]. American Journal of Physiology-Lung Cellular and Molecular Physiology, 2013,305(10): L682-L692.

[723] Komissarov AA, Florova G, Idell S. Effects of Extracellular DNA on Plasminogen Activation and Fibrinolysis [J]. Journal of Biological Chemistry, 2011,286(49): 41949-41962.

[724] Komissarov AA, Stankowska D, Krupa A, et al. Novel aspects of urokinase function in the injured lung: role of alpha(2)-macroglobulin [J]. American Journal of Physiology-Lung Cellular and Molecular Physiology, 2012,303(12): L1037-L1045.

[725] Konstantinides SV, Meyer G, Steering C, et al. Single-bolus tenecteplase plus heparin compared with heparin alone for normotensive patients with acute pulmonary embolism who have evidence of right ventricular dysfunction and myocardial injury: Rationale and design of the Pulmonary Embolism Thrombolysis (PEITHO) trial [J]. American Heart Journal, 2012,163(1): 33-U51.

[726] Koyama K, Madoiwa S, Nunomiya S, et al. Combination of thrombin-antithrombin complex, plasminogen activator inhibitor-1, and protein C activity for early identification of severe coagulopathy in initial phase of sepsis: a prospective observational study [J]. Critical Care, 2014,18(1).

[727] Koyama K, Madoiwa S, Tanaka S, et al. Evaluation of hemostatic biomarker abnormalities that precede platelet count decline in critically ill patients with sepsis [J]. Journal of Critical Care, 2013,28(5): 556-563.

[728] Krause U, Schneider HE, Webel M, et al. Thrombosis of the Aorta Abdominalis in Infants - Diagnosis and Thrombolytic Therapy [J]. Klinische Padiatrie, 2012,224(3): 179-182.

[729] Kudlicka J, Mlcek M, Hala P, et al. Pig Model of Pulmonary Embolism: Where Is the Hemodynamic Break Point? [J]. Physiological Research, 2013,62: S173-S179.

[730] Kuiper JW, Groeneveld ABJ, Haitsma JJ, et al. Injurious mechanical ventilation causes kidney apoptosis and dysfunction during sepsis but not after intra-tracheal acid instillation: an experimental study [J]. Bmc Nephrology, 2014,15.

[731] Kung CW, Lee YM, Yen MH. In vivo anticoagulant effect of ethyl pyruvate in endotoxemic rats [J]. Thrombosis Research, 2011,127(6): 582-588.

[732] Kuntz M, Mysiorek C, Petrault O, et al. Transient oxygen-glucose deprivation sensitizes brain capillary endothelial cells to rtPA at 4 h of reoxygenation [J]. Microvascular Research, 2014,91: 44-57.

[733] Kuo WT. Endovascular Therapy for Acute Pulmonary Embolism [J]. Journal of Vascular and Interventional Radiology, 2012,23(2): 167-179.

[734] Kutcher ME, Xu J, Vilardi RF, et al. Extracellular histone release in response to traumatic injury: Implications for a compensatory role of activated protein C [J]. Journal of Trauma and Acute Care Surgery, 2012,73(6): 1389-1394.

[735] Kwiecinski J, Jacobsson G, Karlsson M, et al. Staphylokinase Promotes the Establishment of Staphylococcus aureus Skin Infections While Decreasing Disease Severity [J]. Journal of Infectious Diseases, 2013,208(6): 990-999.

[736] Lacroix R, Souab KH, Cointe S, et al. Microparticle-dependent plasmin generation predicts the outcome of septic shock patients [J]. Journal of Thrombosis and Haemostasis, 2013,11: 124-125.

[737] Laine O, Joutsi-Korhonen L, Makela S, et al. Polymorphisms of PAI-1 and platelet GP Ia may associate with impairment of renal function and thrombocytopenia in Puumala hantavirus infection [J]. Thrombosis Research, 2012,129(5): 611-615.

[738] Langsdorf EF, Mao X, Chang SL. A role for reactive oxygen species in endotoxin-induced elevation of MOR expression in the nervous and immune systems [J]. Journal of Neuroimmunology, 2011,236(1-2): 57-64.

[739] Lankeit M, Konstantinides S. Thrombolytic therapy for submassive pulmonary embolism [J]. Best Practice & Research Clinical Haematology, 2012,25(3): 379-389.

[740] Lawrence PK, Rokbi B, Arnaud-Barbe N, et al. CD4 T Cell Antigens from Staphylococcus aureus Newman Strain Identified following Immunization with Heat-Killed Bacteria [J]. Clinical and Vaccine Immunology, 2012,19(4): 477-489.

[741] Le Mee A, Mordacq C, Lagree M, et al. Survey of hospital procedures for parapneumonic effusion in children highlights need for standardised management [J]. Acta Paediatrica, 2014,103(9): E393-E398.

[742] Leach HG, Chrobak I, Han R, et al. Endothelial Cells Recruit Macrophages and Contribute to a Fibrotic Milieu in Bleomycin Lung Injury [J]. American Journal of Respiratory Cell and Molecular Biology, 2013,49(6): 1093-1101.

[743] Lee HK, Kwak HS, Chung GH, et al. Balloon-Expandable Stent Placement in Patients with Immediate Reocclusion after Initial Successful Thrombolysis of Acute Middle Cerebral Arterial Obstruction [J]. Interventional Neuroradiology, 2012,18(1): 80-88.

[744] Lee JH, Park S, Cheon S, et al. 1,25-Dihydroxyvitamin D-3 enhances NK susceptibility of human melanoma cells via Hsp60-mediated FAS expression [J]. European Journal of Immunology, 2011,41(10): 2937-2946.

[745] Lee SJ, Kim JH, Na CY, et al. Eleven years of experience with the neurologic complications in Korean patients with acute aortic dissection: a retrospective study [J]. Bmc Neurology, 2013,13.

[746] Lee WY, Chen YC, Shih CM, et al. The induction of heme oxygenase-1 suppresses heat shock protein 90 and the proliferation of human breast cancer cells through its byproduct carbon monoxide [J]. Toxicology and Applied Pharmacology, 2014,274(1): 55-62.

[747] Lerschmacher O, Koch A, Streetz K, et al. Management of decompensated liver cirrhosis in the intensive care unit [J]. Medizinische Klinik-Intensivmedizin Und Notfallmedizin, 2013,108(8): 646-656.

[748] Lesault PF, Theret M, Magnan M, et al. Macrophages Improve Survival, Proliferation and Migration of Engrafted Myogenic Precursor Cells into MDX Skeletal Muscle [J]. Plos One, 2012,7(10).

[749] Levi M. Diagnosis and treatment of disseminated intravascular coagulation [J]. International Journal of Laboratory Hematology, 2014,36(3): 228-236.

[750] Li DB, Hua Q, Li HW, et al. Effects of early angioplasty after fibrinolysis on prognosis of patients with ST-segment elevation acute myocardial infarction [J]. African Journal of Biotechnology, 2011,10(70): 15801-15804.

[751] Li L, Nie W, Zhou HF, et al. Association between Plasminogen Activator Inhibitor-1-675 4G/5G Polymorphism and Sepsis: A Meta-Analysis [J]. Plos One, 2013,8(1).

[752] Li LF, Kao KC, Yang CT, et al. Ethyl pyruvate reduces ventilation-induced neutrophil infiltration and oxidative stress [J]. Experimental Biology and Medicine, 2012,237(6): 720-727.

[753] Li LF, Liu YY, Kao KC, et al. Mechanical ventilation augments bleomycin-induced epithelial- mesenchymal transition through the Src pathway [J]. Laboratory Investigation, 2014,94(9): 1017-1029.

[754] Li PC, Yang CC, Hsu SP, et al. Repetitive progressive thermal preconditioning hinders thrombosis by reinforcing phosphatidylinositol 3-kinase/Akt-dependent heat-shock protein/endothelial nitric oxide synthase signaling [J]. Journal of Vascular Surgery, 2012,56(1): 159-170.

[755] Lim JH, Woo CH, Li JD. Critical role of type 1 plasminogen activator inhibitor (PAI-1) in early host defense against nontypeable Haemophilus influenzae (NTHi) infection [J]. Biochemical and Biophysical Research Communications, 2011,414(1): 67-72.

[756] Lin CH, Lin JT, Chen CH, et al. Ionospheric shock waves triggered by rockets [J]. Annales Geophysicae, 2014,32(9): 1145-1152.

[757] Lin YL, Peng NN, Zhuang HQ, et al. Heat shock proteins HSP70 and MRJ cooperatively regulate cell adhesion and migration through urokinase receptor [J]. Bmc Cancer, 2014,14.

[758] Lisman T, Bakhtiari K, Adelmeijer J, et al. Intact thrombin generation and decreased fibrinolytic capacity in patients with acute liver injury or acute liver failure [J]. Journal of Thrombosis and Haemostasis, 2012,10(7): 1312-1319.

[759] Lisman T, Leuvenink HGD, Porte RJ, et al. Activation of hemostasis in brain dead organ donors: an observational study [J]. Journal of Thrombosis and Haemostasis, 2011,9(10): 1959-1965.

[760] Litmathe J, Boeken U, Bohlen G, et al. Systemic Inflammatory Response Syndrome After Extracorporeal Circulation: A Predictive Algorithm for the Patient at Risk [J]. Hellenic Journal of Cardiology, 2011,52(6): 493-500.

[761] Liu D, Tan H, Zhang L, et al. Effect of intra-abdominal volume increment on lungs in hemorrhagic shock [J]. Bratislava Medical Journal-Bratislavske Lekarske Listy, 2013,114(8): 425-430.

[762] Liu G, Yang YP, Yang SZ, et al. The Receptor for Urokinase Regulates TLR2 Mediated Inflammatory Responses in Neutrophils [J]. Plos One, 2011,6(10).

[763] Liu RM, Vayalil PK, Ballinger C, et al. Transforming growth factor beta suppresses glutamate-cysteine ligase gene expression and induces oxidative stress in a lung fibrosis model [J]. Free Radical Biology and Medicine, 2012,53(3): 554-563.

[764] Liu YH, Han YL, Song J, et al. Heat shock protein 104 inhibited the fibrillization of prion peptide 106-126 and disassembled prion peptide 106-126 fibrils in vitro [J]. International Journal of Biochemistry & Cell Biology, 2011,43(5): 768-774.

[765] Lonjaret L, Lairez O, Minville V, et al. Pulmonary embolism and pregnancy [J]. Annales Francaises D Anesthesie Et De Reanimation, 2013,32(4): 257-266.

[766] Loonen AJM, de Jager CPC, Tosserams J, et al. Biomarkers and Molecular Analysis to Improve Bloodstream Infection Diagnostics in an Emergency Care Unit [J]. Plos One, 2014,9(1).

[767] Lopes AA, Barreto AC, Maeda NY, et al. Plasma von Willebrand factor as a predictor of survival in pulmonary arterial hypertension associated with congenital heart disease [J]. Brazilian Journal of Medical and Biological Research, 2011,44(12): 1269-1275.

[768] Lorente L, Martin M, Plasencia F, et al. The 372 T/C genetic polymorphism of TIMP-1 is associated with serum levels of TIMP-1 and survival in patients with severe sepsis [J]. Critical Care, 2013,17(3).

[769] Lorente L, Martin MM, Borreguero-Leon JM, et al. Sustained high plasma plasminogen activator inhibitor-1 levels are associated with severity and mortality in septic patients [J]. Thrombosis Research, 2014,134(1): 182-186.

[770] Lorente L, Martin MM, Sole-Violan J, et al. Association of Sepsis-Related Mortality with Early Increase of TIMP-1/MMP-9 Ratio [J]. Plos One, 2014,9(4).

[771] Luo DY, Szaba FM, Kummer LW, et al. Protective Roles for Fibrin, Tissue Factor, Plasminogen Activator Inhibitor-1, and Thrombin Activatable Fibrinolysis Inhibitor, but Not Factor XI, during Defense against the Gram-Negative Bacterium Yersinia enterocolitica [J]. Journal of Immunology, 2011,187(4): 1866-1876.

[772] Lupu C, Herlea O, Tang H, et al. Plasmin-dependent proteolysis of tissue factor pathway inhibitor in a mouse model of endotoxemia [J]. Journal of Thrombosis and Haemostasis, 2013,11(1): 142-148.

[773] Machado GB, de Oliveira AV, Saliba AM, et al. Pseudomonas aeruginosa toxin ExoU induces a PAF-dependent impairment of alveolar fibrin turnover secondary to enhanced activation of coagulation and increased expression of plasminogen activator inhibitor-1 in the course of mice pneumosepsis [J]. Respiratory Research, 2011,12.

[774] Mack EH, Wheeler DS, Hirsch R. Endovascular treatment of near-fatal neonatal superior vena cava syndrome [J]. Pediatric Critical Care Medicine, 2011,12(6): E410-E412.

[775] Mackanos MA, Helms M, Kalish F, et al. Image-guided genomic analysis of tissue response to laser-induced thermal stress [J]. Journal of Biomedical Optics, 2011,16(5).

[776] Madoiwa S, Tanaka H, Nagahama Y, et al. Degradation of cross-linked fibrin by leukocyte elastase as alternative pathway for plasmin-mediated fibrinolysis in sepsis-induced disseminated intravascular coagulation [J]. Journal of Thrombosis and Haemostasis, 2011,9: 424-424.

[777] Maegele M. The coagulopathy of trauma [J]. European Journal of Trauma and Emergency Surgery, 2014,40(2): 113-126.

[778] Maegele M, Schochl H, Cohen MJ. AN UPDATE ON THE COAGULOPATHY OF TRAUMA [J]. Shock, 2014,41: 21-25.

[779] Makarova AM, Lebedeva TV, Nassar T, et al. Urokinase-type Plasminogen Activator (uPA) Induces Pulmonary Microvascular Endothelial Permeability through Low Density Lipoprotein Receptor-related Protein (LRP)-dependent Activation of Endothelial Nitric-oxide Synthase [J]. Journal of Biological Chemistry, 2011,286(26): 23044-23053.

[780] Manaa A, Mimouni H, Wasti S, et al. Comparative proteomic analysis of tomato (Solanum lycopersicum) leaves under salinity stress [J]. Plant Omics, 2013,6(4): 268-277.

[781] Manetti M, Rosa I, Milia AF, et al. Inactivation of urokinase-type plasminogen activator receptor (uPAR) gene induces dermal and pulmonary fibrosis and peripheral microvasculopathy in mice: a new model of experimental scleroderma? [J]. Annals of the Rheumatic Diseases, 2014,73(9): 1700-1709.

[782] Mannova J, Silhart Z, Prokes A, et al. Myocardial injury in patients after an elective abdominal aortic aneurysm repair [J]. Bratislava Medical Journal-Bratislavske Lekarske Listy, 2013,114(5): 269-273.

[783] Marudamuthu AS, Bhandary YP, Shetty SK, et al. Role Of The Urokinase-Fibrinolytic System In Epithelial Mesenchymal Transition During Lung Injury [J]. American Journal of Respiratory and Critical Care Medicine, 2014,189.

[784] Mash DC. Commentary on: Johnson MM, David JA, Michelhaugh SK, Schmidt CJ, Bannon MJ. Increased heat shock protein 70 gene expression in the brains of cocaine-related fatalities may be reflective of postdrug survival and intervention rather than excited delirium. J Forensic Sci 2012;57(6):1519-23 [J]. Journal of Forensic Sciences, 2013,58(2): 559-561.

[785] Mata-Greenwood E, Stewart JM, Steinhorn RH, et al. Role of BCL2-Associated Athanogene 1 in Differential Sensitivity of Human Endothelial Cells to Glucocorticoids [J]. Arteriosclerosis Thrombosis and Vascular Biology, 2013,33(5): 1046-U1464.

[786] Matos JM, Fajardo A, Dalsing MC, et al. Evidence for nonoperative management of acute limb ischemia in infants [J]. Journal of Vascular Surgery, 2012,55(4): 1156-1159.

[787] McArthur JD, Cook SM, Venturini C, et al. The Role of Streptokinase as a Virulence Determinant of Streptococcus pyogenes - Potential for Therapeutic Targeting [J]. Current Drug Targets, 2012,13(3): 297-307.

[788] McKleroy W, Lee TH, Atabai K. Always cleave up your mess: targeting collagen degradation to treat tissue fibrosis [J]. American Journal of Physiology-Lung Cellular and Molecular Physiology, 2013,304(11): L709-L721.

[789] McWilliams RS, Spaulding DK, Eggert JH, et al. Phase Transformations and Metallization of Magnesium Oxide at High Pressure and Temperature [J]. Science, 2012,338(6112): 1330-1333.

[790] Medvedev AB. Wide-range multiphase equation of state for iron [J]. Combustion Explosion and Shock Waves, 2014,50(5): 582-598.

[791] Mercer PF, Chambers RC. Coagulation and coagulation signalling in fibrosis [J]. Biochimica Et Biophysica Acta-Molecular Basis of Disease, 2013,1832(7): 1018-1027.

[792] Midde KK, Batchinsky AI, Cancio LC, et al. WOOD BARK SMOKE INDUCES LUNG AND PLEURAL PLASMINOGEN ACTIVATOR INHIBITOR 1 AND STABILIZES ITS mRNA IN PORCINE LUNG CELLS [J]. Shock, 2011,36(2): 128-137.

[793] Mihinova D, Pieckova E. Moldy buildings, health of their occupantsand fungal prevention [J]. Bratislava Medical Journal-Bratislavske Lekarske Listy, 2012,113(5): 314-318.

[794] Miller AC, Elamin EM, Suffredini AF. Inhaled Anticoagulation Regimens for the Treatment of Smoke Inhalation-Associated Acute Lung Injury: A Systematic Review [J]. Critical Care Medicine, 2014,42(2): 413-419.

[795] Mitaka C, Tomita M. POLYMYXIN B-IMMOBILIZED FIBER COLUMN HEMOPERFUSION THERAPY FOR SEPTIC SHOCK [J]. Shock, 2011,36(4): 332-338.

[796] Molkanen T, Ruotsalainen E, Thorball CW, et al. Elevated soluble urokinase plasminogen activator receptor (suPAR) predicts mortality in Staphylococcus aureus bacteremia [J]. European Journal of Clinical Microbiology & Infectious Diseases, 2011,30(11): 1417-1424.

[797] Moorjani N, Price S. Massive Pulmonary Embolism [J]. Cardiology Clinics, 2013,31(4): 503-+.

[798] Moran J, Sun S, Khababa I, et al. A Randomized Trial Comparing Gentamicin/Citrate and Heparin Locks for Central Venous Catheters in Maintenance Hemodialysis Patients [J]. American Journal of Kidney Diseases, 2012,59(1): 102-107.

[799] Morris ME, Maijub JG, Walker SK, et al. Meningococcal sepsis and purpura fulminans: the surgical perspective [J]. Postgraduate Medical Journal, 2013,89(1052): 340-345.

[800] Moshai EF, Wemeau-Stervinou L, Cigna N, et al. Targeting the Hedgehog-Glioma-Associated Oncogene Homolog Pathway Inhibits Bleomycin-Induced Lung Fibrosis in Mice [J]. American Journal of Respiratory Cell and Molecular Biology, 2014,51(1): 11-25.

[801] Murakami Y, Wada Y, Kobayashi H, et al. Inhibition of Aeromonas sobria serine protease (ASP) by alpha(2)-macroglobulin [J]. Biological Chemistry, 2012,393(10): 1193-1200.

[802] Mussap M, Noto A, Cibecchini F, et al. The importance of biomarkers in neonatology [J]. Seminars in Fetal & Neonatal Medicine, 2013,18(1): 56-64.

[803] Naglova H, Bucova M. HMGB1 and its physiological and pathological roles [J]. Bratislava Medical Journal-Bratislavske Lekarske Listy, 2012,113(3): 163-171.

[804] Nagy V, Bober J, Zavacky P, et al. The recurrent primary retroperitoneal liposarcoma [J]. Bratislava Medical Journal-Bratislavske Lekarske Listy, 2013,114(11): 662-667.

[805] Naik PK, Bozyk PD, Bentley JK, et al. Periostin promotes fibrosis and predicts progression in patients with idiopathic pulmonary fibrosis [J]. American Journal of Physiology-Lung Cellular and Molecular Physiology, 2012,303(12): L1046-L1056.

[806] Nassar T, Yarovoi S, Abu Fanne R, et al. Urokinase Plasminogen Activator Regulates Pulmonary Arterial Contractility and Vascular Permeability in Mice [J]. American Journal of Respiratory Cell and Molecular Biology, 2011,45(5): 1015-1021.

[807] Nassiri N, Jain A, McPhee D, et al. Massive and Submassive Pulmonary Embolism: Experience With an Algorithm for Catheter-Directed Mechanical Thrombectomy [J]. Annals of Vascular Surgery, 2012,26(1): 18-24.

[808] Nederhoed JH, Slikkerveer J, Meyer KW, et al. Contrast-enhanced sonothrombolysis in a porcine model of acute peripheral arterial thrombosis and prevention of anaphylactic shock [J]. Lab Animal, 2014,43(3): 91-94.

[809] Neto-Neves EM, Kiss T, Muhl D, et al. Matrix Metalloproteinases as Drug Targets in Acute Pulmonary Embolism [J]. Current Drug Targets, 2013,14(3): 344-352.

[810] Nishiyama T, Kohno Y, Koishi K. Effects of antithrombin and gabexate mesilate on disseminated intravascular coagulation: a preliminary study [J]. American Journal of Emergency Medicine, 2012,30(7): 1219-1223.

[811] Noel P, Cashen S, Patel B. Trauma-Induced Coagulopathy: From Biology to Therapy [J]. Seminars in Hematology, 2013,50(3): 259-269.

[812] Noori N, Aliabad GM, Mohammadi M, et al. The Effects of Ranitidine and Hydrocortisone on the Complications of Femoral Artery Obstruction Treated by Streptokinase Following Cardiac Catheterization in Pediatric Patients with Congenital Heart Diseases [J]. Iranian Red Crescent Medical Journal, 2013,15(2): 117-121.

[813] Ogawa S, Richardson JE, Sakai T, et al. High mortality associated with intracardiac and intrapulmonary thromboses after cardiopulmonary bypass [J]. Journal of Anesthesia, 2012,26(1): 9-19.

[814] Ohshiro M, Kuroda J, Kobayashi Y, et al. ADAMTS-13 activity can predict the outcome of disseminated intravascular coagulation in hematologic malignancies treated with recombinant human soluble thrombomodulin [J]. American Journal of Hematology, 2012,87(1): 116-119.

[815] Okamura D, Starr ME, Lee EY, et al. Age-dependent vulnerability to experimental acute pancreatitis is associated with increased systemic inflammation and thrombosis [J]. Aging Cell, 2012,11(5): 760-769.

[816] Oktar GL, Kirisci M, Dursun AD, et al. Antioxidative effects of adrenomedullin and vascular endothelial growth factor on lung injury induced by skeletal muscle ischemia-reperfusion [J]. Bratislava Medical Journal-Bratislavske Lekarske Listy, 2013,114(11): 625-628.

[817] Onder AM, Billings AA, Chandar J, et al. Antibiotic lock solutions allow less systemic antibiotic exposure and less catheter malfunction without adversely affecting antimicrobial resistance patterns [J]. Hemodialysis International, 2013,17(1): 75-85.

[818] Orsi FA, Angerami RN, Mazetto BM, et al. Reduced thrombin formation and excessive fibrinolysis are associated with bleeding complications in patients with dengue fever: a case-control study comparing dengue fever patients with and without bleeding manifestations [J]. Bmc Infectious Diseases, 2013,13.

[819] Ortiz-Vazquez IC, Mendoza-Perez BC, Madrid-Miller A, et al. Left main coronary artery aneurysm thrombosis in a young patient with acute myocardial infarction [J]. Cirugia Y Cirujanos, 2013,81(2): 138-142.

[820] Osterholzer JJ, Christensen PJ, Lama V, et al. PAI-1 promotes the accumulation of exudate macrophages and worsens pulmonary fibrosis following type II alveolar epithelial cell injury [J]. Journal of Pathology, 2012,228(2): 170-180.

[821] Ostrowski SR, Berg RMG, Windelov NA, et al. Coagulopathy, catecholamines, and biomarkers of endothelial damage in experimental human endotoxemia and in patients with severe sepsis: A prospective study [J]. Journal of Critical Care, 2013,28(5): 586-596.

[822] Ostrowski SR, Berg RMG, Windelov NA, et al. Discrepant Fibrinolytic Response in Plasma and Whole Blood during Experimental Endotoxemia in Healthy Volunteers [J]. Plos One, 2013,8(3).

[823] Ostrowski SR, Johansson PI. Endothelial glycocalyx degradation induces endogenous heparinization in patients with severe injury and early traumatic coagulopathy [J]. Journal of Trauma and Acute Care Surgery, 2012,73(1): 60-66.

[824] Ostrowski SR, Sorensen AM, Windelov NA, et al. High levels of soluble VEGF receptor 1 early after trauma are associated with shock, sympathoadrenal activation, glycocalyx degradation and inflammation in severely injured patients: a prospective study [J]. Scandinavian Journal of Trauma Resuscitation & Emergency Medicine, 2012,20.

[825] Otto M. Staphylococcus aureus toxins [J]. Current Opinion in Microbiology, 2014,17: 32-37.

[826] Palmieri EA, Migliaresi P, Palmieri V, et al. Lytic failure in the current pharmacointensive ST-elevated acute myocardial infarction care: insights from a pilot real-world study [J]. Journal of Cardiovascular Medicine, 2013,14(1): 35-42.

[827] Paraskakis E, Vergadi E, Chatzimichael A, et al. Current evidence for the management of paediatric parapneumonic effusions [J]. Current Medical Research and Opinion, 2012,28(7): 1179-1192.

[828] Parra ER, Aguiar AC, Silva LO, et al. Morphometric evaluation of nitric oxide synthase isoforms and their cytokine regulators predict pulmonary dysfunction and survival in systemic sclerosis [J]. Brazilian Journal of Medical and Biological Research, 2013,46(10): 881-891.

[829] Patel VN, Gupta R, Horn CM, et al. The Neuro-Critical Care Management of the Endovascular Stroke Patient [J]. Current Treatment Options in Neurology, 2013,15(2): 113-124.

[830] Paudel N, Sadagopan S, Balasubramanian S, et al. Kaposi's Sarcoma-Associated Herpesvirus Latency-Associated Nuclear Antigen and Angiogenin Interact with Common Host Proteins, Including Annexin A2, Which Is Essential for Survival of Latently Infected Cells [J]. Journal of Virology, 2012,86(3): 1589-1607.

[831] Paulus P, Jennewein C, Zacharowski K. Biomarkers of endothelial dysfunction: can they help us deciphering systemic inflammation and sepsis? [J]. Biomarkers, 2011,16: S11-S21.

[832] Pavlikova N, Bartonova I, Dincakova L, et al. Differentially expressed proteins in human breast cancer cells sensitive and resistant to paclitaxel [J]. International Journal of Oncology, 2014,45(2): 822-830.

[833] Pillarisetti J, Gupta K. Massive Pulmonary Embolism With Shock: Role of Thrombolysis Using Central Venous Access [J]. Journal of Invasive Cardiology, 2012,24(12): E321-E324.

[834] Pineau N. Molecular Dynamics Simulations of Shock Compressed Graphite [J]. Journal of Physical Chemistry C, 2013,117(24): 12778-12786.

[835] Pinheiro C, Guerra-Guimaraes L, David TS, et al. Proteomics: State of the art to study Mediterranean woody species under stress [J]. Environmental and Experimental Botany, 2014,103: 117-127.

[836] Pini M, Castellanos KJ, Rhodes DH, et al. Obesity and IL-6 interact in modulating the response to endotoxemia in mice [J]. Cytokine, 2013,61(1): 71-77.

[837] Polat E, Ozogul YB, Ercan M, et al. Management of hepatic artery aneurysms [J]. Bratislava Medical Journal-Bratislavske Lekarske Listy, 2012,113(11): 676-679.

[838] Porta CR, Nelson D, McVay D, et al. The effects of tranexamic acid and prothrombin complex concentrate on the coagulopathy of trauma: An in vitro analysis of the impact of severe acidosis [J]. Journal of Trauma and Acute Care Surgery, 2013,75(6): 954-960.

[839] Power A, Hill P, Singh SK, et al. Comparison of Tesio and LifeCath twin permanent hemodialysis catheters: the VyTes randomized trial [J]. Journal of Vascular Access, 2014,15(2): 108-115.

[840] Preziuso S, Pinho MD, Attili AR, et al. PCR based differentiation between Streptococcus dysgalactiae subsp equisimilis strains isolated from humans and horses [J]. Comparative Immunology Microbiology and Infectious Diseases, 2014,37(3): 169-172.

[841] Qiu P, Li Y, Shiloach J, et al. Bacillus anthracis Cell Wall Peptidoglycan but Not Lethal or Edema Toxins Produces Changes Consistent With Disseminated Intravascular Coagulation in a Rat Model [J]. Journal of Infectious Diseases, 2013,208(6): 978-989.

[842] Raeven P, Drechsler S, Weixelbaumer KM, et al. Systemic inhibition and liver-specific over-expression of PAI-1 failed to improve survival in all-inclusive populations or homogenous cohorts of CLP mice [J]. Journal of Thrombosis and Haemostasis, 2014,12(6): 958-969.

[843] Raeven P, Feichtinger GA, Weixelbaumer KM, et al. Compartment-specific expression of plasminogen activator inhibitor-1 correlates with severity/outcome of murine polymicrobial sepsis [J]. Thrombosis Research, 2012,129(5): E238-E245.

[844] Raeven P, Salibasic A, Drechsler S, et al. A Non-Lethal Traumatic/Hemorrhagic Insult Strongly Modulates the Compartment-Specific PAI-1 Response in the Subsequent Polymicrobial Sepsis [J]. Plos One, 2013,8(2).

[845] Rahman NM. Intrapleural agents for pleural infection: fibrinolytics and beyond [J]. Current Opinion in Pulmonary Medicine, 2012,18(4): 326-332.

[846] Ramos MV, Viana CA, Silva AFB, et al. Proteins derived from latex of C. procera maintain coagulation homeostasis in septic mice and exhibit thrombin- and plasmin-like activities [J]. Naunyn-Schmiedebergs Archives of Pharmacology, 2012,385(5): 455-463.

[847] Rancourt RC, Veress LA, Ahmad A, et al. Tissue factor pathway inhibitor prevents airway obstruction, respiratory failure and death due to sulfur mustard analog inhalation [J]. Toxicology and Applied Pharmacology, 2013,272(1): 86-95.

[848] Rehberg S, Yamamoto Y, Sousse LE, et al. Advantages and pitfalls of combining intravenous antithrombin with nebulized heparin and tissue plasminogen activator in acute respiratory distress syndrome [J]. Journal of Trauma and Acute Care Surgery, 2014,76(1): 126-133.

[849] Rein-Smith CM, Church FC. Emerging pathophysiological roles for fibrinolysis [J]. Current Opinion in Hematology, 2014,21(5): 438-444.

[850] Ren YS, Cui F, Lei YQ, et al. High-Altitude Pulmonary Edema Is Associated With Coagulation and Fibrinolytic Abnormalities [J]. American Journal of the Medical Sciences, 2012,344(3): 186-189.

[851] Rendle DI, Armstrong SK, Hughes KJ. Combination fibrinolytic therapy in the treatment of chronic septic pleuropneumonia in a Thoroughbred gelding [J]. Australian Veterinary Journal, 2012,90(9): 358-362.

[852] Robertson CS, Cherian L, Shah M, et al. Neuroprotection with an Erythropoietin Mimetic Peptide (pHBSP) in a Model of Mild Traumatic Brain Injury Complicated by Hemorrhagic Shock [J]. Journal of Neurotrauma, 2012,29(6): 1156-1166.

[853] Rocco A, Fam G, Sykora M, et al. Poststroke infections are an independent risk factor for poor functional outcome after three-months in thrombolysed stroke patients [J]. International Journal of Stroke, 2013,8(8): 639-644.

[854] Rothenberg SS. The Role of Thoracoscopic Decortication in the Treatment of Childhood Empyema [J]. Pediatric Allergy Immunology and Pulmonology, 2012,25(3): 155-158.

[855] Rowan CM, Miller KE, Beardsley AL, et al. Alteplase Use for Malfunctioning Central Venous Catheters Correlates With Catheter-Associated Bloodstream Infections [J]. Pediatric Critical Care Medicine, 2013,14(3): 306-309.

[856] Russo JJ, Goodman SG, Cantor WJ, et al. Efficacy and safety of a routine early invasive strategy after fibrinolysis stratified by glycoprotein IIb/IIIa inhibitor use during percutaneous coronary intervention: a pre-specified subgroup analysis of the TRANSFER-AMI randomised controlled trial [J]. Heart, 2014,100(11): 873-880.

[857] Rygg JR, Eggert JH, Lazicki AE, et al. Powder diffraction from solids in the terapascal regime [J]. Review of Scientific Instruments, 2012,83(11).

[858] Saggini A, Anogeianaki A, Angelucci D, et al. CHOLESTEROL: AN INFLAMMATORY COMPOUND [J]. European Journal of Inflammation, 2011,9(3): 209-217.

[859] Salgado-Pabon W, Herrera A, Vu BG, et al. Staphylococcus aureus beta-toxin Production is Common in Strains With the beta-toxin Gene Inactivated by Bacteriophage [J]. Journal of Infectious Diseases, 2014,210(5): 784-792.

[860] Samet A, Sledzinska A, Krawczyk B, et al. Leukemia and risk of recurrent Escherichia coli bacteremia: genotyping implicates E-coli translocation from the colon to the bloodstream [J]. European Journal of Clinical Microbiology & Infectious Diseases, 2013,32(11): 1393-1400.

[861] Sanches LC, Azevedo LCP, Salomao R, et al. Association between early glycemic control and improvements in markers of coagulation and fibrinolysis in patients with septic shock-induced stress hyperglycemia [J]. Journal of Critical Care, 2014,29(5).

[862] Sandquist M, Wong HR. Biomarkers of sepsis and their potential value in diagnosis, prognosis and treatment [J]. Expert Review of Clinical Immunology, 2014,10(10): 1349-1356.

[863] Santangelo S, Scarlata S, Zito A, et al. Genetic background of idiopathic pulmonary fibrosis [J]. Expert Review of Molecular Diagnostics, 2013,13(4): 389-406.

[864] Saposnik G, Kapral MK, Cote R, et al. Is pre-existing dementia an independent predictor of outcome after stroke? A propensity score-matched analysis [J]. Journal of Neurology, 2012,259(11): 2366-2375.

[865] Sapru A, Zaroff JG, Pawlikowska L, et al. The 4G/4G Genotype of the PAI-1 (Serpine-1) 4G/5G Polymorphism Is Associated With Decreased Lung Allograft Utilization [J]. American Journal of Transplantation, 2012,12(7): 1848-1854.

[866] Savva A, Raftogiannis M, Baziaka F, et al. Soluble urokinase plasminogen activator receptor (suPAR) for assessment of disease severity in ventilator-associated pneumonia and sepsis [J]. Journal of Infection, 2011,63(5): 344-350.

[867] Saygi S, Alioglu E, Karabulut MN, et al. A Floating Thrombus in Sinus of Valsalva Complicated with Cardiogenic Shock in a Patient with Plasminogen Activator Inhibitor 1 4G/5G Polymorphism [J]. Echocardiography-a Journal of Cardiovascular Ultrasound and Allied Techniques, 2011,28(8): E164-E167.

[868] Scarci M, Zahid I, Bille A, et al. Is video-assisted thoracoscopic surgery the best treatment for paediatric pleural empyema? [J]. Interactive Cardiovascular and Thoracic Surgery, 2011,13(1): 70-76.

[869] Schmid D, Svoboda M, Sorgner A, et al. Glibenclamide reduces proinflammatory cytokines in an ex vivo model of human endotoxinaemia under hypoxaemic conditions [J]. Life Sciences, 2011,89(19-20): 725-734.

[870] Schochl H, Voelckel W, Maegele M, et al. Trauma-associated hyperfibrinolysis [J]. Hamostaseologie, 2012,32(1): 22-27.

[871] Schoot RA, van Dalen EC, van Ommen CH, et al. Antibiotic and other lock treatments for tunnelled central venous catheter-related infections in children with cancer [J]. Cochrane Database of Systematic Reviews, 2013, (6).

[872] Schouten M, van 't Veer C, Roelofs J, et al. Recombinant activated protein C attenuates coagulopathy and inflammation when administered early in murine pneumococcal pneumonia [J]. Thrombosis and Haemostasis, 2011,106(6): 1189-1196.

[873] Schouten M, van't Veer C, Levi M, et al. Endogenous protein C inhibits activation of coagulation and transiently lowers bacterial outgrowth in murine Escherichia coli peritonitis [J]. Journal of Thrombosis and Haemostasis, 2011,9(5): 1072-1075.

[874] Schuetz P, Jones AE, Aird WC, et al. ENDOTHELIAL CELL ACTIVATION IN EMERGENCY DEPARTMENT PATIENTS WITH SEPSIS-RELATED AND NON-SEPSIS-RELATED HYPOTENSION [J]. Shock, 2011,36(2): 104-108.

[875] Schulzke SM, Kaempfen S, Patole SK. Pentoxifylline for the prevention of bronchopulmonary dysplasia in preterm infants [J]. Cochrane Database of Systematic Reviews, 2014, (11).

[876] Sebag SC, Bastarache JA, Ware LB. Therapeutic Modulation of Coagulation and Fibrinolysis in Acute Lung Injury and the Acute Respiratory Distress Syndrome [J]. Current Pharmaceutical Biotechnology, 2011,12(9): 1481-1496.

[877] Seki Y, Wada H, Kawasugi K, et al. A Prospective Analysis of Disseminated Intravascular Coagulation in Patients with Infections [J]. Internal Medicine, 2013,52(17): 1893-1898.

[878] Semeraro N, Ammollo CT, Semeraro F, et al. Sepsis, thrombosis and organ dysfunction [J]. Thrombosis Research, 2012,129(3): 290-295.

[879] Sen HS, Abakay O, Sezgi C, et al. AN OVERVIEW OF THROMBOLYTIC TREATMENT FOR PULMONARY EMBOLISM: A SINGLE CENTRE EXPERIENCE [J]. Acta Medica Mediterranea, 2014,30(2): 403-410.

[880] Serpe L, Canaparo R, Zara GP, et al. 5-AMINOLEVULINIC ACID AND HIGH ENERGY SHOCK WAVES FOR SONODYNAMIC THERAPY: EFFECTS ON SK-N-BE AND SH-SY5Y NEUROBLATOMA CELL LINES [J]. Basic & Clinical Pharmacology & Toxicology, 2011,109: 93-93.

[881] Shaghaghi Z, Bonyadi M, Somi MH, et al. Association of plasminogen activator inhibitor-1 gene polymorphism with inflammatory bowel disease in Iranian Azeri Turkish patients [J]. Saudi Journal of Gastroenterology, 2014,20(1): 54-58.

[882] Shah RJ, Bellamy SL, Localio AR, et al. A panel of lung injury biomarkers enhances the definition of primary graft dysfunction (PGD) after lung transplantation [J]. Journal of Heart and Lung Transplantation, 2012,31(9): 942-949.

[883] Shah SS, Hall M, Newland JG, et al. Comparative Effectiveness of Pleural Drainage Procedures for the Treatment of Complicated Pneumonia in Childhood [J]. Journal of Hospital Medicine, 2011,6(5): 256-263.

[884] Sharmin SA, Alam I, Rahman MA, et al. Mapping the leaf proteome of Miscanthus sinensis and its application to the identification of heat-responsive proteins [J]. Planta, 2013,238(3): 459-474.

[885] Shawyer AC, Amaral J, Langer JC. The role of tissue plasminogen activator in the management of complex intra-abdominal abscesses in children [J]. Journal of Pediatric Surgery, 2012,47(7): 1380-1384.

[886] Shen HN, Lu CL, Li CY. Epidemiology of pleural infections in Taiwan from 1997 through 2008 [J]. Respirology, 2012,17(7): 1086-1093.

[887] Shen LH, Wan F, Shen L, et al. Pharmacoinvasive therapy for ST elevation myocardial infarction in China: a pilot study [J]. Journal of Thrombosis and Thrombolysis, 2012,33(1): 101-108.

[888] Shen Z, He WQ, Liu D, et al. Novel technique for the treatment of large subcapsular renal hematoma: combined use of percutaneous drainage and urokinase injection [J]. International Urology and Nephrology, 2014,46(9): 1751-1755.

[889] Shen ZG, Guo JL, Li DS. Screening of differentially expressed genes related to severe sepsis induced by multiple trauma with DNA microarray [J]. European Review for Medical and Pharmacological Sciences, 2014,18(5): 734-739.

[890] Shetty S. Regulation Of Lung Injury And Fibrosis By P53-Mediated Changes In Urokinase And Plasminogen Activator Inhibitor-1 [J]. American Journal of Respiratory and Critical Care Medicine, 2013,187.

[891] Shigemori K, Hironaka Y, Nagatomo H, et al. Extremely high-pressure generation and compression with laser implosion plasmas [J]. Applied Physics Letters, 2013,102(18).

[892] Shomaker KL, Weiner T, Esther CR. Impact of an Evidence-Based Algorithm on Quality of Care in Pediatric Parapneumonic Effusion and Empyema [J]. Pediatric Pulmonology, 2011,46(7): 722-728.

[893] Shugman IM, Hsieh V, Cheng S, et al. Safety and efficacy of rescue angioplasty for ST-elevation myocardial infarction with high utilization rates of glycoprotein IIb/IIIa inhibitors [J]. American Heart Journal, 2012,163(4): 649-+.

[894] Shukla AN, Thakkar B, Jayaram AA, et al. Efficacy and safety of tenecteplase in pulmonary embolism [J]. Journal of Thrombosis and Thrombolysis, 2014,38(1): 24-29.

[895] Siahanidou T, Margeli A, Tsirogianni C, et al. Clinical Value of Plasma Soluble Urokinase-Type Plasminogen Activator Receptor Levels in Term Neonates with Infection or Sepsis: A Prospective Study [J]. Mediators of Inflammation, 2014.

[896] Siegler BH, Weiterer S, Lichtenstern C, et al. Use of biomarkers in sepsis. Update and perspectives [J]. Anaesthesist, 2014,63(8-9): 678-690.

[897] Singh B, Ahuja S, Singhal RK, et al. Radiosensitivity Studies and Radiostability of Ribulose-1,5 Bis-Carboxylase and Gas Exchange Characteristics in Wheat, Garden Pea, Field Pea, Spinach, and Okra [J]. Water Air and Soil Pollution, 2014,225(1).

[898] Sinha K, Pal PB, Sil PC. Cadmium (Cd2+) exposure differentially elicits both cell proliferation and cell death related responses in SK-RC-45 [J]. Toxicology in Vitro, 2014,28(2): 307-318.

[899] Skibsted S, Jones AE, Puskarich MA, et al. BIOMARKERS OF ENDOTHELIAL CELL ACTIVATION IN EARLY SEPSIS [J]. Shock, 2013,39(5): 427-432.

[900] Smithburger PL, Campbell S, Kane-Gill SL. Alteplase Treatment of Acute Pulmonary Embolism in the Intensive Care Unit [J]. Critical Care Nurse, 2013,33(2): 17-27.

[901] Smuder AJ, Min KS, Hudson MB, et al. Endurance exercise attenuates ventilator-induced diaphragm dysfunction [J]. Journal of Applied Physiology, 2012,112(3): 501-510.

[902] Snow SJ, Vizcaya-Ruiz A, Osornio-Vargas A, et al. THE EFFECT OF COMPOSITION, SIZE, AND SOLUBILITY ON ACUTE PULMONARY INJURY IN RATS FOLLOWING EXPOSURE TO MEXICO CITY AMBIENT PARTICULATE MATTER SAMPLES [J]. Journal of Toxicology and Environmental Health-Part a-Current Issues, 2014,77(19): 1164-1182.

[903] Soerensen KE, Olsen HG, Skovgaard K, et al. Disseminated Intravascular Coagulation in a Novel Porcine Model of Severe Staphylococcus aureus Sepsis Fulfills Human Clinical Criteria [J]. Journal of Comparative Pathology, 2013,149(4): 463-474.

[904] Son SK, Oh SH, Kim KM, et al. Successful liver transplantation following veno-arterial extracorporeal membrane oxygenation in a child with fulminant Wilson disease and severe pulmonary hemorrhage: A case report [J]. Pediatric Transplantation, 2012,16(7): E281-E285.

[905] Sopko L, Al Sabty F, Rimajova V, et al. The feasibility of an early hospital discharge following chemotherapy for the acute myeloid leukemia [J]. Bratislava Medical Journal-Bratislavske Lekarske Listy, 2012,113(5): 298-300.

[906] Soria-Guerra RE, Rosales-Mendoza S, Gasic K, et al. Gene Expression is Highly Regulated in Early Developing Fruit of Apple [J]. Plant Molecular Biology Reporter, 2011,29(4): 885-897.

[907] Starr ME, Hu YL, Stromberg AJ, et al. Gene expression profile of mouse white adipose tissue during inflammatory stress: age-dependent upregulation of major procoagulant factors [J]. Aging Cell, 2013,12(2): 194-206.

[908] Stein PD, Matta F. Thrombolytic Therapy in Unstable Patients with Acute Pulmonary Embolism: Saves Lives but Underused [J]. American Journal of Medicine, 2012,125(5): 465-470.

[909] Stephan F, Aarden LA, Zeerleder S. FSAP, a new player in inflammation? [J]. Hamostaseologie, 2012,32(1): 51-55.

[910] Stephan F, Bulder I, Luken BM, et al. Complexes of factor VII-activating protease with plasminogen activator inhibitor-1 in human sepsis [J]. Thrombosis and Haemostasis, 2014,112(1): 219-221.

[911] Suarez PR, Gilart JF, Perez JMH, et al. Treatment of complicated parapneumonic pleural effusion and pleural parapneumonic empyema [J]. Medical Science Monitor, 2012,18(7): CR443-CR449.

[912] Suberviola B, Castellanos-Ortega A, Ruiz AR, et al. Hospital mortality prognostication in sepsis using the new biomarkers suPAR and proADM in a single determination on ICU admission [J]. Intensive Care Medicine, 2013,39(11): 1945-1952.

[913] Sun GH, Liu XQ, Ren LH, et al. Construction of a full-length cDNA library of Solen grandis Dunker and identification of defense- and immune-related genes [J]. Journal of Ocean University of China, 2014,13(1): 169-173.

[914] Suto B, Szitter I, Bagoly T, et al. Plasma somatostatin-like immunoreactivity increases in the plasma of septic patients and rats with systemic inflammatory reaction: Experimental evidence for its sensory origin and protective role [J]. Peptides, 2014,54: 49-57.

[915] Sutter R, Bruder E, Weissenburg M, et al. Thyroid Hemorrhage Causing Airway Obstruction After Intravenous Thrombolysis for Acute Ischemic Stroke [J]. Neurocritical Care, 2013,19(3): 381-384.

[916] Svecova D, Havrankova M, Weismanova E, et al. Anogenital squamous cell carcinoma in neglected patient [J]. Bratislava Medical Journal-Bratislavske Lekarske Listy, 2012,113(4): 246-248.

[917] Swaidani S, Cheng G, Lauer ME, et al. TSG-6 Protein Is Crucial for the Development of Pulmonary Hyaluronan Deposition, Eosinophilia, and Airway Hyperresponsiveness in a Murine Model of Asthma [J]. Journal of Biological Chemistry, 2013,288(1): 412-422.

[918] Swarbreck SB, Secor D, Ellis CG, et al. Short-term effect of ascorbate on bacterial content, plasminogen activator inhibitor-1, and myeloperoxidase in septic mice [J]. Journal of Surgical Research, 2014,191(2): 432-440.

[919] Swift DC, Eggert JH, Hicks DG, et al. MASS-RADIUS RELATIONSHIPS FOR EXOPLANETS [J]. Astrophysical Journal, 2012,744(1).

[920] Syed F, Bagabir RA, Paus R, et al. Ex vivo evaluation of antifibrotic compounds in skin scarring: EGCG and silencing of PAI-1 independently inhibit growth and induce keloid shrinkage [J]. Laboratory Investigation, 2013,93(8): 946-960.

[921] Szklarczyk K, Korostynski M, Golda S, et al. Genotype-dependent consequences of traumatic stress in four inbred mouse strains [J]. Genes Brain and Behavior, 2012,11(8): 977-985.

[922] Taccone FS, Crimi E, Anstey J, et al. Endothelium and Regulatory Inflammatory Mechanisms During Organ Rejection [J]. Angiology, 2014,65(5): 379-387.

[923] Tanaka K, Ohara T, Ishigami A, et al. Fatal Multiple Systemic Emboli after Intravenous Thrombolysis for Cardioembolic Stroke [J]. Journal of Stroke & Cerebrovascular Diseases, 2014,23(2): 395-397.

[924] Tang GHL, Kim MC, Pinney SP, et al. Failed repeated thrombolysis requiring left ventricular assist device pump exchange [J]. Catheterization and Cardiovascular Interventions, 2013,81(6): 1072-1074.

[925] Tariq N, Adil MM, Saeed F, et al. Outcomes of Thrombolytic Treatment for Acute Ischemic Stroke in Dialysis-Dependent Patients in the United States [J]. Journal of Stroke & Cerebrovascular Diseases, 2013,22(8): E354-E359.

[926] Terpstra ML, Aman J, Amerongen GPV, et al. Plasma Biomarkers for Acute Respiratory Distress Syndrome: A Systematic Review and Meta-Analysis [J]. Critical Care Medicine, 2014,42(3): 691-700.

[927] Tetik S, Ak K, Sahin Y, et al. Postoperative Statin Therapy Attenuates the Intensity of Systemic Inflammation and Increases Fibrinolysis After Coronary Artery Bypass Grafting [J]. Clinical and Applied Thrombosis-Hemostasis, 2011,17(5): 526-531.

[928] Tichopad A, Roberts C, Gembula I, et al. Clinical and Economic Burden of Community-Acquired Pneumonia among Adults in the Czech Republic, Hungary, Poland and Slovakia [J]. Plos One, 2013,8(8).

[929] Tine M, Guinand B, Durand JD. Variation in gene expression along a salinity gradient in wild populations of the euryhaline black-chinned tilapia Sarotherodon melanotheron [J]. Journal of Fish Biology, 2012,80(4): 785-801.

[930] Tolstrup M, Johansen C, Toft L, et al. Anti-inflammatory effect of a retrovirus-derived immunosuppressive peptide in mouse models [J]. Bmc Immunology, 2013,14.

[931] Tomulic KL, Frleta N, Cace N, et al. Successful right atrial thrombus lysis with alteplase in a nine month old infant [J]. Signa Vitae, 2013,8(1): 59-61.

[932] Tourtas T, Birke MT, Kruse FE, et al. Preventive Effects of Omega-3 and Omega-6 Fatty Acids on Peroxide Mediated Oxidative Stress Responses in Primary Human Trabecular Meshwork Cells [J]. Plos One, 2012,7(2).

[933] Tsai HJ, Tsao CM, Liao MH, et al. Application of thrombelastography in liver injury induced by endotoxin in rat [J]. Blood Coagulation & Fibrinolysis, 2012,23(2): 118-126.

[934] Tucker T, Idell S. Plasminogen-Plasmin System in the Pathogenesis and Treatment of Lung and Pleural Injury [J]. Seminars in Thrombosis and Hemostasis, 2013,39(4): 373-381.

[935] Uchida Y, Takeshita K, Yamamoto K, et al. Stress Augments Insulin Resistance and Prothrombotic State [J]. Diabetes, 2012,61(6): 1552-1561.

[936] Uusitalo-Seppala R, Huttunen R, Tarkka M, et al. Soluble urokinase-type plasminogen activator receptor in patients with suspected infection in the emergency room: a prospective cohort study [J]. Journal of Internal Medicine, 2012,272(3): 247-256.

[937] Valle-Garay E, Montes AH, Corte JR, et al. tPA Alu (I/D) Polymorphism Associates With Bacterial Osteomyelitis [J]. Journal of Infectious Diseases, 2013,208(2): 218-223.

[938] van der Pals J, Gotberg MI, Gotberg M, et al. Hypothermia in cardiogenic shock reduces systemic t-PA release [J]. Journal of Thrombosis and Thrombolysis, 2011,32(1): 72-81.

[939] Vanska M, Purhonen AK, Koivula I, et al. Soluble form of urokinase-type plasminogen activator receptor as a diagnostic and prognostic marker in hematological patients with neutropenic fever [J]. Leukemia & Lymphoma, 2014,55(3): 718-721.

[940] Vantourout P, Willcox C, Turner A, et al. Immunological Visibility: Posttranscriptional Regulation of Human NKG2D Ligands by the EGF Receptor Pathway [J]. Science Translational Medicine, 2014,6(231).

[941] Vaseva I, Akiscan Y, Demirevska K, et al. Drought stress tolerance of red and white clover-comparative analysis of some chaperonins and dehydrins [J]. Scientia Horticulturae, 2011,130(3): 653-659.

[942] Vassileva V, Demirevska K, Simova-Stoilova L, et al. Long-Term Field Drought Affects Leaf Protein Pattern and Chloroplast Ultrastructure of Winter Wheat in a Cultivar-Specific Manner [J]. Journal of Agronomy and Crop Science, 2012,198(2): 104-117.

[943] Veress LA, Hendry-Hofer TB, Loader JE, et al. Tissue Plasminogen Activator Prevents Mortality from Sulfur Mustard Analog-Induced Airway Obstruction [J]. American Journal of Respiratory Cell and Molecular Biology, 2013,48(4): 439-447.

[944] Vincent JL, Beumier M. Diagnostic and prognostic markers in sepsis [J]. Expert Review of Anti-Infective Therapy, 2013,11(3): 265-275.

[945] Vivien D, Ali C. TRAUMATIC BRAIN INJURY Tissue-type plasminogen activator-harmful or beneficial? [J]. Nature Reviews Neurology, 2012,8(10): 538-539.

[946] Vlaar APJ, Hofstra JJ, Determann RM, et al. Transfusion-related acute lung injury in cardiac surgery patients is characterized by pulmonary inflammation and coagulopathy: A prospective nested case-control study [J]. Critical Care Medicine, 2012,40(10): 2813-2820.

[947] Vohra PK, Hoeppner LH, Sagar G, et al. Dopamine inhibits pulmonary edema through the VEGF-VEGFR2 axis in a murine model of acute lung injury [J]. American Journal of Physiology-Lung Cellular and Molecular Physiology, 2012,302(2): L185-L192.

[948] Vyas PA, Donato AA. Thrombolysis in Acute Pulmonary Thromboembolism [J]. Southern Medical Journal, 2012,105(10): 560-570.

[949] Wada T, Gando S, Mizugaki A, et al. Coagulofibrinolytic changes in patients with disseminated intravascular coagulation associated with post-cardiac arrest syndrome- Fibrinolytic shutdown and insufficient activation of fibrinolysis lead to organ dysfunction [J]. Thrombosis Research, 2013,132(1): E64-E69.

[950] Walley KR. Biomarkers in Sepsis [J]. Current Infectious Disease Reports, 2013,15(5): 413-420.

[951] Wang CT, Lin FC, Khor GT, et al. Life-threatening anaphylactoid shock caused by recombinant tissue plasminogen activator [J]. American Journal of Emergency Medicine, 2012,30(1).

[952] Wang D, Fan JZ, Heckathorn SA. Acclimation of photosynthetic tolerance to acute heat stress at elevated CO2 and N [J]. Plant Science, 2014,226: 162-171.

[953] Wang D, Heckathorn SA, Hamilton EW, et al. EFFECTS OF CO2 ON THE TOLERANCE OF PHOTOSYNTHESIS TO HEAT STRESS CAN BE AFFECTED BY PHOTOSYNTHETIC PATHWAY AND NITROGEN [J]. American Journal of Botany, 2014,101(1): 34-44.

[954] Wang FQ, Wang L, Xu ZY, et al. Identification and Analysis of Multi-Protein Complexes in Placenta [J]. Plos One, 2013,8(4).

[955] Wang H, Madhusudhan T, He T, et al. Low but sustained coagulation activation ameliorates glucose-induced podocyte apoptosis: protective effect of factor V Leiden in diabetic nephropathy [J]. Blood, 2011,117(19): 5231-5242.

[956] Wang X, Wang SX, Liu YT, et al. The Hsp90 inhibitor SNX-2112 induces apoptosis of human hepatocellular carcinoma cells: The role of ER stress [J]. Biochemical and Biophysical Research Communications, 2014,446(1): 160-166.

[957] Wang Z, Zhao Q, Han YX, et al. PAI-1 and IFN-gamma in the regulation of innate immune homeostasis during sublethal yersiniosis [J]. Blood Cells Molecules and Diseases, 2013,50(3): 196-201.

[958] Wang ZF, Fessler EB, Chuang DM. Beneficial effects of mood stabilizers lithium, valproate and lamotrigine in experimental stroke models [J]. Acta Pharmacologica Sinica, 2011,32(12): 1433-1445.

[959] Wang ZH, Ren WY, Zhu L, et al. Plasminogen Activator Inhibitor-1 Regulates LPS Induced Inflammation in Rat Macrophages through Autophagy Activation [J]. Scientific World Journal, 2014.

[960] Wang ZY, Wu SN, Zhu ZZ, et al. Inhaled unfractionated heparin improves abnormalities of alveolar coagulation, fibrinolysis and inflammation in endotoxemia-induced lung injury rats [J]. Chinese Medical Journal, 2013,126(2): 318-324.

[961] Weh JL, Kowaleski MP, Boudrieau RJ. Combination Tibial Plateau Leveling Osteotomy and Transverse Corrective Osteotomy of the Proximal Tibia for the Treatment of Complex Tibial Deformities in 12 dogs [J]. Veterinary Surgery, 2011,40(6): 670-686.

[962] Weng H, Deng YH, Xie YY, et al. Expression and significance of HMGB1, TLR4 and NF-kappa B p65 in human epidermal tumors [J]. Bmc Cancer, 2013,13.

[963] Westaby S, Kharbanda R, Banning AP. Cardiogenic shock in ACS. Part 1: prediction, presentation and medical therapy [J]. Nature Reviews Cardiology, 2012,9(3): 158-171.

[964] Wiergowski M, Anand JS, Krzyzanowski M, et al. Acute methoxetamine and amphetamine poisoning with fatal outcome: A case report [J]. International Journal of Occupational Medicine and Environmental Health, 2014,27(4): 683-690.

[965] Wiersinga WJ, Bonten MJ, Boersma WG, et al. SWAB/NVALT (Dutch Working Party on Antibiotic Policy and Dutch Association of Chest Physicians) Guidelines on the Management of Community-Acquired Pneumonia in Adults [J]. Netherlands Journal of Medicine, 2012,70(2): 90-101.

[966] Wilkinson TS, Morris AC, Kefala K, et al. Ventilator-Associated Pneumonia Is Characterized by Excessive Release of Neutrophil Proteases in the Lung [J]. Chest, 2012,142(6): 1425-1432.

[967] Wingeyer SP, Cunto E, Nogueras C, et al. Biomarkers in sepsis at time zero: intensive care unit scores, plasma measurements and polymorphisms in Argentina [J]. Journal of Infection in Developing Countries, 2012,6(7): 555-562.

[968] Wong CK, Herbison P. Initial Q waves and outcome after reperfusion therapy in patients with ST elevation acute myocardial infarction: A systematic review [J]. International Journal of Cardiology, 2011,148(3): 305-308.

[969] Wong HR. Genetics and genomics in pediatric septic shock [J]. Critical Care Medicine, 2012,40(5): 1618-1626.

[970] Wong MS, Sidik SM, Mahmud R, et al. Molecular targets in the discovery and development of novel antimetastatic agents: Current progress and future prospects [J]. Clinical and Experimental Pharmacology and Physiology, 2013,40(5): 307-319.

[971] Wood KE. Size does matter (not) [J]. Critical Care Medicine, 2011,39(11): 2560-2561.

[972] Wood KE. Major Pulmonary Embolism [J]. Critical Care Clinics, 2011,27(4): 885-+.

[973] Woolley LK, Fell SA, Djordjevic SP, et al. Plasmin activity in the porcine airways is enhanced during experimental infection with Mycoplasma hyopneumoniae, is positively correlated with proinflammatory cytokine levels and is ameliorated by vaccination [J]. Veterinary Microbiology, 2013,164(1-2): 60-66.

[974] Wrotek A, Pawlik K, Jackowska T. Soluble Receptor for Urokinase Plasminogen Activator in Community-Acquired Pneumonia in Children[A]. In: Neurobiology of Respiration (Pokorski M, ed), Vol. 788, 2013: 329-334.

[975] Xiao HB, Liu RH, Ling GH, et al. HSP47 regulates ECM accumulation in renal proximal tubular cells induced by TGF-beta 1 through ERK1/2 and JNK MAPK pathways [J]. American Journal of Physiology-Renal Physiology, 2012,303(5): F757-F765.

[976] Xie XP, Zhao RZ, Shen GX. Influence of Delphinidin-3-glucoside on Oxidized Low-Density Lipoprotein-Induced Oxidative Stress and Apoptosis in Cultured Endothelial Cells [J]. Journal of Agricultural and Food Chemistry, 2012,60(7): 1850-1856.

[977] Xue H, Ix JH, Wang WL, et al. Hemodialysis Access Usage Patterns in the Incident Dialysis Year and Associated Catheter-Related Complications [J]. American Journal of Kidney Diseases, 2013,61(1): 123-130.

[978] Yamori W, Hikosaka K, Way DA. Temperature response of photosynthesis in C-3, C-4, and CAM plants: temperature acclimation and temperature adaptation [J]. Photosynthesis Research, 2014,119(1-2): 101-117.

[979] Yang D, Song Y, Sun J, et al. ROLE OF PEROXIREDOXIN 6 IN THE EXPRESSION OF PLASMINOGEN ACTIVATOR INHIBITOR 1 IN LIPOPOLYSACCHARIDE-INDUCED ACUTE LUNG INJURY [J]. Respirology, 2011,16: 269-269.

[980] Yang XS, Liu MY, Zhang HM, et al. Protein kinase C-delta mediates sepsis-induced activation of complement 5a and urokinase-type plasminogen activator signaling in macrophages [J]. Inflammation Research, 2014,63(7): 581-589.

[981] Yang YQ, Chen JH, Liu Q, et al. Comparative Proteomic Analysis of the Thermotolerant Plant Portulaca oleracea Acclimation to Combined High Temperature and Humidity Stress [J]. Journal of Proteome Research, 2012,11(7): 3605-3623.

[982] Yende S, D'Angelo G, Mayr F, et al. Elevated Hemostasis Markers after Pneumonia Increases One-Year Risk of All-Cause and Cardiovascular Deaths [J]. Plos One, 2011,6(8).

[983] Yende S, Milbrandt EB, Kellum JA, et al. Understanding the potential role of statins in pneumonia and sepsis [J]. Critical Care Medicine, 2011,39(8): 1871-1878.

[984] Yilmaz G, Koksal I, Karahan SC, et al. The diagnostic and prognostic significance of soluble urokinase plasminogen activator receptor in systemic inflammatory response syndrome [J]. Clinical Biochemistry, 2011,44(14-15): 1227-1230.

[985] Yoshimura S, Shirakawa M, Uchida K, et al. Endovascular Treatment of Acute Ischemic Stroke: Honolulu Shock and Thereafter [J]. Journal of Stroke & Cerebrovascular Diseases, 2014,23(5): E295-E298.

[986] Yu D, Buchvald F, Brandt B, et al. Seventeen-year study shows rise in parapneumonic effusion and empyema with higher treatment failure after chest tube drainage [J]. Acta Paediatrica, 2014,103(1): 93-99.

[987] Yu J, Yan C. An artificial diffusivity discontinuous Galerkin scheme for discontinuous flows [J]. Computers & Fluids, 2013,75: 56-71.

[988] Yu L, Wu XL, Wang H, et al. Diagnostic and prognostic significance of suPAR in traumatic brain injury [J]. Neurology India, 2014,62(5): 498-502.

[989] Zalewski J, Bogaerts K, Desmet W, et al. Intraluminal Thrombus in Facilitated Versus Primary Percutaneous Coronary Intervention An Angiographic Substudy of the ASSENT-4 PCI (Assessment of the Safety and Efficacy of a New Treatment Strategy With Percutaneous Coronary Intervention) Trial [J]. Journal of the American College of Cardiology, 2011,57(19): 1867-1873.

[990] Zarar A, Khan AA, Adil MM, et al. Anaphylactic shock associated with intravenous thrombolytics [J]. American Journal of Emergency Medicine, 2014,32(1).

[991] Zemans RL, McClendon J, Aschner Y, et al. Role of beta-catenin-regulated CCN matricellular proteins in epithelial repair after inflammatory lung injury [J]. American Journal of Physiology-Lung Cellular and Molecular Physiology, 2013,304(6): L415-L427.

[992] Zentai C, Spronk HMH, Van Oerle R, et al. Prolonged shock phase in a pig model with multiple injuries is associated with a decreased onset of tPA induced fibrinolysis [J]. Journal of Thrombosis and Haemostasis, 2013,11: 50-50.

[993] Zhang Y, Xiao W, Jiang Y, et al. Levels of Components of the Urokinase-type Plasminogen Activator System are Related to Chronic Obstructive Pulmonary Disease Parenchymal Destruction and Airway Remodelling [J]. Journal of International Medical Research, 2012,40(3): 976-985.

[994] Zhao RZ, Le K, Li WD, et al. Effects of Saskatoon berry powder on monocyte adhesion to vascular wall of leptin receptor-deficient diabetic mice [J]. Journal of Nutritional Biochemistry, 2014,25(8): 851-857.

[995] Zhao RZ, Le K, Moghadasian MH, et al. Regulatory role of NADPH oxidase in glycated LDL-induced upregulation of plasminogen activator inhibitor-1 and heat shock factor-1 in mouse embryo fibroblasts and diabetic mice [J]. Free Radical Biology and Medicine, 2013,61: 18-25.

[996] Zhao RZ, Moghadasian MH, Shen GX. Involvement of NADPH oxidase in up-regulation of plasminogen activator inhibitor-1 and heat shock factor-1 in mouse embryo fibroblasts induced by oxidized LDL and in apolipoprotein E-deficient mice [J]. Free Radical Research, 2011,45(9): 1013-1023.

[997] Zhao RZ, Ren S, Moghadasain MH, et al. Involvement of fibrinolytic regulators in adhesion of monocytes to vascular endothelial cells induced by glycated LDL and to aorta from diabetic mice [J]. Journal of Leukocyte Biology, 2014,95(6): 941-949.

[998] Zhao RZ, Xie XP, Shen GX. Effects of glycated low-density lipoprotein on cell viability, proliferation, and growth factors of mouse embryo fibroblasts [J]. Canadian Journal of Physiology and Pharmacology, 2013,91(1): 64-70.

[999] Zhuang JL, Jiang QW, Xu Y, et al. Recombinant activated factor VII in hemophagocytic lymphohistiocytosis with disseminated intravascular coagulation [J]. Chinese Medical Journal, 2011,124(19): 3189-3191.

[1000] Zmijewski JW, Bae HB, Deshane JS, et al. Inhibition of neutrophil apoptosis by PAI-1 [J]. American Journal of Physiology-Lung Cellular and Molecular Physiology, 2011,301(2): L247-L254.

[1001] Abraham E. Anticoagulants for acute lung injury: Barking up the wrong tree [J]. Critical Care Medicine, 2009,37(6): 2100-2101.

[1002] Ackerman A. Meningococcal sepsis in children: Persistent problem; new insights? [J]. Critical Care Medicine, 2010,38(1): 316-317.

[1003] Adair JE, Stober V, Sobhany M, et al. Inter-alpha-trypsin Inhibitor Promotes Bronchial Epithelial Repair after Injury through Vitronectin Binding [J]. Journal of Biological Chemistry, 2009,284(25): 16922-16930.

[1004] Adams JA, Bassuk JA, Arias J, et al. Effects of "Delayed Postconditioning" With Periodic Acceleration After Asphyxia Induced Shock in Pigs [J]. Pediatric Research, 2008,64(5): 533-537.

[1005] Ahn JS, Moon SH, Kim J, et al. Identification of Differentially Expressed Genes in Human Embryonic Stem Cell-Derived Endothelial Cells Using Suppression Subtractive Hybridization [J]. Stem Cells and Development, 2010,19(8): 1249-1256.

[1006] Ahn SY, Ingulli E. Acute poststreptococcal glomerulonephritis: an update [J]. Current Opinion in Pediatrics, 2008,20(2): 157-162.

[1007] Ait-Oufella H, Maury E, Lehoux S, et al. The endothelium: physiological functions and role in microcirculatory failure during severe sepsis [J]. Intensive Care Medicine, 2010,36(8): 1286-1298.

[1008] Akkus MN, Polat G, Yurtdas M, et al. Admission Levels of C-Reactive Protein and Plasminogen Activator Inhibitor-1 in Patients With Acute Myocardial Infarction With and Without Cardiogenic Shock or Heart Failure on Admission [J]. International Heart Journal, 2009,50(1): 33-45.

[1009] Allen GB, Cloutier ME, Larrabee YC, et al. Neither fibrin nor plasminogen activator inhibitor-1 deficiency protects lung function in a mouse model of acute lung injury [J]. American Journal of Physiology-Lung Cellular and Molecular Physiology, 2009,296(3): L277-L285.

[1010] Amara U, Rittirsch D, Flierl M, et al. Interaction Between the Coagulation and Complement System[A]. In: Current Topics in Complement Ii (Lambris JD, ed), Vol. 632, 2008: 71-79.

[1011] Anaya-Prado R, Perez-Gomez N, Toledo-Pereyra LH, et al. Small molecule selectin inhibitor in global cerebral ischemia and controlled hemorrhagic shock [J]. Journal of Trauma-Injury Infection and Critical Care, 2008,65(3): 678-684.

[1012] Andrades ME, Lorenzi R, Berger M, et al. Glycolaldehyde induces fibrinogen post-translational modification, delay in clotting and resistance to enzymatic digestion [J]. Chemico-Biological Interactions, 2009,180(3): 478-484.

[1013] Arancibia MF, Vega-Briceno LE, Pizarro ME, et al. Empyema and pleural efussion in children [J]. Revista Chilena De Infectologia, 2007,24(6): 454-461.

[1014] Arcaroli J, Sankoff J, Liu N, et al. Association between urokinase haplotypes and outcome from infection-associated acute lung injury [J]. Intensive Care Medicine, 2008,34(2): 300-307.

[1015] Armstrong PW, Gershlick A, Goldstein P, et al. The Strategic Reperfusion Early After Myocardial Infarction (STREAM) study [J]. American Heart Journal, 2010,160(1): 30-U49.

[1016] Arntz HR, Wenzel V, Dissmann R, et al. Out-of-hospital thrombolysis during cardiopulmonary resuscitation in patients with high likelihood of ST-elevation myocardial infarction [J]. Resuscitation, 2008,76(2): 180-184.

[1017] Arora RS, Roberts R, Eden TOB, et al. Interventions other than anticoagulants and systemic antibiotics for prevention of central venous catheter-related infections in children with cancer [J]. Cochrane Database of Systematic Reviews, 2010, (12).

[1018] Autret-Leca E, Bauer S, Alberti C, et al. Glucocorticoide therapy in premature infants: French practices in 2006 [J]. Archives De Pediatrie, 2009,16(7): 999-1004.

[1019] Averna M, Stifanese R, De Tullio R, et al. Calpain-mediated activation of NO synthase in human neuroblastoma SK-N-BE cells [J]. Journal of Neurochemistry, 2009,110(1): 412-421.

[1020] Aydin M, Kislal FM, Ayar A, et al. The effects of lipopolysaccharide-induced endogenous hyperthermia and different antipyretic treatment modalities on rat brain [J]. Bratislava Medical Journal-Bratislavske Lekarske Listy, 2011,112(5): 227-234.

[1021] Aykas A, Yuzbasioglu MF, Kurutas EB, et al. Protective effects of propofol on peritoneal adhesions in cecal ligation and puncture model [J]. Bratislava Medical Journal-Bratislavske Lekarske Listy, 2010,111(5): 253-257.

[1022] Azarisman SMS, Liza RA, Radhiana H, et al. Immediate postpartum cardiorespiratory collapse: a management quandary [J]. Blood Coagulation & Fibrinolysis, 2010,21(6): 601-604.

[1023] Azat C, Pacik D, Varga G, et al. The efficiency of urolithiasis treatment with extracorporeal lithotripsy in relation to the shock waves frequency [J]. Bratislava Medical Journal-Bratislavske Lekarske Listy, 2010,111(12): 644-646.

[1024] Babala J, Cingel V, Hargas M, et al. Devastating injury of the groin with vascular lesion [J]. Bratislava Medical Journal-Bratislavske Lekarske Listy, 2009,110(5): 293-295.

[1025] Backes Y, van der Sluijs KF, de Boer AMT, et al. Soluble urokinase-type plasminogen activator receptor levels in patients with burn injuries and inhalation trauma requiring mechanical ventilation: an observational cohort study [J]. Critical Care, 2011,15(6).

[1026] Bainey KR, Fu YL, Wagner GS, et al. Spontaneous reperfusion in ST-elevation myocardial infarction: Comparison of angiographic and electrocardiographic assessments [J]. American Heart Journal, 2008,156(2): 248-255.

[1027] Baqai FP, Gridley DS, Slater JM, et al. Effects of spaceflight on innate immune function and antioxidant gene expression [J]. Journal of Applied Physiology, 2009,106(6): 1935-1942.

[1028] Barber RC, Chang LYE, Lemaire SM, et al. Epistatic interactions are critical to gene-association studies: PAI-1 and risk for mortality after burn injury [J]. Journal of Burn Care & Research, 2008,29(1): 168-175.

[1029] Basheer M, Schwalb H, Nesher M, et al. Mast cell activation by fibrinogen-related homologous c-terminal peptides (haptides) modulates systemic blood pressure [J]. Journal of Allergy and Clinical Immunology, 2010,126(5): 1041-1048.

[1030] Batyraliev TA, Fettser DV, Vural A, et al. Safety and Efficacy of the Use of Glycoprotein IIb/IIIa Inhibitors in Invasive Treatment of Patients With ST-Elevation Acute Coronary Syndrome [J]. Kardiologiya, 2009,49(6): 4-9.

[1031] Bauer T, Hoffmann R, Junger C, et al. Efficacy of a 24-h primary percutaneous coronary intervention service on outcome in patients with ST elevation myocardial infarction in clinical practice [J]. Clinical Research in Cardiology, 2009,98(3): 171-178.

[1032] Bauman KA, Wettlaufer SH, Okunishi K, et al. The antifibrotic effects of plasminogen activation occur via prostaglandin E-2 synthesis in humans and mice [J]. Journal of Clinical Investigation, 2010,120(6): 1950-1960.

[1033] Bdeir K, Higazi AAR, Kulikovskaya I, et al. Neutrophil alpha-Defensins Cause Lung Injury by Disrupting the Capillary-Epithelial Barrier [J]. American Journal of Respiratory and Critical Care Medicine, 2010,181(9): 935-946.

[1034] Beljanski V, Knaak C, Smith CD. A Novel Sphingosine Kinase Inhibitor Induces Autophagy in Tumor Cells [J]. Journal of Pharmacology and Experimental Therapeutics, 2010,333(2): 454-464.

[1035] Beres SB, Sesso R, Pinto SWL, et al. Genome Sequence of a Lancefield Group C Streptococcus zooepidemicus Strain Causing Epidemic Nephritis: New Information about an Old Disease [J]. Plos One, 2008,3(8).

[1036] Berreau LM, Borowski T, Grubel K, et al. Mechanistic Studies of the O-2-Dependent Aliphatic Carbon-Carbon Bond Cleavage Reaction of a Nickel Enolate Complex [J]. Inorganic Chemistry, 2011,50(3): 1047-1057.

[1037] Beyer J, Halbritter K, Schellong S, et al. Influence of antithrombin and argatroban on disseminated intravascular coagulation parameters in a patient with septic shock [J]. Thrombosis Research, 2009,124(3): 383-386.

[1038] Beyrich C, Loffler J, Kobsar A, et al. Infection of Human Coronary Artery Endothelial Cells by Group B Streptococcus Contributes to Dysregulation of Apoptosis, Hemostasis, and Innate Immune Responses [J]. Mediators of Inflammation, 2011.

[1039] Bhandary YP, Velusamy T, Shetty P, et al. Post-Transcriptional Regulation of Urokinase-type Plasminogen Activator Receptor Expression in Lipopolysaccharide-induced Acute Lung Injury [J]. American Journal of Respiratory and Critical Care Medicine, 2009,179(4): 288-298.

[1040] Bishay M, Short M, Shah K, et al. Efficacy of video-assisted thoracoscopic surgery in managing childhood empyema: a large single-centre study [J]. Journal of Pediatric Surgery, 2009,44(2): 337-342.

[1041] Block AA, Thursky KA, Worth LJ, et al. Thrombolytic therapy for management of complicated catheter-related Candida albicans thrombophlebitis [J]. Internal Medicine Journal, 2009,39(1): 61-63.

[1042] Boates B, Hamel S, Schwegler E, et al. Structural and optical properties of liquid CO2 for pressures up to 1 TPa [J]. Journal of Chemical Physics, 2011,134(6).

[1043] Bouchama A, Kunzelmann C, Dehbi M, et al. Recombinant activated protein C attenuates endothelial injury and inhibits procoagulant microparticles release in baboon heatstroke [J]. Arteriosclerosis Thrombosis and Vascular Biology, 2008,28(7): 1318-1325.

[1044] Bradley DK, Eggert JH, Smith RF, et al. Diamond at 800 GPa [J]. Physical Review Letters, 2009,102(7).

[1045] Brander L, Sinderby C, Lecomte F, et al. Neurally adjusted ventilatory assist decreases ventilator-induced lung injury and non-pulmonary organ dysfunction in rabbits with acute lung injury [J]. Intensive Care Medicine, 2009,35(11): 1979-1989.

[1046] Brims FJH, Chauhan AJ, Higgins B, et al. Up-regulation of the Extrinsic Coagulation Pathway in Acute Asthma-A Case Study [J]. Journal of Asthma, 2010,47(6): 695-698.

[1047] Brohi K, Cohen MJ, Davenport RA. Acute coagulopathy of trauma: mechanism, identification and effect [J]. Current Opinion in Critical Care, 2007,13(6): 680-685.

[1048] Brohi K, Cohen MJ, Ganter MT, et al. Acute coagulopathy of trauma: Hypoperfusion induces systemic anticoagulation and hyperfibrinolysis [J]. Journal of Trauma-Injury Infection and Critical Care, 2008,64(5): 1211-1217.

[1049] Brorsson AC, Kumita JR, MacLeod I, et al. Methods and models in neurodegenerative and systemic protein aggregation diseases [J]. Frontiers in Bioscience-Landmark, 2010,15: 373-396.

[1050] Bundesmann MM, Schnapp LM. Urokinase Plasminogen Activator Receptor Associated Protein (uPARAP) -/- Mice Have Improved Survival In A Bleomycin Model Of Acute Lung Injury [J]. American Journal of Respiratory and Critical Care Medicine, 2010,181.

[1051] Bune LT, Thaning P, Johansson PI, et al. Effects of nucleotides and nucleosides on coagulation [J]. Blood Coagulation & Fibrinolysis, 2010,21(5): 436-441.

[1052] Burkhart TA, Andrews DM. Activation level of extensor carpi ulnaris affects wrist and elbow acceleration responses following simulated forward falls [J]. Journal of Electromyography and Kinesiology, 2010,20(6): 1203-1210.

[1053] Busk M, Maeng M, Kristensen SD, et al. Timing, Causes, and Predictors of Death After Three Years' Follow-Up in the Danish Multicenter Randomized Study of Fibrinolysis Versus Primary Angioplasty in Acute Myocardial Infarction (DANAMI-2) Trial [J]. American Journal of Cardiology, 2009,104(2): 210-215.

[1054] Buyne OR, Bleichrodt RP, van Goor H, et al. Plasminogen activator, but not systemic antibiotic therapy, prevents abscess formation in an experimental model of secondary peritonitis [J]. British Journal of Surgery, 2008,95(10): 1287-1293.

[1055] Buyne OR, van Goor H, Verweij PE, et al. Timing and Dose of Tissue Plasminogen Activator to Prevent Abscess Formation After Surgical Treatment of Secondary Peritonitis in the Rat [J]. Surgical Innovation, 2009,16(4): 299-305.

[1056] Cai GY, Chen XM. Immunoglobulin A Nephropathy in China: Progress and Challenges [J]. American Journal of Nephrology, 2009,30(3): 268-273.

[1057] Cantor WJ, Fitchett D, Borgundvaag B, et al. Routine Early Angioplasty after Fibrinolysis for Acute Myocardial Infarction [J]. New England Journal of Medicine, 2009,360(26): 2705-2718.

[1058] Cantor WJ, Fitchett D, Borgundvaag B, et al. Rationale and design of the trial of routine angioplasty and stenting after fibrinolysis to enhance reperfusion in acute myocardial infarction (TRANSFER-AMI) [J]. American Heart Journal, 2008,155(1): 19-25.

[1059] Carter E, Waldhausen J, Zhang WY, et al. Management of Children With Empyema: Pleural Drainage Is Not Always Necessary [J]. Pediatric Pulmonology, 2010,45(5): 475-480.

[1060] Castellino FJ, Ploplis VA. The protein C pathway and pathologic processes [J]. Journal of Thrombosis and Haemostasis, 2009,7: 140-145.

[1061] Cauwels A, Brouckaert P. Critical role for small and large conductance calcium-dependent potassium channels in endotoxemia and TNF toxicity [J]. Shock, 2008,29(5): 577-582.

[1062] Cauwels A, Janssen B, Brouckaert P. The potential involvement of ROS and SK channels in inflammatory hypotension and shock [J]. Basic & Clinical Pharmacology & Toxicology, 2008,102: 42-42.

[1063] Cauwels A, Rogge E, Janssen B, et al. Reactive oxygen species and small-conductance calcium-dependent potassium channels are key mediators of inflammation-induced hypotension and shock [J]. Journal of Molecular Medicine-Jmm, 2010,88(9): 921-930.

[1064] Cawthorn WP, Sethi JK. TNF-alpha and adipocyte biology [J]. Febs Letters, 2008,582(1): 117-131.

[1065] Cesaro S, Tridello G, Cavaliere M, et al. Prospective, Randomized Trial of Two Different Modalities of Flushing Central Venous Catheters in Pediatric Patients With Cancer [J]. Journal of Clinical Oncology, 2009,27(12): 2059-2065.

[1066] Cevik Y, Ozer M, Das M, et al. Complications of misuse of Bacillus Calmette-Guerin [J]. Bratislava Medical Journal-Bratislavske Lekarske Listy, 2011,112(6): 363-364.

[1067] Chalmers JD, Singanayagam A, Murray MP, et al. Risk factors for complicated parapneumonic effusion and empyema on presentation to hospital with community-acquired pneumonia [J]. Thorax, 2009,64(7): 592-597.

[1068] Chan MR. Hemodialysis Central Venous Catheter Dysfunction [J]. Seminars in Dialysis, 2008,21(6): 516-521.

[1069] Chang WH, Tien CL, Chen TJ, et al. Decreased protein synthesis of Hsp27 associated with cellular toxicity in a cell model of Machado-Joseph disease [J]. Neuroscience Letters, 2009,454(2): 152-156.

[1070] Chauhan H, Khurana N, Tyagi AK, et al. Identification and characterization of high temperature stress responsive genes in bread wheat (Triticum aestivum L.) and their regulation at various stages of development [J]. Plant Molecular Biology, 2011,75(1-2): 35-51.

[1071] Chechi T, Vecchio S, Spaziani G, et al. Rheolytic Thrombectomy in Patients With Massive and Submassive Acute Pulmonary Embolism [J]. Catheterization and Cardiovascular Interventions, 2009,73(4): 506-513.

[1072] Chen BY, Wei JG, Wang W, et al. Identification of Signaling Pathways Involved in Aberrant Production of Adipokines in Adipocytes Undergoing Oxidative Stress [J]. Archives of Medical Research, 2009,40(4): 241-248.

[1073] Chen CM, Chou HC, Wang LF, et al. Captopril decreases plasminogen activator inhibitor-1 in rats with ventilator-induced lung injury [J]. Critical Care Medicine, 2008,36(6): 1880-1885.

[1074] Chen J, Hayes G, Waterer GW, et al. Tissue Plasminogen Activator (tPA) Polymorphism Associated with Response to Tifacogin in Severe Sepsis [J]. American Journal of Respiratory and Critical Care Medicine, 2009,179.

[1075] Chetty C, Bhoopathi P, Rao JS, et al. Inhibition of matrix metalloproteinase-2 enhances radiosensitivity by abrogating radiation-induced FoxM1-mediated G2/M arrest in A549 lung cancer cells [J]. International Journal of Cancer, 2009,124(10): 2468-2477.

[1076] Chiu CY, Wong KS, Huang JL, et al. Proinflammatory cytokines, fibrinolytic system enzymes, and biochemical indices in children with infectious para-pneumonic effusions [J]. Pediatric Infectious Disease Journal, 2008,27(8): 699-703.

[1077] Choi G, Hofstra JJH, Roelofs J, et al. Antithrombin inhibits bronchoalveolar activation of coagulation and limits lung injury during Streptococcus pneumoniae pneumonia in rats [J]. Critical Care Medicine, 2008,36(1): 204-210.

[1078] Christie JD, Bellamy S, Ware LB, et al. Construct validity of the definition of primary graft dysfunction after lung transplantation [J]. Journal of Heart and Lung Transplantation, 2010,29(11): 1231-1239.

[1079] Christie NA. Management of Pleural Space: Effusions and Empyema [J]. Surgical Clinics of North America, 2010,90(5): 919-+.

[1080] Chu SC, Yang SF, Lue KH, et al. Naproxen, meloxicam and methylprednisolone inhibit urokinase plasminogen activator and inhibitor and gelatinases expression during the early stage of osteoarthritis [J]. Clinica Chimica Acta, 2008,387(1-2): 90-96.

[1081] Chu SC, Yang SF, Tzang BS, et al. Cathepsin B and cystatin C play an inflammatory role in gouty arthritis of the knee [J]. Clinica Chimica Acta, 2010,411(21-22): 1788-1792.

[1082] Chung MC, Jorgensen SC, Popova TG, et al. Activation of plasminogen activator inhibitor implicates protease InhA in the acute-phase response to Bacillus anthracis infection [J]. Journal of Medical Microbiology, 2009,58(6): 737-744.

[1083] Clavijo JA, van Bastelaar J, Pinsky MR, et al. Minimally invasive Real Time Monitoring of mitochondrial NADH and tissue blood flow in the urethral wall during hemorrhage and resuscitation [J]. Medical Science Monitor, 2008,14(9): BR175-BR182.

[1084] Cohen MJ, Brohi K, Calfee CS, et al. Early release of high mobility group box nuclear protein 1 after severe trauma in humans: role of injury severity and tissue hypoperfusion [J]. Critical Care, 2009,13(6).

[1085] Cohen MJ, Carles M, Brohi K, et al. Early Release of Soluble Receptor for Advanced Glycation Endproducts After Severe Trauma in Humans [J]. Journal of Trauma-Injury Infection and Critical Care, 2010,68(6): 1273-1278.

[1086] Collard HR, Calfee CS, Wolters PJ, et al. Plasma biomarker profiles in acute exacerbation of idiopathic pulmonary fibrosis [J]. American Journal of Physiology-Lung Cellular and Molecular Physiology, 2010,299(1): L3-L7.

[1087] Collins D, Hogan AM, O'Shea D, et al. The Omentum: Anatomical, Metabolic, and Surgical Aspects [J]. Journal of Gastrointestinal Surgery, 2009,13(6): 1138-1146.

[1088] Cornell TT, Wynn J, Shanley TP, et al. Mechanisms and Regulation of the Gene-Expression Response to Sepsis [J]. Pediatrics, 2010,125(6): 1248-1258.

[1089] Correa-Costa M, Semedo P, Monteiro A, et al. Induction of Heme Oxygenase-1 Can Halt and Even Reverse Renal Tubule-Interstitial Fibrosis [J]. Plos One, 2010,5(12).

[1090] Cox JM, Kalns JE. Development and Characterization of a Rat Model of Nonpenetrating Liver Trauma [J]. Comparative Medicine, 2010,60(3): 218-224.

[1091] Craik CS, Page MJ, Madison EL. Proteases as therapeutics [J]. Biochemical Journal, 2011,435: 1-16.

[1092] Crosby LM, Waters CM. Epithelial repair mechanisms in the lung [J]. American Journal of Physiology-Lung Cellular and Molecular Physiology, 2010,298(6): L715-L731.

[1093] Dahlem P, van Aalderen WMC, Bos AP. Pediatric acute lung injury [J]. Paediatric Respiratory Reviews, 2007,8(4): 348-362.

[1094] Davis MDP, Dy KM, Nelson S. Presentation and outcome of purpura fulminans associated with peripheral gangrene in 12 patients at Mayo Clinic [J]. Journal of the American Academy of Dermatology, 2007,57(6): 944-956.

[1095] de Bock CE, Lin Z, Mekkawy AH, et al. Interaction between urokinase receptor and heat shock protein MRJ enhances cell adhesion [J]. International Journal of Oncology, 2010,36(5): 1155-1163.

[1096] de Bonis S, Rendina D, Vargas G, et al. Predictors of In-Hospital and Long-Term Clinical Outcome in Elderly Patients with Massive Pulmonary Embolism Receiving Thrombolytic Therapy [J]. Journal of the American Geriatrics Society, 2008,56(12): 2273-2277.

[1097] de Visser YP, Walther FJ, Laghmani EH, et al. Sildenafil attenuates pulmonary inflammation and fibrin deposition, mortality and right ventricular hypertrophy in neonatal hyperoxic lung injury [J]. Respiratory Research, 2009,10.

[1098] della Vergiliana JFV, Asokananthan N, Stewart GA. Activation of the plasma kallikrein-kinin system on human lung epithelial cells [J]. Biological Chemistry, 2010,391(9): 1067-1077.

[1099] Demirel A, Celkan T, Kasapcopur O, et al. Is Familial Mediterranean Fever a thrombotic disease or not? [J]. European Journal of Pediatrics, 2008,167(3): 279-285.

[1100] Dempfle CE, Borggrefe M. Thrombin-activatable fibrinolysis inhibitor-a inhibitors: Drugs for sepsis or drugs for disseminated intravascular coagulation? [J]. Critical Care Medicine, 2009,37(5): 1823-1824.

[1101] Dempfle CEH, Elmas E, Link A, et al. Endogenous plasma activated protein C levels and the effect of enoxaparin and drotrecogin alfa (activated) on markers of coagulation activation and fibrinolysis in pulmonary embolism [J]. Critical Care, 2011,15(1).

[1102] Dessing MC, Butter LM, Teske GJ, et al. S100A8/A9 Is Not Involved in Host Defense against Murine Urinary Tract Infection [J]. Plos One, 2010,5(10).

[1103] Dessing MC, Schouten M, Draing C, et al. Role played by Toll-like receptors 2 and 4 in lipoteichoic acid-induced lung inflammation and coagulation [J]. Journal of Infectious Diseases, 2008,197(2): 245-252.

[1104] Ding Q, Gladson CL, Wu HJ, et al. Focal adhesion kinase (FAK)-related non-kinase inhibits myofibroblast differentiation through differential MAPK activation in a FAK-dependent manner [J]. Journal of Biological Chemistry, 2008,283(40): 26839-26849.

[1105] Diskin CJ, Stokes TJ, Dansby LM, et al. Understanding the pathophysiology of hemodialysis access problems as a prelude to developing innovative therapies [J]. Nature Clinical Practice Nephrology, 2008,4(11): 628-638.

[1106] Dixon B, Opeskin K, Stamaratis G, et al. Pre-operative heparin reduces pulmonary microvascular fibrin deposition following cardiac surgery [J]. Thrombosis Research, 2011,127(1): E27-E30.

[1107] Dixon B, Santamaria JD, Campbell DJ. A phase 1 trial of nebulised heparin in acute lung injury [J]. Critical Care, 2008,12(3).

[1108] Doganay M, Yuksek YN, Daglar G, et al. Clinical determinants of suppurative cholangitis in malignant biliary tract obstruction [J]. Bratislava Medical Journal-Bratislavske Lekarske Listy, 2010,111(6): 336-339.

[1109] Drakos SG, Anastasiou-Nana MI, Terrovitis JV, et al. Intra-aortic balloon counterpulsation and delayed revascularization for myocardial infarction and shock in the absence of primary angioplasty: a treatment strategy with or without thrombolysis? [J]. Coronary Artery Disease, 2008,19(7): 521-526.

[1110] Dudek D, Dziewierz A, Siudak Z, et al. Transportation with very long transfer delays (> 90 min) for facilitated PCI with reduced-dose fibrinolysis in patients with ST-segment elevation myocardial infarction The Krakow Network [J]. International Journal of Cardiology, 2010,139(3): 218-227.

[1111] Dudek D, Rakowski T, El Massri N, et al. Patency of infarct related artery after pharmacological reperfusion during transfer to primary percutaneous coronary intervention influences left ventricular function and one-year clinical outcome [J]. International Journal of Cardiology, 2008,124(3): 326-331.

[1112] Eberlein M, Scheibner KA, Black KE, et al. Anti-oxidant inhibition of hyaluronan fragment-induced inflammatory gene expression [J]. Journal of Inflammation-London, 2008,5.

[1113] Eggert JH, Hicks DG, Celliers PM, et al. Melting temperature of diamond at ultrahigh pressure [J]. Nature Physics, 2010,6(1): 40-43.

[1114] Elikowski W, Malek M, Bar-Letkiewicz I, et al. Massive pulmonary embolism treated with a reduced dose of alteplase in a patient with acute renal failure [J]. Kardiologia Polska, 2008,66(8): 885-888.

[1115] Ellis SG, Tendera M, de Belder MA, et al. Facilitated PCI in patients with ST-elevation myocardial infarction [J]. New England Journal of Medicine, 2008,358(21): 2205-2217.

[1116] Elmas E, Suvajac N, Jilma B, et al. Factor V Leiden mutation enhances fibrin formation and dissolution in vivo in a human endotoxemia model [J]. Blood, 2010,116(5): 801-805.

[1117] Erdede M, Denizbasi A, Onur O, et al. Do we really need blood cultures in treating patients with community-acquired pneumonia? [J]. Bratislava Medical Journal-Bratislavske Lekarske Listy, 2010,111(5): 286-289.

[1118] Eren B, Fedakar R, Turkmen N, et al. Deaths in the Turkish Hamam (Hot Bath) [J]. Bratislava Medical Journal-Bratislavske Lekarske Listy, 2009,110(11): 697-700.

[1119] Ergelen M, Uyarel H, Gorgulu S, et al. Comparison of outcomes in young versus nonyoung patients with ST elevation myocardial infarction treated by primary angioplasty [J]. Coronary Artery Disease, 2010,21(2): 72-77.

[1120] Esmon CT. Crosstalk between inflammation and thrombosis (Reprinted from Maturitas, vol 47, pg 305-314, 2004) [J]. Maturitas, 2008,61(1-2): 122-131.

[1121] Faber J, Henninger N, Finn A, et al. A toll-like receptor 4 variant is associated with fatal outcome in children with invasive meningococcal disease [J]. Acta Paediatrica, 2009,98(3): 548-552.

[1122] Favreau F, Thuillier R, Cau J, et al. Anti-thrombin Therapy During Warm Ischemia and Cold Preservation Prevents Chronic Kidney Graft Fibrosis in a DCD Model [J]. American Journal of Transplantation, 2010,10(1): 30-39.

[1123] Feichtinger G, Raeven P, Weixelbaumer K, et al. siRNA mediated knockdown of plasminogen activator inhibitor 1 in experimental sepsis [J]. Human Gene Therapy, 2009,20(11): 1529-1529.

[1124] Ferreira ED, de Carvalho JB, Peixoto RJM, et al. The interaction of Bacteroides fragilis with components of the human fibrinolytic system [J]. Fems Immunology and Medical Microbiology, 2009,56(1): 48-55.

[1125] Fontanelli A, Bonanno C. Primary percutaneous coronary intervention in 'early' latecomers with ST-segment elevation acute myocardial infarction: the role of the infarct-related artery status [J]. Journal of Cardiovascular Medicine, 2011,12(1): 13-18.

[1126] Foreman MG, Demeo DL, Hersh CP, et al. Polymorphic variation in surfactant protein B is associated with COPD exacerbations [J]. European Respiratory Journal, 2008,32(4): 938-944.

[1127] Fouassier M, Soulweine B, Sapin AF, et al. Increase in proinflammatory cytokines in peripheral blood without haemostatic changes after LPS inhalation [J]. Thrombosis Research, 2009,124(5): 584-587.

[1128] Franchini M, Di Minno MND, Coppola A. Disseminated Intravascular Coagulation in Hematologic Malignancies [J]. Seminars in Thrombosis and Hemostasis, 2010,36(4): 388-403.

[1129] Francischetti IMB, Seydel KB, Monteiro RQ. Blood coagulation, inflammation, and malaria [J]. Microcirculation, 2008,15(2): 81-107.

[1130] Franco AA, Kothary MH, Gopinath G, et al. Cpa, the Outer Membrane Protease of Cronobacter sakazakii, Activates Plasminogen and Mediates Resistance to Serum Bactericidal Activity [J]. Infection and Immunity, 2011,79(4): 1578-1587.

[1131] Fu JM, Momcilovic I, Clemente TE, et al. Heterologous expression of a plastid EF-Tu reduces protein thermal aggregation and enhances CO(2) fixation in wheat (Triticum aestivum) following heat stress [J]. Plant Molecular Biology, 2008,68(3): 277-288.

[1132] Fu Q, Wang JR, Boerma M, et al. Involvement of heat shock factor 1 in statin-induced transcriptional upregulation of endothelial thrombomodulin [J]. Circulation Research, 2008,103(4): 369-377.

[1133] Fukuoka Y, Schwartz LB. Active monomers of human beta-tryptase have expanded substrate specificities [J]. International Immunopharmacology, 2007,7(14): 1900-1908.

[1134] Galvan EM, Lasaro MAS, Schifferli DM. Capsular antigen fraction 1 and pla modulate the susceptibility of Yersinia pestis to pulmonary antimicrobial peptides such as cathelicidin [J]. Infection and Immunity, 2008,76(4): 1456-1464.

[1135] Gamaly EG, Juodkazis S, Misawa H, et al. Formation of nano-voids in transparent dielectrics by femtosecond lasers [J]. Current Applied Physics, 2008,8(3-4): 412-415.

[1136] Gaultier A, Wu XH, Le Moan N, et al. Low-density lipoprotein receptor-related protein 1 is an essential receptor for myelin phagocytosis [J]. Journal of Cell Science, 2009,122(8): 1155-1162.

[1137] Gharaee-Kermani M, Hu B, Phan SH, et al. The role of urokinase in idiopathic pulmonary fibrosis and implication for therapy [J]. Expert Opinion on Investigational Drugs, 2008,17(6): 905-916.

[1138] Gibson BR, Sevransky JE. Is target population more important than patient location when evaluating tight glycemic control? [J]. Critical Care Medicine, 2009,37(2): 741-742.

[1139] Giebelen IAJ, Leendertse M, Dessing MC, et al. Endogenous beta-adrenergic receptors inhibit lipopolysaccharide-induced pulmonary cytokine release and coagulation [J]. American Journal of Respiratory Cell and Molecular Biology, 2008,39(3): 373-379.

[1140] Gimenez-Bastida JA, Martinez-Florensa M, Espin JC, et al. A Citrus Extract Containing Flavanones Represses Plasminogen Activator Inhibitor-1 (PAI-1) Expression and Regulates Multiple Inflammatory, Tissue Repair, and Fibrosis Genes in Human Colon Fibroblasts [J]. Journal of Agricultural and Food Chemistry, 2009,57(19): 9305-9315.

[1141] Goggs R, Benigni L, Fuentes VL, et al. Pulmonary thromboembolism [J]. Journal of Veterinary Emergency and Critical Care, 2009,19(1): 30-52.

[1142] Gonzalez A, Sagardia J, Redondo A, et al. Alveolar hemorrhage as a complication of thrombolytic therapy [J]. Medicina-Buenos Aires, 2011,71(6): 547-549.

[1143] Goodman SG, Cantor WJ. Drip-and-ship for acute ST-segment myocardial infarction The pharmacoinvasive strategy for patients treated with fibrinolytic therapy [J]. Polskie Archiwum Medycyny Wewnetrznej-Polish Archives of Internal Medicine, 2009,119(11): 726-730.

[1144] Greksova K, Parrak V, Chovancova D, et al. Procalcitonin, neopterin and C-reactive protein in diagnostics of intrauterine infection and preterm delivery [J]. Bratislava Medical Journal-Bratislavske Lekarske Listy, 2009,110(10): 623-626.

[1145] Groger M, Pasteiner W, Ignatyev G, et al. Peptide B beta(15-42) Preserves Endothelial Barrier Function in Shock [J]. Plos One, 2009,4(4).

[1146] Guo H, Singh I, Wang YM, et al. Neuroprotective activities of activated protein C mutant with reduced anticoagulant activity [J]. European Journal of Neuroscience, 2009,29(6): 1119-1130.

[1147] Haitsma JJ, Schultz MJ, Hofstra JJH, et al. Ventilator-induced coagulopathy in experimental Streptococcus pneumoniae pneumonia [J]. European Respiratory Journal, 2008,32(6): 1599-1606.

[1148] Hamm A, Veeck J, Bektas N, et al. Frequent expression loss of Inter-alpha-trypsin inhibitor heavy chain (ITIH) genes in multiple human solid tumors: A systematic expression analysis [J]. Bmc Cancer, 2008,8.

[1149] Han CK, Park YH, Jin DQ, et al. SK-PC-B70M from Pulsatilla koreana improves scopolamine-induced impairments of memory consolidation and spatial working memory [J]. Brain Research, 2007,1184: 254-259.

[1150] Han F, Chen H, Li XJ, et al. A comparative proteomic analysis of rice seedlings under various high-temperature stresses [J]. Biochimica Et Biophysica Acta-Proteins and Proteomics, 2009,1794(11): 1625-1634.

[1151] Harada A, Shiosaka S, Ishikawa Y, et al. Acute stress increases neuropsin mRNA expression in the mouse hippocampus through the glucocorticoid pathway [J]. Neuroscience Letters, 2008,436(2): 273-277.

[1152] Hartemink KJ, Hack CE, Groeneveld ABJ. Relation between coagulation/fibrinolysis and lactate in the course of human septic shock [J]. Journal of Clinical Pathology, 2010,63(11): 1021-1026.

[1153] Hayakawa M, Sawamura A, Gando S, et al. Disseminated intravascular coagulation at an early phase of trauma is associated with consumption coagulopathy and excessive fibrinolysis both by plasmin and neutrophil elastase [J]. Surgery, 2011,149(2): 221-230.

[1154] He HY, Wang C, Pang BS. Effects of activated protein C on coagulation and fibrinolysis in rabbits with endotoxin induced acute lung injury [J]. Chinese Medical Journal, 2008,121(24): 2561-2565.

[1155] He X, Han B, Mura M, et al. Anti-Human Tissue Factor Antibody Ameliorated Intestinal Ischemia Reperfusion-Induced Acute Lung Injury in Human Tissue Factor Knock-In Mice [J]. Plos One, 2008,3(1).

[1156] Hellerud BC, Nielsen EW, Thorgersen EB, et al. Dissecting the effects of lipopolysaccharides from nonlipopolysaccharide molecules in experimental porcine meningococcal sepsis [J]. Critical Care Medicine, 2010,38(6): 1467-1474.

[1157] Helling H, Schenk HJ, Pindur G, et al. Fibrinolytic and procoagulant activity in septic and haemorrhagic shock [J]. Clinical Hemorheology and Microcirculation, 2010,45(2-4): 295-300.

[1158] Hellstern P. Fresh-frozen plasma, pathogen-reduced single-donor plasma or bio-pharmaceutical plasma? [J]. Transfusion and Apheresis Science, 2008,39(1): 69-74.

[1159] Hellstern P, Solheim BG. The Use of Solvent/Detergent Treatment in Pathogen Reduction of Plasma [J]. Transfusion Medicine and Hemotherapy, 2011,38(1): 65-70.

[1160] Hemmelgarn BR, Moist LM, Lok CE, et al. Prevention of Dialysis Catheter Malfunction with Recombinant Tissue Plasminogen Activator [J]. New England Journal of Medicine, 2011,364(4): 303-312.

[1161] Hemmen TM, Raman R, Guluma KZ, et al. Intravenous Thrombolysis Plus Hypothermia for Acute Treatment of Ischemic Stroke (ICTuS-L) Final Results [J]. Stroke, 2010,41(10): 2265-2270.

[1162] Hendriks T, Bleichrodt RP, Lomme R, et al. Peritoneal Cytokines Predict Mortality after Surgical Treatment of Secondary Peritonitis in the Rat [J]. Journal of the American College of Surgeons, 2010,211(2): 263-270.

[1163] Herrmann HC, Lu JD, Brodie BR, et al. Benefit of Facilitated Percutaneous Coronary Intervention in High-Risk ST-Segment Elevation Myocardial Infarction Patients Presenting to Nonpercutaneous Coronary Intervention Hospitals [J]. Jacc-Cardiovascular Interventions, 2009,2(10): 917-924.

[1164] Hess JR, Brohi K, Dutton RP, et al. The Coagulopathy of Trauma: A Review of Mechanisms [J]. Journal of Trauma-Injury Infection and Critical Care, 2008,65(4): 748-754.

[1165] Hidaka S, Iwasaka H, Hagiwara S, et al. Gabexate Mesilate Inhibits the Expression of HMGB1 in Lipopolysaccharide-Induced Acute Lung Injury [J]. Journal of Surgical Research, 2011,165(1): 142-150.

[1166] Hirabayashi K, Shiohara M, Saito S, et al. Polymyxin-Direct Hemoperfusion for Sepsis-Induced Multiple Organ Failure [J]. Pediatric Blood & Cancer, 2010,55(1): 202-205.

[1167] Hirano T, Nakagawa A, Ohki T, et al. A Laser-induced Liquid Jet Catheter System: A Novel Endovascular Device for Rapid and Reliable Fibrinolysis in Acute Cerebral Embolism [J]. Minimally Invasive Neurosurgery, 2008,51(6): 324-328.

[1168] Hofstra JJH, Haitsma JJ, Juffermans NP, et al. The Role of Bronchoalveolar Hemostasis in the Pathogenesis of Acute Lung Injury [J]. Seminars in Thrombosis and Hemostasis, 2008,34(5): 475-484.

[1169] Hoogerwerf JJ, de Vos AF, Levi M, et al. Activation of coagulation and inhibition of fibrinolysis in the human lung on bronchial instillation of lipoteichoic acid and lipopolysaccharide [J]. Critical Care Medicine, 2009,37(2): 619-625.

[1170] Horowitz JC, Rogers DS, Simon RH, et al. Plasminogen activation-induced pericellular fibronectin proteolysis promotes fibroblast apoptosis [J]. American Journal of Respiratory Cell and Molecular Biology, 2008,38(1): 78-87.

[1171] Hsieh YS, Yang SF, Lue KH, et al. Effects of different molecular weight hyaluronan products on the expression of urokinase plasminogen activator and inhibitor and gelatinases during the early stage of osteoarthritis [J]. Journal of Orthopaedic Research, 2008,26(4): 475-484.

[1172] Hsu TC, Chen YC, Lai WX, et al. Beneficial effects of treatment with cystamine on brain in NZB/W F1 mice [J]. European Journal of Pharmacology, 2008,591(1-3): 307-314.

[1173] Huang XG, Fu SZ, Shu H, et al. Experimental study on shock compression properties of polyethylene [J]. Acta Physica Sinica, 2010,59(9): 6394-6398.

[1174] Hupkova H, Trupl J, Stankovic I, et al. Microbiological and therapeutical aspects of pneumococcal diseases in the Slovak Republic [J]. Bratislava Medical Journal-Bratislavske Lekarske Listy, 2010,111(7): 404-409.

[1175] Hutan M, Hutan M, Payer J. Selected indicators of care in patients with acute pancreatitis in the Slovak Republic [J]. Bratislava Medical Journal-Bratislavske Lekarske Listy, 2010,111(11): 599-603.

[1176] Idell S. The pathogenesis of pleural space loculation and fibrosis [J]. Current Opinion in Pulmonary Medicine, 2008,14(4): 310-315.

[1177] Ince DA, Atac FB, Ozkiraz S, et al. The Role of Plasminogen Activator Inhibitor-1 and Angiotensin-Converting Enzyme Gene Polymorphisms in Bronchopulmonary Dysplasia [J]. Genetic Testing and Molecular Biomarkers, 2010,14(5): 643-647.

[1178] Itagaki K, Zhang Q, Hauser CJ. SPHINGOSINE KINASE INHIBITION ALLEVIATES ENDOTHELIAL PERMEABILITY INDUCED BY THROMBIN AND ACTIVATED NEUTROPHILS [J]. Shock, 2010,33(4): 381-386.

[1179] Ito M, Kase H, Shimoyama O, et al. Effects of Polymyxin B-Immobilized Fiber Using a Rat Cecal Ligation and Perforation Model [J]. Asaio Journal, 2009,55(3): 246-250.

[1180] Iwamoto S, Takasu A, Sakamoto T. Therapeutic Mild Hypothermia: Effects on Coagulopathy and Survival in a Rat Hemorrhagic Shock Model [J]. Journal of Trauma-Injury Infection and Critical Care, 2010,68(3): 669-675.

[1181] Jablonska E, Markart P, Zakrzewicz D, et al. Transforming Growth Factor-beta 1 Induces Expression of Human Coagulation Factor XII via Smad3 and JNK Signaling Pathways in Human Lung Fibroblasts [J]. Journal of Biological Chemistry, 2010,285(15): 11638-11651.

[1182] Jaffe A, Calder AD, Owens CM, et al. Role of routine computed tomography in paediatric pleural empyema [J]. Thorax, 2008,63(10): 897-902.

[1183] Jalkanen V, Yang R, Linko R, et al. PROGNOSTIC VALUE OF SOLUBLE UROKINASE PLASMINOGEN ACTIVATOR RECEPTOR (SUPAR) AND PLASMINOGEN ACTIVATOR INHIBITOR 1 (PAI-1) LEVELS IN CRITICALLY ILL PATIENTS WITH RESPIRATORY FAILURE [J]. Intensive Care Medicine, 2010,36: S353-S353.

[1184] Jang JY, Jeon YK, Kim CW. Degradation of HER2/neu by ANT2 shRNA suppresses migration and invasiveness of breast cancer cells [J]. Bmc Cancer, 2010,10.

[1185] Janin S, Meneveau N, Mahemuti A, et al. Safety and efficacy of fondaparinux as an adjunctive treatment to thrombolysis in patients with high and intermediate risk pulmonary embolism [J]. Journal of Thrombosis and Thrombolysis, 2009,28(3): 320-324.

[1186] Jansen MC, van Hillegersberg R, Schoots IG, et al. Cryoablation induces greater inflammatory and coagulative responses than radiofrequency ablation or laser induced thermotherapy in a rat liver model [J]. Surgery, 2010,147(5): 686-695.

[1187] Janssens L, Van Haesendonck C, Van Boven AJ, et al. Early treatment of ST elevation myocardial infarction with abciximab or with abciximab and reteplase reduces the incidence of cardiogenic shock: results of the FINESSE trial [J]. European Heart Journal, 2008,29: 136-136.

[1188] Jarai R, Huber K, Bogaerts K, et al. Plasma N-terminal fragment of the prohormone B-type natriuretic peptide concentrations in relation to time to treatment and Thrombolysis in Myocardial Infarction (TIMI) flow: A substudy of the Assessment of the Safety and Efficacy of a New Treatment Strategy with Percutaneous Coronary Intervention (ASSENT IV-PCI) trial [J]. American Heart Journal, 2010,159(1): 131-140.

[1189] Jarai R, Huber K, Bogaerts K, et al. Prediction of cardiogenic shock using plasma B-type natriuretic peptide and the N-terminal fragment of its pro-hormone concentrations in ST elevation myocardial infarction: An analysis from the ASSENT-4 Percutaneous Coronary Intervention Trial [J]. Critical Care Medicine, 2010,38(9): 1793-1801.

[1190] Jenkins G. The role of proteases in transforming growth factor-beta activation [J]. International Journal of Biochemistry & Cell Biology, 2008,40(6-7): 1068-1078.

[1191] Jenkins PO, Sultanzadeh J, Bhagwat M, et al. Should thrombolysis have a greater role in the management of pulmonary embolism? [J]. Clinical Medicine, 2009,9(5): 431-435.

[1192] Jesmin S, Gando S, Zaedi S, et al. PROTEASE-ACTIVATED RECEPTOR 2 BLOCKING PEPTIDE COUNTERACTS ENDOTOXIN-INDUCED INFLAMMATION AND COAGULATION AND AMELIORATES RENAL FIBRIN DEPOSITION IN A RAT MODEL OF ACUTE RENAL FAILURE [J]. Shock, 2009,32(6): 626-632.

[1193] Jiang JS, Chou HC, Wang LF, et al. Effects of Activated Protein C on Ventilator-Induced Lung Injury in Rats [J]. Respiration, 2010,80(3): 246-253.

[1194] Johansson PI, Sorensen AM, Perner A, et al. Disseminated intravascular coagulation or acute coagulopathy of trauma shock early after trauma? An observational study [J]. Critical Care, 2011,15(6).

[1195] Kale S, Yende S, Kong L, et al. The Effects of Age on Inflammatory and Coagulation-Fibrinolysis Response in Patients Hospitalized for Pneumonia [J]. Plos One, 2010,5(11).

[1196] Kalra N, Bhui K, Roy P, et al. Regulation of p53, nuclear factor KB and cyclooxygenase-2 expression by bromelain through targeting mitogen-activated protein kinase pathway in mouse skin [J]. Toxicology and Applied Pharmacology, 2008,226(1): 30-37.

[1197] Kang JS, Lee KH, Han MH, et al. Antiinflammatory activity of methanol extract isolated from stem bark of Magnolia kobus [J]. Phytotherapy Research, 2008,22(7): 883-888.

[1198] Kang K, Kim H, Il Kim K, et al. SK-126, a synthetic compound, regulates the production of inflammatory cytokines induced by LPS in antigen-presenting cells [J]. Biochemical Pharmacology, 2008,75(5): 1054-1064.

[1199] Kang SH, Kang KW, Kim KH, et al. Upregulated HSP27 in human breast cancer cells reduces Herceptin susceptibility by increasing Her2 protein stability [J]. Bmc Cancer, 2008,8.

[1200] Kang YH, Ji NY, Il Lee C, et al. ESM-1 silencing decreased cell survival, migration, and invasion and modulated cell cycle progression in hepatocellular carcinoma [J]. Amino Acids, 2011,40(3): 1003-1013.

[1201] Kanse SM, Parahuleva M, Muhl L, et al. Factor VII-activating protease (FSAP): Vascular functions and role in atherosclerosis [J]. Thrombosis and Haemostasis, 2008,99(2): 286-289.

[1202] Kaplan AP. Enzymatic pathways in the pathogenesis of hereditary angioedema: The role of C1 inhibitor therapy [J]. Journal of Allergy and Clinical Immunology, 2010,126(5): 918-925.

[1203] Kaplan AP, Ghebrehiwet B. The plasma bradykinin-forming pathways and its interrelationships with complement [J]. Molecular Immunology, 2010,47(13): 2161-2169.

[1204] Kasetty G, Papareddy P, Kalle M, et al. The C-Terminal Sequence of Several Human Serine Proteases Encodes Host Defense Functions [J]. Journal of Innate Immunity, 2011,3(5): 471-482.

[1205] Kasperczuk A, Pisarczyk T, Gus'kov SY, et al. Laser energy transformation to shock waves in multi-layer flyers [J]. Radiation Effects and Defects in Solids, 2008,163(4-6): 519-533.

[1206] Kasperczuk A, Pisarczyk T, Kalal M, et al. PALS laser energy transfer into solid targets and its dependence on the lens focal point position with respect to the target surface [J]. Laser and Particle Beams, 2008,26(2): 189-196.

[1207] Kavazis AN, Smuder AJ, Min K, et al. Short-term exercise training protects against doxorubicin-induced cardiac mitochondrial damage independent of HSP72 [J]. American Journal of Physiology-Heart and Circulatory Physiology, 2010,299(5): H1515-H1524.

[1208] Kawa K, Tsutsui H, Uchiyama R, et al. IFN-gamma is a master regulator of endotoxin shock syndrome in mice primed with heat-killed Propionibacterium acnes [J]. International Immunology, 2010,22(3): 157-166.

[1209] Kawagoe S, Yoshida T, Kajino T, et al. Neutrino oscillation and expected event rate of supernova neutrinos in the adiabatic explosion model [J]. Physical Review D, 2010,81(12).

[1210] Kerachian MA, Cournoyer D, Harvey EJ, et al. Effect of high-dose dexamethasone on endothelial haemostatic gene expression and neutrophil adhesion [J]. Journal of Steroid Biochemistry and Molecular Biology, 2009,116(3-5): 127-133.

[1211] Kern L, Robert J, Brutsche M. Management of Parapneumonic Effusion and Empyema: Medical Thoracoscopy and Surgical Approach [J]. Respiration, 2011,82(2): 193-196.

[1212] Kethireddy S, Safdar N. Urokinase lock or flush solution for prevention of bloodstream infections associated with central venous catheters for chemotherapy: a meta-analysis of prospective randomized trials [J]. Journal of Vascular Access, 2008,9(1): 51-57.

[1213] Kim M, Park SW, Kim M, et al. Isoflurane activates intestinal sphingosine kinase to protect against bilateral nephrectomy-induced liver and intestine dysfunction [J]. American Journal of Physiology-Renal Physiology, 2011,300(1): F167-F176.

[1214] Kim M, Park SW, Kim M, et al. Isoflurane Activates Intestinal Sphingosine Kinase to Protect against Renal Ischemia-Reperfusion-induced Liver and Intestine Injury [J]. Anesthesiology, 2011,114(2): 363-373.

[1215] Kingsmore SF, Kennedy N, Halliday HL, et al. Identification of Diagnostic Biomarkers for Infection in Premature Neonates [J]. Molecular & Cellular Proteomics, 2008,7(10): 1863-1875.

[1216] Klak M, Anakkala N, Wang WZ, et al. Tranexamic acid, an inhibitor of plasminogen activation, aggravates staphylococcal septic arthritis and sepsis [J]. Scandinavian Journal of Infectious Diseases, 2010,42(5): 351-358.

[1217] Kline JA, Steuerwald MT, Marchick MR, et al. Prospective Evaluation of Right Ventricular Function and Functional Status 6 Months After Acute Submassive Pulmonary Embolism Frequency of Persistent or Subsequent Elevation in Estimated Pulmonary Artery Pressure [J]. Chest, 2009,136(5): 1202-1210.

[1218] Knudson MD, Desjarlais MP. Shock Compression of Quartz to 1.6 TPa: Redefining a Pressure Standard [J]. Physical Review Letters, 2009,103(22).

[1219] Kobashi Y, Mouri K, Yagi S, et al. Clinical Analysis of Cases of Empyema Due to Streptococcus milleri Group [J]. Japanese Journal of Infectious Diseases, 2008,61(6): 484-486.

[1220] Koca V, Ari H. Angioplasty as early revascularization in acute myocardial infarction [J]. Anadolu Kardiyoloji Dergisi-the Anatolian Journal of Cardiology, 2008,8: 77-83.

[1221] Kocaeli H, Zuccarello M. Management of spontaneous intracerebral hematomas - Review of the literature [J]. Neurosurgery Quarterly, 2008,18(3): 207-215.

[1222] Koch A, Tacke F. Why high suPAR is not super - diagnostic, prognostic and potential pathogenic properties of a novel biomarker in the ICU [J]. Critical Care, 2011,15(6).

[1223] Koch A, Voigt S, Kruschinski C, et al. Circulating soluble urokinase plasminogen activator receptor is stably elevated during the first week of treatment in the intensive care unit and predicts mortality in critically ill patients [J]. Critical Care, 2011,15(1).

[1224] Koegelenberg CFN, Diacon AH, Bolliger CT. Parapneumonic pleural effusion and empyema [J]. Respiration, 2008,75(3): 241-250.

[1225] Kofoed K, Eugen-Olsen J, Petersen J, et al. Predicting mortality in patients with systemic inflammatory response syndrome: an evaluation of two prognostic models, two soluble receptors, and a macrophage migration inhibitory factor [J]. European Journal of Clinical Microbiology & Infectious Diseases, 2008,27(5): 375-383.

[1226] Kolte D, Bryant JW, Holsworth D, et al. Biochemical characterization of a novel high-affinity and specific plasma kallikrein inhibitor [J]. British Journal of Pharmacology, 2011,162(7): 1639-1649.

[1227] Komissarov AA, Andreasen PA, Declerck PJ, et al. Redirection of the reaction between activated protein C and a serpin to the substrate pathway [J]. Thrombosis Research, 2008,122(3): 397-404.

[1228] Konecny FA. A model of massive pulmonary embolism, development and characterization - The pre-clinical steps forward and details of the progress [J]. Journal of Research in Medical Sciences, 2008,13(3): 121-134.

[1229] Kong M, Olman MA. Urokinase Receptor Is Increased in Pediatric and Adult Acute Lung Injury [J]. American Journal of Respiratory and Critical Care Medicine, 2009,179.

[1230] Konstantinides SV. Massive Pulmonary Embolism: What Level of Aggression? [J]. Seminars in Respiratory and Critical Care Medicine, 2008,29(1): 47-55.

[1231] Korkmaz SD, Korkmaz S. A study for structure and inter-diffusion coefficient of liquid K1-xCsx metal alloys [J]. Physics and Chemistry of Liquids, 2011,49(6): 801-810.

[1232] Koseki M, Asada N, Uryu H, et al. Successful combined use of tranexamic acid and unfractionated heparin for life-threatening bleeding associated with intravascular coagulation in a patient with chronic myelogenous leukemia in blast crisis [J]. International Journal of Hematology, 2007,86(5): 403-406.

[1233] Koshimizu T, Tsuchiya H, Tsuda H, et al. Inhibition of heat shock protein 90 attenuates adenylate cyclase sensitization after chronic morphine treatment [J]. Biochemical and Biophysical Research Communications, 2010,392(4): 603-607.

[1234] Kostrubiec M, Nieweglowska N, Pruszczyk P. Diagnosis and treatment of pulmonary embolism in pregnancy [J]. Ginekologia Polska, 2010,81(4): 283-286.

[1235] Krechetova AV, Galstyan GM, Vasilyev SA. BLOOD CLOTTING SYSTEM IN SEPSIS [J]. Gematologiya I Transfuziologiya, 2010,55(5): 20-34.

[1236] Kristofova B, Oetterova M, Valocikova I, et al. Successful therapy with intravenous immunoglobulin in the management of polymyositis [J]. Bratislava Medical Journal-Bratislavske Lekarske Listy, 2008,109(9): 412-413.

[1237] Kuiper JW, Vaschetto R, Della Corte F, et al. Bench-to-bedside review: Ventilation-induced renal injury through systemic mediator release - just theory or a causal relationship? [J]. Critical Care, 2011,15(4).

[1238] Kujanik SS, Mikulecky SM. Circa(ultra)dians of single extrasystoles in chronic respiratory failure versus health at lowland [J]. Bratislava Medical Journal-Bratislavske Lekarske Listy, 2010,111(6): 321-324.

[1239] Kuka P, Bucova M, Penz P, et al. HSP60, oxidative stress parameters and cardiometabolic risk markers in hypertensive and normotensive Slovak females [J]. Bratislava Medical Journal-Bratislavske Lekarske Listy, 2010,111(10): 527-534.

[1240] Kula R, Chylek V, Szturz P, et al. A response to infection in patients with severe sepsis - do we need a "Stage-Directed Therapy Concept"? [J]. Bratislava Medical Journal-Bratislavske Lekarske Listy, 2009,110(8): 459-464.

[1241] Kula R, Maca J, Sklienka P, et al. Exogenous surfactant as a component of complex non-ECMO therapy of ARDS caused by influenza A virus (2009 H1N1) [J]. Bratislava Medical Journal-Bratislavske Lekarske Listy, 2011,112(4): 218-222.

[1242] Kuo WT, van den Bosch M, Hofmann LV, et al. Catheter-directed embolectomy, fragmentation, and thrombolysis for the treatment of massive pulmonary embolism after failure of systemic thrombolysis [J]. Chest, 2008,134(2): 250-254.

[1243] Kushi H, Miki T, Sakagami Y, et al. Hemoperfusion With a Polymyxin B Fiber Column Decreases Clotting Activity [J]. Therapeutic Apheresis and Dialysis, 2009,13(6): 528-533.

[1244] Kwiecinski J, Josefsson E, Mitchell J, et al. Activation of Plasminogen by Staphylokinase Reduces the Severity of Staphylococcus aureus Systemic Infection [J]. Journal of Infectious Diseases, 2010,202(7): 1041-1049.

[1245] Lackowski NP, Pitzer JE, Tobias M, et al. Safety of prolonged, repeated administration of a pulmonary formulation of tissue plasminogen activator in mice [J]. Pulmonary Pharmacology & Therapeutics, 2010,23(2): 107-114.

[1246] Lang CH, Frost RA, Bronson SK, et al. Skeletal muscle protein balance in mTOR heterozygous mice in response to inflammation and leucine [J]. American Journal of Physiology-Endocrinology and Metabolism, 2010,298(6): E1283-E1294.

[1247] Langley JM, Kellner JD, Solomon N, et al. Empyema associated with community-acquired pneumonia: A Pediatric Investigator's Collaborative Network on Infections in Canada (PICNIC) study [J]. Bmc Infectious Diseases, 2008,8.

[1248] Langouche L, Meersseman W, Perre SV, et al. Effect of insulin therapy on coagulation and fibrinolysis in medical intensive care patients [J]. Critical Care Medicine, 2008,36(5): 1475-1480.

[1249] Lankeit M, Konstantinides S. Thrombolysis for pulmonary embolism: Past, present and future [J]. Thrombosis and Haemostasis, 2010,103(5): 877-883.

[1250] Lapchak PA. Efficacy and safety profile of the carotenoid trans sodium crocetinate administered to rabbits following multiple infarct ischemic strokes: A combination therapy study with tissue plasminogen activator [J]. Brain Research, 2010,1309: 136-145.

[1251] Larmann J, Frenzel T, Hahnenkamp A, et al. In Vivo Fluorescence-mediated Tomography for Quantification of Urokinase Receptor-dependent Leukocyte Trafficking in Inflammation [J]. Anesthesiology, 2010,113(3): 610-618.

[1252] Laterre PF, Macias WL, Janes J, et al. Influence of enrollment sequence effect on observed outcomes in the ADDRESS and PROWESS studies of drotrecogin alfa (activated) in patients with severe sepsis [J]. Critical Care, 2008,12(5).

[1253] Lau CL, Zhao YG, Kim J, et al. Enhanced fibrinolysis protects against lung ischemia-reperfusion injury [J]. Journal of Thoracic and Cardiovascular Surgery, 2009,137(5): 1241-1248.

[1254] Lavrentieva A, Kontakiotis T, Bitzani M, et al. Early coagulation disorders after severe burn injury: impact on mortality [J]. Intensive Care Medicine, 2008,34(4): 700-706.

[1255] Lavrentieva A, Kontakiotis T, Bitzani M, et al. The efficacy of antithrombin administration in the acute phase of burn injury [J]. Thrombosis and Haemostasis, 2008,100(2): 286-290.

[1256] Leal JL, Lora P, Enciso G, et al. Massive pulmonary thromboembolism treated successfully with streptokinase. Report of one case [J]. Revista Medica De Chile, 2010,138(7): 856-861.

[1257] Lee JY, Lee BS, Kim JY, et al. Heat Shock Protein 27 overexpression prevents Ox-LDL induced anoikis by inhibiting the expression of inflammatory cytokines and tissue-type plasminogen activator in vascular smooth muscle cells [J]. Journal of the American College of Cardiology, 2008,51(10): A344-A344.

[1258] Lee SF, Lawrence D, Booth H, et al. Thoracic empyema: current opinions in medical and surgical management [J]. Current Opinion in Pulmonary Medicine, 2010,16(3): 194-200.

[1259] Lee Y, Paik D, Bang S, et al. Loss of spastic paraplegia gene atlastin induces age-dependent death of dopaminergic neurons in Drosophila [J]. Neurobiology of Aging, 2008,29(1): 84-94.

[1260] Leo JC, Skurnik M. Adhesins of Human Pathogens from the Genus Yersinia[A]. In: Bacterial Adhesion: Chemistry, Biology and Physics (Linke D, Goldman A, eds), Vol. 715, 2011: 1-15.

[1261] Levi M. Disseminated intravascular coagulation in cancer patients [J]. Best Practice & Research Clinical Haematology, 2009,22(1): 129-136.

[1262] Levi M, Schultz M, van der Poll T. Disseminated Intravascular Coagulation in Infectious Disease [J]. Seminars in Thrombosis and Hemostasis, 2010,36(4): 367-377.

[1263] Levi M, Schultz M, van der Poll T. Coagulation Biomarkers in Critically Ill Patients [J]. Critical Care Clinics, 2011,27(2): 281-+.

[1264] Li LF, Huang CC, Lin HC, et al. Unfractionated heparin and enoxaparin reduce high-stretch ventilation augmented lung injury: a prospective, controlled animal experiment [J]. Critical Care, 2009,13(4).

[1265] Liangos O, Jaber BL. Multiple organ dysfunction syndrome in children with sepsis: Role of genetic factors [J]. Seminars in Nephrology, 2008,28(5): 499-509.

[1266] Liao WP, Bao Z, Cheng C, et al. Dendritic cell-derived interferon-gamma-induced protein mediates tumor necrosis factor-alpha stimulation of human lung fibroblasts [J]. Proteomics, 2008,8(13): 2640-2650.

[1267] Libungan BG, Eyjolfsson K, Porgeirsson G. Primary percutaneous coronary interventions in Iceland [J]. Laeknabladid, 2008,94(2): 103-107.

[1268] Lillis AP, Van Duyn LB, Murphy-Ullrich JE, et al. LDL receptor-related protein 1: Unique tissue-specific functions revealed by selective gene knockout studies [J]. Physiological Reviews, 2008,88(3): 887-918.

[1269] Lim KM, Lee JY, Lee SM, et al. Potent anti-inflammatory effects of two quinolinedione compounds, OQ1 and OQ21, mediated by dual inhibition of inducible NO synthase and cyclooxygenase-2 [J]. British Journal of Pharmacology, 2009,156(2): 328-337.

[1270] Lindholm MG, Boesgaard S, Thune JJ, et al. Percutaneous coronary intervention for acute MI does not prevent in-hospital development of cardiogenic shock compared to fibrinolysis [J]. European Journal of Heart Failure, 2008,10(7): 668-674.

[1271] Liu K, Mori S, Takahashi HK, et al. Anti-high mobility group box 1 monoclonal antibody ameliorates brain infarction induced by transient ischemia in rats [J]. Faseb Journal, 2007,21(14): 3904-3916.

[1272] Liu KD, Glidden DV, Eisner MD, et al. Predictive and pathogenetic value of plasma biomarkers for acute kidney injury in patients with acute lung injury [J]. Critical Care Medicine, 2007,35(12): 2755-2761.

[1273] Liu RM. Oxidative stress, plasminogen activator inhibitor 1, and lung fibrosis [J]. Antioxidants & Redox Signaling, 2008,10(2): 303-319.

[1274] Liu RM, Pravia KAG. Oxidative stress and glutathione in TGF-beta-mediated fibrogenesis [J]. Free Radical Biology and Medicine, 2010,48(1): 1-15.

[1275] Liu YY, Liao SK, Huang CC, et al. Role for nuclear factor-kappa B in augmented lung injury because of interaction between hyperoxia and high stretch ventilation [J]. Translational Research, 2009,154(5): 228-240.

[1276] Looney MR, Esmon CT, Matthay MA. Role of coagulation pathways and treatment with activated protein C in hyperoxic lung injury [J]. Thorax, 2009,64(2): 114-120.

[1277] MacRae JM, Dojcinovic L, Djurdjev O, et al. Citrate 4% versus heparin and the reduction of thrombosis study (CHARTS) [J]. Clinical Journal of the American Society of Nephrology, 2008,3(2): 369-374.

[1278] Madach K, Aladzsity I, Szilagyi A, et al. 4G/5G polymorphism of PAI-1 gene is associated with multiple organ dysfunction and septic shock in pneumonia induced severe sepsis: prospective, observational, genetic study [J]. Critical Care, 2010,14(2).

[1279] Madoiwa S, Tanaka H, Nagahama Y, et al. Degradation of cross-linked fibrin by leukocyte elastase as alternative pathway for plasmin-mediated fibrinolysis in sepsis-induced disseminated intravascular coagulation [J]. Thrombosis Research, 2011,127(4): 349-355.

[1280] Mahmoodpoor A, Eslami K, Mojtahedzadeh M, et al. Examination of Setarud (IMOD (TM)) in the management of patients with severe sepsis [J]. Daru-Journal of Faculty of Pharmacy, 2010,18(1): 23-28.

[1281] Makinodan K, Itoh T, Tomoda K, et al. Acute pulmonary thromboembolism associated with interstitial pneumonia [J]. Internal Medicine, 2008,47(7): 647-650.

[1282] Mammen EF. Antithrombin: Its Physiological Importance and Role in DIC (Reprinted from Semin Thromb Hemost, vol 24, pg 19-25, 1998) [J]. Seminars in Thrombosis and Hemostasis, 2008,34(8): A22-A28.

[1283] Margheri M, Vittori G, Vecchio S, et al. Early and long-term clinical results of AngioJet rheolytic thrombectomy in patients with acute pulmonary embolism [J]. American Journal of Cardiology, 2008,101(2): 252-258.

[1284] Marshall SA, Nyquist P. A Change of Position for Neurogenic Pulmonary Edema [J]. Neurocritical Care, 2009,10(2): 213-217.

[1285] Mastroiacovo F, Busceti CL, Biagioni F, et al. Induction of the Wnt antagonist, Dickkopf-1, contributes to the development of neuronal death in models of brain focal ischemia [J]. Journal of Cerebral Blood Flow and Metabolism, 2009,29(2): 264-276.

[1286] Matic I, Titlic M, Lucic I, et al. Recombinant activated factor VII in refractory gastrointestinal haemorrhage of unknown aetiology [J]. Bratislava Medical Journal-Bratislavske Lekarske Listy, 2008,109(10): 438-440.

[1287] Mauri T, Bellani G, Patroniti N, et al. Persisting high levels of plasma pentraxin 3 over the first days after severe sepsis and septic shock onset are associated with mortality [J]. Intensive Care Medicine, 2010,36(4): 621-629.

[1288] Maya ID, Smith T, Allon M. Does the Heparin Lock Concentration Affect Hemodialysis Catheter Patency? [J]. Clinical Journal of the American Society of Nephrology, 2010,5(8): 1458-1462.

[1289] McBride SC. Management of parapneumonic effusions in pediatrics: Current practice [J]. Journal of Hospital Medicine, 2008,3(3): 263-270.

[1290] McClintock D, Zhuo HJ, Wickersham N, et al. Biomarkers of inflammation, coagulation and fibrinolysis predict mortality in acute lung injury [J]. Critical Care, 2008,12(2).

[1291] McKay RG, Dada MR, Mather JF, et al. Comparison of Outcomes and Safety of "Facilitated" Versus Primary Percutaneous Coronary Intervention in Patients With ST-Segment Elevation Myocardial Infarction [J]. American Journal of Cardiology, 2009,103(3): 316-321.

[1292] McWilliams RS, Eggert JH, Hicks DG, et al. Strength effects in diamond under shock compression from 0.1 to 1 TPa [J]. Physical Review B, 2010,81(1).

[1293] Meneveau N. Therapy for acute high-risk pulmonary embolism: thrombolytic therapy and embolectomy [J]. Current Opinion in Cardiology, 2010,25(6): 560-567.

[1294] Meng QW, Chen XS, Sun LC, et al. Carbamazepine promotes Her-2 protein degradation in breast cancer cells by modulating HDAC6 activity and acetylation of Hsp90 [J]. Molecular and Cellular Biochemistry, 2011,348(1-2): 165-171.

[1295] Metrailler-Ruchonnet I, Pagano A, Carnesecchi S, et al. Bcl-2 overexpression in type II epithelial cells does not prevent hyperoxia-induced acute lung injury in mice [J]. American Journal of Physiology-Lung Cellular and Molecular Physiology, 2010,299(3): L312-L322.

[1296] Meyer G, Sanchez O. Indications and modalities of fibrinolytic therapy for pulmonary embolism [J]. Sang Thrombose Vaisseaux, 2009,21(4): 181-185.

[1297] Mifkovic A, Skultety J, Pindak D, et al. Specific aspects of acute pancreatitis [J]. Bratislava Medical Journal-Bratislavske Lekarske Listy, 2009,110(9): 544-552.

[1298] Migliorini A, Valenti R, Moschi G, et al. Predictor of Stent Thrombosis in Patients Treated with Turbostratic Carbon-Coated Stent Implantation for Acute Myocardial Infarction [J]. Journal of Interventional Cardiology, 2010,23(6): 554-559.

[1299] Milbrandt EB, Reade MC, Lee M, et al. Prevalence and Significance of Coagulation Abnormalities in Community-Acquired Pneumonia [J]. Molecular Medicine, 2009,15(11-12): 438-445.

[1300] Mimuro J, Niimura M, Kashiwakura Y, et al. Unbalanced expression of ADAMTS13 and von Witlebrand factor in mouse endotoxinemia [J]. Thrombosis Research, 2008,122(1): 91-97.

[1301] Minami M, Kamimura T, Isaka M, et al. Clindamycin-Induced CovS-Mediated Regulation of the Production of Virulent Exoproteins Streptolysin O, NAD Glycohydrolase, and Streptokinase in Streptococcus pyogenes [J]. Antimicrobial Agents and Chemotherapy, 2010,54(1): 98-102.

[1302] Mogan MJ, Coley BD. Interventional radiology treatment of emplyema and bung abscesses [J]. Paediatric Respiratory Reviews, 2008,9(2): 77-84.

[1303] Mohan B, Mahajan V, Chhabra ST. Combined Modality of Mechanical Breakdown and Intraembolus Thrombolysis in Failed Systemic Thrombolysis of Subacute Pulmonary Embolism Patients [J]. Journal of Interventional Cardiology, 2010,23(5): 479-484.

[1304] Montaner J, Chacon P, Krupinski J, et al. Simvastatin in the acute phase of ischemic stroke: a safety and efficacy pilot trial [J]. European Journal of Neurology, 2008,15(1): 82-90.

[1305] Moos PJ, Olszewski K, Honeggar M, et al. Responses of human cells to ZnO nanoparticles: a gene transcription study [J]. Metallomics, 2011,3(11): 1199-1211.

[1306] Moreau P, Flajollet S, Carosella ED. Non-classical transcriptional regulation of HLA-G: an update [J]. Journal of Cellular and Molecular Medicine, 2009,13(9B): 2973-2989.

[1307] Moreno-Millan E, Castarnado-Calvo M, Moreno-Cano S, et al. Recurrent refractory ventricular fibrillation: How many times is it necessary to defibrillate? [J]. Medicina Intensiva, 2010,34(3): 215-218.

[1308] Murinello A, Figeiredo AM, Semedo J, et al. Thoracic empyema - A review based on three cases reports [J]. Revista Portuguesa De Pneumologia, 2009,15(3): 507-519.

[1309] Namath A, Patterson AJ. Genetic Polymorphisms in Sepsis [J]. Critical Care Clinics, 2009,25(4): 835-+.

[1310] Naples RM, Harris JW, Ghaemmaghami CA. Critical care aspects in the management of patients with acute coronary syndromes [J]. Emergency Medicine Clinics of North America, 2008,26(3): 685-+.

[1311] Nellis WJ. Water at high dynamic pressures and temperatures [J]. Zeitschrift Fur Naturforschung Section B-a Journal of Chemical Sciences, 2008,63(6): 605-607.

[1312] Ngow HA, Khairina W. A Case of Successful Thrombolysis in Pulmonary Embolism with Tenecteplase during Peripartum Period [J]. Acta Cardiologica Sinica, 2011,27(1): 52-55.

[1313] Nguyen TC, Han YY, Kiss JE, et al. Intensive plasma exchange increases a disintegrin and metalloprotease with thrombospondin motifs-13 activity and reverses organ dysfunction in children with thrombocytopenia-associated multiple organ failure [J]. Critical Care Medicine, 2008,36(10): 2878-2887.

[1314] Nishikawa T, Yoshida A, Khanal A, et al. A study of the efficacy of ultrasonic waves in removing biofilms [J]. Gerodontology, 2010,27(3): 199-206.

[1315] Nyberg A, Jakob SM, Seeman-Lodding H, et al. Time- and dose-related regional fluxes of tissue-type plasminogen activator in anesthetized endotoxemic pigs [J]. Acta Anaesthesiologica Scandinavica, 2008,52(1): 57-64.

[1316] O'Brien JM, Ali NA, Aberegg SK, et al. Sepsis [J]. American Journal of Medicine, 2007,120(12): 1012-1022.

[1317] O'Connor E, Fraser JF. How can we prevent and treat cardiogenic shock in patients who present to non-tertiary hospitals with myocardial infarction? A systematic review [J]. Medical Journal of Australia, 2009,190(8): 440-445.

[1318] Ohbayashi T, Irie A, Murakami Y, et al. Degradation of fibrinogen and collagen by staphopains, cysteine proteases released from Staphylococcus aureus [J]. Microbiology-Sgm, 2011,157: 786-792.

[1319] Onder AM, Chandar J, Billings A, et al. Prophylaxis of catheter-related bacteremia using tissue plasminogen activator-tobramycin locks [J]. Pediatric Nephrology, 2009,24(11): 2233-2243.

[1320] Onder AM, Chandar J, Simon N, et al. Treatment of catheter-related bacteremia with tissue plasminogen activator antibiotic locks [J]. Pediatric Nephrology, 2008,23(3): 457-464.

[1321] Oram J, Bodenham A. Hypothesis: Is the skin and tissue loss associated with septic purpura fulminas temperature related? Parallels with the anatomical distribution of frostbite [J]. Medical Hypotheses, 2008,70(6): 1155-1159.

[1322] Ozdemir L. Unilateral Acute Pulmonary Edema Due To Streptokinase Hypersensitivity: Letter to the Editor [J]. Turkiye Klinikleri Tip Bilimleri Dergisi, 2010,30(3): 1123-1124.

[1323] Park JH, Jong HS, Kim SG, et al. Inhibitors of histone deacetylases induce tumor-selective cytotoxicity through modulating Aurora-A kinase [J]. Journal of Molecular Medicine-Jmm, 2008,86(1): 117-128.

[1324] Park SK, Amos L, Rao A, et al. Identification and characterization of a novel ABCA3 mutation [J]. Physiological Genomics, 2010,40(2): 94-99.

[1325] Park YJ, Liu G, Lorne EF, et al. PAI-1 inhibits neutrophil efferocytosis [J]. Proceedings of the National Academy of Sciences of the United States of America, 2008,105(33): 11784-11789.

[1326] Park YJ, Liu G, Tsuruta Y, et al. Participation of the urokinase receptor in neutrophil efferocytosis [J]. Blood, 2009,114(4): 860-870.

[1327] Pasquariello N, Catanzaro G, Marzano V, et al. Characterization of the Endocannabinoid System in Human Neuronal Cells and Proteomic Analysis of Anandamide-induced Apoptosis [J]. Journal of Biological Chemistry, 2009,284(43): 29413-29426.

[1328] Patel N, Sundaram N, Yang MY, et al. Placenta Growth Factor (PlGF), a Novel Inducer of Plasminogen Activator Inhibitor-1 (PAI-1) in Sickle Cell Disease (SCD) [J]. Journal of Biological Chemistry, 2010,285(22): 16713-16722.

[1329] Pedron L, Baldi P, Hietala AM, et al. Genotype-specific regulation of cold-responsive genes in cypress (Cupressus sempervirens L.) [J]. Gene, 2009,437(1-2): 45-53.

[1330] Peng YH, Reyes JL, Wei H, et al. RcDhn5, a cold acclimation-responsive dehydrin from Rhododendron catawbiense rescues enzyme activity from dehydration effects in vitro and enhances freezing tolerance in RcDhn5-overexpressing Arabidopsis plants [J]. Physiologia Plantarum, 2008,134(4): 583-597.

[1331] Pernes JM, Auguste M. Acute lower limb ischaemia and endovascular techniques: why, for whom, how? [J]. Sang Thrombose Vaisseaux, 2010,22(3): 114-125.

[1332] Peterson DB, Sander T, Kaul S, et al. Comparative proteomic analysis of PAI-1 and TNF-alpha-derived endothelial microparticles [J]. Proteomics, 2008,8(12): 2430-2446.

[1333] Pfeilschifter W, Czech B, Hoffmann BP, et al. Pyrrolidine Dithiocarbamate Activates p38 MAPK and Protects Brain Endothelial Cells From Apoptosis: A Mechanism for the Protective Effect in Stroke? [J]. Neurochemical Research, 2010,35(9): 1391-1401.

[1334] Piazza G, Goldhaber SZ. Fibrinolysis for acute pulmonary embolism [J]. Vascular Medicine, 2010,15(5): 419-428.

[1335] Picard E, Joseph L, Goldberg S, et al. Predictive Factors of Morbidity in Childhood Parapneumonic Effusion-associated Pneumonia A Retrospective Study [J]. Pediatric Infectious Disease Journal, 2010,29(9): 840-843.

[1336] Pickard CJ, Needs RJ. Aluminium at terapascal pressures [J]. Nature Materials, 2010,9(8): 624-627.

[1337] Pilav I, Guska S, Musanovic S, et al. Pathogens of Pleural Empyema in patients treated on the Clinic for thoracic surgery of UKCS [J]. Healthmed, 2011,5(5): 1344-1348.

[1338] Ploppa A, Unertl KE, Nohe B. Thrombelastometry-guided thrombolytic therapy in massive pulmonary artery embolism [J]. Acta Anaesthesiologica Scandinavica, 2010,54(9): 1145-1148.

[1339] Pneumatikos I, Bouros D. Pleural effusions in critically ill patients [J]. Respiration, 2008,76(3): 241-248.

[1340] Poggi M, Paulmyer-Lacroix O, Verdier M, et al. Chronic plasminogen activator inhibitor-1 (PAI-1) overexpression dampens CD25+lymphocyte recruitment after lipopolysaccharide endotoxemia in mouse lung [J]. Journal of Thrombosis and Haemostasis, 2007,5(12): 2467-2475.

[1341] Polli F, Savioli M, Cugno M, et al. Effects of recombinant human activated protein C the fibrinolytic system of patients undergoing conventional or tight glycemic control [J]. Minerva Anestesiologica, 2009,75(7-8): 417-426.

[1342] Poonai N, Kornecki A, Buffo I, et al. Neonatal myocardial infarction secondary to umbilical venous catheterization: A case report and review of the literature [J]. Paediatrics & Child Health, 2009,14(8): 539-541.

[1343] Porcel JM, Light RW. Parapneumonic pleural effusions and empyema in adults: current practice [J]. Revista Clinica Espanola, 2009,209(10): 485-494.

[1344] Power A, Duncan N, Singh SK, et al. Sodium Citrate Versus Heparin Catheter Locks for Cuffed Central Venous Catheters: A Single-Center Randomized Controlled Trial [J]. American Journal of Kidney Diseases, 2009,53(6): 1034-1041.

[1345] Prinz V, Endres M. The Acute (Cerebro)Vascular Effects of Statins [J]. Anesthesia and Analgesia, 2009,109(2): 572-584.

[1346] Putz SM, Vogiatzi F, Stiewe T, et al. Malignant transformation in a defined genetic background: proteome changes displayed by 2D-PAGE [J]. Molecular Cancer, 2010,9.

[1347] Radwan M, Miller I, Grunert T, et al. The impact of tyrosine kinase 2 (Tyk2) on the proteome of murine macrophages and their response to lipopolysaccharide (LPS) [J]. Proteomics, 2008,8(17): 3469-3485.

[1348] Rahman NM, Maskell N, Davies CWH, et al. PRIMARY RESULT OF THE SECOND MULTICENTRE INTRAPLEURAL SEPSIS (MIST2) TRIAL; RANDOMISED TRIAL OF INTRAPLEURAL TPA AND DNASE IN PLEURAL INFECTION [J]. Thorax, 2009,64: A1-A1.

[1349] Rahman NM, Maskell NA, Davies CWH, et al. Primary result of the 2nd Multi-centre Intrapleural Sepsis (MIST2) trial; randomised trial of intrapleural tPA and DNAse in pleural infection [J]. American Journal of Respiratory and Critical Care Medicine, 2010,181.

[1350] Reddy AJ, Kleeberger SR. Genetic polymorphisms associated with acute lung injury [J]. Pharmacogenomics, 2009,10(9): 1527-1539.

[1351] Reid JM, Dai DW, Gubitz GJ, et al. Gender differences in stroke examined in a 10-year cohort of patients admitted to a Canadian teaching hospital [J]. Stroke, 2008,39(4): 1090-1095.

[1352] Renckens R, Roelofs J, Stegenga ME, et al. Transgenic tissue-type plasminogen activator expression improves host defense during Klebsiella pneumonia [J]. Journal of Thrombosis and Haemostasis, 2008,6(4): 660-668.

[1353] Richardson MA, Gupta A, O'Brien LA, et al. Treatment of sepsis-induced acquired protein C deficiency reverses angiotensin-converting enzyme-2 inhibition and decreases pulmonary inflammatory response [J]. Journal of Pharmacology and Experimental Therapeutics, 2008,325(1): 17-26.

[1354] Richardson PG, Soiffer RJ, Antin JH, et al. Defibrotide for the Treatment of Severe Hepatic Veno-Occlusive Disease and Multiorgan Failure after Stem Cell Transplantation: A Multicenter, Randomized, Dose-Finding Trial [J]. Biology of Blood and Marrow Transplantation, 2010,16(7): 1005-1017.

[1355] Ristic Z, Momcilovic I, Fu JM, et al. Chloroplast protein synthesis elongation factor, EF-Tu, reduces thermal aggregation of rubisco activase [J]. Journal of Plant Physiology, 2007,164(12): 1564-1571.

[1356] Roesslein M, Froehlich C, Miltenberger V, et al. Thiopental protects human SK-N-SH neuroblastoma cells from apoptosis by inducing a heat shock response [J]. Inflammation Research, 2010,59: S12-S12.

[1357] Roncon-Albuquerque R, Beco A, Ferreira AL, et al. Therapeutic implications of heparin-induced thrombocytopenia complicating acute hemodialysis [J]. Clinical Nephrology, 2010,73(4): 326-330.

[1358] Ruelland E, Zachowski A. How plants sense temperature [J]. Environmental and Experimental Botany, 2010,69(3): 225-232.

[1359] Ruiz-Bailen M, Rucabado-Aguilar L, Castillo-Rivera AM, et al. Cardiogenic shock in acute coronary syndrome in the Spanish population [J]. Medical Science Monitor, 2008,14(11): PH46-PH57.

[1360] Ruppert C, Mahavadi P, Wygrecka M, et al. Recombinant production of a hybrid plasminogen activator composed of surfactant protein B and low-molecular-weight urokinase [J]. Thrombosis and Haemostasis, 2008,100(6): 1185-1192.

[1361] Rupprecht HJ. Facilitated PCI: Did the concept fail? [J]. Herz, 2008,33(2): 143-147.

[1362] Saadoun D, Elalamy I, Ghillani-Dalbin P, et al. Cryofibrinogenemia: New Insights into Clinical and Pathogenic Features [J]. American Journal of Medicine, 2009,122(12): 1128-1135.

[1363] Sage E, Mercier O, Van den Eyden F, et al. Endothelial cell apoptosis in chronically obstructed and reperfused pulmonary artery [J]. Respiratory Research, 2008,9.

[1364] Sahn SA. Diagnosis and management of parapneumonic effusions and empyema [J]. Clinical Infectious Diseases, 2007,45(11): 1480-1486.

[1365] Salvucci ME. Association of Rubisco activase with chaperonin-60 beta: a possible mechanism for protecting photosynthesis during heat stress [J]. Journal of Experimental Botany, 2008,59(7): 1923-1933.

[1366] Sanchez O, Planquette B, Wermert D, et al. Massive pulmonary embolism [J]. Presse Medicale, 2008,37(10): 1439-1446.

[1367] Sanderson-Smith ML, Dinkla K, Cole JN, et al. M protein-mediated plasminogen binding is essential for the virulence of an invasive Streptococcus pyogenes isolate [J]. Faseb Journal, 2008,22(8): 2715-2722.

[1368] Sangle G, Zhao R, Mizuno T, et al. INVOLVEMENT OF RAGE, NADPH OXIDASE AND H-RAS/RAF-1 IN GLYCATED LDL-INDUCED EXPRESSION OF HEAT SHOCK FACTOR-1 AND PLASMINOGEN ACTIVATOR INHIBITOR-1 IN VASCULAR ENDOTHELIAL CELLS [J]. Atherosclerosis Supplements, 2009,10(2).

[1369] Sangle GV, Shen GX. Signaling mechanisms for oxidized LDL-induced oxidative stress and the upregulation of plasminogen activator inhibitor-1 in vascular cells [J]. Clinical Lipidology, 2010,5(2): 221-232.

[1370] Sangle GV, Zhao RZ, Mizuno T, et al. RAGE, NADPH Oxidase and Ras/Raf-1 Pathway Mediate Glycated LDL-Induced Expression of Heat Shock Factor-1 and Plasminogen Activator Inhibitor-1 in Vascular Endothelial tells [J]. Diabetes, 2009,58: A91-A91.

[1371] Sangle GV, Zhao RZ, Mizuno TM, et al. Involvement of RAGE, NADPH Oxidase, and Ras/Raf-1 Pathway in Glycated LDL-Induced Expression of Heat Shock Factor-1 and Plasminogen Activator Inhibitor-1 in Vascular Endothelial Cells [J]. Endocrinology, 2010,151(9): 4455-4466.

[1372] Saposnik G, Hill MD, O'Donnell M, et al. Variables associated with 7-day, 30-day, and 1-year fatality after ischemic stroke [J]. Stroke, 2008,39(8): 2318-2324.

[1373] Sapru A, Curley MAQ, Brady S, et al. Elevated PAI-1 is associated with poor clinical outcomes in pediatric patients with acute lung injury [J]. Intensive Care Medicine, 2010,36(1): 157-163.

[1374] Sapru A, Hansen H, Ajayi T, et al. 4G/5G Polymorphism of Plasminogen Activator Inhibitor-1 Gene Is Associated with Mortality in Intensive Care Unit Patients with Severe Pneumonia [J]. Anesthesiology, 2009,110(5): 1086-1091.

[1375] Sari I, Davutoglu V, Bayram N, et al. Fatal giant aortic thrombus presenting with pulmonary edema in a patient with chronic obstructive pulmonary disease [J]. Clinical and Applied Thrombosis-Hemostasis, 2008,14(4): 486-488.

[1376] Savioli M, Cugno M, Polli F, et al. Tight glycemic control may favor fibrinolysis in patients with sepsis [J]. Critical Care Medicine, 2009,37(2): 424-431.

[1377] Scafaro AP, Haynes PA, Atwell BJ. Physiological and molecular changes in Oryza meridionalis Ng., a heat-tolerant species of wild rice [J]. Journal of Experimental Botany, 2010,61(1): 191-202.

[1378] Schochl H, Solomon C, Schulz A, et al. Thromboelastometry (TEM (R)) Findings in Disseminated Intravascular Coagulation in a Pig Model of Endotoxinemia [J]. Molecular Medicine, 2011,17(3-4): 266-272.

[1379] Schouten M, van der Sluijs KF, Gerlitz B, et al. Activated protein C ameliorates coagulopathy but does not influence outcome in lethal H1N1 influenza: a controlled laboratory study [J]. Critical Care, 2010,14(2).

[1380] Schouten M, van't Veer C, Roelofs J, et al. Impact of the factor V Leiden mutation on the outcome of pneumococcal pneumonia: a controlled laboratory study [J]. Critical Care, 2010,14(4).

[1381] Schuetz P, Yano K, Sorasaki M, et al. Influence of diabetes on endothelial cell response during sepsis [J]. Diabetologia, 2011,54(5): 996-1003.

[1382] Schultz MJ, Dixon B, Levi M. The pulmonary protein C system: preventive or therapeutic target in acute lung injury? [J]. Thorax, 2009,64(2): 95-97.

[1383] Sekine H, Mimura J, Oshima M, et al. Hypersensitivity of Aryl Hydrocarbon Receptor-Deficient Mice to Lipopolysaccharide-Induced Septic Shock [J]. Molecular and Cellular Biology, 2009,29(24): 6391-6400.

[1384] Sen P, Komissarov AA, Florova G, et al. Plasminogen activator inhibitor-1 inhibits factor VIIa bound to tissue factor [J]. Journal of Thrombosis and Haemostasis, 2011,9(3): 531-539.

[1385] Senoo T, Hattori N, Tanimoto T, et al. Suppression of plasminogen activator inhibitor-1 by RNA interference attenuates pulmonary fibrosis [J]. Thorax, 2010,65(4): 334-340.

[1386] Seymour LM, Deutscher AT, Jenkins C, et al. A Processed Multidomain Mycoplasma hyopneumoniae Adhesin Binds Fibronectin, Plasminogen, and Swine Respiratory Cilia [J]. Journal of Biological Chemistry, 2010,285(44): 33971-33978.

[1387] Shapiro NI, Aird WC. Sepsis and the broken endothelium [J]. Critical Care, 2011,15(2).

[1388] Shapiro NI, Schuetz P, Yano K, et al. The association of endothelial cell signaling, severity of illness, and organ dysfunction in sepsis [J]. Critical Care, 2010,14(5).

[1389] Shashaty MGS, Kohn NE, Christie JD, et al. Emergency Department Antithrombin III And Plasminogen Activator Inhibitor-1 Initial Plasma Levels Are Associated With Acute Lung Injury In Patients With Severe Sepsis And Septic Shock [J]. American Journal of Respiratory and Critical Care Medicine, 2010,181.

[1390] Shen GX. Oxidative stress and diabetic cardiovascular disorders: roles of mitochondria and NADPH oxidase [J]. Canadian Journal of Physiology and Pharmacology, 2010,88(3): 241-248.

[1391] Shetty S, Bhandary YP, Shetty SK, et al. Induction of Tissue Factor by Urokinase in Lung Epithelial Cells and in the Lungs [J]. American Journal of Respiratory and Critical Care Medicine, 2010,181(12): 1355-1366.

[1392] Shetty S, Padijnayayveetil J, Tucker T, et al. The fibrinolytic system and the regulation of lung epithelial cell proteolysis, signaling, and cellular viability [J]. American Journal of Physiology-Lung Cellular and Molecular Physiology, 2008,295(6): L967-L975.

[1393] Shetty S, Velusamy T, Shetty RS, et al. Post-Transcriptional Regulation of Plasminogen Activator Inhibitor Type-1 Expression in Human Pleural Mesothelial Cells [J]. American Journal of Respiratory Cell and Molecular Biology, 2010,43(3): 358-367.

[1394] Shiehmorteza M, Ahmadi A, Abdollahi M, et al. Recombinant human erythropoietin reduces plasminogen activator inhibitor and ameliorates pro-inflammatory responses following trauma [J]. Daru-Journal of Pharmaceutical Sciences, 2011,19(2): 159-165.

[1395] Shimomura Y, Okumura K, Murayama SY, et al. Complete genome sequencing and analysis of a Lancefield group G Streptococcus dysgalactiae subsp equisimilis strain causing streptococcal toxic shock syndrome (STSS) [J]. Bmc Genomics, 2011,12.

[1396] Shin HS, Xu FY, Bagchi A, et al. Bacterial Lipoprotein TLR2 Agonists Broadly Modulate Endothelial Function and Coagulation Pathways In Vitro and In Vivo [J]. Journal of Immunology, 2011,186(2): 1119-1130.

[1397] Shu H, Fu SZ, Huang XG, et al. Laser-driven shock wave stability in Al through Au and determination of the TPa hugoniot point in Au [J]. Journal of Applied Physics, 2008,103(9).

[1398] Shyu HW, Lin YY, Chen LC, et al. The dengue virus envelope protein induced PAI-1 gene expression via MEK/ERK pathways [J]. Thrombosis and Haemostasis, 2010,104(6): 1219-1227.

[1399] Sienczyk M, Oleksyszyn J. Irreversible Inhibition of Serine Proteases - Design and In Vivo Activity of Diaryl alpha-Aminophosphonate Derivatives [J]. Current Medicinal Chemistry, 2009,16(13): 1673-1687.

[1400] Simon A, Bode U, Lieber K, et al. Review and update of the use of urokinase in the prevention and management of CVAD-related complications in pediatric oncology patients [J]. American Journal of Infection Control, 2008,36(1): 54-58.

[1401] Sinnaeve PR, Ezekowitz JA, Bogaerts K, et al. Reperfusion before percutaneous coronary intervention in ST-elevation myocardial infarction patients is associated with lower N-terminal pro-brain natriuretic peptide levels during follow-up, irrespective of pre-treatment with full-dose fibrinolysis [J]. European Heart Journal, 2009,30(18): 2213-2219.

[1402] Sizlan A, Cekmen N, Bedir S, et al. Comparison of alfentanil and remifentanil infusions in combination with propofol for the outpatient extra-corporeal shock wave lithotripsy [J]. Bratislava Medical Journal-Bratislavske Lekarske Listy, 2011,112(7): 380-384.

[1403] Skagius E, Siegbahn A, Bergqvist D, et al. Fibrinolysis in patients with an abdominal aortic aneurysm with special emphasis on rupture and shock [J]. Journal of Thrombosis and Haemostasis, 2008,6(1): 147-150.

[1404] Smuder AJ, Kavazis AN, Min K, et al. Exercise protects against doxorubicin-induced oxidative stress and proteolysis in skeletal muscle [J]. Journal of Applied Physiology, 2011,110(4): 935-942.

[1405] Smyth AR, Barbato A, Beydon N, et al. Respiratory medicines for children: current evidence, unlicensed use and research priorities [J]. European Respiratory Journal, 2010,35(2): 247-265.

[1406] Sohal M, Foo F, Sirker A, et al. Rescue Angioplasty for Failed Fibrinolysis-Long-Term Follow-Up of a Large Cohort [J]. Catheterization and Cardiovascular Interventions, 2011,77(5): 599-604.

[1407] Song WF, Wei SP, Zhou YJ, et al. Inhibition of Lung Fluid Clearance and Epithelial Na+ Channels by Chlorine, Hypochlorous Acid, and Chloramines [J]. Journal of Biological Chemistry, 2010,285(13): 9716-9728.

[1408] Sonoi H, Matsumoto N, Ogura H, et al. THE EFFECT OF ANTITHROMBIN ON PULMONARY ENDOTHELIAL DAMAGE INDUCED BY CRUSH INJURY [J]. Shock, 2009,32(6): 593-600.

[1409] Sontineni SP, White M, Singh S, et al. Thrombectomy reduces the systemic complications in device-related right atrial septic thrombosis [J]. Canadian Journal of Cardiology, 2009,25(2): E36-E41.

[1410] Soylu H, Brandao LR, Lee KS. Efficacy of Local Instillation of Recombinant Tissue Plasminogen Activator for Restoring Occluded Central Venous Catheters in Neonates [J]. Journal of Pediatrics, 2010,156(2): 197-U152.

[1411] Spinella PC, Holcomb JB. Resuscitation and transfusion principles for traumatic hemorrhagic shock [J]. Blood Reviews, 2009,23(6): 231-240.

[1412] Spohr F, Wenzel V, Bottiger BW. Thrombolysis and other drugs during cardiopulmonary resuscitation [J]. Current Opinion in Critical Care, 2008,14(3): 292-298.

[1413] Stapleton RD, Dixon AE, Parsons PE, et al. The Association Between BMI and Plasma Cytokine Levels in Patients With Acute Lung Injury [J]. Chest, 2010,138(3): 568-577.

[1414] Stastkova Z, Karpiskova R, Koukalova K, et al. Differentiation of Toxigenic Staphylococcus aureus Strains Isolated from Retail Meat Products [J]. Czech Journal of Food Sciences, 2011,29: S17-S22.

[1415] Stein PD, Beemath A, Matta F, et al. Enlarged right ventricle without shock in acute pulmonary embolism: Prognosis [J]. American Journal of Medicine, 2008,121(1): 34-42.

[1416] Stolz A, Pafko P, Harustiak T, et al. Risk factor analysis for early mortality and morbidity following pneumonectomy for non-small cell lung cancer [J]. Bratislava Medical Journal-Bratislavske Lekarske Listy, 2011,112(4): 165-169.

[1417] Stringer KA, Ellingrod VL, Kong FM, et al. Angiotensin Converting Enzyme (ACE) Insertion (I)/Deletion (D) Polymorphism and Tissue Plasminogen Activator (tPA)-7351C>T Enhancer Single Nucleotide Polymorphism (SNP) are Associated with Sepsis-Induced Acute Lung Injury (ALI) [J]. American Journal of Respiratory and Critical Care Medicine, 2010,181.

[1418] Stringer KA, Tobias M, Dunn JS, et al. ACCELERATED DOSING FREQUENCY OF A PULMONARY FORMULATION OF TISSUE PLASMINOGEN ACTIVATOR IS WELL-TOLERATED IN MICE [J]. Clinical and Experimental Pharmacology and Physiology, 2008,35(12): 1454-1460.

[1419] Sun HM, Wang XX, Degen JL, et al. Reduced thrombin generation increases host susceptibility to group A streptococcal infection [J]. Blood, 2009,113(6): 1358-1364.

[1420] Sundaram S, Rathinasabapathi B. Transgenic expression of fern Pteris vittata glutaredoxin PvGrx5 in Arabidopsis thaliana increases plant tolerance to high temperature stress and reduces oxidative damage to proteins [J]. Planta, 2010,231(2): 361-369.

[1421] Swaney JS, Moreno KM, Gentile AM, et al. Sphingosine-1-phosphate (S1P) is a novel fibrotic mediator in the eye [J]. Experimental Eye Research, 2008,87(4): 367-375.

[1422] Takahashi T, Ubukata K, Watanabe H. Invasive infection caused by Streptococcus dysgalactiae subsp equisimilis: characteristics of strains and clinical features [J]. Journal of Infection and Chemotherapy, 2011,17(1): 1-10.

[1423] Tanabe K, Takai S, Matsushima-Nishiwaki R, et al. alpha(2) adrenoreceptor agonist regulates protein kinase C-induced heat shock protein 27 phosphorylation in C6 glioma cells [J]. Journal of Neurochemistry, 2008,106(2): 519-528.

[1424] Tanaka A, Minoguchi K, Chen XY, et al. Activated protein C attenuates leukocyte elastase-induced lung injury in mice [J]. Shock, 2008,30(2): 153-158.

[1425] Tardy B, Venet C, Zeni F, et al. Short term effect of recombinant tissue plasminogen activator in patients with hemodynamically stable acute pulmonary embolism: Results of a meta-analysis involving 464 patients [J]. Thrombosis Research, 2009,124(6): 672-677.

[1426] Tascilar N, Irkorucu O, Tascilar O, et al. Bacterial translocation in experimental stroke: what happens to the gut barrier? [J]. Bratislava Medical Journal-Bratislavske Lekarske Listy, 2010,111(4): 194-199.

[1427] Tasnadi G, Bihari I, Bihari P. Peritoneo-venous shunt implantation as a therapy for chylous ascites [J]. Phlebologie, 2010,39(1): 24-27.

[1428] Thiyagarajan M, Fernandez JA, Lane SM, et al. Activated Protein C Promotes Neovascularization and Neurogenesis in Postischemic Brain via Protease-Activated Receptor 1 [J]. Journal of Neuroscience, 2008,28(48): 12788-12797.

[1429] Thomas RM, Ahmad SA. Management of Acute Post-operative Portal Venous Thrombosis [J]. Journal of Gastrointestinal Surgery, 2010,14(3): 570-577.

[1430] Thorgersen EB, Hellerud BC, Nielsen EW, et al. CD14 inhibition efficiently attenuates early inflammatory and hemostatic responses in Escherichia coli sepsis in pigs [J]. Faseb Journal, 2010,24(3): 712-722.

[1431] Thornburg CD, Smith PB, Smithwick ML, et al. Association between thrombosis and bloodstream infection in neonates with peripherally inserted catheters [J]. Thrombosis Research, 2008,122(6): 782-785.

[1432] Titlic M, Josipovic-Jelic Z. Spondylodiscitis [J]. Bratislava Medical Journal-Bratislavske Lekarske Listy, 2008,109(8): 345-347.

[1433] Todd JL, Tapson VF. Thrombolytic Therapy for Acute Pulmonary Embolism A Critical Appraisal [J]. Chest, 2009,135(5): 1321-1329.

[1434] Treska V, Sutnar A, Mukensnabl P, et al. Liver abscess in human toxocariasis [J]. Bratislava Medical Journal-Bratislavske Lekarske Listy, 2011,112(11): 644-647.

[1435] Trzeciak S, Jones AE, Shapiro NI, et al. A prospective multicenter cohort study of the association between global tissue hypoxia and coagulation abnormalities during early sepsis resuscitation [J]. Critical Care Medicine, 2010,38(4): 1092-1100.

[1436] Tsai TH, Chai HT, Sun CK, et al. Comparison of 30-Day Mortality between Anterior-Wall versus Inferior-Wall ST-Segment Elevation Myocardial Infarction Complicated by Cardiogenic Shock in Patients Undergoing Primary Coronary Angioplasty [J]. Cardiology, 2010,116(2): 144-150.

[1437] Tsangaris I, Tsantes A, Bagos P, et al. The Effect of Plasma Homocysteine Levels on Clinical Outcomes of Patients With Acute Lung Injury/Acute Respiratory Distress Syndrome [J]. American Journal of the Medical Sciences, 2009,338(6): 474-477.

[1438] Tsangaris I, Tsantes A, Bonovas S, et al. The impact of the PAI-1 4G/5G polymorphism on the outcome of patients with ALI/ARDS [J]. Thrombosis Research, 2009,123(6): 832-836.

[1439] Tsantes AE, Nikolopoulos GK, Bagos PG, et al. The effect of the plasminogen activator inhibitor-1 4G/5G polymorphism on the thrombotic risk [J]. Thrombosis Research, 2008,122(6): 736-742.

[1440] Tsantes AE, Tsangaris I, Bonovas S, et al. The effect of four hemostatic gene polymorphisms on the outcome of septic critically ill patients [J]. Blood Coagulation & Fibrinolysis, 2010,21(2): 175-181.

[1441] Tsutsumi S, Beebe K, Neckers L. Impact of heat-shock protein 90 on cancer metastasis [J]. Future Oncology, 2009,5(5): 679-688.

[1442] Tuinman PR, Vlaar AP, Cornet AD, et al. Blood transfusion during cardiac surgery is associated with inflammation and coagulation in the lung: a case control study [J]. Critical Care, 2011,15(1).

[1443] Tukiainen E, Kylanpaa ML, Repo H, et al. Hemostatic Gene Polymorphisms in Severe Acute Pancreatitis [J]. Pancreas, 2009,38(2): E43-E46.

[1444] Ubel PA, Silbergleit R. Behavioral Equipoise: A Way to Resolve Ethical Stalemates in Clinical Research [J]. American Journal of Bioethics, 2011,11(2): 1-8.

[1445] Uyarel H, Ergelen M, Akkaya E, et al. Impact of Day Versus Night as Intervention Time on the Outcomes of Primary Angioplasty for Acute Myocardial Infarction [J]. Catheterization and Cardiovascular Interventions, 2009,74(6): 826-834.

[1446] Valentino LA, Kawji M, Grygotis M. Venous access in the management of hemophilia [J]. Blood Reviews, 2011,25(1): 11-15.

[1447] van den Boogaard FE, Brands X, Schultz MJ, et al. Recombinant human tissue factor pathway inhibitor exerts anticoagulant, anti-inflammatory and antimicrobial effects in murine pneumococcal pneumonia [J]. Journal of Thrombosis and Haemostasis, 2011,9(1): 122-132.

[1448] van der Worp HB, Macleod MR, Kollmar R, et al. Therapeutic hypothermia for acute ischemic stroke: ready to start large randomized trials? [J]. Journal of Cerebral Blood Flow and Metabolism, 2010,30(6): 1079-1093.

[1449] van Zoelen MAD, Florquin S, de Beer R, et al. Urokinase Plasminogen Activator Receptor-Deficient Mice Demonstrate Reduced Hyperoxia-Induced Lung Injury [J]. American Journal of Pathology, 2009,174(6): 2182-2189.

[1450] van Zoelen MAD, Schmidt AM, Florquin S, et al. Receptor for Advanced Glycation End Products Facilitates Host Defense during Escherichia coli-Induced Abdominal Sepsis in Mice [J]. Journal of Infectious Diseases, 2009,200(5): 765-773.

[1451] Varet J, Douglas SK, Gilmartin L, et al. VEGF in the lung: a role for novel isoforms [J]. American Journal of Physiology-Lung Cellular and Molecular Physiology, 2010,298(6): L768-L774.

[1452] Vaschetto R, Kuiper JW, Chiang SR, et al. Inhibition of Poly(Adenosine diphosphate-ribose) polymerase attenuates ventilator-induced lung injury [J]. Anesthesiology, 2008,108(2): 261-268.

[1453] Veress LA, O'Neill HC, Hendry-Hofer TB, et al. Airway Obstruction Due to Bronchial Vascular Injury after Sulfur Mustard Analog Inhalation [J]. American Journal of Respiratory and Critical Care Medicine, 2010,182(11): 1352-1361.

[1454] Vitiello M, Galdiero S, D'Isanto M, et al. Pathophysiological changes of gram-negative bacterial infection can be reproduced by a synthetic peptide mimicking loop L7 sequence of Haemophilus influenzae porin [J]. Microbes and Infection, 2008,10(6): 657-663.

[1455] Wagenaar JFP, Goris MGA, Partiningrum DL, et al. Coagulation disorders in patients with severe leptospirosis are associated with severe bleeding and mortality [J]. Tropical Medicine & International Health, 2010,15(2): 152-159.

[1456] Wang L, Bastarache JA, Ware LB. The coagulation cascade in sepsis [J]. Current Pharmaceutical Design, 2008,14(19): 1860-1869.

[1457] Wang RR, Qiu PX, Jiang WJ, et al. Recombinant fibrinogenase from Agkistrodon acutus venom protects against sepsis via direct degradation of fibrin and TNF-alpha [J]. Biochemical Pharmacology, 2008,76(5): 620-630.

[1458] Wang YM, Thiyagarajan M, Chow N, et al. Differential Neuroprotection and Risk for Bleeding From Activated Protein C With Varying Degrees of Anticoagulant Activity [J]. Stroke, 2009,40(5): 1864-1869.

[1459] Ward PN, Abu-Median A, Leigh JA. Structural consideration of the formation of the activation complex between the staphylokinase-like streptococcal plasminogen activator PadA and bovine plasminogen [J]. Journal of Molecular Biology, 2008,381(3): 734-747.

[1460] Ware LB, Koyama T, Billheimer D, et al. Prognostic and Pathogenetic Value of Combining Clinical and Biochemical Indices in Patients With Acute Lung Injury [J]. Chest, 2010,137(2): 288-296.

[1461] Watkins AM, Cheek DJ, Harvey AE, et al. Heat acclimation and HSP-72 expression in exercising humans [J]. International Journal of Sports Medicine, 2008,29(4): 269-276.

[1462] Watson KE, Dovi WF, Harms BA, et al. Tissue Plasminogen Activator (TPA) Reduces Lung Injury After Acute Blood Loss In Rats [J]. American Journal of Respiratory and Critical Care Medicine, 2011,183.

[1463] Weidle UH, Maisel D, Klostermann S, et al. Intracellular Proteins Displayed on the Surface of Tumor Cells as Targets for Therapeutic Intervention with Antibody-related Agents [J]. Cancer Genomics & Proteomics, 2011,8(2): 49-63.

[1464] Wessmann A, Chandler K, Garosi L. Ischaemic and haemorrhagic stroke in the dog [J]. Veterinary Journal, 2009,180(3): 290-303.

[1465] Wiersinga WJ, Kager LM, Hovius JWR, et al. Urokinase Receptor Is Necessary for Bacterial Defense against Pneumonia-Derived Septic Melioidosis by Facilitating Phagocytosis [J]. Journal of Immunology, 2010,184(6): 3079-3086.

[1466] Wiersinga WJ, Meijers JCM, Levi M, et al. Activation of coagulation with concurrent impairment of anticoagulant mechanisms correlates with a poor outcome in severe melioidosis [J]. Journal of Thrombosis and Haemostasis, 2008,6(1): 32-39.

[1467] Willemse JL, Heylen E, Nesheim ME, et al. Carboxypeptidase U (TAFIa): a new drug target for fibrinolytic therapy? [J]. Journal of Thrombosis and Haemostasis, 2009,7(12): 1962-1971.

[1468] Wingeyer SP, de Larranaga G, Fontana L, et al. Role of 4G/5G promoter polymorphism of Plasminogen Activator Inhibitor-1 (PAI-1) gene in outcome of sepsis [J]. Thrombosis Research, 2010,125(4): 367-369.

[1469] Wright CM, Chovatiya RJ, Jameson NE, et al. Pyrimidinone-peptoid hybrid molecules with distinct effects on molecular chaperone function and cell proliferation [J]. Bioorganic & Medicinal Chemistry, 2008,16(6): 3291-3301.

[1470] Wrightson JM, Davies RJO. The Approach to the Patient with a Parapneumonic Effusion [J]. Seminars in Respiratory and Critical Care Medicine, 2010,31(6): 706-715.

[1471] Wurfel MM. Genetic insights into sepsis: What have we learned and how will it help? [J]. Current Pharmaceutical Design, 2008,14(19): 1900-1911.

[1472] Wygrecka M, Jablonska E, Guenther A, et al. Current view on alveolar coagulation and fibrinolysis in acute inflammatory and chronic interstitial lung diseases [J]. Thrombosis and Haemostasis, 2008,99(3): 494-501.

[1473] Wygrecka M, Marsh LM, Morty RE, et al. Enolase-1 promotes plasminogen-mediated recruitment of monocytes to the acutely inflamed lung [J]. Blood, 2009,113(22): 5588-5598.

[1474] Xie QA, Wondergem R, Shen YH, et al. Benzoquinone ansamycin 17AAG binds to mitochondrial voltage-dependent anion channel and inhibits cell invasion [J]. Proceedings of the National Academy of Sciences of the United States of America, 2011,108(10): 4105-4110.

[1475] Xu CP, Huang BR. Differential proteomic response to heat stress in thermal Agrostis scabra and heat-sensitive Agrostis stolonifera [J]. Physiologia Plantarum, 2010,139(2): 192-204.

[1476] Yamaguchi M, Terao Y, Mori Y, et al. PfbA, a Novel Plasmin- and Fibronectin-binding Protein of Streptococcus pneumoniae, Contributes to Fibronectin-dependent Adhesion and Antiphagocytosis [J]. Journal of Biological Chemistry, 2008,283(52): 36272-36279.

[1477] Yamamoto T, Murai K, Tokita Y, et al. Thrombolysis With a Novel Modified Tissue-Type Plasminogen Activator, Monteplase, Combined With Catheter-Based Treatment for Major Pulmonary Embolism [J]. Circulation Journal, 2009,73(1): 106-110.

[1478] Yang D, Tong L, Wang DAE, et al. Roles of CC chemokine receptors (CCRs) on lipopolysaccharide-induced acute lung injury [J]. Respiratory Physiology & Neurobiology, 2010,170(3): 253-259.

[1479] Yang KY, Liu KT, Chen YC, et al. Plasma soluble vascular endothelial growth factor receptor-1 levels predict outcomes of pneumonia-related septic shock patients: a prospective observational study [J]. Critical Care, 2011,15(1).

[1480] Yang SF, Hsieh YS, Lue KH, et al. Effects of nonsteroidal anti-inflammatory drugs on the expression of urokinase plasminogen activator and inhibitor and gelatinases in the early osteoarthritic knee of humans [J]. Clinical Biochemistry, 2008,41(1-2): 109-116.

[1481] Yang YP, Friggeri A, Banerjee S, et al. Urokinase-Type Plasminogen Activator Inhibits Efferocytosis of Neutrophils [J]. American Journal of Respiratory and Critical Care Medicine, 2010,182(12): 1516-1523.

[1482] Yeh LT, Wu ML, Charles AL, et al. A novel steamed bread making process using salt-stressed baker's yeast [J]. International Journal of Food Science and Technology, 2009,44(12): 2637-2643.

[1483] Yende S, Angus DC, Ding JZ, et al. 4G/5G plasminogen activator inhibitor-1 polymorphisms and haplotypes are associated with pneumonia [J]. American Journal of Respiratory and Critical Care Medicine, 2007,176(11): 1129-1137.

[1484] Yende S, van der Poll T, Lee M, et al. The influence of pre-existing diabetes mellitus on the host immune response and outcome of pneumonia: analysis of two multicentre cohort studies [J]. Thorax, 2010,65(10): 870-877.

[1485] Yoon IS, Au QY, Barber JR, et al. Development of a high-throughput screening assay for cytoprotective agents in rotenone-induced cell death [J]. Analytical Biochemistry, 2010,407(2): 205-210.

[1486] Yousef AA, Jaffe A. The Management of Paediatric Empyema [J]. Hong Kong Journal of Paediatrics, 2009,14(1): 16-21.

[1487] Yun TH, Cott JE, Tapping RI, et al. Proteolytic inactivation of tissue factor pathway inhibitor by bacterial omptins [J]. Blood, 2009,113(5): 1139-1148.

[1488] Yun TH, Morrissey JH. Polyphosphate and omptins: novel bacterial procoagulant agents [J]. Journal of Cellular and Molecular Medicine, 2009,13(10): 4146-4153.

[1489] Zamanian RT, Gould MK. Effectiveness and cost effectiveness of thrombolysis in patients with acute pulmonary embolism [J]. Current Opinion in Pulmonary Medicine, 2008,14(5): 422-426.

[1490] Zhan XH, Kuczynski B, Sharp FR. Post stroke intervention: Is the window widening? [J]. Neuropharmacology, 2011,60(6): 1000-1002.

[1491] Zhang MH, Li GW, Huang W, et al. Proteomic study of Carissa spinarum in response to combined heat and drought stress [J]. Proteomics, 2010,10(17): 3117-3129.

[1492] Zhao R, Moghadasian M, Mizuno T, et al. ROLE OF HEAT SHOCK FACTOR-1 (HSF1) IN UPREGULATION OF PLASMINOGEN ACTIVATOR INHIBITOR-1 IN DIABETIC, APOLIPOPROTEIN E-KNOCKOUT MICE AND FIBROBLASTS FROM HSF1-DEFICIENT MICE [J]. Atherosclerosis Supplements, 2009,10(2).

[1493] Zhao RZ, Ma XL, Shen GX. Transcriptional regulation of plasminogen activator inhibitor-1 in vascular endothelial cells induced by oxidized very low density lipoproteins [J]. Molecular and Cellular Biochemistry, 2008,317(1-2): 197-204.

[1494] Zhao RZ, Ma XL, Xie XP, et al. Involvement of NADPH oxidase in oxidized LDL-induced upregulation of heat shock factor-1 and plasminogen activator inhibitor-1 in vascular endothelial cells [J]. American Journal of Physiology-Endocrinology and Metabolism, 2009,297(1): E104-E111.

[1495] Zhao RZ, Mizuno T, Mogha-Dasain M, et al. Involvement of heat shock factor-1 (HSF1) in upregulation of plasminogen activator inhibitor-1 in streptozotocin-induced diabetic, apolipoprotein E-deficiency mice and fibroblasts from HSF1-knockout mice [J]. Diabetes, 2008,57: A202-A202.

[1496] Zhou SP, Sauve R, Fish T, et al. Salt-induced and Salt-suppressed Proteins in Tomato Leaves [J]. Journal of the American Society for Horticultural Science, 2009,134(2): 289-294.

[1497] Zhou T, Garcia JGN, Zhang W. Integrating microRNAs into a system biology approach to acute lung injury [J]. Translational Research, 2011,157(4): 180-190.

[1498] Zhu YC, Lu F, Dai Y, et al. Synergistic enhancement of immunogenicity and protection in mice against Schistosoma japonicum with codon optimization and electroporation delivery of SjTPI DNA vaccines [J]. Vaccine, 2010,28(32): 5347-5355.

[1499] Zonca P, Cambal M, Jacobi CA. Complicated course after sleeve gastrectomy for obesity [J]. Bratislava Medical Journal-Bratislavske Lekarske Listy, 2010,111(4): 231-234.

[1500] Zuckerman ST, Kao WJ. LC/MS identification of 12 intracellular cytoskeletal and inflammatory proteins from monocytes adherent on surface-adsorbed fibronectin-derived peptides [J]. Journal of Biomedical Materials Research Part A, 2008,85A(2): 513-529.

[1501] Adams RA, Schachtrup C, Davalos D, et al. Fibrinogen signal transduction as a mediator and therapeutic target in inflammation: Lessons from multiple sclerosis [J]. Current Medicinal Chemistry, 2007,14(27): 2925-2936.

[1502] Adrie C, Monchi M, Laurent I, et al. Coagulopathy after successful cardiopulmonary resuscitation following cardiac arrest - Implication of the protein C anticoagulant pathway [J]. Journal of the American College of Cardiology, 2005,46(1): 21-28.

[1503] Ahmed SI, Gripaldo RE, Alao OA. Empyema necessitans in the setting of pneumonia and parapneumonic effusion [J]. American Journal of the Medical Sciences, 2007,333(2): 106-108.

[1504] Aird WC. Phenotypic heterogeneity of the endothelium I. Structure, function, and mechanisms [J]. Circulation Research, 2007,100(2): 158-173.

[1505] Albert J, Harbut P, Zielinski S, et al. Prolonged exposure to inhaled nitric oxide does not affect haemostasis in piglets [J]. Intensive Care Medicine, 2007,33(9): 1594-1601.

[1506] Alexander KP, Newby LK, Armstrong PW, et al. Acute coronary care in the elderly, Part II - ST-segment-elevation myocardial infarction - A scientific statement for healthcare professionals from the American Heart Association council on clinical cardiology - In collaboration with the Society of Geriatric Cardiology [J]. Circulation, 2007,115(19): 2570-2589.

[1507] Alidoosti M, Salarifar M, Hajizeinali A, et al. Outcomes of primary percutaneous coronary intervention in acute myocardial infarction at Tehran Heart Center [J]. Medical Principles and Practice, 2007,16(5): 333-338.

[1508] Allen GB, Leclair T, Cloutier M, et al. The response to recruitment worsens with progression of lung injury and fibrin accumulation in a mouse model of acid aspiration [J]. American Journal of Physiology-Lung Cellular and Molecular Physiology, 2007,292(6): L1580-L1589.

[1509] Alomari AI, Falk A. The natural history of tunneled hemodialysis catheters removed or exchanged: A single-institution experience [J]. Journal of Vascular and Interventional Radiology, 2007,18(2): 227-235.

[1510] Alsfasser G, Warshaw AL, Thayer SP, et al. Decreased inflammation and improved survival with recombinant human activated protein C treatment in experimental acute pancreatitis [J]. Archives of Surgery, 2006,141(7): 670-676.

[1511] Altinbas A, Kucuktepe Z, Ozaydin M, et al. A rare cause of myocardial infarction: acute inferoposterior myocardial infarction after successful intravenous thrombolytic treatment of mechanical mitral prosthetic valve thrombosis [J]. International Journal of Cardiovascular Imaging, 2005,21(5): 509-512.

[1512] Anderson PD, Mitchell PM, Rathlev NK, et al. Potential diversion rates associated with prehospital acute myocardial infarction triage strategies [J]. Journal of Emergency Medicine, 2004,27(4): 345-353.

[1513] Angelici E, Spezzano M, Vaccari S, et al. Urokinase plasminogen activator and TGF-beta production in immunosuppressed patients with and without P-Jiroveci infection [J]. Microbial Pathogenesis, 2006,41(1): 1-9.

[1514] Aoyagi T, Shimba S, Tezuka M. Characteristics of circadian gene expressions in mice white adipose tissue and 3T3-L1 adipocytes [J]. Journal of Health Science, 2005,51(1): 21-32.

[1515] Armstrong PW, Comm WS. A comparison of pharmacologic therapy with/without timely coronary intervention vs. primary percutaneous intervention early after ST-elevation myocardial infarction: the WEST (Which Early ST-elevation myocardial infarction Therapy) study [J]. European Heart Journal, 2006,27(13): 1530-1538.

[1516] Arndt PG, Young SK, Poch KR, et al. Systemic inhibition of the angiotensin-converting enzyme limits lipopolysaccharide-induced lung neutrophil recruitment through both bradykinin and angiotensin II-regulated pathways [J]. Journal of Immunology, 2006,177(10): 7233-7241.

[1517] Arndt PG, Young SK, Worthen GS. Regulation of lipopolysaccharide-induced lung inflammation by plasminogen activator inhibitor-1 through a JNK-mediated pathway [J]. Journal of Immunology, 2005,175(6): 4049-4059.

[1518] Asakura H, Wada H, Okamoto K, et al. Evaluation of haemostatic molecular markers for diagnosis of disseminated intravascular coagulation in patients with infections [J]. Thrombosis and Haemostasis, 2006,95(2): 282-287.

[1519] Avrorin EN, Simonenko VA, Shibarshov LI. Physics research during nuclear explosions [J]. Physics-Uspekhi, 2006,49(4): 432-437.

[1520] Aydin MD, Gundogdu C, Akcay F, et al. Protective effects of cisternal irrigation on leptomeningeal and cortical structures in meningitis: An experimental study [J]. Neurology India, 2005,53(1): 90-92.

[1521] Bae J, Ahn S, Yim H, et al. Prevention of intraperitoneal adhesions and abscesses by polysaccharides isolated from Phellinus spp in a rat peritonitis model [J]. Annals of Surgery, 2005,241(3): 534-540.

[1522] Bae JS, Jin HK, Jang KH. The effect of polysaccharides and carboxymethylcellulose combination to prevent intraperitoneal adhesion and abscess formation in a rat peritonitis model [J]. Journal of Veterinary Medical Science, 2004,66(10): 1205-1211.

[1523] Bai H, Ma D, Zhang YG, et al. Molecular design and characterization of recombinant long half-life mutants of human tissue factor pathway inhibitor [J]. Thrombosis and Haemostasis, 2005,93(6): 1055-1060.

[1524] Bastarache JA, Ware LB, Bernard GR. The role of the coagulation cascade in the continuum of sepsis and acute lung injury and acute respiratory distress syndrome [J]. Seminars in Respiratory and Critical Care Medicine, 2006,27(4): 365-376.

[1525] Bergheim I, Luyendyk JP, Steele C, et al. Metformin prevents endotoxin-induced liver injury after partial hepatectomy [J]. Journal of Pharmacology and Experimental Therapeutics, 2006,316(3): 1053-1061.

[1526] Beutel K, Simon A. Diagnostic and management of central venous line infections in pediatric cancer patients [J]. Klinische Padiatrie, 2005,217: S91-S100.

[1527] Binder A, Endler G, Muller M, et al. 4G4G genotype of the plasminogen activator inhibitor-1 promoter polymorphism associates with disseminated intravascular coagulation in children with systemic meningococcemia [J]. Journal of Thrombosis and Haemostasis, 2007,5(10): 2049-2054.

[1528] Binette TM, Taylor FB, Peer G, et al. Thrombin-thrombomodulin connects coagulation and fibrinolysis: more than an in vitro phenomenon [J]. Blood, 2007,110(9): 3168-3175.

[1529] Blanc P, Dubus JC, Bosdure E, et al. Community-acquired parapneumonic effusion in children: what's new? [J]. Archives De Pediatrie, 2007,14(1): 64-72.

[1530] Blaney M, Shen V, Kerner JA, et al. Alteplase for the treatment of central venous catheter occlusion in children: Results of a prospective, open-label, single-arm study (The Cathflo Activase Pediatric Study) [J]. Journal of Vascular and Interventional Radiology, 2006,17(11): 1745-1751.

[1531] Bokarewa MI, Jin T, Tarkowski A. Staphylococcus aureus: Staphylokinase [J]. International Journal of Biochemistry & Cell Biology, 2006,38(4): 504-509.

[1532] Borden WB, Faxon DP. Facilitated percutaneous coronary intervention [J]. Journal of the American College of Cardiology, 2006,48(6): 1120-1128.

[1533] Borodziuk S, Kasperczuk A, Pisarczyk T, et al. Study of the conditions for the effective energy transfer in a process of acceleration and collision of the thin metal disks with the massive target [J]. European Physical Journal D, 2007,41(2): 311-317.

[1534] Bouki KP, Pavlakis G, Papasteriadis E. Management of cardiogenic shock due to acute coronary syndromes [J]. Angiology, 2005,56(2): 123-130.

[1535] Bouman CSC, De Pont A, Meijers JCM, et al. The effects of continuous venovenous hemofiltration on coagulation activation [J]. Critical Care, 2006,10(5).

[1536] Bouros D, Tzouvelekis A, Antoniou KM, et al. Intrapleural fibrinolytic therapy for pleural infection [J]. Pulmonary Pharmacology & Therapeutics, 2007,20(6): 616-626.

[1537] Boursier F, Maistre JP, Saddedine M, et al. Prehospital thrombolysis of a pulmonary embolism with a severe shock [J]. Annales Francaises D Anesthesie Et De Reanimation, 2004,23(12): 1182-1184.

[1538] Braff MH, Jones AL, Skerrett SJ, et al. Staphylococcus aureus exploits cathelicidin antimicrobial peptides produced during early pneumonia to promote staphylokinase-dependent fibrinolysis [J]. Journal of Infectious Diseases, 2007,195(9): 1365-1372.

[1539] Briones ML, Blanquer J, Ferrando D, et al. Assessment of analysis of urinary pneumococcal antigen by immunochromatography for etiologic diagnosis of community-acquired pneumonia in adults [J]. Clinical and Vaccine Immunology, 2006,13(10): 1092-1097.

[1540] Brohi K, Cohen MJ, Ganter MT, et al. Acute traumatic coagulopathy: Initiated by hypoperfusion - Modulated through the protein C pathway? [J]. Annals of Surgery, 2007,245(5): 812-818.

[1541] Bucki R, Pastore JJ. Bacterial endotoxin as inhibitor of the enzymatic activity of human thrombin [J]. European Journal of Haematology, 2006,76(6): 510-515.

[1542] Budinger GRS, Sznajder JI. The alveolar-epithelial barrier: A target for potential therapy [J]. Clinics in Chest Medicine, 2006,27(4): 655-+.

[1543] Buyne OR, Bleichrodt RP, van Goor H, et al. Tissue-type plasminogen activator prevents formation of intra-abdominal abscesses after surgical treatment of secondary peritonitis in a rat model [J]. International Journal of Colorectal Disease, 2007,22(7): 819-825.

[1544] Caceres-Loriga FM, Perez-Lopez H, Santos-Gracia J, et al. Prosthetic heart valve thrombosis: Pathogenesis, diagnosis and management [J]. International Journal of Cardiology, 2006,110(1): 1-6.

[1545] Caner I, Olgun H, Buyukavci M, et al. A giant thrombus in the right ventricle of a newborn with Down syndrome: Successful treatment with rt-PA [J]. Journal of Pediatric Hematology Oncology, 2006,28(3): 120-122.

[1546] Cantor WJ, Brunet F, Ziegler CP, et al. Immediate angioplasty after thrombolysis: a systematic review [J]. Canadian Medical Association Journal, 2005,173(12): 1473-1481.

[1547] Cantor WJ, Burstein J, Choi R, et al. Transfer for urgent percutaneous coronary intervention early after thrombolysis for ST-elevation myocardial infarction: The TRANSFER-AMI pilot feasibility study [J]. Canadian Journal of Cardiology, 2006,22(13): 1121-1126.

[1548] Carmassi F, De Negri F, Fioriti R, et al. Insulin resistance causes impaired vasodilation and hypofibrinolysis in young women with polycystic ovary syndrome [J]. Thrombosis Research, 2005,116(3): 207-214.

[1549] Cederqvist K, Siren V, Petaja J, et al. High concentrations of plasminogen activator inhibitor-1 in lungs of preterm infants with respiratory distress syndrome [J]. Pediatrics, 2006,117(4): 1226-1234.

[1550] Celliers PM, Collins GW, Hicks DG, et al. Systematic uncertainties in shock-wave impedance-match analysis and the high-pressure equation of state of Al [J]. Journal of Applied Physics, 2005,98(11).

[1551] Cequier A, Bueno H, Auge JM, et al. Characteristics and mortality following primary percutaneous coronary intervention for acute myocardial infarction in Spain. Results from the TRIANA 1 (TRatamiento del Infarto Agudo de miocardio eN Ancianos) registry [J]. Revista Espanola De Cardiologia, 2005,58(4): 341-350.

[1552] Chang SL, Beltran JA, Swarup S. Expression of the Mu opioid receptor in the human immunodeficiency virus type 1 transgenic rat model [J]. Journal of Virology, 2007,81(16): 8406-8411.

[1553] Chang WH, Cemal CK, Hsu YH, et al. Dynamic expression of Hsp27 in the presence of mutant ataxin-3 [J]. Biochemical and Biophysical Research Communications, 2005,336(1): 258-267.

[1554] Chen CH, Hsu WH, Chen HJ, et al. Different bacteriology and prognosis of thoracic empyemas between patients with chronic and end-stage renal disease [J]. Chest, 2007,132(2): 532-539.

[1555] Chen TJ, Jeng JY, Lin CW, et al. Quercetin inhibition of ROS-dependent and -independent apoptosis in rat glioma C6 cells [J]. Toxicology, 2006,223(1-2): 113-126.

[1556] Cheng G, Vintch JRE. A retrospective analysis of the management of parapneumonic empyemas in a county teaching facility from 1992 to 2004 [J]. Chest, 2005,128(5): 3284-3290.

[1557] Chittari M, Ahmad I, Chambers B, et al. Retrospective observational case-control study comparing prehospital thrombolytic therapy for ST-elevation myocardial infarction with in-hospital thrombolytic therapy for patients from same area [J]. Emergency Medicine Journal, 2005,22(8): 582-585.

[1558] Choi G, Hofstra JJH, Roelofs J, et al. Recombinant human activated protein C inhibits local and systemic activation of coagulation without influencing inflammation during Pseudomonas aeruginosa pneumonia in rats [J]. Critical Care Medicine, 2007,35(5): 1362-1368.

[1559] Choi G, Schultz MJ, van Till JWO, et al. Disturbed alveolar fibrin turnover during pneumonia is restricted to the site of infection [J]. European Respiratory Journal, 2004,24(5): 786-789.

[1560] Choi G, Vlaar APJ, Schouten M, et al. Natural anticoagulants limit lipopolysaccharide-induced pulmonary coagulation but not inflammation [J]. European Respiratory Journal, 2007,30(3): 423-428.

[1561] Chowdary TK, Raman B, Ramakrishna T, et al. Interaction of mammalian Hsp22 with lipid membranes [J]. Biochemical Journal, 2007,401: 437-445.

[1562] Christie JD, Robinson N, Ware LB, et al. Association of protein C and type 1 plasminogen activator inhibitor with primary graft dysfunction [J]. American Journal of Respiratory and Critical Care Medicine, 2007,175(1): 69-74.

[1563] Chromy BA, Choi MW, Murphy GA, et al. Proteomic characterization of Yersinia pestis virulence [J]. Journal of Bacteriology, 2005,187(23): 8172-8180.

[1564] Chua F, Sly PD, Laurent GJ. Pediatric lung disease: From proteinases to pulmonary fibrosis [J]. Pediatric Pulmonology, 2005,39(5): 392-401.

[1565] Chung CL, Chen CH, Sheu JR, et al. Proinflammatory cytokines, transforming growth factor-beta 1, and fibrinolytic enzymes in loculated and free-flowing pleural exudates [J]. Chest, 2005,128(2): 690-697.

[1566] Chunn JL, Molina JG, Mi TJ, et al. Adenosine-dependent pulmonary fibrosis in adenosine deaminase-deficient mice [J]. Journal of Immunology, 2005,175(3): 1937-1946.

[1567] Cinar A, Dilber E, Karagoz T, et al. Association of prothrombin gene mutation with sepsis in a preterm with multiple intracardiac thrombi [J]. Echocardiography-a Journal of Cardiovascular Ultrasound and Allied Techniques, 2005,22(4): 340-344.

[1568] Colman RW. Regulation of angiogenesis by the kallikrein-kinin system [J]. Current Pharmaceutical Design, 2006,12(21): 2599-2607.

[1569] Congote LF. Serpin A1 and CD91 as host instruments against HIV-1 infection: Are extracellular antiviral peptides acting as intracellular messengers? [J]. Virus Research, 2007,125(2): 119-134.

[1570] Constantin JM, Mira JP, Guerin R, et al. Lemierre's syndrome and genetic polymorphisms: a case report [J]. Bmc Infectious Diseases, 2006,6.

[1571] Costa SM, Freire MS, Alves AMB. DNA vaccine against the non-structural 1 protein (NS1) of dengue 2 virus [J]. Vaccine, 2006,24(21): 4562-4564.

[1572] Cremonesini D, Thomson AH. How should we manage empyema: Antibiotics alone, fibrinolytics, or primary video-assisted thoracoscopic surgery (VATS)? [J]. Seminars in Respiratory and Critical Care Medicine, 2007,28(3): 322-332.

[1573] Cucherat M, Bonnefoy E, Tremeau G. Primary angioplasty versus intravenous thrombolysis for acute myocardial infarction (Withdrawn paper. 2003, art. no. CD001560.pub2) [J]. Cochrane Database of Systematic Reviews, 2005, (1).

[1574] Dahlem P, Bos AP, Haitsma JJ, et al. Alveolar fibrinolytic capacity suppressed by injurious mechanical ventilation [J]. Intensive Care Medicine, 2005,31(5): 724-732.

[1575] Dahlem P, Bos AP, Haitsma JJ, et al. Mechanical ventilation affects alveolar fibrinolysis in LPS-induced lung injury [J]. European Respiratory Journal, 2006,28(5): 992-998.

[1576] Dahm JB, Ebersole D, Das T, et al. Prevention of distal embolization and no-reflow in patients with acute myocardial infarction and total occlusion in the infarct-related vessel: A subgroup analysis of the cohort of acute revascularization in myocardial infarction with excimer laser - CARMEL multicenter study [J]. Catheterization and Cardiovascular Interventions, 2005,64(1): 67-74.

[1577] Dangelser G, Gottwalles Y, Huk M, et al. Acute ST-elevation myocardial infarction in the elderly (> 75 years) - Results from a regional multicenter study [J]. Presse Medicale, 2005,34(14): 983-989.

[1578] Dauer DJ, Ferraro B, Song LX, et al. Stat3 regulates genes common to both wound healing and cancer [J]. Oncogene, 2005,24(21): 3397-3408.

[1579] Davies MR, McMillan DJ, Van Domselaar GH, et al. Phage 3396 from a Streptococcus dysgalactiae subsp equisimilis pathovar may have its origins in Streptococcus pyogenes [J]. Journal of Bacteriology, 2007,189(7): 2646-2652.

[1580] de Kruif MD, Lemaire LC, Giebelen IA, et al. Prednisolone dose-dependently influences inflammation and coagulation during human endotoxemia [J]. Journal of Immunology, 2007,178(3): 1845-1851.

[1581] De Luca G, Suryapranata H, Grimaldi R, et al. Coronary stenting and abciximab in primary angioplasty for ST-segment-elevation myocardial infarction [J]. Qjm-an International Journal of Medicine, 2005,98(9): 633-641.

[1582] Demirevska-Kepova K, Holzer R, Simova-Stoilova L, et al. Heat stress effects on ribulose-1,5-bisphosphate carboxylase/oxygenase, Rubisco binding protein and Rubisco activase in wheat leaves [J]. Biologia Plantarum, 2005,49(4): 521-525.

[1583] Deora AB, Kreitzer G, Jacovina AT, et al. An annexin 2 phosphorylation switch mediates p11-dependent translocation of annexin 2 to the cell surface [J]. Journal of Biological Chemistry, 2004,279(42): 43411-43418.

[1584] Determann RM, Millo JL, Garrard CS, et al. Bronchoalveolar levels of plasminogen activator inhibitor-1 and soluble tissue factor are sensitive and specific markers of pulmonary inflammation [J]. Intensive Care Medicine, 2006,32(6): 946-947.

[1585] Diehl JL, Borgel D. Sepsis and coagulation [J]. Current Opinion in Critical Care, 2005,11(5): 454-460.

[1586] Dieker HJ, van Horssen EV, Hersbach F, et al. Transport for abciximab facilitated primary angioplasty versus on-site thrombolysis with a liberal rescue policy: the randomised Holland Infarction Study (HIS) [J]. Journal of Thrombosis and Thrombolysis, 2006,22(1): 39-45.

[1587] Dixon B. The role of microvascular thrombosis in sepsis [J]. Anaesthesia and Intensive Care, 2004,32(5): 619-629.

[1588] Dixon B, Santamaria J, Campbell D. Coagulation activation and organ dysfunction following cardiac surgery [J]. Chest, 2005,128(1): 229-236.

[1589] Douma RA, Kamphuisen PW. Thrombolysis for pulmonary embolism and venous thrombosis: Is it worthwhile? [J]. Seminars in Thrombosis and Hemostasis, 2007,33(8): 821-828.

[1590] Drake RP. Hydrodynamic instabilities in astrophysics and in laboratory high-energy-density systems [J]. Plasma Physics and Controlled Fusion, 2005,47: B419-B440.

[1591] Dunn JS, Nayar R, Campos J, et al. Feasibility of tissue plasminogen activator formulated for pulmonary delivery [J]. Pharmaceutical Research, 2005,22(10): 1700-1707.

[1592] Edge SE, Morgan MB, Gleason DF, et al. Development of a coral cDNA array to examine gene expression profiles in Montastraea faveolata exposed to environmental stress [J]. Marine Pollution Bulletin, 2005,51(5-7): 507-523.

[1593] El Solh AA, Alhajjhasan A, Ramadan FH, et al. A comparative study of community- and nursing home-acquired empyema thoracis [J]. Journal of the American Geriatrics Society, 2007,55(11): 1847-1852.

[1594] El Solh AA, Bhora M, Pineda L, et al. Alveolar plasminogen activator inhibitor-1 predicts ARDS in aspiration pneumonitis [J]. Intensive Care Medicine, 2006,32(1): 110-115.

[1595] El Solh AA, Choi G, Schultz MJ, et al. Clinical and hemostatic responses to treatment in ventilator-associated pneumonia: Role of bacterial pathogens [J]. Critical Care Medicine, 2007,35(2): 490-496.

[1596] El-Banayosy A, Cobaugh D, Zittermann A, et al. A multidisciplinary network to save the lives of severe, persistent cardiogenic shock patients [J]. Annals of Thoracic Surgery, 2005,80(2): 543-547.

[1597] Elg M, Gustaftson D. A combination of a thrombin inhibitor and dexamethasone prevents the development of experimental disseminated intravascular coagulation in rats [J]. Thrombosis Research, 2006,117(4): 429-437.

[1598] El-Solh AA, Okada M, Pietrantoni C, et al. Procoagulant and fibrinolytic activity in ventilator-associated pneumonia: impact of inadequate antimicrobial therapy [J]. Intensive Care Medicine, 2004,30(10): 1914-1920.

[1599] Epaud R, Aubertin G, Larroquet M, et al. Conservative use of chest-tube insertion in children with pleural effusion [J]. Pediatric Surgery International, 2006,22(4): 357-362.

[1600] Escobar MA, Hoelz DJ, Sandoval JA, et al. Profiling of nuclear extract proteins from human neuroblastoma cell lines: the search for fingerprints [J]. Journal of Pediatric Surgery, 2005,40(2): 349-358.

[1601] Fan GH, Qi C, Chen SD. Heat shock proteins reduce toxicity of 1-methyl-4-phenylpyridinium ion in SK-N-SH cells [J]. Journal of Neuroscience Research, 2005,82(4): 551-562.

[1602] Fan GH, Zhou HY, Yang H, et al. Heat shock proteins reduce alpha-synuclein aggregation induced by MPP+ in SK-N-SH cells [J]. Febs Letters, 2006,580(13): 3091-3098.

[1603] Fasano R, Kent P, Valentino L. Superior vena cava thrombus treated with low-dose, peripherally administered recombinant tissue plasminogen activator in a child - Case report and review of the literature [J]. Journal of Pediatric Hematology Oncology, 2005,27(12): 692-695.

[1604] Fernandez-Monreal M, Lopez-Atalaya JP, Benchenane K, et al. Arginine 260 of the amino-terminal domain of NR1 subunit is critical for tissue-type plasminogen activator-mediated enhancement of N-methyl-D-aspartate receptor signaling [J]. Journal of Biological Chemistry, 2004,279(49): 50850-50856.

[1605] Fingleton B. Matrix metalloproteinases as valid clinical targets [J]. Current Pharmaceutical Design, 2007,13(3): 333-346.

[1606] Firdaus WJJ, Wyttenbach A, Giuliano P, et al. Huntingtin inclusion bodies are iron-dependent centers of oxidative events [J]. Febs Journal, 2006,273(23): 5428-5441.

[1607] Fouassier M, Moreau D, Thiolliere F, et al. Evolution of thrombin formation and fibrinolysis markers, including thrombin activatable fibrinolysis inhibitor, during severe meningococcemia [J]. Pathophysiology of Haemostasis and Thrombosis, 2005,34(6): 284-287.

[1608] Franchini M, Veneri D, Lippi G. Inflammation and hemostasis: A bidirectional interaction [J]. Clinical Laboratory, 2007,53(1-2): 63-67.

[1609] Frenkel V, Oberoi J, Stone MJ, et al. Pulsed high-intensity focused ultrasound enhances thrombolysis in an in vitro model [J]. Radiology, 2006,239(1): 86-93.

[1610] Friedrich EB, Kindermann M, Link A, et al. Splenic rupture complicating periinterventional glycoprotein IIb/IIIa antagonist therapy for in polycythemia vera myocardial infarction [J]. Zeitschrift Fur Kardiologie, 2005,94(3): 200-204.

[1611] Frye SR, Yee A, Eskin SG, et al. CDNA microarray analysis of endothelial cells subjected to cyclic mechanical strain: importance of motion control [J]. Physiological Genomics, 2005,21(1): 124-130.

[1612] Fu SZ, Huang XG, Ma MX, et al. Analysis of measurement error in the experiment of laser equation of state with impedance-match way and the Hugoniot data of Cu up to similar to 2.24 TPa with high precision [J]. Journal of Applied Physics, 2007,101(4).

[1613] Fujino M, Yamakami K, Oda T, et al. Sequence and expression of NAPIr is conserved among group A streptococci isolated from patients with acute poststreptococcal glomerulonephritis (APSGN) and non-APSGN [J]. Journal of Nephrology, 2007,20(3): 364-369.

[1614] Fuller MK, Helmrath MA. Thoracic empyema, application of video-assisted thoracic surgery and its current management [J]. Current Opinion in Pediatrics, 2007,19(3): 328-332.

[1615] Furuyama A, Hirano S, Koike E, et al. Induction of oxidative stress and inhibition of plasminogen activator inhibitor-1 production in endothelial cells following exposure to organic extracts of diesel exhaust particles and urban fine particles [J]. Archives of Toxicology, 2006,80(3): 154-162.

[1616] Gaedeke J, Noble NA, Border WA. Curcumin blocks fibrosis in anti-Thy 1 glomerulonephritis through up-regulation of heme oxygenase 1 [J]. Kidney International, 2005,68(5): 2042-2049.

[1617] Gando S, Hayakawa M, Sawamura A, et al. The activation of neutrophil elastase-mediated fibrinolysis is not: Sufficient to overcome the fibrinolytic shutdown of disseminated intravascular coagulation associated with systemic inflammation [J]. Thrombosis Research, 2007,121(1): 67-73.

[1618] Gando S, Sawamura A, Hayakawa M, et al. High macrophage migration inhibitory factor levels in disseminated intravascular coagulation patients with systemic inflammation [J]. Inflammation, 2007,30(3-4): 118-124.

[1619] Garbarino-Pico E, Niu S, Rollag MD, et al. Immediate early response of the circadian polyA ribonuclease nocturnin to two extracellular stimuli [J]. Rna-a Publication of the Rna Society, 2007,13(5): 745-755.

[1620] Garcia-Segarra G, Espinosa G, Tassies D, et al. Increased mortality in septic shock with the 4G/4G genotype of plasminogen activator inhibitor 1 in patients of white descent [J]. Intensive Care Medicine, 2007,33(8): 1354-1362.

[1621] Geden SE, Gardner RA, Fabbrini MS, et al. Lipopolyamine treatment increases the efficacy of intoxication with saporin and an anticancer saporin conjugate [J]. Febs Journal, 2007,274(18): 4825-4836.

[1622] Geishofer G, Binder A, Muller M, et al. 4G/5G promoter polymorphism in the plasminogen-activator-inhibitor-1 gene in children with systemic meningococcaemia [J]. European Journal of Pediatrics, 2005,164(8): 486-490.

[1623] Gheribi AE, Roussel JM, Rogez J. Phenomenological Hugoniot curves for transition metals up to 1 TPa [J]. Journal of Physics-Condensed Matter, 2007,19(47).

[1624] Gibson CM, Zorkun C, Molhoek P, et al. Dose escalation trial of the efficacy, safety, and pharmacokinetics of a novel fibrinolytic agent, BB-10153, in patients with ST elevation MI: Results of the TIMI 31 trial [J]. Journal of Thrombosis and Thrombolysis, 2006,22(1): 13-21.

[1625] Gilmour PS, Morrison ER, Vickers MA, et al. The procoagulant potential of environmental particles (PM10) [J]. Occupational and Environmental Medicine, 2005,62(3): 164-171.

[1626] Gilmour PS, Nyska A, Schladweiler MC, et al. Cardiovascular and blood coagulative effects of pulmonary zinc exposure [J]. Toxicology and Applied Pharmacology, 2006,211(1): 41-52.

[1627] Ginsberg MD, Hill MD, Palesch YY, et al. The ALIAS Pilot Trial - A dose-escalation and safety study of albumin therapy for acute ischemic stroke-I: Physiological responses and safety results [J]. Stroke, 2006,37(8): 2100-2106.

[1628] Gomes I, Mathur SK, Espenshade BM, et al. Eosinophil-fibroblast interactions induce fibroblast IL-6 secretion and extracellular matrix gene expression: Implications in fibrogenesis [J]. Journal of Allergy and Clinical Immunology, 2005,116(4): 796-804.

[1629] Griffin JH, Fernandez JA, Mosnier LO, et al. The promise of protein C [J]. Blood Cells Molecules and Diseases, 2006,36(2): 211-216.

[1630] Gupta PJ. Infra red photocoagulation of early grades of hemorrhoids 5-year follow-up study [J]. Bratislava Medical Journal-Bratislavske Lekarske Listy, 2007,108(4-5): 223-226.

[1631] Gurisik E, Warton K, Martin DK, et al. An in vitro study of the effects of exposure to a GSM signal in two human cell lines: Monocytic U937 and neuroblastoma SK-N-SH [J]. Cell Biology International, 2006,30(10): 793-799.

[1632] Gurm HS, Bates ER. Cardiogenic shock complicating myocardial infarction [J]. Critical Care Clinics, 2007,23(4): 759-+.

[1633] Guyot N, Zani ML, Berger P, et al. Proteolytic susceptibility of the serine protease inhibitor trappin-2 (pre-elafin): evidence for tryptase-mediated generation of elafin [J]. Biological Chemistry, 2005,386(4): 391-399.

[1634] Ha HY, Kim Y, Ryoo ZY, et al. Inhibition of the TPA-induced cutaneous inflammation and hyperplasia by EC-SOD [J]. Biochemical and Biophysical Research Communications, 2006,348(2): 450-458.

[1635] Hacimustafaoglu M, Celebi S, Sarimehmet H, et al. The evaluation and cluster analysis of parapneumonic effusion in childhood [J]. Journal of Tropical Pediatrics, 2006,52(1): 52-55.

[1636] Hadi HAR, Al Suwaidi J, Bener A, et al. Thrombolytic therapy use for acute myocardial infarction and outcome in Qatar [J]. International Journal of Cardiology, 2005,102(2): 249-254.

[1637] Hagiwara S, Iwasaka H, Noguchi T. Nafamostat mesilate inhibits the expression of HMGB1 in lipopolysaccharide-induced acute lung injury (Retracted article. See vol. 29, pg. 484, 2015) [J]. Journal of Anesthesia, 2007,21(2): 164-170.

[1638] Hampton T. Pneumonic plague [J]. Jama-Journal of the American Medical Association, 2007,297(9): 941-941.

[1639] Hardaway RM. A brief overview of acute respiratory distress syndrome [J]. World Journal of Surgery, 2006,30(10): 1829-1835.

[1640] Harris T, Meek S. When should we thrombolyse patients with pulmonary embolism? A systematic review of the literature [J]. Emergency Medicine Journal, 2005,22(11): 766-771.

[1641] Hawreliak J, Colvin J, Eggert J, et al. Modeling planetary interiors in laser based experiments using shockless compression [J]. Astrophysics and Space Science, 2007,307(1-3): 285-289.

[1642] Hermans PWM, Hazelzet JA. Plasminogen activator inhibitor type 1 gene polymorphism and sepsis [J]. Clinical Infectious Diseases, 2005,41: S453-S458.

[1643] Herrmann HC. Update and rationale for ongoing acute myocardial infarction trials: Combination therapy, facilitation, and myocardial preservation [J]. American Heart Journal, 2006,151(6): S30-S39.

[1644] Hirade K, Tanabe K, Niwa M, et al. Adenylyl cyclase-cAMP system inhibits thrombin-induced HSP27 in vascular smooth muscle cells [J]. Journal of Cellular Biochemistry, 2005,94(3): 573-584.

[1645] Hofstra JJ, Schouten M, Levi M. Thrombophilia and outcome in severe infection and sepsis [J]. Seminars in Thrombosis and Hemostasis, 2007,33(6): 604-609.

[1646] Hollingshead M, Burke CT, Mauro MA, et al. Transcatheter thrombolytic therapy for acute mesenteric and portal vein thrombosis [J]. Journal of Vascular and Interventional Radiology, 2005,16(5): 651-661.

[1647] Honore A, Trillard M, Ould-Hocine Z, et al. Contribution of urinary pneurnococcal antigen detection combined with the research of legionella antigen for diagnosis of pneumonia in hospitalized patients [J]. Pathologie Biologie, 2004,52(8): 429-433.

[1648] Hou RF, Tu WC, Lee HH, et al. Elevation of plasminogen activators in cerebrospinal fluid of mice with eosinophilic meningitis caused by Angiostrongylus cantonensis [J]. International Journal for Parasitology, 2004,34(12): 1355-1364.

[1649] Hryszko T, Inaba K, Ihara H, et al. Nafamostat attenuated the impairment of fibrinolysis in animal sepsis model by suppressing the increase of plasminogen activator inhibitor type 1 [J]. Journal of Trauma-Injury Infection and Critical Care, 2006,60(4): 859-864.

[1650] Hsieh YS, Yang SF, Lue KH, et al. Upregulation of urokinase-type plasminogen activator and inhibitor and gelatinase expression via 3 mitogen-activated protein kinases and PI3K pathways during the early development of osteoarthritis [J]. Journal of Rheumatology, 2007,34(4): 785-793.

[1651] Hsieh YS, Yang SF, Lue KH, et al. Clinical correlation with the PA/plasmin system in septic arthritis of the knee [J]. Clinical Orthopaedics and Related Research, 2006, (447): 172-178.

[1652] Iakhiaev A, Idell S. Activation and degradation of protein C by primary rabbit pleural mesothelial cells [J]. Lung, 2006,184(2): 81-88.

[1653] Iba T, Gando S, Murata A, et al. Predicting the severity of systemic inflammatory response syndrome (SIRS)-associated coagulopathy with hemostatic molecular markers and vascular endothelial injury markers [J]. Journal of Trauma-Injury Infection and Critical Care, 2007,63(5): 1093-1098.

[1654] Iba T, Kidokoro A, Fukunaga M, et al. Association between the severity of sepsis and the changes in hemostatic molecular markers and vascular endothelial damage markers [J]. Shock, 2005,23(1): 25-29.

[1655] Ichinose K, Okamoto T, Tashiro M, et al. The effects of pre-arrest heparin administration dose for cardiac arrest model using extracorporeal lung and a heart assist (ECLHA) in dogs [J]. Resuscitation, 2006,69(2): 311-318.

[1656] Imperatore F, Cuzzocrea S, De Lucia D, et al. Hyperbaric oxygen therapy prevents coagulation disorders in an experimental model of multiple organ failure syndrome [J]. Intensive Care Medicine, 2006,32(11): 1881-1888.

[1657] Incamps A, Hely-Joly F, Chagvardieff P, et al. Industrial process proteomics: Alfalfa protein patterns during wet fractionation processing [J]. Biotechnology and Bioengineering, 2005,91(4): 447-459.

[1658] Isordia-Salas I, Sainz IM, Pixley RA, et al. High molecular weight kininogen in inflammation and angiogenesis: a review of its properties and therapeutic applications [J]. Revista De Investigacion Clinica-Clinical and Translational Investigation, 2005,57(6): 802-813.

[1659] Jaffe A, Balfour-Lynn IM. Management of empyema in children [J]. Pediatric Pulmonology, 2005,40(2): 148-156.

[1660] Jancar S, Crespo MS. Immune complex-mediated tissue injury: a multistep paradigm [J]. Trends in Immunology, 2005,26(1): 48-55.

[1661] Jeanloz R, Celliers PM, Collins GW, et al. Achieving high-density states through shock-wave loading of precompressed samples [J]. Proceedings of the National Academy of Sciences of the United States of America, 2007,104(22): 9172-9177.

[1662] Jesmin S, Gando S, Matsuda N, et al. Temporal changes in pulmonary expression of key procoagulant molecules in rabbits with endotoxin-induced acute lung injury: elevated expression levels of protease-activated receptors [J]. Thrombosis and Haemostasis, 2004,92(5): 966-979.

[1663] Jesmin S, Gando S, Zaedi S, et al. Chronological expression of PAR isoforms in acute liver injury and its amelioration by PAR2 blockade in a rat model of sepsis [J]. Thrombosis and Haemostasis, 2006,96(6): 830-838.

[1664] Jessen KM, Lindboe SB, Petersen AL, et al. Common TNF-alpha, IL-1 beta, PAI-1, uPA, CD14 and TLR4 polymorphisms are not associated with disease severity or outcome from Gram negative sepsis [J]. Bmc Infectious Diseases, 2007,7.

[1665] Jimenez JJ, Iribarren JL, Lorente L, et al. Tranexamic acid attenuates inflammatory response in cardiopulmonary bypass surgery through blockade of fibrinolysis: a case control study followed by a randomized double-blind controlled trial [J]. Critical Care, 2007,11(6).

[1666] Jin T, Bokarewa M, Tarkowski A. Urokinase-type plasminogen activator, an endogenous antibiotic [J]. Journal of Infectious Diseases, 2005,192(3): 429-437.

[1667] Jobin MC, Fortin J, Willson PJ, et al. Acquisition of plasmin activity and induction of arachidonic acid release by Streptococcus suis in contact with human brain microvascular endothelial cells [J]. Fems Microbiology Letters, 2005,252(1): 105-111.

[1668] Joram N, Lopez E, Texereau J, et al. Genetic polymorphisms and infections [J]. Medecine Et Maladies Infectieuses, 2006,36(6): 314-321.

[1669] Joseph K, Kaplan AP. Formation of bradykinin: A major contributor to the innate inflammatory response[A]. In: Advances in Immunology, Vol 86 (Alt FW, ed), Vol. 86, 2005: 159-208.

[1670] Juodkazis S, Nishimura K, Tanaka S, et al. Laser-induced microexplosion confined in the bulk of a sapphire crystal: Evidence of multimegabar pressures [J]. Physical Review Letters, 2006,96(16).

[1671] Kahles H, Trobisch H, Kehren H. Disseminated coronary occlusions and massive pulmonary embolism in a 40-year-old woman [J]. Deutsche Medizinische Wochenschrift, 2006,131(13): 672-675.

[1672] Kalayoglu-Besisik S, Yenerel MN, Caliskan Y, et al. Time-related changes in the incidence, severity, and clinical outcome of hepatic veno-occlusive disease in hematopoietic stem cell transplantation patients during the past 10 years [J]. Transplantation Proceedings, 2005,37(5): 2285-2289.

[1673] Kanefsky J, Lenburg M, Hai CM. Cholinergic receptor and cyclic stretch-mediated inflammatory gene expression in intact ASM [J]. American Journal of Respiratory Cell and Molecular Biology, 2006,34(4): 417-425.

[1674] Kang J, Kamal A, Burrows FJ, et al. Inhibition of neuroblastoma xenograft growth by Hsp90 inhibitors [J]. Anticancer Research, 2006,26(3A): 1903-1908.

[1675] Kanno Y, Matsuno H. The possibility of novel antiplatelet peptides: The physiological effects of low molecular weight HSPs on platelets [J]. Current Pharmaceutical Design, 2006,12(7): 887-892.

[1676] Karamarkovic A, Radenkovic D, Milic N, et al. Protein C as an early marker of severe septic complications in diffuse secondary peritonitis [J]. World Journal of Surgery, 2005,29(6): 759-765.

[1677] Kasanuki H, Honda T, Haze K, et al. A large-scale prospective cohort study on the current status of therapeutic modalities for acute myocardial infarction in Japan: Rationale and initial results of the HIJAMI Registry [J]. American Heart Journal, 2005,150(3): 411-418.

[1678] Kashani A, Gibson CM, Murphy SA, et al. Angiography and revascularization in patients with heart failure following fibrinalytic therapy for ST-elevation acute myocardial infarction [J]. American Journal of Cardiology, 2005,95(2): 228-233.

[1679] Katoh M, Haage P, Spuentrup E, et al. A porcine deep vein thrombosis model for magnetic resonance-guided monitoring of different thrombectomy procedures [J]. Investigative Radiology, 2007,42(11): 727-731.

[1680] Katona L, Nagy B, Kappelaayer J, et al. Factor XIII in bronchoalveolar lavage fluid from children with chronic bronchoalveolar inflammation [J]. Journal of Thrombosis and Haemostasis, 2005,3(7): 1407-1413.

[1681] Keller TT, van der Sluijs KF, de Kruif MD, et al. Effects on coagulation and fibrinolysis induced by influenza in mice with a reduced capacity to generate activated protein C and a deficiency in plasminogen activator inhibitor type 1 [J]. Circulation Research, 2006,99(11): 1261-1269.

[1682] Kelly RV, Crouch E, Krumnacher H, et al. Safety of adjunctive intracoronary thrombolytic therapy during complex percutaneous coronary intervention: Initial experience with intracoronary tenecteplase [J]. Catheterization and Cardiovascular Interventions, 2005,66(3): 327-332.

[1683] Knoebl PN. Is there an association between hemostatic abnormalities and the outcome of acute lung injury? [J]. Critical Care Medicine, 2007,35(8): 1980-1982.

[1684] Kofoed K, Andersen O, Kronborg G, et al. Use of plasma C-reactive protein, procalcitonin, neutrophils, macrophage migration inhibitory factor, soluble urokinase-type plasminogen activator receptor, and soluble triggering receptor expressed on myeloid cells-1 in combination to diagnose infections: a prospective study [J]. Critical Care, 2007,11(2).

[1685] Kofoed K, Schneider UV, Scheel T, et al. Development and validation of a multiplex add-on assay for sepsis biomarkers using xMAP technology [J]. Clinical Chemistry, 2006,52(7): 1284-1293.

[1686] Koga F, Tsutsumi S, Neckers LM. Low dose geldanamycin inhibits hepatocyte growth factor and hypoxia-stimulated invasion of cancer cells [J]. Cell Cycle, 2007,6(11): 1393-1402.

[1687] Koh HJ, Cheng LY, Bessho K, et al. Intraocular properties of urokinase-derived antiangiogenic angstrom 6 peptide in rabbits [J]. Journal of Ocular Pharmacology and Therapeutics, 2004,20(5): 439-449.

[1688] Kolls JK. Balancing mucosal immunity: Caught between CYLD and Charybdis [J]. Immunity, 2007,27(2): 187-189.

[1689] Koppl J, Karovic D, Podhoransky B, et al. Hemodynamic monitoring using PiCCO (R) system in a 10 months old infant suffering from serious burn injury [J]. Bratislava Medical Journal-Bratislavske Lekarske Listy, 2007,108(8): 359-363.

[1690] Kucher N. Catheter embolectomy for acute pulmonary embolism [J]. Chest, 2007,132(2): 657-663.

[1691] Kukla P, Malarczyk-Zaher B, Szczuka K, et al. Massive pulmonary embolism treated successfully with embolectomy following failed thrombolysis - the role of repeated spiral computerised tomography and echocardiography - a case report [J]. Kardiologia Polska, 2007,65(1): 58-62.

[1692] Kunadian B, Sutton AGC, Vijayalakshmi K, et al. Early invasive versus conservative treatment in patients with failed fibrinolysis-no late survival benefit: The final analysis of the Middlesbrough Early Revascularisation to Limit Infarction (MERLIN) randomized trial [J]. American Heart Journal, 2007,153(5): 763-771.

[1693] Kuo GP, Brodsky RA, Kim HS. Catheter-directed thrombolysis and thrombectomy for the Budd-Chiari syndrome in paroxysmal nocturnal hemoglobinuria in three patients [J]. Journal of Vascular and Interventional Radiology, 2006,17(2): 383-387.

[1694] Kurt BA, Winterhalter KM, Connors RH, et al. Therapy of parapneumonic effusions in children: Video-assisted thoracoscopic surgery versus conventional thoracostomy drainage [J]. Pediatrics, 2006,118(3): E547-E553.

[1695] Kushi H, Miki T, Okamaoto K, et al. Early hemoperfusion with an immobilized polymyxin B fiber column eliminates humoral mediators and improves pulmonary oxygenation [J]. Critical Care, 2005,9(6): R653-R661.

[1696] Kushi H, Nakahara J, Miki T, et al. Hemoperfusion with an immobilized polymyxin B fiber column inhibits activation of vascular endothelial cells [J]. Therapeutic Apheresis and Dialysis, 2005,9(4): 303-307.

[1697] Kwak SH, Wang XQ, He Q, et al. Plasminogen activator inhibitor-I potentiates LPS-induced neutrophil activation through a JNK-mediated pathway [J]. Thrombosis and Haemostasis, 2006,95(5): 829-835.

[1698] Lagoa CE, Vodovotz Y, Stolz DB, et al. The role of hepatic type 1 plasminogen activator inhibitor (PAI-1) during murine hemorrhagic shock [J]. Hepatology, 2005,42(2): 390-399.

[1699] Lakobishvili Z, Hasdai D. Cardiogenic shock: Treatment [J]. Medical Clinics of North America, 2007,91(4): 713-+.

[1700] Langer F, Amirkhosravi A, Loges S, et al. An in vitro study on the mechanisms of coagulation activation in acute myelogenous leukemia (AML): role of tissue factor regulation by cytotoxic drugs and GM-CSF [J]. Thrombosis and Haemostasis, 2004,92(5): 1136-1146.

[1701] Latacz P, Rostoff P, Wyderka R, et al. Massive pulmonary embolism mimicking ST-elevation acute coronary syndrome successfully treated with hybrid therapy in a trauma patient receiving nadroparin: diagnostic and therapeutic dilemmas [J]. Kardiologia Polska, 2007,65(10): 1235-1242.

[1702] Lathem WW, Price PA, Miller VL, et al. A plasminogen-activating protease specifically controls the development of primary pneumonic plague [J]. Science, 2007,315(5811): 509-513.

[1703] Lattouf JB, Srinivasan R, Pinto PA, et al. Mechanisms of Disease: the role of heat-shock protein 90 in genitourinary malignancy [J]. Nature Clinical Practice Urology, 2006,3(11): 590-601.

[1704] Laurent GJ. No bit PARt for PAR-1 [J]. American Journal of Respiratory Cell and Molecular Biology, 2005,33(3): 213-215.

[1705] Lazar MH, Christensen PJ, Du M, et al. Plasminogen activator inhibitor-1 impairs alveolar epithelial repair by binding to vitronectin [J]. American Journal of Respiratory Cell and Molecular Biology, 2004,31(6): 672-678.

[1706] Lee AS, Mellins RB. Lung injury from smoke inhalation [J]. Paediatric Respiratory Reviews, 2006,7(2): 123-128.

[1707] Lee C, Xu DZ, Feketeova E, et al. Attenuation of shock-induced acute lung injury by sphingosine kinase inhibition [J]. Journal of Trauma-Injury Infection and Critical Care, 2004,57(5): 955-960.

[1708] Lee JS, Huang TQ, Kim TH, et al. Radiofrequency radiation does not induce stress response in human T-lymphocytes and rat primary astrocytes [J]. Bioelectromagnetics, 2006,27(7): 578-588.

[1709] Lee JS, Zhang MH, Yun EK, et al. Heat shock protein 27 interacts with vimentin and prevents insolubilization of vimentin subunits induced by cadmium [J]. Experimental and Molecular Medicine, 2005,37(5): 427-435.

[1710] Lemaire LC, de Kruif MD, Giebelen IA, et al. Dobutamine does not influence inflammatory pathways during human endotoxemia [J]. Critical Care Medicine, 2006,34(5): 1365-1371.

[1711] Leung C, Chang YC. Video-assisted thoracoscopic surgery in a 1-month-old infant with pleural empyema [J]. Journal of the Formosan Medical Association, 2006,105(11): 936-940.

[1712] Levi M. Disseminated intravascular coagulation: What's new? [J]. Critical Care Clinics, 2005,21(3): 449-+.

[1713] Levi M, de Jonge E, van der Poll T. Plasma and plasma components in the management of disseminated intravascular coagulation [J]. Best Practice & Research Clinical Haematology, 2006,19(1): 127-142.

[1714] Levi M, van der Poll T. Two-way interactions between inflammation and coagulation [J]. Trends in Cardiovascular Medicine, 2005,15(7): 254-259.

[1715] Levy EI, Mehta R, Gupta R, et al. Self-expanding stents for recanalization of acute cerebrovascular occlusions [J]. American Journal of Neuroradiology, 2007,28(5): 816-822.

[1716] Lijnen HR. Pleiotropic functions of plasminogen activator inhibitor-1 [J]. Journal of Thrombosis and Haemostasis, 2005,3(1): 35-45.

[1717] Lim JH, Stirling B, Derry J, et al. Tumor suppressor CYLD regulates acute lung injury in lethal Streptococcus pneumoniae infections [J]. Immunity, 2007,27(2): 349-360.

[1718] Lin X, Liang XX, Chen JS, et al. The effect of fibrinolytic enzyme FIIa from Agkistrodon ocutus venom on disseminated intravascular coagulation in rabbits [J]. Translational Research, 2007,150(5): 295-302.

[1719] Lindenblatt N, Menger MD, Klar E, et al. Systemic hypothermia increases PAI-1 expression and accelerates microvascular thrombus formation in endotoxemic mice [J]. Critical Care, 2006,10(5).

[1720] Liu DY, Li CS, Chen YL, et al. Nuclear import of proinflammatory transcription factors is required for massive liver apoptosis induced by bacterial lipopolysaccharide [J]. Journal of Biological Chemistry, 2004,279(46): 48434-48442.

[1721] Liu W, Zhu ZQ, Wang W, et al. Crucial roles of GATA-2 and SPI in adrenomedullin-affected expression of tissue factor pathway inhibitor in human umbilical vein endothelial cells exposed to lipopolysaccharide [J]. Thrombosis and Haemostasis, 2007,97(5): 839-846.

[1722] Liu ZH, Wei R, Wu YP, et al. Elevated plasma tissue-type plasminogen activator (t-PA) and soluble thrombomodulin in patients suffering from severe acute respiratory syndrome (SARS) as a possible index for prognosis and treatment strategy [J]. Biomedical and Environmental Sciences, 2005,18(4): 260-264.

[1723] Ma SF, Grigoryev DN, Taylor AD, et al. Bioinformatic identification of novel early stress response genes in rodent models of lung injury [J]. American Journal of Physiology-Lung Cellular and Molecular Physiology, 2005,289(3): L468-L477.

[1724] Macic-Dzankovic A, Pojskic B. Acute heart failure after myocardial infarction [J]. Bosnian Journal of Basic Medical Sciences, 2007,7(1): 40-47.

[1725] MacLaren R, Stringer KA. Emerging role of anticoagulants and fibrinolytics in the treatment of acute respiratory distress syndrome [J]. Pharmacotherapy, 2007,27(6): 860-873.

[1726] Madoiwa S, Nunomiya S, Ono T, et al. Plasminogen activator inhibitor 1 promotes a poor prognosis in sepsis-induced disseminated intravascular coagulation [J]. International Journal of Hematology, 2006,84(5): 398-405.

[1727] Maier LS, Teucher N, Dorge H, et al. Large emboli on their way through the heart - First live demonstration of large paradoxical embolisms through a patent foramen ovale [J]. European Journal of Echocardiography, 2007,8(2): 158-160.

[1728] Maison SF, Parker LL, Young L, et al. Overexpression of SK2 channels enhances efferent suppression of cochlear responses without enhancing noise resistance [J]. Journal of Neurophysiology, 2007,97(4): 2930-2936.

[1729] Maquerlot F, Galiacy S, Malo M, et al. Dual role for plasminogen activator inhibitor type 1 as soluble and as matricellular regulator of epithelial alveolar cell wound healing [J]. American Journal of Pathology, 2006,169(5): 1624-1632.

[1730] Maris NA, de Vos AF, Bresser P, et al. Salmeterol enhances pulmonary fibrinolysis in healthy volunteers [J]. Critical Care Medicine, 2007,35(1): 57-63.

[1731] Maris NA, de Vos AF, Bresser P, et al. Activation of coagulation and inhibition of fibrinolysis in the lung after inhalation of lipopolysaccharide by healthy volunteers [J]. Thrombosis and Haemostasis, 2005,93(6): 1036-1040.

[1732] Marshall JC, Vincent JL, Guyatt G, et al. Outcome measures for clinical research in sepsis: A report of the 2nd Cambridge Colloquium of the International Sepsis Forum [J]. Critical Care Medicine, 2005,33(8): 1708-1716.

[1733] Martin-Ventura JL, Nicolas V, Houard X, et al. Biological significance of decreased HSP27 in human atherosclerosis [J]. Arteriosclerosis Thrombosis and Vascular Biology, 2006,26(6): 1337-1343.

[1734] Maskell NA, Davies CWH, Nunn AJ, et al. UK controlled trial of intrapleural streptokinase for pleural infection [J]. New England Journal of Medicine, 2005,352(9): 865-874.

[1735] Masuya K, Okada H, Imai T, et al. A case of intractable methicillin-resistant Staphylococcus aureus sepsis after an operation for tetralogy of Fallot that was successfully resolved by combined treatment with vancomycin and urokinase [J]. Pediatrics International, 2006,48(2): 163-165.

[1736] Matejovic M, Krouzecky A, Martinkova V, et al. Effects of tempol, a free radical scavenger, on long-term hyperdynamic porcine bacteremia [J]. Critical Care Medicine, 2005,33(5): 1057-1063.

[1737] Matejovic M, Krouzecky A, Radej J, et al. Coagulation and endothelial dysfunction during longterm hyperdynamic porcine bacteremia - effects of selective inducible nitric oxide synthase inhibition [J]. Thrombosis and Haemostasis, 2007,97(2): 304-309.

[1738] Matsumoto T, Wada H, Nobori T, et al. Elevated plasma levels of fibrin degradation products by granulocyte-derived elastase in patients with disseminated intravascular coagulation [J]. Clinical and Applied Thrombosis-Hemostasis, 2005,11(4): 391-400.

[1739] Matsuno H. alpha 2-antiplasmin on cardiovascular diseases [J]. Current Pharmaceutical Design, 2006,12(7): 841-847.

[1740] Matsuoka H, Sisson TH, Nishiuma T, et al. Plasminogen-mediated activation and release of hepatocyte growth factor from extracellular matrix [J]. American Journal of Respiratory Cell and Molecular Biology, 2006,35(6): 705-713.

[1741] Matsuu A, Kanda T, Sugiyama A, et al. Mitral stenosis with bacterial myocarditis in a cat [J]. Journal of Veterinary Medical Science, 2007,69(11): 1171-1174.

[1742] Mehdiratta M, Murphy C, At-Harthil A, et al. Myocardial infarction following t-PA for acute stroke [J]. Canadian Journal of Neurological Sciences, 2007,34(4): 417-420.

[1743] Mehta RH, Califf RM, Garg J, et al. Association of height with outcomes in patients with acute myocardial infarction receiving reperfusion therapy [J]. American Journal of Cardiology, 2005,95(11): 1371-1375.

[1744] Mehta RH, Granger CB, Alexander KP, et al. Reperfusion strategies for acute myocardial infarction in the elderly - Benefits and risks [J]. Journal of the American College of Cardiology, 2005,45(4): 471-478.

[1745] Mello P, Sharma VK, Dellinger RP. Shock overview [J]. Seminars in Respiratory and Critical Care Medicine, 2004,25(6): 619-628.

[1746] Mendoza CE, Bhatt MR, Virani S, et al. Management of failed thrombolysis after acute myocardial infarction: An overview of current treatment options [J]. International Journal of Cardiology, 2007,114(3): 291-299.

[1747] Meneveau N, Seronde MF, Blonde MC, et al. Management of unsuccessful thrombolysis in acute massive pulmonary embolism [J]. Chest, 2006,129(4): 1043-1050.

[1748] Milani V, Frankenberger B, Heinz O, et al. Melanoma-associated antigen tyrosinase but not Melan-A/MART-1 expression and presentation dissociate during the heat shock response [J]. International Immunology, 2005,17(3): 257-268.

[1749] Milliat F, Francois A, Isoir M, et al. Influence of endothelial cells on vascular smooth muscle cells phenotype after irradiation - Implication in radiation-induced vascular damages [J]. American Journal of Pathology, 2006,169(4): 1484-1495.

[1750] Mischke R. Acute haemostatic changes in accidentally traumatised dogs [J]. Veterinary Journal, 2005,169(1): 60-64.

[1751] Misthos P, Sepsas E, Konstantinou M, et al. Early use of intrapleural fibrinolytics in the management of postpneumonic empyema. A prospective study [J]. European Journal of Cardio-Thoracic Surgery, 2005,28(4): 599-603.

[1752] Moller HJ, Moestrup SK, Weis N, et al. Macrophage serum markers in pneumococcal bacteremia: Prediction of survival by soluble CD163 [J]. Critical Care Medicine, 2006,34(10): 2561-2566.

[1753] Monecke S, Kuhnert P, Hotzel H, et al. Microarray based study on virulence-associated genes and resistance determinants of Staphylococcus aureus isolates from cattle [J]. Veterinary Microbiology, 2007,125(1-2): 128-140.

[1754] Moon EJ, Brizel DM, Chi JTA, et al. The potential role of intrinsic hypoxia markers as prognostic variables in cancer [J]. Antioxidants & Redox Signaling, 2007,9(8): 1237-1294.

[1755] Mosnier LO, Griffin JH. Protein C anticoagulant activity in relation to anti-inflammatory and anti-apoptotic activities [J]. Frontiers in Bioscience, 2006,11: 2381-2399.

[1756] Moulder JE, Cohen EP. Future strategies for mitigation and treatment of chronic radiation-induced normal tissue injury [J]. Seminars in Radiation Oncology, 2007,17(2): 141-148.

[1757] Mousa A, Henderson P, Dayal R, et al. Endoluminal Recanalization in a Patient with Phlegmasia Cerulea Dolens Using a Multimodality Approach [J]. Vascular, 2005,13(5): 313-317.

[1758] Mueller L, Goettsche J, Abdulgawad A, et al. Tumor growth-promoting cellular host response during liver atrophy after portal occlusion [J]. Liver International, 2005,25(5): 994-1001.

[1759] Muiesan P, Girlanda R, Jassem W, et al. Single-center experience with liver transplantation from controlled non-heartbeating donors - A viable source of grafts [J]. Annals of Surgery, 2005,242(5): 732-738.

[1760] Muro S, Muzykantov VR. Targeting of antioxidant and anti-thrombotic drugs to endothelial cell adhesion molecules [J]. Current Pharmaceutical Design, 2005,11(18): 2383-2401.

[1761] Murthy SC, Okereke I, Mason DP, et al. A simple solution for complicated pleural effusions [J]. Journal of Thoracic Oncology, 2006,1(7): 697-700.

[1762] Nagao H, Nakamura KG, Kondo K, et al. Hugoniot measurement of diamond under laser shock compression up to 2 TPa [J]. Physics of Plasmas, 2006,13(5).

[1763] Nakamura M, Nakanishi N, Yamada N, et al. Effectiveness and safety of the thrombolytic therapy for acute pulmonary thromboembolism: results of a multicenter registry in the Japanese Society of Pulmonary Embolism Research [J]. International Journal of Cardiology, 2005,99(1): 83-89.

[1764] Nakamura T, Suzuki H, Wada Y, et al. Fucoidan induces nitric oxide production via p38 mitogen-activated protein kinase and NF-kappa B-dependent signaling pathways through macrophage scavenger receptors [J]. Biochemical and Biophysical Research Communications, 2006,343(1): 286-294.

[1765] Nielsen JS, Larsson A, Rix T, et al. The effect of activated protein C on plasma cytokine levels in a porcine model of acute endotoxemia [J]. Intensive Care Medicine, 2007,33(6): 1085-1093.

[1766] Nishiuma T, Sisson TH, Subbotina N, et al. Localization of plasminogen activator activity within normal and injured lungs by in situ zymography [J]. American Journal of Respiratory Cell and Molecular Biology, 2004,31(5): 552-558.

[1767] Nobuhisa T, Naomoto Y, Okawa T, et al. Translocation of heparanase into nucleus results in cell differentiation [J]. Cancer Science, 2007,98(4): 535-540.

[1768] Nor AM, Davis J, Sen B, et al. The Recognition of Stroke in the Emergency Room (ROSIER) scale: development and validation of a stroke recognition instrument [J]. Lancet Neurology, 2005,4(11): 727-734.

[1769] Novoselova TV, Margulis BA, Novoselov SS, et al. Treatment with extracellular HSP70/HSC70 protein can reduce polyglutamine toxicity and aggregation [J]. Journal of Neurochemistry, 2005,94(3): 597-606.

[1770] Nowak P, Zbikowska HM, Ponczek M, et al. Different vulnerability of fibrinogen subunits to oxidative/nitrative modifications induced by peroxynitrite: Functional consequences [J]. Thrombosis Research, 2007,121(2): 163-174.

[1771] Nunes H. Idiopathic interstitial pneumopathies [J]. Revue Des Maladies Respiratoires, 2005,22(5): S112-S118.

[1772] Oguzkaya F, Akcali Y, Bilgin M. Videothoracoscopy versus intrapleural streptokinase for management of post traumatic retained haemothorax: a retrospective study of 65 cases [J]. Injury-International Journal of the Care of the Injured, 2005,36(4): 526-529.

[1773] Ohta K, Okoshi R, Wakabayashi M, et al. Geldanamycin, a heat-shock protein 90-binding agent, induces thymocyte apoptosis through destabilization of Lck in presence of 12-O-tetradecanoylphorbol 13-acetate [J]. Biomedical Research-Tokyo, 2007,28(1): 33-42.

[1774] Onder AM, Kato T, Simon N, et al. Prevention of catheter-related bacteremia in pediatric intestinal transplantation/short gut syndrome children with long-term central venous catheters [J]. Pediatric Transplantation, 2007,11(1): 87-93.

[1775] Ondrouskova E, Povolna K, Vana P, et al. A proteomic analysis of protein variations during differentiation of v-myb-transformed monoblasts [J]. Leukemia Research, 2007,31(2): 221-229.

[1776] Ozaki N, Ono T, Takamatsu K, et al. Equation-of-state measurements for polystyrene at multi-TPa pressures in laser direct-drive experiments [J]. Physics of Plasmas, 2005,12(12).

[1777] Pain JC. Quantum-statistical equation-of-state models of dense plasmas: High-pressure hugoniot shock adiabats [J]. Contributions to Plasma Physics, 2007,47(6): 421-434.

[1778] Paran H, Gutman M, Mayo A. The effect of aprotinin in a model of uncontrolled hemorrhagic shock [J]. American Journal of Surgery, 2005,190(3): 463-466.

[1779] Park J, Jung Y, Kim J, et al. TC1 (C8orf4) is upregulated by cellular stress and mediates heat shock response [J]. Biochemical and Biophysical Research Communications, 2007,360(2): 447-452.

[1780] Parmar N, Albisetti M, Berry LR, et al. The fibrinolytic system in newborns and children [J]. Clinical Laboratory, 2006,52(3-4): 115-124.

[1781] Pauluhn J. Acute nose-only exposure of rats to phosgene. Part II. Concentration x time dependence of changes in bronchoalveolar lavage during a follow-up period of 3 months [J]. Inhalation Toxicology, 2006,18(9): 595-607.

[1782] Peng ZY, Kiss JE, Cortese-Hassett A, et al. Plasma filtration on mediators of thrombotic microangiopathy: An in vitro study [J]. International Journal of Artificial Organs, 2007,30(5): 401-406.

[1783] Perel P, Roberts I, Sena E, et al. Comparison of treatment effects between animal experiments and clinical trials: systematic review [J]. Bmj-British Medical Journal, 2007,334(7586): 197-200.

[1784] Perkowski SZ, Kowalska MA, Albelda SM, et al. Expression of urokinase-type plasminogen activator in platelets decreases oxygen-induced lung injury in mice [J]. Blood, 2004,104(11): 198A-198A.

[1785] Petersen MB. The effect of vasopressin and related compounds at V-1a and V-2 receptors in animal models relevant to human disease [J]. Basic & Clinical Pharmacology & Toxicology, 2006,99(2): 96-103.

[1786] Pleyer L, Went P, Russ G, et al. Massive infiltration of bone marrow in colon carcinoma after treatment with activated protein C [J]. Wiener Klinische Wochenschrift, 2007,119(7-8): 254-258.

[1787] Pons D, Monraats PS, de Maat MPM, et al. The influence of established genetic variation in the haemostatic system on clinical restenosis after percutaneous coronary interventions [J]. Thrombosis and Haemostasis, 2007,98(6): 1323-1328.

[1788] Prelog M, Fischer H, Gassner I, et al. Bilateral renal vein thrombosis in a twin newborn without known risk factors [J]. Clinical Nephrology, 2006,66(2): 135-139.

[1789] Quin LR, Onwubiko C, Moore QC, et al. Factor H binding to PspC of Streptococcus pneumoniae increases adherence to human cell lines in vitro and enhances invasion of mouse lungs in vivo [J]. Infection and Immunity, 2007,75(8): 4082-4087.

[1790] Rastogi S, Shukla Y, Paul BN, et al. Protective effect of Ocimum sanctum on 3-methylcholanthrene, 7,12-dimethylbenz(a)anthracene and aflatoxin B1 induced skin tumorigenesis in mice [J]. Toxicology and Applied Pharmacology, 2007,224(3): 228-240.

[1791] Renckens R, Pater JM, van der Poll T. Plasminogen activator inhibitor type-1-deficient mice have an enhanced IFN-gamma response to lipopolysaccharide and staphylococcal enterotoxin B [J]. Journal of Immunology, 2006,177(11): 8171-8176.

[1792] Renckens R, Roelofs J, Bonta PI, et al. Plasminogen activator inhibitor type 1 is protective during severe Gram-negative pneumonia [J]. Blood, 2007,109(4): 1593-1601.

[1793] Renckens R, Roelofs J, de Waard V, et al. The role of plasminogen activator inhibitor type 1 in the inflammatory response to local tissue injury [J]. Journal of Thrombosis and Haemostasis, 2005,3(5): 1018-1025.

[1794] Renckens R, Roelofs J, Florquin S, et al. Endogenous tissue-type plasminogen activator is protective during Escherichia coli-induced abdominal sepsis in mice [J]. Journal of Immunology, 2006,177(2): 1189-1196.

[1795] Renckens R, Roelofs J, Florquin S, et al. Urokinase-type plasminogen activator receptor plays a role in neutrophil migration during lipopolysaccharide-induced peritoneal inflammation but not during Escherichia coli-induced peritonitis [J]. Journal of Infectious Diseases, 2006,193(4): 522-530.

[1796] Rijneveld AW, de Vos AF, Florquin S, et al. CD11b limits bacterial outgrowth and dissemination during murine pneumococcal pneumonia [J]. Journal of Infectious Diseases, 2005,191(10): 1755-1760.

[1797] Ripley RT, Cothren CC, Moore EE, et al. Streptococcus milleri infections of the pleural space: operative management predominates [J]. American Journal of Surgery, 2006,192(6): 817-820.

[1798] Rubenstein DS, Diaz LA. Pemphigus antibody induced phosphorylation of keratinocyte proteins [J]. Autoimmunity, 2006,39(7): 577-586.

[1799] Rucker M, Schafer T, Scheuer C, et al. Local heat shock priming promotes recanalization of thromboembolized microvasculature by upregulation of plasminogen activators [J]. Arteriosclerosis Thrombosis and Vascular Biology, 2006,26(7): 1632-1639.

[1800] Saadoun D, Bieche I, Authier FJ, et al. Role of matrix metalloproteinases, proinflammatory cytokines, and oxidative stress-derived molecules in hepatitis C virus-associated mixed cryoglobulinemia vasculitis neuropathy [J]. Arthritis and Rheumatism, 2007,56(4): 1315-1324.

[1801] Sakagami Y, Yamamoto K, Sugiura S, et al. Essential roles of Homer-1a in homeostatic regulation of pyramidal cell excitability: a possible link to clinical benefits of electroconvulsive shock [J]. European Journal of Neuroscience, 2005,21(12): 3229-3239.

[1802] Sakamoto Y, Mashiko K, Matsumoto H, et al. Relationship between effect of polymyxin B-immobilized fiber and high-mobility group box-1 protein in septic shock patients [J]. Asaio Journal, 2007,53(3): 324-328.

[1803] Samanta A, Ali SM, Ghosh SK. Universal scaling laws of diffusion: Application to liquid metals [J]. Journal of Chemical Physics, 2005,123(8).

[1804] Sanderson S, Valenti M, Gowan S, et al. Benzoquinone ansamycin heat shock protein 90 inhibitors modulate multiple functions required for tumor angiogenesis [J]. Molecular Cancer Therapeutics, 2006,5(3): 522-532.

[1805] Sandoval JA, Hoelz DJ, Woodruff HA, et al. Novel peptides secreted from human neuroblastoma: useful clinical tools? [J]. Journal of Pediatric Surgery, 2006,41(1): 245-251.

[1806] Sapru A, Wiemels JL, Witte JS, et al. Acute lung injury and the coagulation pathway: potential role of gene polymorphisms in the protein C and fibrinolytic pathways [J]. Intensive Care Medicine, 2006,32(9): 1293-1303.

[1807] Sardet A, Marteletti O, Maboudou I. Pneumococcus and lower repiratory tract infections in children: management of pleural effusion [J]. Revue Francaise D Allergologie Et D Immunologie Clinique, 2005,45(7): 525-529.

[1808] Satoh J, Onoue H, Arima K, et al. The 14-3-3 protein forms a molecular complex with heat shock protein Hsp60 and cellular prion protein [J]. Journal of Neuropathology and Experimental Neurology, 2005,64(10): 858-868.

[1809] Schellongowski P, Bauer E, Holzinger U, et al. Treatment of adult patients with sepsis-induced coagulopathy and purpura fulminans using a plasma-derived protein C concentrate (Ceprotin (R)) [J]. Vox Sanguinis, 2006,90(4): 294-301.

[1810] Schiza S, Siafakas NM. Clinical presentation and management of empyema, lung abscess and pleural effusion [J]. Current Opinion in Pulmonary Medicine, 2006,12(3): 205-211.

[1811] Schmidt R, Redecke V, Breitfeld Y, et al. EMMPRIN (CID 147) is a central activator of extracellular matrix degradation by Chlamydia pneumoniae-infected monocytes. Implications for plaque rupture [J]. Thrombosis and Haemostasis, 2006,95(1): 151-158.

[1812] Schneider H, Weber F, Paranskaja L, et al. Interventional treatment of ST-segment elevation myocardial infarction according to the guidelines in a rural area facilitated by network collaboration [J]. Deutsche Medizinische Wochenschrift, 2004,129(41): 2162-2166.

[1813] Schultz MJ, Haitsma JJ, Zhang HB, et al. Pulmonary coagulopathy as a new target in therapeutic studies of acute lung injury or pneumonia - A review [J]. Critical Care Medicine, 2006,34(3): 871-877.

[1814] Sen B, Mahadevan B, DeMarini DM. Transcriptional responses to complex mixtures - A review [J]. Mutation Research-Reviews in Mutation Research, 2007,636(1-3): 144-177.

[1815] Shavelle DM, Salami A, Abdelkarim M, et al. Rescue percutaneous coronary intervention for failed thrombolysis [J]. Catheterization and Cardiovascular Interventions, 2006,67(2): 214-220.

[1816] Shaw D, Russell JA, Walley KR. Pharmacogenomics in sepsis and septic shock [J]. Drug Development Research, 2005,64(4): 181-194.

[1817] Shen G, Zhao R. Oxidized LDL increased the expression of heat shock factor-1 in vascular endothelial cells and its relationship with plasminogen activator inhibitor-1 [J]. Atherosclerosis Supplements, 2006,7(3): 88-88.

[1818] Shen GX, Zhao RZ. Involvement of heat shock factor-1 in glycated low density lipoprotein-induced upregulation of plasminogen activator inhibitor-1 in vascular endothelial cells [J]. Diabetes, 2007,56: A174-A174.

[1819] Shen YH, Xie Q, Norberg M, et al. Geldanamycin derivative inhibition of HGF/SF-mediated Met tyrosine kinase receptor-dependent urokinase-plasminogen activation [J]. Bioorganic & Medicinal Chemistry, 2005,13(16): 4960-4971.

[1820] Shenberger JS, Zhang LQ, Powell RJ, et al. Hyperoxia enhances VEGF release from A549 cells via post-transcriptional processes [J]. Free Radical Biology and Medicine, 2007,43(5): 844-852.

[1821] Shetty S, Gyetko MR, Mazar AP. Induction of p53 by urokinase in lung epithelial cells [J]. Journal of Biological Chemistry, 2005,280(30): 28133-28141.

[1822] Shukla M, Kashyap Y, Sarkar PS, et al. Laser induced shock pressure multiplication in multi layer thin foil targets [J]. Nuclear Fusion, 2006,46(4): 419-431.

[1823] Siddiqui F, Ehrhart EJ, Charles B, et al. Anti-angiogenic effects of interleukin-12 delivered by a novel hyperthermia induced gene construct [J]. International Journal of Hyperthermia, 2006,22(7): 587-606.

[1824] Simon A, Bode U, Beutel K. Diagnosis and treatment of catheter-related infections in paediatric oncology: an update [J]. Clinical Microbiology and Infection, 2006,12(7): 606-620.

[1825] Singh M, White J, Hasdai D, et al. Long-term outcome and its predictors among patients with ST-Segment elevation myocardial infarction complicated by shock: Insights from the GUSTO-I trial [J]. Journal of the American College of Cardiology, 2007,50(18): 1752-1758.

[1826] Sinn DI, Chu K, Lee ST, et al. Pharmacological induction of heat shock protein exerts neuroprotective effects in experimental intracerebral hemorrhage [J]. Brain Research, 2007,1135(1): 167-176.

[1827] Sinn DI, Kim SJ, Chu K, et al. Valproic acid-mediated neuroprotection in intracerebral hemorrhage via histone deacetylase inhibition and transcriptional activation [J]. Neurobiology of Disease, 2007,26(2): 464-472.

[1828] Sipahi T, Pocan H, Akar N. Effect of various genetic polymorphisms on the incidence and outcome of severe sepsis [J]. Clinical and Applied Thrombosis-Hemostasis, 2006,12(1): 47-54.

[1829] Sisson TH, Simon RH. The plasminogen activation system in lung disease [J]. Current Drug Targets, 2007,8(9): 1016-1029.

[1830] Slofstra SH, Groot AP, Obdeijn MHP, et al. Gene expression profiling identifies C/EBP delta as a candidate regulator of endotoxin-induced disseminated intravascular coagulation [J]. American Journal of Respiratory and Critical Care Medicine, 2007,176(6): 602-609.

[1831] Smyth DS, Kennedy J, Twohig J, et al. Staphylococcus aureus isolates from Irish domestic refrigerators possess novel enterotoxin and enterotoxin-like genes and are clonal in nature [J]. Journal of Food Protection, 2006,69(3): 508-515.

[1832] Song YL, Lynch SV, Flanagan J, et al. Increased plasminogen activator inhibitor-1 concentrations in bronchoalveolar lavage fluids are associated with increased mortality in a cobort of patients with Pseudomonas aeruginosa [J]. Anesthesiology, 2007,106(2): 252-261.

[1833] Sosothikul D, Seksarn P, Pongsewalak S, et al. Activation of endothelial cells, coagulation and fibrinolysis in children with Dengue virus infection [J]. Thrombosis and Haemostasis, 2007,97(4): 627-634.

[1834] Spronk PE, Rommes JH, Schaar C, et al. Thrombolysis in fulminant purpura: Observations on changes in microcirculatory perfusion during succesful treatment [J]. Thrombosis and Haemostasis, 2006,95(3): 576-578.

[1835] Stief TW, Ijagha O, Weiste B, et al. Analysis of hemostasis alterations in sepsis [J]. Blood Coagulation & Fibrinolysis, 2007,18(2): 179-186.

[1836] Suchar AM, Zureikat AH, Glynn L, et al. Ready for the frontline: Is early thoracoscopic decortication the new standard of care for advanced pneumonia with empyema? [J]. American Surgeon, 2006,72(8): 688-692.

[1837] Sutton AGC, Campbell PG, Graham R, et al. One year results of the Middlesbrough early revascularisation to limit infarction (MERLIN) trial [J]. Heart, 2005,91(10): 1330-1337.

[1838] Sutton AGC, Finn P, Hall JA, et al. Predictors of outcome after percutaneous treatment for cardiogenic shock [J]. Heart, 2005,91(3): 339-344.

[1839] Swift DC, Tierney TE, Luo SN, et al. Dynamic response of materials on subnanosecond time scales, and beryllium properties for inertial confinement fusion [J]. Physics of Plasmas, 2005,12(5).

[1840] Takai S, Matsushima-Nishiwaki R, Tokuda H, et al. Protein kinase C delta regulates the phosphorylation of heat shock protein 27 in human hepatocellular carcinoma [J]. Life Sciences, 2007,81(7): 585-591.

[1841] Tan H, Kizilkaya M, Alper F, et al. Thrombolytic therapy with tissue plasminogen activator for superior vena cava thrombosis in an infant with sepsis [J]. Acta Paediatrica, 2005,94(2): 239-241.

[1842] Tang H, Ivanciu L, Popescu N, et al. Sepsis-induced coagulation in the baboon lung is associated with decreased tissue factor pathway inhibitor [J]. American Journal of Pathology, 2007,171(3): 1066-1077.

[1843] Tanriverdi F, Gul A, Gul I, et al. Massive cardiac thrombosis in a patient with Sheehan's syndrome [J]. Endocrine Journal, 2005,52(6): 709-714.

[1844] Tayara W, Starling RC, Yamani MH, et al. Improved survival after acute myocardial infarction complicated by cardiogenic shock with circulatory support and transplantation: Comparing aggressive intervention with conservative treatment [J]. Journal of Heart and Lung Transplantation, 2006,25(5): 504-509.

[1845] ter Horst SAJ, Wagenaar GTM, de Boer E, et al. Pentoxifylline reduces fibrin deposition and prolongs survival in neonatal hyperoxic lung injury [J]. Journal of Applied Physiology, 2004,97(5): 2014-2019.

[1846] ter Horst SAJ, Walther FJ, Poorthuis B, et al. Inhaled nitric oxide attenuates pulmonary inflammation and fibrin deposition and prolongs survival in neonatal hyperoxic lung injury [J]. American Journal of Physiology-Lung Cellular and Molecular Physiology, 2007,293(1): 35-44.

[1847] Terao Y, Yamaguchi M, Hamada S, et al. Multifunctional glyceraldehyde-3-phosphate dehydrogenase of Streptococcus pyogenes is essential for evasion from neutrophils [J]. Journal of Biological Chemistry, 2006,281(20): 14215-14223.

[1848] Thimmulappa RK, Fuchs RJ, Malhotra D, et al. Preclinical evaluation of targeting the Nrf2 pathway by triterpenoids (CDDO-Im and CDDO-Me) for protection from LPS-induced inflammatory response and reactive oxygen species in human peripheral blood mononuclear cells and neutrophils [J]. Antioxidants & Redox Signaling, 2007,9(11): 1963-1970.

[1849] Tokuda Y, Matsushima D, Stein GH, et al. Intrapleural fibrinolytic agents for empyema and complicated parapneumonic effusions - A meta-analysis [J]. Chest, 2006,129(3): 783-790.

[1850] Toltl LJ, Shin LYY, Liaw PCY. Activated protein C in sepsis and beyond: Update 2006 [J]. Frontiers in Bioscience-Landmark, 2007,12: 1963-1972.

[1851] Toumpoulis IK, Anagnostopoulos CE, Katritsis DG, et al. The impact of preoperative thrombolysis on long-term survival after coronary artery bypass grafting [J]. Circulation, 2005,112(9): I351-I357.

[1852] Trzos E, Kurpesa M, Bednarkiewicz Z, et al. Impact of the time to reperfusion on early outcomes in patients with acute myocardial infarction undergoing primary angioplasty [J]. Kardiologia Polska, 2007,65(11): 1296-1306.

[1853] Tsuruta Y, Park YJ, Siegal GP, et al. Involvement of vitronectin in lipopolysaccaride-induced acute lung injury [J]. Journal of Immunology, 2007,179(10): 7079-7086.

[1854] Twomley KM, Rao SV, Becker RC. Proinflammatory, immunomodulating, and prothrombotic properties of anemia and red blood cell transfusions [J]. Journal of Thrombosis and Thrombolysis, 2006,21(2): 167-174.

[1855] Uchiyama T, Atsuta H, Utsugi T, et al. HSF1 and constitutively active HSF1 improve vascular endothelial function (heat shock proteins improve vascular endothelial function) [J]. Atherosclerosis, 2007,190(2): 321-329.

[1856] Uchiyama T, Atsuta H, Utsugi T, et al. Simvastatin induces heat shock factor 1 in vascular endothelial cells [J]. Atherosclerosis, 2006,188(2): 265-273.

[1857] Van de Werf F, Ross A, Armstrong P, et al. Primary versus tenecteplase-facilitated percutaneous coronary intervention in patients with ST-segment elevation acute myocardial infarction (ASSENT-4 PCI): randomised trial [J]. Lancet, 2006,367(9510): 569-578.

[1858] van der Poll T, Levi M, Nick JA, et al. Activated protein C inhibits local coagulation after intrapulmonary delivery of endotoxin in humans [J]. American Journal of Respiratory and Critical Care Medicine, 2005,171(10): 1125-1128.

[1859] van Gaal WJ, Clark D, Barlis P, et al. Results of primary percutaneous coronary intervention in a consecutive group of patients with acute ST elevation myocardial infarction at a tertiary Australian centre [J]. Internal Medicine Journal, 2007,37(7): 464-471.

[1860] Van Leer C, Stutz M, Haeberli A, et al. Urokinase plasminogen activator released by alveolar epithelial cells modulates alveolar epithelial repair in vitro [J]. Thrombosis and Haemostasis, 2005,94(6): 1257-1264.

[1861] van Till JWO, Levi M, Bresser P, et al. Early procoagulant shift in the bronchoalveollar compartment of patients with secondary peritonitis [J]. Journal of Infectious Diseases, 2006,194(9): 1331-1339.

[1862] van Veen SQ, Cheung CW, Meijers JCM, et al. Anticoagulant and anti-inflammatory effects after peritoneal lavage with antithrombin in experimental polymicrobial peritonitis [J]. Journal of Thrombosis and Haemostasis, 2006,4(11): 2343-2351.

[1863] van Veen SQ, Levi M, van Vliet AK, et al. Peritoneal lavage with activated protein C alters compartmentalized coagulation and fibrinolysis and improves survival in polymicrobial peritonitis [J]. Critical Care Medicine, 2006,34(11): 2799-2805.

[1864] van Veen SQ, Meijers JCM, Levi M, et al. Effects of intra-abdominal administration of recombinant tissue plasminogen activator on coagulation, fibrinolysis and inflammatory responses in experimental polymicrobial perrronitis [J]. Shock, 2007,27(5): 534-541.

[1865] Van Westerloo DJ, Giebelen IAJ, Meijers JCM, et al. Vagus nerve stimulation inhibits activation of coagulation and fibrinolysis during endotoxemia in rats [J]. Journal of Thrombosis and Haemostasis, 2006,4(9): 1997-2002.

[1866] Vincent JL. Drotrecogin alfa (activated): the treatment for severe sepsis? [J]. Expert Opinion on Biological Therapy, 2007,7(11): 1763-1777.

[1867] Voves C, Wuillemin WA, Zeerleder S. International Society on Thrombosis and Haemostasis score for overt disseminated intravascular coagulation predicts organ dysfunction and fatality in sepsis patients [J]. Blood Coagulation & Fibrinolysis, 2006,17(6): 445-451.

[1868] Wacker P, Wacker R. Thrombolytic therapy in acute pulmonary embolism [J]. Herz, 2005,30(4): 261-268.

[1869] Wada H, Sase T, Yamaguchi M. Hypercoagulant states in malignant lymphoma [J]. Experimental Oncology, 2005,27(3): 179-185.

[1870] Wagner A, MacGregor JM, Berg J, et al. Septic pericarditis in a Yorkshire Terrier [J]. Journal of Veterinary Emergency and Critical Care, 2006,16(2): 136-140.

[1871] Wakahara K, Kobayashi H, Yagyu T, et al. Bikunin suppresses lipopolysaccharide-induced lethality through down-regulation of tumor necrosis factor-alpha and interleukin-1 beta in macrophages [J]. Journal of Infectious Diseases, 2005,191(6): 930-938.

[1872] Wang J, Yin L, Lazar MA. The orphan nuclear receptor Rev-erb alpha regulates circadian expression of plasminogen activator inhibitor type 1 [J]. Journal of Biological Chemistry, 2006,281(45): 33842-33848.

[1873] Wang JN, Yao CT, Yeh CN, et al. Once-daily vs. twice-daily intrapleural urokinase treatment of complicated parapneumonic effusion in paediatric patients: a randomised, prospective study [J]. International Journal of Clinical Practice, 2006,60(10): 1225-1230.

[1874] Wang XQ, Bdeir K, Yarovoi S, et al. Involvement of the urokinase kringle domain in lipopolysaccharide-induced acute lung injury [J]. Journal of Immunology, 2006,177(8): 5550-5557.

[1875] Wang Y, Liu ZK, Chen LQ, et al. Mean-field potential calculations of shock-compressed porous carbon [J]. Physical Review B, 2005,71(5).

[1876] Wang ZY, Aris VM, Ogburn KD, et al. Prostaglandin J2 alters pro-survival and pro-death gene expression patterns and 26 S proteasome assembly in human neuroblastoma cells [J]. Journal of Biological Chemistry, 2006,281(30): 21377-21386.

[1877] Ware LB, Matthay MA, Parsons PE, et al. Pathogenetic and prognostic significance of altered coagulation and fibrinolysis in acute lung injury/acute respiratory distress syndrome [J]. Critical Care Medicine, 2007,35(8): 1821-1828.

[1878] Wesselkamper SC, Case LM, Henning LN, et al. Gene expression changes during the development of acute lung injury - Role of transforming growth factor beta [J]. American Journal of Respiratory and Critical Care Medicine, 2005,172(11): 1399-1411.

[1879] Wiel E, Vallet B, ten Cate H. The endothelium in intensive care [J]. Critical Care Clinics, 2005,21(3): 403-+.

[1880] Woo PCY, Lau SKP, Tsoi HW, et al. SARS coronavirus spike polypeptide DNA vaccine priming with recombinant spike polypeptide from Escherichia coli as booster induces high titer of neutralizing antibody against SARS coronavirus [J]. Vaccine, 2005,23(42): 4959-4968.

[1881] Wygrecka M, Markart P, Ruppert C, et al. Cellular origin of pro-coagulant and (anti)fibrinolytic factors in bleomycin-injured lungs [J]. European Respiratory Journal, 2007,29(6): 1105-1114.

[1882] Wygrecka M, Morty RE, Markart P, et al. Plasminogen activator inhibitor-1 is an inhibitor of factor VII-activating protease in patients with acute respiratory distress syndrome [J]. Journal of Biological Chemistry, 2007,282(30): 21671-21682.

[1883] Xie Q, Gao CF, Shinomiya N, et al. Geldanamycins exquisitely inhibit HGF/SF-mediated tumor cell invasion [J]. Oncogene, 2005,24(23): 3697-3707.

[1884] Xu WP, Neckers L. Targeting the molecular chaperone heat shock protein 90 provides a multifaceted effect on diverse cell signaling pathways of cancer cells [J]. Clinical Cancer Research, 2007,13(6): 1625-1629.

[1885] Yagyu T, Kobayashi H, Matsuzaki H, et al. Enhanced spontaneous metastasis in bikunin-deficient mice [J]. International Journal of Cancer, 2006,118(9): 2322-2328.

[1886] Yates PJ, Nicholson ML. The aetiology and pathogenesis of chronic allograft nephropathy [J]. Transplant Immunology, 2006,16(3-4): 148-157.

[1887] Yende S, Kammerer CM, Angus DC. Bench-to-bedside review: Genetics and proteomics: deciphering gene association studies in critical illness [J]. Critical Care, 2006,10(4).

[1888] Yin SP, Li XH, Meng YH, et al. Tumor-suppressive maspin regulates cell response to oxidative stress by direct interaction with glutathione S-transferase [J]. Journal of Biological Chemistry, 2005,280(41): 34985-34996.

[1889] Yordy JS, Moussa O, Pei HP, et al. SP100 inhibits ETS1 activity in primary endothelial cells [J]. Oncogene, 2005,24(5): 916-931.

[1890] Youinou P. New target antigens for antiendothelial cell antibodies [J]. Immunobiology, 2005,210(10): 789-797.

[1891] Yu M, Yu MM, Ru BG. Expression of an antibody-targeted plasminogen activator in Escherichia coli using chemically induced stress responses [J]. Biotechnology Letters, 2004,26(21): 1629-1634.

[1892] Yuan VW, Bowman JD, Funk DJ, et al. Shock temperature measurement using neutron resonance spectroscopy [J]. Physical Review Letters, 2005,94(12).
[truncated: 1,683,613 more chars]
