# Supplementary material for: Meta-Analysis of Preclinical Studies of Fibrinolytic Therapy for Acute Lung Injury
Source: Front Immunol. 2018 Aug 20;9:1898. doi: 10.3389/fimmu.2018.01898 (PMC6110197; doi:10.3389/fimmu.2018.01898)
Supplement: Supplementary file 3 [file Data_Sheet_3.docx]

**Table S3:** Summary of characteristics in studies included for the meta-analysis

| **Author (year)** | **Start time of drug administration** | **Therapy initiated after ALI induction** | **End-point** | **Follow up time** |
| --- | --- | --- | --- | --- |
| [Enkhbaatar et al. (2004)](file:///C:\Users\Administrator\Desktop\20180514paper%20预防性措施.xlsx#RANGE!_ENREF_16) | 4 h after burn and smoke injury | Yes | 48 h | 44 h |
| [Choi et al. (2007)](file:///C:\Users\Administrator\Desktop\20180514paper%20预防性措施.xlsx#RANGE!_ENREF_14) | 30 min before injection of LPS | Not | 16 h | 16 h |
|  |  | Not | 4 h | 4 h |
| [Choi et al. (2007)*](file:///C:\Users\Administrator\Desktop\20180514paper%20预防性措施.xlsx#RANGE!_ENREF_13) | 30 min before induction of pneumonia | Not | 16 h | 16 h |
|  |  | Not | 6 h | 6 h |
| [Huang et al. (2012)](file:///C:\Users\Administrator\Desktop\20180514paper%20预防性措施.xlsx#RANGE!_ENREF_25) | 15 min before ventilation | Not | 2 h | 2.25 h |
| [Stringer et al. (1998)](file:///C:\Users\Administrator\Desktop\20180514paper%20预防性措施.xlsx#RANGE!_ENREF_44) | half 10 min before IL-1 and half 2.5 h later | Not | 5 h later | 5 h |
| [Hofstra et al. (2013)](file:///C:\Users\Administrator\Desktop\20180514paper%20预防性措施.xlsx#RANGE!_ENREF_23) | 30 min before and at 6 and 12 h after induction of pneumonia or endotoxemia | Not | 16 h after injury | 16 h |
| [Conhaim et al. (2014)](file:///C:\Users\Administrator\Desktop\20180514paper%20预防性措施.xlsx#RANGE!_ENREF_15) | 6 h after blood loss | Yes | at 24 h | 18 h |
| [Veress et al. (2013)](file:///C:\Users\Administrator\Desktop\20180514paper%20预防性措施.xlsx#RANGE!_ENREF_48) | 5.5 and 6.5 h after exposure to SM | Yes | 12/48 h | 41.5 h |
| [Veress et al. (2015)](file:///C:\Users\Administrator\Desktop\20180514paper%20预防性措施.xlsx#RANGE!_ENREF_47) | after 6.5 h of exposure, every 4 h for 48 h | Yes | 48 h | 41.5 h |
| [Renckens et al. (2008)](file:///C:\Users\Administrator\Desktop\20180514paper%20预防性措施.xlsx#RANGE!_ENREF_40) | 24 h before intranasal infection with K. pneumoniae | Not | 24/48 h/10 d | 24/48 h/10 d |
| [Hardaway et al. (1990)](file:///C:\Users\Administrator\Desktop\20180514paper%20预防性措施.xlsx#RANGE!_ENREF_20) | 4 h after trauma | Yes | 48 h | 48 h |
| [Hardaway et al. (1996)](file:///C:\Users\Administrator\Desktop\20180514paper%20预防性措施.xlsx#RANGE!_ENREF_21) | 20 min after the injection of Escherichia coli | Yes | 24 h | 24 h |
| [Vasquez et al. (1998)](file:///C:\Users\Administrator\Desktop\20180514paper%20预防性措施.xlsx#RANGE!_ENREF_46) | urokinase 1 h after heat-killed E. coli infusion | Yes | 24 h | 24 h |
|  | bolus dose and a continuous drip of urokinase 2 h after heat-killed E. coli infusion | Yes | 24 h | 24 h |
| [Munster et al. (2002)](file:///C:\Users\Administrator\Desktop\20180514paper%20预防性措施.xlsx#RANGE!_ENREF_37) | 24 h after trauma | Yes | 48 h | 24 h |
| [Chen et al. (2006)](file:///C:\Users\Administrator\Desktop\20180514paper%20预防性措施.xlsx#RANGE!_ENREF_11) | 2 h after injury | Yes | 6 h | 6 h |
| [Gunther et al. (2003)](file:///C:\Users\Administrator\Desktop\20180514paper%20预防性措施.xlsx#RANGE!_ENREF_17) | 2-12/14-24 day post-bleomycin | Not | 28 d | 28 d |
| [Yu et al. (2014)](file:///C:\Users\Administrator\Desktop\20180514paper%20预防性措施.xlsx#RANGE!_ENREF_52) | after injury, time not reported | Yes | 14 d | 14 d |
| [Chen et al. (2009)](file:///C:\Users\Administrator\Desktop\20180514paper%20预防性措施.xlsx#RANGE!_ENREF_10) | 30 min after injury | Yes | 12 h | 12 h |
| [Sisson et al. (2002)](file:///C:\Users\Administrator\Desktop\20180514paper%20预防性措施.xlsx#RANGE!_ENREF_42) | NR | Not | 28 d | 28 d |
| Hardaway et al. (1964) | 12500 U after initial blood sample, 12500 U after hemolyzed blood, 6250 U every 30min during shock period | Not | 48 h | 48 h |
| Motoyama et al. (2013) | plasmin or placebo was administered upon EVLP initiation | Not | 60 min | 60 min |
| [Motoyama et al. (2014)](file:///C:\Users\Administrator\Desktop\20180514paper%20预防性措施.xlsx#RANGE!_ENREF_35) | plasmin or placebo was administered on EVLP initiation | Not | 3.5 h | 3.5 h |

LPS: lipopolysaccharide, SM: Sulfur mustard, EVLP: ex vivo lung perfusion, NR: not reported
